# Supplementary material for: An umpolung strategy to react catalytic enols with nucleophiles
Source: Nat Commun. 2019 Nov 20;10:5244. doi: 10.1038/s41467-019-13175-5 (PMC6868166; doi:10.1038/s41467-019-13175-5)
Supplement: Supplementary file 2 — Supplementary Information [file 41467_2019_13175_MOESM2_ESM.pdf]

Supplementary Information

An Umpolung Strategy to React Catalytic Enols with Nucleophiles

Sanz-Marco et al.

Supplementary Methods

General information

All reagents were utilized without any further purification as obtained from commercial sources. Flash chromatography was performed with 60 Å (35-70 μm) silica gel (GC 60A 35-70 Micron, DAVISIL). Analytical TLC was performed on aluminum plates pre-coated (0-25 mm) with silica gel (Merck, Silica Gel 60 F254). Compounds were detected by exposure to UV light or by revealing the plates in a solution of 5% $KMnO_4$ in water. Melting points were recorded in metal block and are uncorrected. ^1H and ^{13}C NMR spectra were recorded at 400 MHz and 100 MHz respectively on a Bruker Advance spectrometer. Chemical shifts (δ) are shown in ppm, using as a reference the residual peaks of $CDCl_3$ (δ_H 7.26 and δ_C 77.00). Coupling constants (J) are given in Hz. NMR yields were calculated using 1 equiv. of 1,3,5-trimethoxybenzene as internal standard. High resolution mass spectra (HRMS) were recorded on Brucker microTOF ESI-TOF mass spectrometer.

Substrates **1c**, **1s**, **1v** and **1w** were obtained directly from commercial suppliers. **1a**,[1] **1b**,[2] **1d**,[3] **1e**,[4] **1f**,[4] **1g**,[3] **1h**,[2] **1i**,[2] **1k**,[5] **1o**,[2] **1p**,[6] **1q**,[7] **1r**,[8] **1t**,[2] **1u**,[9] **8a**,[10] **8i**,[10] were synthesized according to methods described in the literature.

Supplementary Table 1. Optimization studies

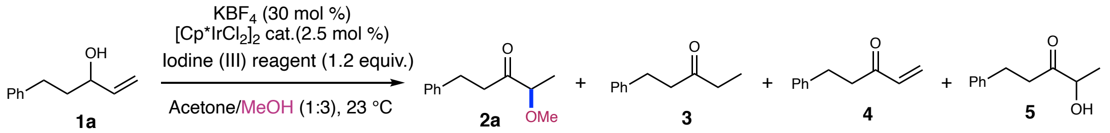

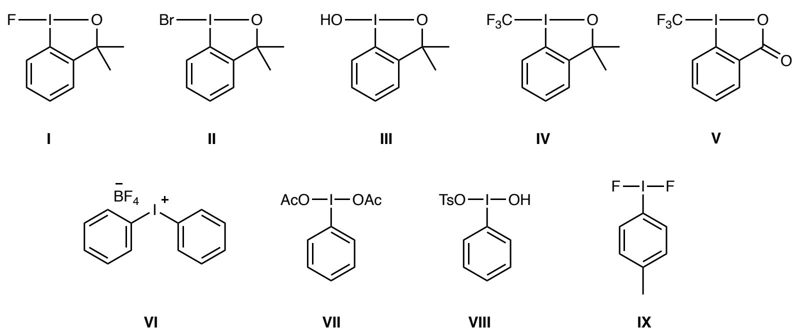

Entry	Iodine (III) Reagent	Yield[a] **2a/3/4/5**
1	**I**	43/12/12/10
2	**II**	-/-/-/-[b]
3	**III**	-/-/-/- [b]
4	**IV**	-/-/-/- [b]
5	**V**	-/23/-/-
6	**VI**	-/89/3/-
7	**VII**	-/21/-/-
8	**VIII**	3/3/68/-
9	**IX**	15/29/5/10

All experiments were carried out under air atmosphere on 0.15 mmol scale of **1a** and 0.2 M, KBF_4 (0.3 equiv.) for 2 h. [a]Determined by ^{1}H NMR spectroscopy against an internal standard (1,2,4,5-tetrachloro-3-nitrobenzene). [b] Decomposition.

Synthesis of 5-phenylpent-1-en-3-*d*-3-ol (1a–*d*)

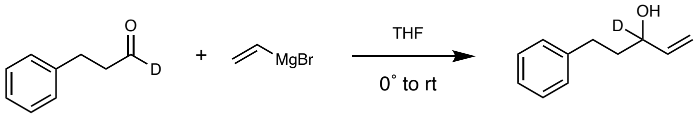

According to a literature procedure,[11] a 1 M solution of vinyl magnesium bromide (1.2 mmol, 1.2 equiv.) was added dropwise to a solution of 3-phenylpropanal-1-*d* (1 mmol, 1 equiv.) in dry THF (10 mL) at 0 °C. The reaction was allowed to warm to room temperature and quenched with NH_4Cl sat. aqueous solution (10 mL). The mixture was then extracted with EtOAc (3 x 10 mL) and the combined organic phases were dried with $MgSO_4$ and reduced under vacuum. The final product was purified using silica chromatography and mixture petroleum ether / EtOAc (1:1) as eluent yielding the desired product as a colorless oil with 68% yield with >99% D.

^{1}H NMR (400 MHz, $CDCl_3$) δ 7.31–7.27 (m, 2H), 7.22–7.18 (m, 3H), 5.91 (dd, J = 17.2, 10.4, 1H), 5.25 (dd, J = 17.2, 1.5 Hz, 1H), 5.15 (dt, J = 10.4, 1.5 Hz, 1H), 2.80–2.66 (m, 2H), 1.90–1.82 (m, 2H). **^{13}C NMR (100 MHz, $CDCl_3$)** δ 142.0, 141.1, 128.6, 128.5, 126.0, 115.1, 72.18 (t, J = 22 Hz), 38.5, 31.7. Characterization in accordance to the previously reported data.[12]

Synthesis of 5-(phenylsulfonyl)pent-1-en-3-ol (1j)

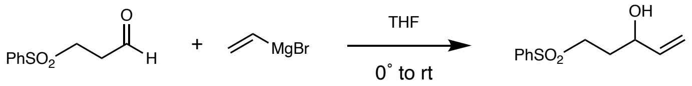

To a solution of 3-(phenylsulfonyl)propanal (1 mmol, 1 equiv.) in dry THF (10 mL) at 0 °C, a 1 M solution of vinyl magnesium bromide (1.2 mmol, 1.2 equiv.) was added dropwise. The reaction was allowed to warm to room temperature and quenched with NH_4Cl sat. aqueous solution (10 mL). The mixture was then extracted with EtOAc (3 x 10 mL) and the combined organic phases were dried with $MgSO_4$ and reduced under vacuum. The final product was purified using silica chromatography and mixture petroleum ether / EtOAc (1:1) as eluent yielding the desired product as a colorless oil with 60% yield.

^{1}H NMR (400 MHz, $CDCl_3$) δ 7.94–7.91 (m, 2H), 7.69–7.64 (m, 1H), 7.60–7.56 (m, 2H), 5.81 (ddd, J = 17.2, 10.4, 5.8 Hz, 1H), 5.25 (dt, J = 17.2, 1.3 Hz, 1H), 5.17 (dt, J = 10.4, 1.3 Hz, 1H), 4.28–4.23 (m, 1H), 3.29–3.17 (m, 2H), 2.07–2.00 (m, 1H), 1.98–1.85 (m, 1H), 1.69 (d, J = 4 Hz, 1H). **^{13}C NMR (100 MHz, $CDCl_3$)** δ 139.6, 133.9, 129.7, 129.5, 128.2, 116.2, 71.0, 52.7, 31.1. **HRMS (ESI)** m/z calcd for $[C_{11}H_{14}O_3S+Na^+]$: 249.0556; found: 249.0554.

Synthesis of 6-hydroxyoct-7-enenitrile (1l)

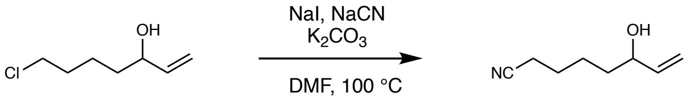

Allylic alcohol 7-chlorohept-1-en-3-ol (0.67 mmol, 1 equiv.), NaI (1 mmol, 1.5 equiv.) and K_2CO_3 (0.67 mmol, 1 equiv.) were dissolved in dry DMF (3 mL) at room temperature. NaCN (0.67 mmol, 1 equiv.) was subsequently added and the mixture was heated at 100 °C during 16 h. The reaction was then allowed to warm to room temperature and quenched with H_2O (3 mL). The mixture was then extracted with EtOAc (3 x 3 mL) and the combined organic phases were dried with $MgSO_4$ and reduced under vacuum. The final product was purified using silica chromatography and mixture petroleum ether / EtOAc (8:2) as eluent yielding the desired product as a colorless oil with 54% yield.

^{1}H NMR (400 MHz, $CDCl_3$) δ 5.86 (ddd, *J* = 16.9, 10.4, 6.3 Hz, 1H), 5.24 (dt, *J* = 17.2, 1.4 Hz, 1H), 5.13 (dt, *J* = 10.4, 1.4 Hz, 1H), 4.15–4.10 (m, 1H), 2.36 (t, *J* = 7 Hz, 2H), 1.73–1.67 (m, 2H), 1.60–1.54 (m, 4H), 1.52 (bs, 1H). **^{13}C NMR (100 MHz, $CDCl_3$)** δ 140.9, 119.8, 115.3, 73.0, 36.1, 25.5, 24.7, 17.3. **HRMS (ESI)** m/z calcd for [$C_8H_{13}O+Na^+$] : 162.0889; found: 162.0898.

Synthesis of allylic alcohol (3*S*,8*R*,9*S*,10*S*,13*S*,14*S*)-10,13-Dimethyl-17-oxohexadecahydro-1*H*-cyclopenta[*a*] phenanthren-3-yl 4-(1-hydroxyallyl)benzoate (1x)

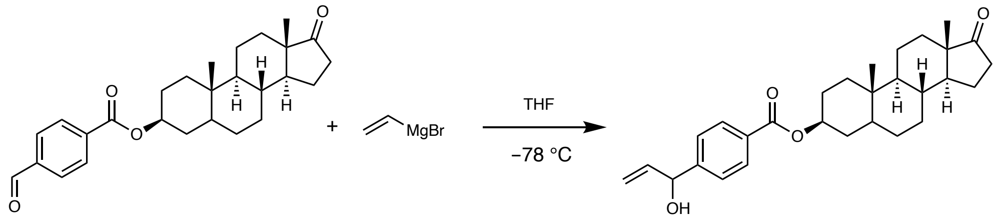

To a solution of (3S,8R,9S,10S,13S,14S)-10,13-dimethyl-17-oxohexadecahydro-1H-cyclopenta[a]phenanthren-3-yl-4-formylbenzoate (1 mmol, 1 equiv.) in dry THF (10 mL) at −78 °C, a 1 M solution of vinyl magnesium bromide (1 mmol, 1 equiv.) was added dropwise. The reaction was subsequently stirred during 3 hours and quenched with NH_4Cl sat. aqueous solution (10 mL). The mixture was then extracted with EtOAc (3 x 10 mL) and the combined organic phases were dried with $MgSO_4$ and reduced under vacuum. The final product was purified using silica chromatography and mixture petroleum ether / EtOAc (8:2) as eluent yielding the desired product as a white solid with 48% yield. m.p = 187–189 °C.

^{1}H NMR (400 MHz, $CDCl_3$) δ 8.01 (d, *J* = 8.4 Hz, 2H), 7.44 (d, *J* = 8.4 Hz, 2H), 6.01 (ddd, *J* = 17.1, 10.3, 6.2 Hz, 1H), 5.36 (dt, *J* = 17.1, 1.3 Hz, 1H), 5.26 (d, *J* = 6.2 Hz, 1H), 5.22 (dt, *J* = 10.3, 1.3 Hz, 1H), 4.98–4.90 (m, 1H), 2.47–2.40 (m, 1H), 2.12–2.03 (m, 1H), 1.97–1.90 (m, 2H), 1.82–1.73 (m, 4H), 1.70–1.48 (m, 7H),

1.39−1.21 (m, 7H), 0.90 (s, 3H), 0.87 (s, 3H). **^{13}C NMR (100 MHz, $CDCl_3$)** δ 221.4, 166.0, 147.4, 139.9, 130.3, 129.9, 126.2, 116.0, 75.2, 74.3, 54.5, 51.5, 48.0, 44.9, 36.9, 36.0, 35.9, 35.2, 34.2, 31.7, 31.0, 28.4, 27.7, 21.9, 20.6, 14.0, 12.4. **HRMS (ESI)** m/z calcd for [$C_{29}H_{38}O_4$+Na^+] : 473.2662; found: 473.2665.

Synthesis of allylic alcohol (8R,9S,10S,13R,14S)-17-((2R)-5-hydroxyhept-6-en-2-yl)-10,13-dimethylhexadecahydro-3H-cyclopenta[a]phenanthren-3-one (1y)

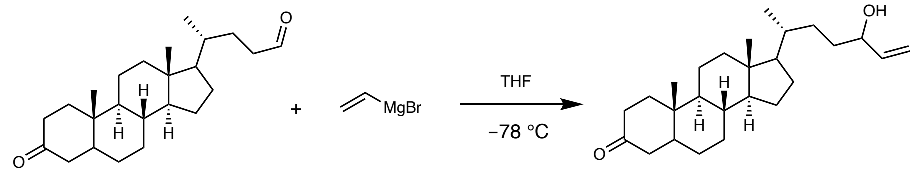

To a solution of (4R)-4-((8R,9S,10S,13R,14S)-10,13-dimethyl-3-oxohexadecahydro-1H-cyclopenta[a]phenanthren-17-yl)pentanal (1 mmol, 1 equiv.) in dry THF (10 mL) at −78 °C, a 1 M solution of vinyl magnesium bromide (1 mmol, 1 equiv.) was added dropwise. The reaction was subsequently stirred during 3 hours and quenched with NH_4Cl sat. aqueous solution (10 mL). The mixture was then extracted with EtOAc (3 x 10 mL) and the combined organic phases were dried with $MgSO_4$ and reduced under vacuum. The final product was purified using silica chromatography and mixture DCM / EtOAc (95:05) as eluent yielding the desired product as a colorless oil with 27% yield as a mixture of two diastereoisomers ca. 1:1.

^{1}H NMR (400 MHz, $CDCl_3$, mixture of diastereoisomers *ca.* 1:1) δ 5.91−5.82 (m, 1H (both diast.)), 5.24−5.19 (m, 1H (both diast.)), 5.12−5.09 (m, 1H (both diast.)), 4.08−4.02 (m, 1H (both diast.)), 2.73−2.66 (m, 1H (both diast.)), 2.38−2.29 (m, 1H (both diast.)), 2.19−2.13 (m, 1H (both diast.)), 2.05−2.00 (m, 2H (both diast.)), 1.92−1.79 (m, 3H (both diast.)), 1.61−1.52 (m, 2H (both diast.)), 1.48−1.37 (m, 11H (both diast.)), 1.27−1.08 (m, 8H (both diast.)), 1.02 (s, 3H (both diast.)), 0.93 (d, J = 6.4 Hz, 3H (both diast.)), 0.68 (s, 3H (both diast.)). **^{13}C NMR (100 MHz, $CDCl_3$, mixture of diastereoisomers *ca.* 1:1)** δ 213.5, 141.6, 141.4, 114.6, 114.4, 73.8, 73.6, 56.5, 56.1, 53.5, 44.4, 42.8, 42.4, 40.8, 40.1, 37.3, 37.1, 35.6, 35.60, 35.59, 34.9, 33.60, 33.55, 31.46, 31.42, 28.30, 28.27, 26.7, 25.8, 24.2, 22.7, 21.2, 18.7, 12.1. **HRMS (ESI)** m/z calcd for [$C_{26}H_{42}O_2$+Na^+] : 409.3077; found: 409.3076.

Synthesis of γ-carbonyl allylic alcohols

To a stirred solution of diisopropylamine (1.70 mL, 12 mmol) in dry THF (15 mL) at −78 °C, n-BuLi was added dropwise (5 mL, 12 mmol, 2.5 M solution in hexanes). After 3 min, the corresponding ketone (10 mmol) in 2 mL of dry THF was added and the resulting solution was stirred 1 h at −78 °C. After that, a solution of the α,β-unsaturated aldehyde (11 mmol) in 2 mL of dry THF was added dropwise and the resulting solution was stirred for another 15 min. The reaction was quenched at −78 °C with a saturated ammonium chloride solution (2 mL) and extracted with EtOAc (3 x 20 mL). The

combined organic phases were dried over $MgSO_4$ and the solvent reduced under vacuum. The resulting product was purified by column chromatography using petroleum ether / EtOAc (9:1) mixture as eluent.

1-Cyclohexyl-3-hydroxy-2-methylpent-4-en-1-one (8b)

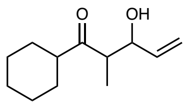

The title compound was synthesized according to the above procedure using 1-cyclohexylpropan-1-one and acrolein as substrates. The final compound was isolated as a yellowish oil with 76% isolated yield as a mixture of two diastereoisomers ca. 75:25.

^{1}H NMR (400 MHz, $CDCl_3$, mixture of diastereoisomers *ca.* 75:25) δ 5.81–5.72 (m, 1H (both diast.)), 5.30–5.23 (m, 1H (both diast.)), 5.17–5.14 (m, 1H (both diast.)), 4.40–4.34 (m, 1H (major diast.)), 4.17–4.15 (m, 1H, (minor diast.)), 2.91 (bs, 1H (both diast.)), 2.81–2.78 (m, 1H (both diast.)), 2.50–2.43 (m, 1H (both diast.)), 1.83–1.64 (m, 5H (both diast.)), 1.34–1.20 (m, 5H (both diast.)), 1.11–1.08 (m, 3H (both diast.)); **^{13}C NMR (100 MHz, $CDCl_3$, mixture of diastereoisomers *ca.* 75:25)** δ 218.5, 138.9, 138.0, 116.5, 115.9, 75.5, 72.6, 51.2, 50.4, 49.2, 48.6, 28.6, 28.6, 28.3, 28.2, 28.2, 26.0, 25.9, 25.8, 25.8, 25.7, 25.6, 14.4, 11.0; **HRMS (ESI)** m/z calcd for [$C_{12}H_{20}O_2$+Na^+] : 219.1356; found: 219.1135.

(*E*)-1-Cyclohexyl-3-hydroxy-2-methylhex-4-en-1-one (8c)

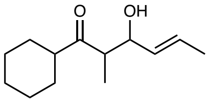

The title compound was synthesized according to the above procedure using 1-cyclohexylpropan-1-one and crotonaldehyde as substrates. The final compound was isolated as a yellowish oil with 49% isolated yield as a mixture of two diastereoisomers ca. 70:30.

^{1}H NMR (400 MHz, $CDCl_3$, mixture of diastereoisomers *ca.* 70:30) δ 5.71–5.64 (m, 1H (both diast.)), 5.45–5.40 (m, 1H (both diast.)), 4.27–4.25 (m, 1H (major diast.)), 4.13-4.09 (m, 1H (minor diast.)), 2.81–2.75 (m, 1H (both diast.)), 2.72 (bs, 1H (both diast)), 2.54–2.42 (m, 1H (both diast.)), 1.82–1.64 (m, 8H (both diast.)), 1.34–1.19 (m, 5H (both diast.)), 1.09 (d, ^3J (^{1}H,^{1}H) = 7.1 Hz, 3H (major diast.)), 1.02 (d, ^3J (^{1}H, ^{1}H) = 7.2 Hz, 3H(minor diast.)); **^{13}C NMR (100 MHz, $CDCl_3$, mixture of diastereoisomers *ca.* 70:30)** δ 218.7, 218.5, 132.0, 131.0, 128.6, 127.9, 75.4, 72.9, 51.2, 50.7, 49.7, 49.1, 28.5, 28.3, 28.3, 28.2, 26.0, 25.9, 25.80, 25.80, 25.75, 25.70, 17.8, 14.50, 14.48, 11.3.; **HRMS (ESI)** m/z calcd for [$C_{13}H_{22}O_2$+Na^+] : 233.1512; found: 233.1524.

5-Hydroxy-4-methylhept-6-en-3-one (8d)

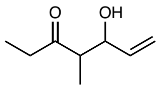

The title compound was synthesized according to the above procedure using pentan-3-one and acrolein as substrates. The final compound was isolated as a colorless oil with 56% isolated yield as a mixture of two diastereoisomers ca. 65:35.

^{1}H NMR (400 MHz, CDCl$_3$, mixture of diastereoisomers *ca.* 65:35) δ 5.84–5.73 (m, 1H (both diast.)), 5.29–5.22 (m, 1H (both diast.)), 5.17–5.13 (m, 1H (both diast.)), 4.41–4.39 (m, 1H (major diast.)), 4.19–4.14 (m, 1H (minor diast.)), 2.83 (bs, 1H (major diast.)), 2.72 (bs, 1H (minor diast.)), 2.70–2.62 (m, 1H (both diast.)), 2.57–2.42 (m, 2H (both diast.)), 1.12–1.00 (m, 6H (both diast.)). **^{13}C NMR (100 MHz, CDCl$_3$, mixture of diastereoisomers *ca.* 65:35)** δ 215.74, 215.67, 138.7, 138.0, 116.7, 116.0, 75.4, 72.7, 50.9, 50.4, 36.3, 35.4, 14.0, 10.9, 7.6, 7.5. **HRMS (ESI)** m/z calcd for [$C_8H_{14}O_2$+Na^+] : 165.0886; found: 165.0883.

5-Ethyl-6-hydroxyoct-7-en-4-one (8e)

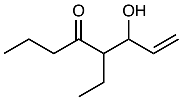

The title compound was synthesized according to the above procedure using heptan-4-one and acrolein as substrates. The final compound was isolated as a colorless oil with 71% isolated yield as a mixture of two diastereoisomers ca. 55:45.

^{1}H NMR (400 MHz, CDCl$_3$, mixture of diastereoisomers *ca.* 55:45) δ 5.85–5.76 (m, 1H (both diast.)), 5.27–5.22 (m, 1H (both diast.)), 5.15–5.12 (m, 1H (both diast.)), 4.30–4.26 (m, 1H (major diast.)), 4.22–4.19 (m, 1H (minor diast.)), 2.60–2.33(m, 4H (both diast.)), 1.60–1.54 (m, 4H (both diast.)), 0.90–0.85 (m, 6H (both diast.)). **^{13}C NMR (100 MHz, CDCl$_3$, mixture of diastereoisomers *ca.* 55:45)** δ 215.8, 214.8, 139.1, 138.2, 116.4, 116.3, 73.9, 73.1, 58.4, 58.0, 47.0, 46.8, 44.8, 22.3, 20.4, 17.4, 16.7, 13.8, 12.3, 11.8. **HRMS (ESI)** m/z calcd for [$C_{10}H_{18}O_2$+Na^+] : 193.1199; found: 193.1195.

3-Hydroxy-4-propylnon-1-en-5-one (8f)

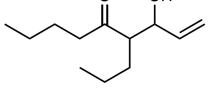

The title compound was synthesized according to the above procedure using nonan-5-one and acrolein as substrates. The final compound was isolated as a colorless oil with 81% isolated yield as a mixture of two diastereoisomers ca. 65:35.

^{1}H NMR (400 MHz, $CDCl_3$, mixture of diastereoisomers *ca.* 65:35) δ 5.84–5.76 (m, 1H (both diast.)), 5.27–5.22 (m, 1H (both diast.)), 5.15–5.13 (m, 1H (both diast.)), 4.27–4.25 (m, 1H (major diast.)), 4.17–4.16 (m, 1H (minor diast.)), 2.72–2.60 (m, 2H (both diast.)), 2.54–2.39 (m, 2H (both diast.)), 1.67–1.48 (m, 4H (both diast.)), 1.33–1.21 (m, 4H (both diast.)), 0.90–0.86 (m, 6H (both diast.)). **^{13}C NMR (100 MHz, $CDCl_3$, mixture of diastereoisomers *ca.* 65:35)** δ 215.7, 214.7, 139.1, 138.0, 116.13, 116.07, 74.1, 73.1, 56.5, 56.3, 44.6, 44.3, 31.3, 29.3, 25.2, 25.1, 22.2, 21,1, 20.6, 14.2, 14.1, 13.8. **HRMS (ESI)** m/z calcd for [$C_{12}H_{22}O_2$+Na^+] : 221.1512; found: 221.1501.

2-(1-Hydroxyallyl)cyclohexan-1-one (8g)

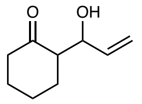

The title compound was synthesized according to the above procedure using cyclohexanone and acrolein as substrates. The final compound was isolated as a colorless oil with 65% isolated yield as a mixture of two diastereoisomers ca. 80:20.

^{1}H NMR (400 MHz, $CDCl_3$, mixture of diastereoisomers *ca.* 80:20) δ 5.83–5.75 (m, 1H (both diast.)), 5.27–5.21 (m, 1H (both diast.)), 5.17–5.11 (m, 1H (both diast.)), 4.57–4.54 (m, 1H (minor diast.)), 4.21 (t, J = 7 Hz, 1H (major diast.)), 3.53 (bs, 1H (major diast.)), 2.85 (bs, 1H (minor diast.)), 2.40–2.25 (m, 3H (both diast.)), 2.08–2.00 (m, 2H (both diast.)), 1.90–1.80 (m, 2H (both diast.)), 1.65–1.56 (m, 1H (both diast.)). **^{13}C NMR (100 MHz, $CDCl_3$, mixture of diastereoisomers *ca.* 80:20)** δ 214.9, 214.1, 137.9, 137.8, 116.9, 115.6, 73.4, 70.6, 55.8, 55.3, 42.7, 42.6, 30.6, 27.8, 27.6, 27.1, 24.9, 24.8. **HRMS (ESI)** m/z calcd for [$C_9H_{14}O_2$+Na^+] : 177.0886; found: 177.0885.

(3*R*,6*S*)-2-((*E*)-1-Hydroxybut-2-en-1-yl)-6-isopropyl-3-methylcyclohexan-1-one (8h)

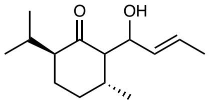

The title compound was synthesized according to the above procedure using (2*S*,5*R*)-2-isopropyl-5-methylcyclohexanone and crotonaldehyde as substrates. The final compound was isolated as a colorless oil with 80% isolated yield.

^{1}H NMR (400 MHz, $CDCl_3$) δ 5.75–5.57 (m, 2H), 4.16–4.11 (m, 1H), 3.41 (d, J = 11 Hz, 1H), 2.14–2.09 (m, 4H), 2.06–1.98 (m, 1H), 1.93–1.88 (m, 1H), 1.67 (d, J = 6 Hz, 3H), 1.52–1.33 (m, 2H), 1.12 (d, J = 6 Hz, 3H), 0.89 (d, J = 6.6 Hz, 3H), 0.85 (d, J = 6.8 Hz, 3H). **^{13}C NMR (100 MHz, $CDCl_3$)** δ 217.3, 133.5, 126.8, 70.9, 62.7,

58.4, 38.6, 34.8, 30.7, 26.1, 21.7, 20.5, 19.0, 17.8. **HRMS (ESI)** m/z calcd for [$C_{14}H_{24}O_2$+Na^+] : 247.1669; found: 247.1678.

3-Hydroxy-*N,N*-2-trimethylpent-4-enamide (8j)

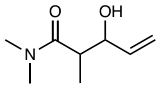

The title compound was synthesized according to the above procedure using *N,N*-dimethylpropionamide and acrolein as substrates. The final compound was isolated as a colorless oil with 73% isolated yield as a mixture of two diastereoisomers ca. 66:33.

^{1}H NMR (400 MHz, $CDCl_3$, mixture of diastereoisomers *ca.* 66:33) δ 5.91−5.75 (m, 1H (both diast.)), 5.39−5.28 (m, 1H (both diast.)), 5.21−5.15 (m, 1H (both diast.)), 4.67 (s, 1H (minor diast.)), 4.48 (s, 1H (major diast)), 4.18−4.12 (m, 1H (both diast.)), 3.05 (s, 3H (both diast.)), 2.96 (s, 3H (both diast.)), 2.82−2.68 (m, 1H (both diast.)), 1.22 (d, *J* = 7 Hz, 3H (minor diast.)), 1.14 (d, *J* = 7 Hz, 3H (major diast.)). **^{13}C NMR (100 MHz, $CDCl_3$, mixture of diastereoisomers *ca.* 66:33)** δ 177.4, 176.1, 139.3, 137.9, 116.2, 115.8, 75.6, 72.2, 40.7, 39.5, 37.50, 37.50, 35.52, 35.50, 14.9, 10.0. **HRMS (ESI)** m/z calcd for [$C_8H_{15}O_2$+Na^+] : 180.0995; found: 180.1004.

***N*-Benzyl-3-hydroxy-*N*-methylpent-4-enamide (8k)**

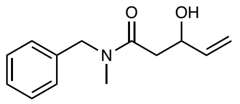

The title compound was synthesized according to the above procedure using *N*-benzyl-*N*-methylacetamide and acrolein as substrates. The final compound was isolated as a colorless oil with 63% isolated yield as a mixture of two rotamers at rt ca. 60:40.

^{1}H NMR (400 MHz, $CDCl_3$, mixture of two rotamers at rt *ca.* 60:40) δ 7.39−7.28 (m, 3H (both rotamers)), 7.26-7.14 (m, 2H (both rotamers)), 5.97−5.82 (m, 1H (both rotamers)), 5.38−5.28 (m, 1H (both rotamers)), 5.18−5.11 (m, 1H (both rotamers)), 4.66−4.51 (m, 2H (minor diast.)), 4.43 (m, 1H (both rotamers), 4.42 (m, 1H (both rotamers), 2.97 (s, 3H (minor rotamer)), 2.91 (s, 3H (major rotamer)), 2.65−2.48 (m, 2H (both rotamer)). **^{13}C NMR (100 MHz, $CDCl_3$, mixture of two rotamers at rt *ca.* 60:40)** δ 172.8, 172.4, 139.25, 139.21, 136.9, 136.1, 129.2, 128.8, 128.1, 128.0, 127.7, 126.4, 115.2, 69.3, 69.2, 53.3, 50.8, 39.7, 39.2, 34.8, 33.9. **HRMS (ESI)** m/z calcd for [$C_{13}H_{17}O_2$+Na^+] : 242.1151; found: 242.1157.

General methods for the synthesis of α-alcoxy ketones and 3(2H)-furanones

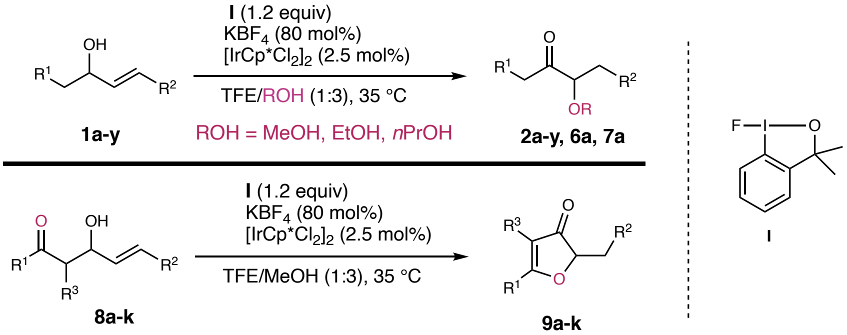

Procedure A. The corresponding allylic alcohol (0.3 mmol, 1 equiv.) was dissolved in a mixture MeOH / TFE (3:1) (15 mL). KBF_4 (30 mg, 0.24 mmol, 80 mol%), 1-fluoro-3,3-dimethyl-1,3-dihydro-1-λ-3-benzo[*d*][1,2]iodaoxole (102 mg, 0.36 mmol, 1.2 equiv.), and $[Cp^*IrCl_2]_2$ (6 mg, 0.0075 mmol, 0.025 equiv.) were added and the mixture stirred at 35 ºC during 2 h. After that, H_2O was added to dilute the reaction and the mixture was extracted with Et_2O (3 x 1 mL). The combined organic phases were dried with $MgSO_4$ and the solvent was evaporated under vacuum. The resulting crude was purified by column chromatography using petroleum ether / EtOAc (90:10) mixture as eluent.

Procedure B. $[Cp^*IrCl_2]_2$ (6 mg, 0.0075 mmol, 0.025 equiv.), KBF_4 (30 mg, 0.24 mmol, 80 mol%) were dissolved in a mixture MeOH / TFE (3:1) (15 mL). A solution of the corresponding allylic alcohol (0.3 mmol, 1 equiv.) in MeOH / TFE (3:1) (3 mL) and a solution of 1-fluoro-3,3-dimethyl-1,3-dihydro-1λ3-benzo[*d*][1,2]iodaoxole (102 mg, 0.36 mmol, 1.2 equiv.) in TFE (3 mL) were both slowly added by means of a syringe pump with a flow of 0.3 mL / h. The resulting solution was heated at 35 ºC during 4 h. Afterwards, the reaction was diluted with H_2O and extracted with Et_2O (3 x 1 mL). The combined organic phases were dried with $MgSO_4$ and the solvent was evaporated under vacuum. The resulting crude was purified by column chromatography using petroleum ether / EtOAc (90:10) mixture as eluent.

4-Methoxy-1-phenylpentan-3-one (2a)

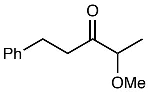

The title compound was prepared according to general procedure A from 5-phenylpent-1-en-3-ol (**1a**, 49 mg). Purification by column chromatography (SiO_2; petroleum ether / ethyl acetate, 90:10) afforded **2a** as a colorless oil (45 mg, 78%).

^{1}H NMR (400 MHz, $CDCl_3$): δ 7.30–7.27 (m, 2H), 7.21–7.17 (m, 3H), 3.71 (q, *J* = 6.9 Hz, 1H), 3.30 (s, 3H), 2.93–2.80 (m, 4H), 1.24 (d, *J* = 6.9 Hz, 3H) ppm. **^{13}C NMR (100 MHz, $CDCl_3$)**: δ 212.1, 141.3, 128.6, 128.5, 126.2, 83.0, 57.6, 39.0, 29.5, 17.0. ppm. **HRMS (ESI)**: m/z calcd for $[C_{12}H_{16}O_2+Na^+]$: 215.1043; found: 215.1030.

4-Methoxy-1-phenylpentan-3-one (2a-*d*)

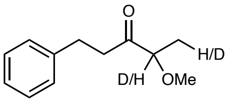

The title compound was prepared according to general procedure A from 5-phenylpent-1-en—3-*d*-3-ol (**1a**, 49 mg). Purification by column chromatography (SiO_2; petroleum ether / ethyl acetate, 90:10) afforded **2a-*d*** as a colorless oil (36 mg, 62%) with 47% D at Cβ and 49% D at Cα.

^{1}H NMR (400 MHz, CDCl$_3$): δ 7.29–7.25 (m, 2H), 7.20–7.17 (m, 3H), 3.70 (7, *J* = 6.8 Hz, 1H), 3.30 (d, *J* = 1.6 Hz, 3H), 2.92–2.82 (m, 4H), 1.22–1.21 (m, 3H) ppm. **^{13}C NMR (100 MHz, CDCl$_3$)**: δ 212.2, 141.3, 128.6, 128.5, 126.2, 83.0, 57.6, 39.0, 29.4, 16.9 ppm. **HRMS (ESI)**: m/z calcd for [$C_{12}H_{15}DO_2$+Na^+] : 216.1105; found: 216.1092.

2-Methoxy-7-phenylheptan-3-one (2b)

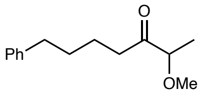

The title compound was prepared according to general procedure A from 7-phenylhept-1-en-3-ol (**1b**, 57 mg). Purification by column chromatography (SiO_2; petroleum ether / ethyl acetate, 90:10) afforded **2b** as a colorless oil (44 mg, 66%).

^{1}H NMR (400 MHz, CDCl$_3$): δ 7.29–7.26 (m, 2H), 7.19–7.16 (m, 3H), 3.72 (q, *J* = 6.9 Hz, 1H), 3.34 (s, 3H), 2.65–2.61 (m, 2H), 2.56–2.53 (m, 2H), 1.65–1.61 (m, 4H), 1.28 (d, *J* = 6.9 Hz, 3H) ppm. **^{13}C NMR (100 MHz, CDCl$_3$):** δ 212.8, 142.2, 128.4, 128.3, 125.7, 82.8, 57.5, 36.9, 35.8, 31.1, 22.8, 17.1 ppm. **HRMS (ESI)**: m/z calcd for [$C_{14}H_{20}O_2$+Na^+] : 243.1356; found: 243.1368.

2-Methoxyoctan-3-one (2c)

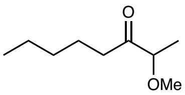

The title compound was prepared according to general procedure A from oct-1-en-3-ol (**1c**, 38 mg). Purification by column chromatography (SiO_2; petroleum ether / ethyl acetate, 90:10) afforded **2c** as a colorless oil (21 mg, 45%).

^{1}H NMR (400 MHz, CDCl$_3$): δ 3.73 (q, *J* = 6.9 Hz, 1H), 3.35 (s, 3H), 2.54–2.49 (m, 2H), 1.62–1.54 (m, 2H), 1.34–1.24 (m, 7H), 0.89 (t, *J* = 7.0 Hz, 3H) ppm. **^{13}C NMR (100 MHz, CDCl$_3$):** δ 213.3, 83.0, 57.7, 37.3, 31.6, 23.1, 22.6, 17.3, 14.1. ppm. **HRMS (ESI)**: m/z calcd for [$C_9H_{18}O_2$+Na^+] : 181.1199; found: 181.1196.

4-Ethyl-2-methoxyoctan-3-one (2d)

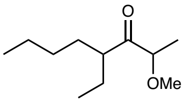

The title compound was prepared according to general procedure A from 4-ethyloct-1-en-3-ol (**1d**, 47mg). Purification by column chromatography (SiO_2; petroleum ether / ethyl acetate, 90:10) afforded **2d** as a colorless oil (20 mg, 36% as a mixture of two diastereoisomers 1:1).

^{1}H NMR (400 MHz, $CDCl_3$, mixture of two diastereoisomers ca. 1:1): δ 3.83 (q, J = 6.9 Hz, 1H), 3.36 (s, 3H), 2.72–2.68 (m, 1H), 1.70–1.56 (m, 2H), 1.48–1.16 (m, 11H), 0.90–0.82 (m, 6H). ppm. **^{13}C NMR (100 MHz, $CDCl_3$, mixture of two diastereoisomers ca. 1:1):** δ 215.09, 215.07, 82.31, 82.28, 57.59, 57.58, 48.7, 48.6, 31.4, 30.3, 29.9, 29.7, 24.9, 23 .9, 23.00, 22.98, 16.7, 16.6, 14.10, 14.08, 12.2, 11.9 ppm. **HRMS (ESI)**: m/z calcd for [$C_{11}H_{22}O_2$+Na^+] : 209.1512; found: 209.1506.

2-Methoxy-1-phenylpropan-1-one (2e)

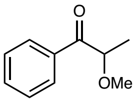

The title compound was prepared according the general procedure from 1-phenylprop-2-en-1-ol (**1e**, 40 mg) according to general procedure B. Purification by column chromatography (SiO_2; petroleum ether / ethyl acetate, 90:10) afforded **2e** as a colorless oil (28 mg, 58%).

^{1}H NMR (400 MHz, $CDCl_3$): δ 8.06–8.04 (m, 2H), 7.58–7.56 (m, 1H), 7.49–7.46 (m, 2H), 4.63 (q, J = 6.9 Hz, 1H), 3.39 (s, 3H), 1.49 (d, J = 6.9 Hz, 3H). ppm. **^{13}C NMR (100 MHz, $CDCl_3$):** δ 200.7, 135.0, 133.5, 128.9, 128.8, 80.4, 57.4, 18.6. ppm. **HRMS (ESI)**: m/z calcd for [$C_{10}H_{12}O_2$+Na^+] : 187.0730; found: 187.0731.

2-Methoxy-5,9-dimethyldec-8-en-3-one (2f)

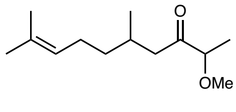

The title compound was prepared according to general procedure A from 7,11-dimethyldodeca-1,10-dien-3-ol (**1f**, 55 mg). Purification by column chromatography (SiO_2; petroleum ether / ethyl acetate, 90:10) afforded **2f** as a colorless oil (43 mg, 67% as a mixture of two diastereoisomers ca. 1:1).

^{1}H NMR (400 MHz, $CDCl_3$, mixture of two diastereoisomers ca. 1:1): δ 5.10–5.07 (m, 1H), 3.73–3.68 (m, 1H), 3.35 (s, 3H), 2.53–2.31 (m, 2H), 2.09–2.00 (m, 3H), 1.68 (s, 3H), 1.60 (s, 3H), 1.36–1.16 (m, 5H), 0.91–0.88 (m, 3H). ppm. **^{13}C NMR**

(100 MHz, $CDCl_3$, mixture of two diastereoisomers ca. 1:1): δ 212.7, 212.6, 131.6, 124.5, 83.2, 83.1, 57.66, 57.63, 44.8, 44.7, 37.18, 37.15, 28.27, 28.21, 25.8, 25.7, 20.01, 19.96, 17.8, 17.2, 17.1. ppm. **HRMS (ESI)**: m/z calcd for $C_{13}H_{24}O_2$+Na^+] : 235.1669; found: 235.1677.

1-(Benzyloxy)-3-methoxybutan-2-one (2g)

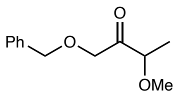

The title compound was prepared according to general procedure A from 1-(benzyloxy)but-3-en-2-ol (**1g**, 54 mg). Purification by column chromatography (SiO_2; petroleum ether / ethyl acetate, 90:10) afforded **2g** as a colorless oil (56 mg, 90%).

^{1}H NMR (400 MHz, $CDCl_3$): δ 7.39–7.34 (m, 5H), 4.63 (s, 2H), 4.44–4.32 (m, 2H), 3.93 (q, *J* = 6.9 Hz, 1H), 3.36 (s, 3H), 1.34 (d, *J* = 6.9 Hz, 3H) ppm. **^{13}C NMR (100 MHz, $CDCl_3$):** δ 208.9, 137.1, 128.5, 128.0, 127.9, 81.3, 79.3, 72.0, 57.5, 16.8 ppm. **HRMS (ESI)**: m/z calcd for $C_{12}H_{16}O_3$+Na^+] : 231.0992; found: 231.0989.

5-((*Tert*-butyldimethylsilyl)oxy)-2-methoxyhexan-3-one (2h)

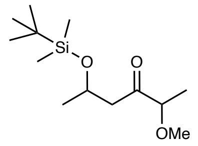

The title compound was prepared according to general procedure A from 5-((*tert*-butyldimethylsilyl)oxy)hex-1-en-3-ol (**1h**, 69 mg). Purification by column chromatography (SiO_2; petroleum ether / ethyl acetate, 90:10) afforded **2h** as a colorless oil (56 mg, 72% as a mixture of two diastereoisomers ca. 1:1).

^{1}H NMR (400 MHz, $CDCl_3$ as mixture of two diastereoisomers ca. 1:1): δ 4.40–4.32 (m, 1H), 3.75–3.68 (m, 1H), 3.36 (s, 3H), 2.86–2.78 (m, 1H), 2.49–2.39 (m, 1H), 1.27 (d, *J* = 6.9 Hz, 3H), 1.18–1.16 (m, 3H), 0.85 (s, 9H), 0.07 (s, 3H), 0.04 (s, 3H). ppm. **^{13}C NMR (100 MHz, $CDCl_3$ as mixture of two diastereoisomers ca. 1:1):** δ 211.1, 211.0, 83.3, 83.2, 64.9, 57.64, 57.62, 47.3, 47.2, 26.0, 24.3, 24.1, 18.1, 16.7, –4.40, –4.45, –4.7. ppm. **HRMS (ESI)**: m/z calcd for $C_{13}H_{28}O_3Si$+Na^+] : 283.1700; found: 283.1709.

6-Methoxyheptane-2,5-dione (2i)

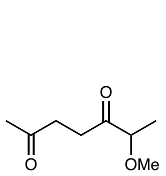

The title compound was prepared according to general procedure A from 1-cyclohexyl-3-hydroxypent-4-en-1-one (**1i**, 38 mg). Purification by column

chromatography (SiO_2; petroleum ether / DCM, 80:20) afforded **2i** as a colorless oil (15 mg, 31%).

^{1}H NMR (400 MHz, $CDCl_3$): δ 3.80 (q, *J* = 6.9 Hz, 1H), 3.39 (s, 3H), 2.81-2.72 (m, 4H), 2.19 (s, 3H), 1.32 (d, *J* = 6.9 Hz, 3H). **^{13}C NMR (100 MHz, $CDCl_3$):** δ 211.7, 207.2, 82.9, 57.8, 36.7, 31.2, 30.1, 17.4. ppm. **HRMS (ESI)**: m/z calcd for [$C_8H_{14}O_3$+Na^+] : 181.0835; found: 181.0828.

4-Methoxy-1-(phenylsulfonyl)pentan-3-one (2j)

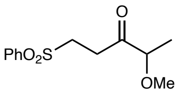

The title compound was prepared according to general procedure A from 5-(phenylsulfonyl)pent-1-en-3-ol (**1j**, 68 mg). Purification by column chromatography (SiO_2; petroleum ether / ethyl acetate, 70:30) afforded **2j** as a colorless oil (53 mg, 69%).

^{1}H NMR (400 MHz, $CDCl_3$): δ 7.94–7.92 (m, 2H), 7.70–7.65 (m, 1H), 7.61–7.57 (m, 2H), 3.75 (q, *J* = 6.8 Hz, 1H), 3.39 (t, *J* = 7.0 Hz, 2H), 3.35 (s, 3H), 3.09-3.05 (m, 2H), 2.19 (s, 3H), 1.27 (d, *J* = 6.8 Hz, 3H). **^{13}C NMR (100 MHz, $CDCl_3$):** δ 208.6, 139.2, 134.0, 129.5, 128.1, 82.6, 57.8, 50.4, 30.5, 16.9 ppm. **HRMS (ESI)**: m/z calcd for [$C_{12}H_{16}O_4S$+Na^+] : 279.0662; found: 279.0663.

7-Chloro-2-methoxyheptan-3-one (2k)

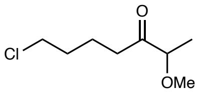

The title compound was prepared according to general procedure A from 7-chlorohept-1-en-3-ol (**1k**, 44 mg). Purification by column chromatography (SiO_2; petroleum ether / acetone, 95:05) afforded **2k** as a colorless oil (28 mg, 53%).

^{1}H NMR (400 MHz, $CDCl_3$): δ 3.73 (q, *J* = 6.8 Hz, 1H), 3.54 (t, *J* = 6.4 Hz, 2H), 3.36 (s, 3H), 2.60–2.54 (m, 2H), 1.83–1.71 (m, 2H), 1.29 (d, *J* = 6.8 Hz, 3H). **^{13}C NMR (100 MHz, $CDCl_3$):** δ 212.4, 83.0, 57.7, 44.8, 36.3, 32.2, 20.7, 17.1 ppm. **HRMS (ESI)**: m/z calcd for [$C_8H_{15}O_2{}^{35}Cl$+Na^+] : 201.0653; found: 201.0643.

7-Methoxy-6-oxooctanenitrile (2l)

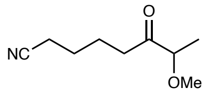

The title compound was prepared according to general procedure A from 6-hydroxyoct-7-enenitrile (**1l**, 42 mg). Purification by column chromatography (SiO_2; petroleum ether / DCM, 60:40) afforded **2l** as a colorless oil (45 mg, 88%).

^{1}H NMR (400 MHz, CDCl$_3$): δ 3.72 (q, *J* = 6.8 Hz, 1H), 3.36 (s, 3H), 2.62–2.57 (m, 2H), 2.36 (t, *J* = 6.9 Hz, 2H), 1.77–1.65 (m, 2H), 1.28 (d, *J* = 6.8 Hz, 3H). **^{13}C NMR (100 MHz, CDCl$_3$):** δ 212.0, 119.6, 82.9, 57.7, 36.1, 25.1, 22.3, 17.3, 17.1 ppm. **HRMS (ESI)**: m/z calcd for [$C_9H_{15}NO_2$+Na^+] : 192.0995; found: 192.0997.

7-Azido-2-methoxyheptan-3-one (2m)

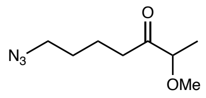

The title compound was prepared according to general procedure A from 7-chlorohept-1-en-3-ol (**1k**, 44 mg). After the reaction was finished, the mixture was evaporated under reduce pressure and redissolved in dry DMF (1 mL). NaI (0.45 mmol, 1 equiv.), K_2CO_3 (0.3 mmol, 1 equiv.) and NaN_3 (0.3 mmol, 1 equiv.) were added and the mixture was heated at 100 °C during 16 h. The reaction was then allowed to warm to room temperature and quenched with H_2O (3 mL). The mixture was then extracted with EtOAc (3 x 3 mL) and the combined organic phases were dried with $MgSO_4$ and reduced under vacuum. Purification by column chromatography (SiO_2; petroleum ether / ethyl acetate, 80:20) afforded **2m** as a colorless oil (36 mg, 65%).

^{1}H NMR (400 MHz, CDCl$_3$): δ 3.73 (q, *J* = 6.8 Hz, 1H), 3.36 (s, 3H), 3.29 (t, *J* = 6.6 Hz, 2H), 2.63–2.53 (m, 2H), 1.70–1.58 (m, 2H), 1.29 (d, *J* = 6.8 Hz, 3H). **^{13}C NMR (100 MHz, CDCl$_3$):** δ 212.5, 83.0, 57.7, 51.4, 36.6, 28.6, 20.5, 17.2 ppm. **HRMS (ESI)**: m/z calcd for [$C_8H_{15}N_3O_2$+Na^+] : 208.1056; found: 208.1062.

2-Methoxy-7-morpholinoheptan-3-one (2n)

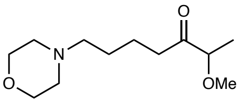

The title compound was prepared according to general procedure A from 7-chlorohept-1-en-3-ol (**1k**, 44 mg). After the reaction was finished, the mixture was evaporated under reduce pressure and redissolved in dry DMF (1 mL). NaI (0.45 mmol, 1 equiv.), K_2CO_3 (0.3 mmol, 1 equiv.) and morpholine (0.3 mmol, 1 equiv.) were added and the mixture was heated at 100 °C during 16 h. The reaction was then allowed to warm to room temperature and quenched with H_2O (3 mL). The mixture was then extracted with EtOAc (3 x 3 mL) and the combined organic phases were dried with $MgSO_4$ and reduced under vacuum. Purification by column chromatography (SiO_2; petroleum ether / ethyl acetate, 40:60) afforded **2n** as a colorless oil (48 mg, 70%).

^{1}H NMR (400 MHz, CDCl$_3$): δ 3.67–3.63 (m, 3H), 3.58–3.56 (m, 1H), 3.40–3.34 (m, 1H), 3.34 (s, 3H), 2.57–2.52 (m, 2H), 2.43–2.40 (m, 4H), 2.35–2.31 (m, 2H), 1.63–1.56 (m, 2H), 1.52–1.43 (m, 2H), 1.28 (d, *J* = 6.8 Hz, 3H). **^{13}C NMR (100 MHz, CDCl$_3$):** δ 212.8, 83.0, 67.1, 53.8, 46.0, 40.8, 37.0, 26.2, 21.2, 17.2. ppm. **HRMS (ESI)**: m/z calcd for [$C_{12}H_{24}NO_3$+H^+] : 230.1751; found: 230.1756.

3-Methoxyoctan-2-one (2o)

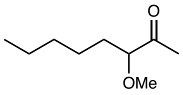

The title compound was prepared according to general procedure A from (*E*)-oct-3-en-2-ol (**1o**, 38 mg). Purification by column chromatography (SiO_2; petroleum ether / ethyl acetate, 90:10) afforded **2o** as a colorless oil (37 mg, 79%).

^{1}H NMR (400 MHz, $CDCl_3$): δ 3.54 (t, *J* = 6.7 Hz, 1H), 3.35 (s, 3H), 2.15 (s, 3H), 1.64–1.56 (m, 2H), 1.36–1.25 (m, 6H), 0.88 (t, *J* = 6.3 Hz, 3H). **^{13}C NMR (100 MHz, $CDCl_3$):** δ 211.7, 87.8, 58.2, 32.0, 31.8, 25.2, 24.9, 22.6, 14.1 ppm. **HRMS (ESI)**: m/z calcd for [$C_9H_{18}O_2$+Na^+] : 181.1199; found: 181.1189.′12′pelp

3-Methoxy-6-phenylhexan-2-one (2p)

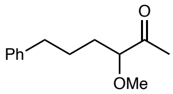

The title compound was prepared according to general procedure A from (*E*)-6-phenylhex-3-en-2-ol (**1p**, 53 mg). Purification by column chromatography (SiO_2; petroleum ether / ethyl acetate, 90:10) afforded **2p** as a colorless oil (47 mg, 76%).

^{1}H NMR (400 MHz, $CDCl_3$): δ 7.30–7.26 (m, 2H), 7.20–7.15 (m, 3H), 3.57–3.54 (m, 1H), 3.34 (s, 3H), 2.64–2.61 (m, 2H), 2.13 (s, 3H), 1.74–1.59 (m, 4H) ppm. **^{13}C NMR (100 MHz, $CDCl_3$):** δ 211.5, 141.8, 128.4, 128.3, 125.9, 87.3, 58.1, 35.6, 31.3, 26.8, 25.1 ppm. **HRMS (ESI)**: m/z calcd for $C_{13}H_{18}O_2$+Na^+] : 229.1199; found: 229.1195.

4-Methoxy-7-phenylheptan-3-one (2q)

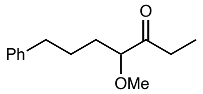

The title compound was prepared according to general procedure A from (*E*)-7-phenylhept-4-en-3-ol (**1q**, 57 mg). Purification by column chromatography (SiO_2; petroleum ether / ethyl acetate, 90:10) afforded **2q** as a colorless oil (30 mg, 45%).

^{1}H NMR (400 MHz, $CDCl_3$): δ 7.29–7.26 (m, 2H), 7.19–7.15 (m, 3H), 3.62–3.59 (m, 1H), 3.33 (s, 3H), 2.64–2.60 (m, 2H), 2.53–2.47 (m, 2H), 1.74–1.63 (m, 4H), 1.04 (t, *J* = 7.3 Hz, 3H) ppm. **^{13}C NMR (100 MHz, $CDCl_3$):** δ 213.9, 142.0, 128.53, 128.49, 126.0, 87.2, 58.4, 35.8, 31.8, 30.9, 27.09, 7.4. ppm. **HRMS (ESI)**: m/z calcd for [$C_{14}H_{20}O_2$+Na^+] : 243.1356; found: 243.1342.

4-Methoxy-1-phenylhexan-3-one (2r)

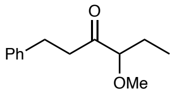

The title compound was prepared according to general procedure A from (*E*)-1-phenylhex-4-en-3-ol (**1r**, 53 mg). Purification by column chromatography (SiO_2; petroleum ether / ethyl acetate, 90:10) afforded **2r** as a colorless oil (40 mg, 65%).

^{1}H NMR (400 MHz, $CDCl_3$): δ 7.28–7.25 (m, 2H), 7.21–7.18 (m, 3H), 3.50 (t, *J* = 6.2 Hz, 1H), 3.29 (s, 3H), 2.92–2.89 (m, 2H), 2.85–2.81 (m, 2H), 1.66–1.59 (m, 2H), 0.88 (t, *J* = 7.4 Hz, 3H) ppm. **^{13}C NMR (100 MHz, $CDCl_3$):** δ 212.2, 141.1, 128.43, 128.40, 126.1, 88.4, 58.1, 39.3, 29.2, 24.8, 9.4 ppm. **HRMS (ESI)**: m/z calcd for [$C_{13}H_{18}O_2$+Na^+] : 229.1199; found: 229.1201.

2-Methoxycyclohexan-1-one (2s)

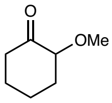

The title compound was prepared according to general procedure A from cyclohex-2-en-1-ol (**1s**, 29 mg). Purification by column chromatography (SiO_2; petroleum ether / ethyl acetate, 90:10) afforded **2s** as a colorless oil (23mg, 60%).

^{1}H NMR (400 MHz, $CDCl_3$): δ 3.72–3.68 (m, 1H), 3.41 (s, 3H), 2.54–2.48 (m, 1H), 2.32–2.19 (m, 2H), 1.97–1.91 (m, 2H), 1.74–1.63 (m, 3H) ppm. **^{13}C NMR (100 MHz, $CDCl_3$):** δ 210.1, 84.4, 57.8, 40.7, 34.3, 27.8, 23.2. ppm. **HRMS (ESI)**: m/z calcd for [$C_7H_{12}O_2$+Na^+] : 151.0730; found: 151.0731.

3-Methoxy-6,10-dimethylundec-9-en-2-one (2t)

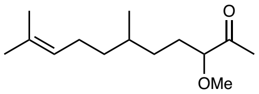

The title compound was prepared according to general procedure A from (*E*)-6,10-dimethylundeca-3,9-dien-2-ol (**1t**, 59 mg). Purification by column chromatography (SiO_2; petroleum ether / ethyl acetate, 90:10) afforded **2t** as a colorless oil (55 mg, 81% as a mixture of two diastereoisomers ca. 1:1).

^{1}H NMR (400 MHz, $CDCl_3$, mixture of two diastereoisomers ca. 1:1): δ 5.07 (t, *J* = 7.0 Hz, 1H), 3.53–3.49 (m, 1H), 3.34 (s, 3H), 2.15 (s, 3H), 2.02–1.91 (m, 2H), 1.67 (s, 3H), 1.65–1.61 (m, 2H), 1.59 (s, 3H), 1.42–1.28 (m, 3H), 1.20–1.09 (m, 2H), 0.86 (d, *J* = 6.4 Hz, 3H) ppm. **^{13}C NMR (100 MHz, $CDCl_3$, mixture of two diastereoisomers ca. 1:1):** δ 211.74, 211.72, 131.3, 124.9, 88.0, 87.9, 58.23, 58.20, 37.0, 36.9, 32.5, 32.3, 32.2, 32.1, 29.6, 25.8, 25.6, 25.3, 25.2, 19.53, 19.48, 17.8 ppm. **HRMS (ESI)**: m/z calcd for [$C_{14}H_{26}O_2$+Na^+] : 249.1825; found: 249.1828.

4-Methoxy-1,7-diphenylheptan-3-one (2u)

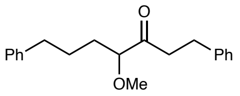

The title compound was prepared according to general procedure A from (*E*)-1,7-diphenylhept-4-en-3-ol (**1u**, 80 mg). Purification by column chromatography (SiO_2; petroleum ether / ethyl acetate, 90:10) afforded **2u** as a colorless oil (59 mg, 66%).

^{1}H NMR (400 MHz, $CDCl_3$): δ 7.28–7.24 (m, 4H), 7.19–7.16 (m, 4H), 7.14–7.12 (m, 2H), 3.56–3.54 (m, 1H), 3.26 (s, 3H), 2.89–2.87 (m, 2H), 2.81–2.79 (m, 2H), 2.58–2.55 (m, 2H), 1.59–1.56 (m, 4H) ppm. **^{13}C NMR (100 MHz, $CDCl_3$):** δ 212.1, 141.8, 141.7, 128.42, 128.40, 128.36, 128.3, 126.1, 125.8, 87.1, 58.1, 39.2, 35.5, 31.2, 29.2, 26.8 ppm. **HRMS (ESI)**: m/z calcd for [$C_{20}H_{24}O_2$+Na^+] : 319.1669; found: 319.1681.

2-Methoxy-5-phenylpentanal (2v)

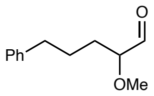

The title compound was prepared according to general procedure A from (*E*)-5-phenylpent-2-en-1-ol (**1v**, 49 mg). Purification by column chromatography (SiO_2; petroleum ether / ethyl acetate, 90:10) afforded **2v** as a colorless oil (36 mg, 62%).

^{1}H NMR (400 MHz, $CDCl_3$): δ 9.64 (d, *J* = 2.0 Hz, 1H), 7.31–7.28 (m, 2H), 7.20–7.16 (m, 3H), 3.58–3.55 (m, 1H), 3.43 (s, 3H), 2.66–2.62 (m, 2H), 1.78–1.67 (m, 4H) ppm. **^{13}C NMR (100 MHz, $CDCl_3$):** δ 204.0, 141.8, 128.52, 128.50, 126.1, 85.8, 58.4, 35.8, 29.5, 26.6. ppm. **HRMS (ESI)**: m/z calcd for [$C_{12}H_{15}O_2$+CH_3OH+Na^+] : 247.1305; found: 247.1309.

2-Methoxyoctanal (2w)

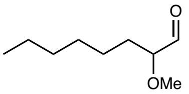

The title compound was prepared according to general procedure A from (*E*)-oct-2-en-1-ol (**1w**, 38 mg). Purification by column chromatography (SiO_2; petroleum ether / ethyl acetate, 90:10) afforded **2w** as a colorless oil (31 mg, 65%).

^{1}H NMR (400 MHz, $CDCl_3$): δ 3.56–3.53 (m, 1H), 3.35 (s, 3H), 1.67–1.58 (m, 2H), 1.41–1.25 (m, 8H), 0.88 (t, *J* = 7 Hz, 3H). ppm. **^{13}C NMR (100 MHz, $CDCl_3$):** δ 211.7, 87.8, 58.2, 32.0, 31.8, 25.2, 24.9, 22.6, 14.1.ppm. **HRMS (ESI)**: m/z calcd for [$C_{10}H_{22}O_3$+CH_3OH+Na^+] : 213.1467; found: 213.1461.

(3*S*,8*R*,9*S*,10*S*,13*S*,14*S*)-10,13-Dimethyl-17-oxohexadecahydro-1*H*-cyclopenta [*a*]phenanthren-3-yl 4-(2-methoxypropanoyl)benzoate (2x)

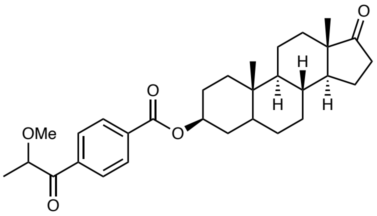

The title compound was prepared according to general procedure B from (3S,8R,9S,10S,13S,14S)-10,13-dimethyl-17-oxohexadecahydro-1H-cyclopenta[a] phenanthren-3-yl 4-(1-hydroxyallyl)benzoate (**1x**, 135 mg). Purification by column chromatography (SiO_2; petroleum ether / ethyl acetate, 90:10) afforded **2x** as a white solid (94 mg, 65%). m. p. 152−154 °C.

^{1}H NMR (400 MHz, $CDCl_3$) δ 8.15−8.06 (m, 4H), 5.01−4.93 (m, 1H), 4.59 (q, *J* = 6.9 Hz, 1H), 3.39 (s, 3H), 2.48−2.41 (m, 1H), 2.13−2.03 (m, 1H), 2.00−1.91 (m, 2H), 1.83−1.75 (m, 4H), 1.72−1.53 (m, 7H), 1.49 (d, *J* = 6.9 Hz, 3H), 1.38−1.29 (m, 7H), 0.91 (s, 3H), 0.87 (s, 3H). **^{13}C NMR (100 MHz, $CDCl_3$)** δ 221.4, 200.5, 165.3, 138.0, 134.9, 129.9, 128.8, 80.8, 74.9, 57.5, 54.5, 51.5, 47.9, 44.8, 36.9, 35.8, 35.2, 34.1, 31.7, 31.0, 29.8, 28.4, 27.6, 21.9, 20.6, 18.3, 14.0, 12.4. **HRMS (ESI)** m/z calcd for [$C_{30}H_{40}O_5$+Na^+] : 503.2768; found: 503.2777.

(8*R*,9*S*,10*S*,13*R*,14*S*)-17-((2*R*)-6-Methoxy-5-oxoheptan-2-yl)-10,13-dimethylhexa decahydro-3*H*-cyclopenta[*a*]phenanthren-3-one (2y)

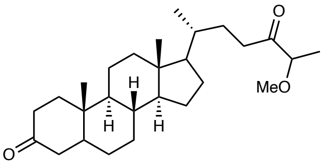

The title compound was prepared according to general procedure A from (8R,9S,10S,13R,14S)-17-((2R)-5-hydroxyhept-6-en-2-yl)-10,13-dimethylhexadeca hydro-3H-cyclopenta[a]phenanthren-3-one (**1y**, 116 mg). Purification by column chromatography (SiO_2; petroleum ether / ethyl acetate, 95:05) afforded **2y** as a colorless oil (77 mg, 62% as a mixture of two diastereoisomers *ca.* 60:40).

^{1}H NMR (400 MHz, $CDCl_3$, mixture of diastereoisomers *ca.* 1:1) δ 3.73 (q, *J* = 6.9 Hz, 1H (one diast.)), 3.72 (q, *J* = 6.9 Hz, 1H (one diast.)), 3.35 (s, 3H (both diast.)), 2.72−2.65 (m, 1H (both diast.)), 2.56−2.41 (m, 2H (both diast.)), 2.37−2.28 (m, 1H (both diast.)), 2.17−2.12 (m, 1H (both diast.)), 2.04−2.00 (m, 3H (both diast.)), 1.91−1.78 (m, 3H (both diast.)), 1.47−1.36 (m, 7H (both diast.)), 1.29−1.20 (m, 8H (both diast.)), 1.16−1.07 (m, 5H (both diast.)), 1.01 (s, 3H (both diast.)), 0.92 (d, *J* = 6.4 Hz, 3H (both diast.)), 0.67 (s, 3H (both diast.)). **^{13}C NMR (100 MHz, $CDCl_3$, mixture of diastereoisomers *ca.* 1:1)** δ 214.0, 213.86, 213.85, 83.0, 82.9, 57.73, 57.72, 56.47, 56.00, 55.96, 44.4, 42.8, 42.5, 40.7, 40.1, 37.4, 37.1, 35.7, 35.5, 35.40, 35.38, 35.0, 34.1, 34.0, 29.8, 29.3, 28.3, 26.7, 25.8, 24.3, 22.8, 21.3, 18.6, 17.4, 17.3, 12.2. **HRMS (ESI)** m/z calcd for [$C_{27}H_{44}O_3$+Na^+] : 439.3183; found: 439.3185.

4-Ethoxy-1-phenylpentan-3-one (6a)

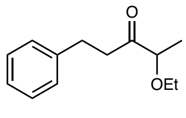

The title compound was prepared according to general procedure A from 5-phenylpent-1-en-3-ol (**1a**, 49 mg) but using EtOH instead. Purification by column chromatography (SiO_2; petroleum ether / ethyl acetate, 90:10) afforded **6a** as a colorless oil (20 mg, 32%).

^{1}H NMR (400 MHz, $CDCl_3$): δ 7.31–7.27 (m, 2H), 7.22–7.19 (m, 3H), 4.29 (q, *J* = 6.9 Hz, 1H), 3.13–2.90 (m, 6H), 1.56 (d, *J* = 6.9 Hz, 3H), 0.90–0.83 (m, 3H) ppm. **^{13}C NMR (100 MHz, $CDCl_3$):** δ 212.7, 141.4, 128.58, 128.54, 126.2, 81.4, 65.6, 38.9, 29.5, 17.5, 15.5. ppm. **HRMS (ESI):** m/z calcd for [$C_{13}H_{18}O_2$+Na^+]: 229.1199 [M-Na]$^+$; found: 229.1193.

1-Phenyl-4-propoxypentan-3-one (7a)

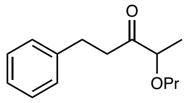

The title compound was prepared according to general procedure A from 5-phenylpent-1-en-3-ol (**1a**, 49 mg) but using PrOH instead. Purification by column chromatography (SiO_2; petroleum ether / ethyl acetate, 90:10) afforded **7a** as a colorless oil (13 mg, 20%).

^{1}H NMR (400 MHz, $CDCl_3$): δ 7.29–7.27 (m, 2H), 7.20–7.17 (m, 3H), 3.77 (q, *J* = 6.9 Hz, 1H), 3.36–3.26 (m, 2H), 2.92–2.84 (m, 4H), 1.23 (d, *J* = 6.9 Hz, 3H), 0.91 (t, *J* = 7.4 Hz, 3H) ppm. **^{13}C NMR (100 MHz, $CDCl_3$):** δ 212.8, 141.4, 128.6, 128.5, 126.2, 81.6, 72.0, 38.9, 29.5, 23.2, 17.4, 10.7 ppm. **HRMS (ESI):** m/z calcd for [$C_{14}H_{20}O_2$+Na^+]: 243.1356 [M-Na]$^+$; found: 243.1365.

5-Cyclohexyl-2-methylfuran-3(2*H*)-one (9a)

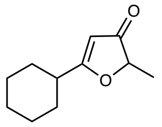

The title compound was prepared according to general procedure A from 1-cyclohexyl-3-hydroxypent-4-en-1-one (**8a**, 55 mg). Purification by column chromatography (SiO_2; petroleum ether / DCM, 80:20) afforded **9a** as a colorless oil (33 mg, 61%).

^{1}H NMR (400 MHz, $CDCl_3$): δ 5.37 (s, 1H), 4.46 (q, *J* = 7.4 Hz, 1H), 2.45–2.41 (m, 1H), 1.98–1.95 (m, 2H), 1.82–1.79 (m, 2H), 1.42 (d, *J* = 7.4 Hz, 3 H), 1.40–1.26 (m, 6H) ppm. **^{13}C NMR (100 MHz, $CDCl_3$):** δ 206.0, 197.6, 100.8, 82.3, 53.6, 39.8, 30.0, 29.9, 25.9, 25.7, 16.6 ppm. **HRMS (ESI):** m/z calcd for [$C_{11}H_{16}O_2$+Na]$^+$: 203.1043 [M-Na]$^+$; found: 203.1048.

5-Cyclohexyl-2,4-dimethylfuran-3(2*H*)-one (9b)

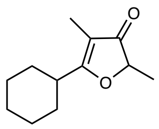

The title compound was prepared according to general procedure A from 1-cyclohexyl-3-hydroxy-2-methylpent-4-en-1-one (**8b**, 59 mg). Purification by column chromatography (SiO_2; petroleum ether / DCM, 80:20) afforded **9b** as a colorless oil (46 mg, 80%).

^{1}H NMR (400 MHz, CDCl$_3$): δ 4.37 (q, *J* = 7.1 Hz, 1H), 2.69–2.61 (m, 1H), 1.85–1.81 (m, 2H), 1.77–1.75 (m, 2H), 1.66 (s, 3H), 1.58–1.49 (m, 2H), 1.38 (d, *J* = 7.1 Hz, 3 H), 1.39–1.26 (m, 4H) ppm. **^{13}C NMR (100 MHz, CDCl$_3$):** δ 206.5, 191.1, 108.1, 80.6, 38.6, 29.2, 29.0, 25.92, 25.92, 25.8, 16.8, 5.6 ppm. **HRMS (ESI):** m/z calcd for $[C_{12}H_{18}O_2+Na]^+$: 217.1199 $[M\text{-}Na]^+$; found: 217.1202.

5-Cyclohexyl-2-ethyl-4-methylfuran-3(2*H*)-one (9c)

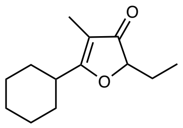

The title compound was prepared according to general procedure A from (*E*)-1-cyclohexyl-3-hydroxy-2-methylhex-4-en-1-one (**8c**, 63 mg). Purification by column chromatography (SiO_2; petroleum ether / DCM, 80:20) afforded **9c** as a colorless oil (47 mg, 75%).

^{1}H NMR (400 MHz, CDCl$_3$): δ 4.30–4.28 (m, 1H), 2.70–2.63 (m, 1H), 1.98–1.90 (m, 1H), 1.85–1.82 (m, 2H), 1.79–1.75 (m, 3H), 1.66 (s, 3H), 1.58–1.52 (m, 2H), 1.39–1.24 (m, 4H), 0.91 (t, *J* = 7.4 Hz, 3 H) ppm. **^{13}C NMR (100 MHz, CDCl$_3$):** δ 205.7, 191.6, 109.2, 85.0, 38.6, 29.3, 29.0, 25.9, 25.9, 25.8, 24.7, 8.5, 5.5 ppm. **HRMS (ESI):** m/z calcd for $[C_{13}H_{20}O_2+Na]^+$: 231.1356 $[M\text{-}Na]^+$; found: 231.1367.

5-Ethyl-2,4-dimethylfuran-3(2*H*)-one (9d)

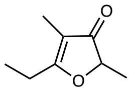

The title compound was prepared according to general procedure A from 5-hydroxy-4-methylhept-6-en-3-one (**8d**, 43 mg). Purification by column chromatography (SiO_2; petroleum ether / DCM, 80:20) afforded **9d** as a colorless oil (30 mg, 71%).

^{1}H NMR (400 MHz, CDCl$_3$): δ 4.38 (q, *J* = 7.1 Hz, 1H), 2.51 (q, *J* = 7.6 Hz, 2H), 1.65 (s, 3H), 1.39 (d, *J* = 7.1 Hz, 3H), 1.20 (t, *J* = 7.6 Hz, 3H) ppm. **^{13}C NMR (100 MHz, CDCl$_3$):** δ 206.2, 188.8, 109.0, 80.8, 22.4, 16.6, 10.5, 5.5 ppm. **HRMS (ESI):** m/z calcd for $[C_8H_{12}O_2+Na]^+$: 163.0730 $[M\text{-}Na]^+$; found: 163.0731.

4-Ethyl-2-methyl-5-propylfuran-3(2*H*)-one (9e)

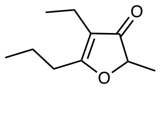

The title compound was prepared according to general procedure A from 5-ethyl-6-hydroxyoct-7-en-4-one (**8e**, 51 mg). Purification by column chromatography (SiO_2; petroleum ether / DCM, 80:20) afforded **9e** as a colorless oil (38 mg, 75%).

^{1}H NMR (400 MHz, $CDCl_3$): δ 4.37 (q, *J* = 7.1 Hz, 1H), 2.48 (t, J = 7.4 Hz, 2H), 2.13 (q, *J* = 7.5 Hz, 2H), 1.72–1.63 (m, 2H), 1.39 (d, *J* = 7.1 Hz, 3H), 1.03 (t, *J* = 7.5 Hz, 3H), 0.98 (t, *J* = 7.4 Hz, 3H) ppm. **^{13}C NMR (100 MHz, $CDCl_3$):** δ 206.0, 187.6, 115.9, 80.7, 30.7, 20.0, 16.6, 14.6, 13.92, 13.88 ppm. **HRMS (ESI):** m/z calcd for $[C_{10}H_{16}O_2+CH_3OH+Na]^+$: 223.1305; found: 223.1310.

5-Butyl-2-methyl-4-propylfuran-3(2*H*)-one (9f)

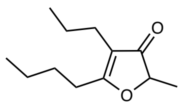

The title compound was prepared according to general procedure A from 3-hydroxy-4-propylnon-1-en-5-one (**8f**, 59 mg). Purification by column chromatography (SiO_2; petroleum ether / DCM, 80:20) afforded **9f** as a colorless oil (49 mg, 82%).

^{1}H NMR (400 MHz, $CDCl_3$): δ 4.36 (q, *J* = 7.1 Hz, 1H), 2.51–2.47 (m, 2H), 2.10–2.06 (m, 2H), 1.65–1.58 (m, 2H), 1.48–1.33 (m, 7H), 0.93 (t, *J* = 7.3 Hz, 3H), 0.88 (t, *J* = 7.4 Hz, 3H) ppm. **^{13}C NMR (100 MHz, $CDCl_3$):** δ 206.0, 188.1, 114.1, 80.6, 28.7, 28.6, 23.3, 22.6, 22.3, 16.7, 13.90, 13.90 ppm. **HRMS (ESI):** m/z calcd for $[C_{12}H_{20}O_2+Na]^+$: 219.1356 $[M-Na]^+$; found: 219.1351.

2-Methyl-4,5,6,7-tetrahydrobenzofuran-3(2*H*)-one (9g)

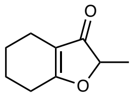

The title compound was prepared according to general procedure A from 2-(1-hydroxyallyl)octahydronaphthalen-1(2*H*)-one (**8g**, 46 mg). Purification by column chromatography (SiO_2; petroleum ether / DCM, 80:20) afforded **9g** as a colorless oil (21 mg, 46%).

^{1}H NMR (400 MHz, $CDCl_3$): δ 4.46–4.41 (m, 1H), 2.44–2.41 (m, 2H), 2.20–2.16 (m, 2H), 1.85–1.81 (m, 2H), 1.69–1.64 (m, 2H), 1.43 (d, *J* = 7.4 Hz, 3H) ppm. **^{13}C NMR (100 MHz, $CDCl_3$):** δ 204.0, 188.1, 112.3, 81.9, 26.0, 22.0, 21.9, 18.3, 16.5 ppm. **HRMS (ESI):** m/z calcd for $[C_9H_{12}O_2+Na]^+$: 175.0730 $[M-Na]^+$; found: 175.0737.

(4*R*,7*S*)-2-Ethyl-7-isopropyl-4-methyl-4,5,6,7-tetrahydrobenzofuran-3(2*H*)-one (9h)

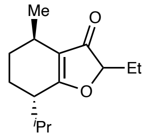

The title compound was prepared according to general procedure A from (3R,6S)-2-((E)-1-hydroxybut-2-en-1-yl)-6-isopropyl-3-methylcyclohexan-1-one (**8h**, 67 mg). Purification by column chromatography (SiO_2; petroleum ether / ethyl acetate, 90:10) afforded **9h** as a colorless oil (49 mg, 74% as a mixture of two diastereoisomers *ca*. 1:1).

^{1}H NMR (400 MHz, $CDCl_3$ as a mixture of two diastereoisomers *ca*. 1:1): δ 4.29–4.24 (m, 1H (both diast.)), 2.52–2.44 (m, 2H (both diast.)), 2.31–2.22 (m, 1H (both diast.)), 2.02–1.80 (m, 4H (both diast.)), 1.73–1.63 (m, 1H (both diast.)), 1.54–1.46 (m, 1H (both diast.)), 1.21–1.15 (m, 3H (both diast.)), 1.04–0.94 (m, 6H (both diast.)), 0.89–0.84 (m, 3H (both diast.)) ppm. **^{13}C NMR (100 MHz, $CDCl_3$ as a mixture of two diastereoisomers *ca*. 1:1):** δ 204.0, 203.8, 190.6, 190.2, 117.8, 117.7, 86.3, 86.0, 42.8, 42.7, 31.34, 31.32, 28.5, 28.4, 26.79, 26.76, 24.8, 24.4, 22.1, 22.0, 20.17, 20.16, 18.85, 18.77, 18.5, 18.4, 9.6, 8.8. ppm. **HRMS (ESI):** m/z calcd for $[C_{14}H_{22}O_2+Na]^+$: 245.1512 $[M-Na]^+$; found: 245.1520.

5-(Dimethylamino)-2-methylfuran-3(2*H*)-one (9i)

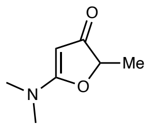

The title compound was prepared according to general procedure A from 3-hydroxy-*N,N*-dimethylpent-4-enamide (**8i**, 43 mg). Purification by column chromatography (SiO_2; DCM, and then DCM / MeOH, 95/5) afforded **9i** as a colorless oil (38 mg, 91%).

^{1}H NMR (400 MHz, $CDCl_3$): δ 4.59 (q, J = 7.0 Hz, 1H), 4.54 (s, 1H), 2.98 (bs, 6H), 1.43 (d, J = 7.0 Hz, 3H) ppm. **^{13}C NMR (100 MHz, $CDCl_3$):** δ 197.7, 178.1, 83.4, 78.4, 38.7, 35.9, 17.3 ppm. **HRMS (ESI):** m/z calcd for $[C_7H_{11}NO_2+Na]^+$: 164.0682 $[M-Na]^+$; found: 164.0680.

5-(Dimethylamino)-2,4-dimethylfuran-3(2*H*)-one (9j)

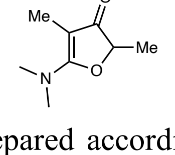

The title compound was prepared according to general procedure A from 3-hydroxy-*N,N*,2-trimethylpent-4-enamide (**8j**, 47 mg). Purification by column

chromatography (SiO_2; DCM, and then DCM / MeOH, 95/5) afforded **9j** as a colorless oil (40 mg, 87%).

^{1}H NMR (400 MHz, $CDCl_3$): δ 4.47 (q, *J* = 6.9 Hz, 1H), 3.12 (s, 6H), 1.86 (s, 3H), 1.39 (d, *J* = 6.9 Hz, 3H) ppm. **^{13}C NMR (100 MHz, $CDCl_3$):** 198.0, 175.5, 86.1, 80.9, 38.1, 17.5, 7.8. ppm. **HRMS (ESI):** m/z calcd for $[C_8H_{13}NO_2+Na]^+$: 178.0838 $[M-Na]^+$; found: 178.0843.

5-(Benzyl(methyl)amino)-2-methylfuran-3(2*H*)-one (9k)

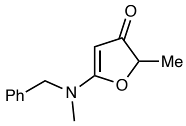

The title compound was prepared according to general procedure A from N-benzyl-3-hydroxy-N-methylpent-4-enamide (**8k**, 66 mg). Purification by column chromatography (SiO_2; DCM, and then DCM / MeOH, 95/5) afforded **9k** as a colorless oil (48 mg, 74%).

^{1}H NMR (400 MHz, $CDCl_3$): δ 7.38–7.30 (m, 3H), 7.22–7.20 (m, 2H), 4.66 (q, *J* = 6.9 Hz, 1H), 4.60–4.44 (m, 3H), 2.97–2.86 (bs, 3H), 1.39 (d, *J* = 6.9 Hz, 3H) ppm. **^{13}C NMR (100 MHz, $CDCl_3$):** 198.0, 177.9, 135.7, 129.1, 128.2, 127.6, 83.5, 78.7, 52.0, 36.4, 17.3 ppm. **HRMS (ESI):** m/z calcd for $[C_{13}H_{15}NO_2+Na]^+$: 240.0995 $[M-Na]^+$; found: 240.1002.

Mechanistic studies

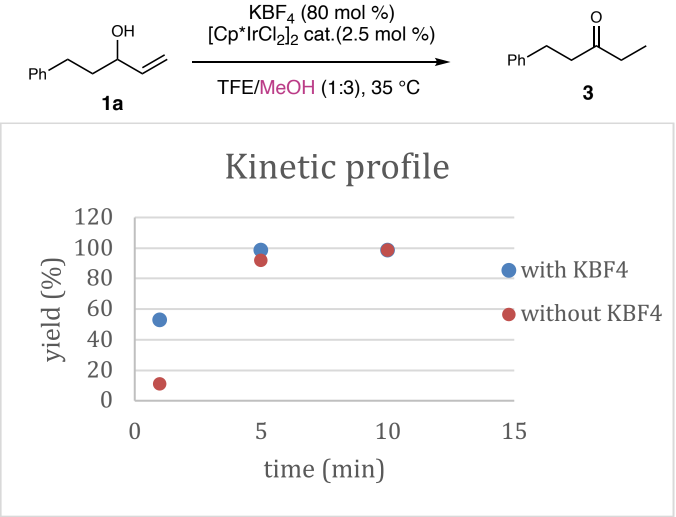

Supplementary Figure 1. Kinetic profile of isomerization of **1a** with (blue dots) and without (red dots) KBF_4.

(*Z*)-trimethyl((1-phenylprop-1-en-1-yl)oxy)silane **10** (0.1 mmol, 1 equiv.) in presence or in absence of KBF_4 (10 mg, 0.08 mmol, 80 mol%) was dissolved in a mixture MeOH / TFE (3:1) (5 mL). Then, 1-fluoro-3,3-dimethyl-1,3-dihydro-1-λ-3-benzo[*d*][1,2]iodaoxole (34 mg, 0.12 mmol, 1.2 equiv.) was added and the mixture stirred at 35 ºC during the depicted time. The mixture was then cooled to 0 °C and treated with $NaBH_4$ (20 mg, 0.5 mmol) to stop the reaction. After 10 minutes, H_2O was added to dilute the reaction and the mixture was extracted with Et_2O (3 x 1 mL). The combined organic phases were dried with $MgSO_4$ and the solvent was evaporated under vacuum. Yields of **11** were determined by ^{1}H NMR spectroscopy using an internal standard (1,2,4,5-tetrachloro-3-nitrobenzene) obtaining a diastereomeric ratio of 6:4 in all cases.

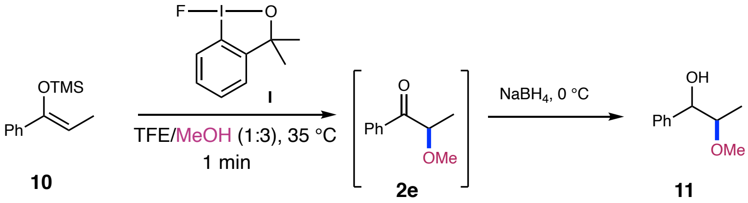

	Yield of **11** (%)	
time (minutes)	In absence of KBF_4	In presence of KBF_4
1	59	57
20	99	99

Supplementary Figure 2. Kinetic profile of **10** with and without KBF_4.

(Z)-trimethyl((1-phenylprop-1-en-1-yl)oxy)silane **10** (0.1 mmol, 1 equiv.) in presence or in absence of $[Cp^*IrCl_2]_2$ (2 mg, 0.0025 mmol, 2.5 mol%) was dissolved in a mixture MeOH / TFE (3:1) (5 mL). Then, 1-fluoro-3,3-dimethyl-1,3-dihydro-1-λ-3-benzo[*d*][1,2]iodaoxole (34 mg, 0.12 mmol, 1.2 equiv.) was added and the mixture stirred at 35 ºC during the depicted time. The mixture was then cooled to 0 °C and treated with $NaBH_4$ (20 mg, 0.5 mmol) to stop the reaction. After 10 minutes, H_2O was added to dilute the reaction and the mixture was extracted with Et_2O (3 x 1 mL). The combined organic phases were dried with $MgSO_4$ and the solvent was evaporated under vacuum. Yields of **11** were determined by ^{1}H NMR spectroscopy using an internal standard (1,2,4,5-tetrachloro-3-nitrobenzene) obtaining a diastereomeric ratio of 6:4 in all cases.

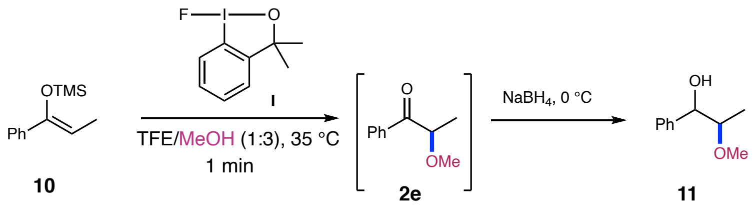

	Yield of **11** (%)	
time (minutes)	In absence of $[Cp^*IrCl_2]_2$	In presence of $[Cp^*IrCl_2]_2$
1	59	30
20	99	85

Supplementary Figure 3. Kinetic profile of **10** with and without $[Cp^*IrCl_2]_2$.

$[Cp^*Ir(H_2O)_3]SO_4$ (2.5 mg, 0.005 mmol, 0.025 equiv.), *N*-chlorosuccinimide (1.4 mg, 0.010 mmol, 0.05 equiv.) in presence or in absence of KBF_4 (20 mg, 0.16 mmol, 80 mol%) were dissolved in in a mixture MeOH / TFE (3:1) (8 mL). The mixture was stirred for 15 minutes at rt. Then, a solution of allylic alcohol **1a** (32 mg, 0.2 mmol, 1 equiv.) in a mixture MeOH / TFE (3:1) (2 mL) and 1-fluoro-3,3-dimethyl-1,3-dihydro-1-λ-3-benzo[*d*][1,2]iodaoxole (68 mg, 0.24 mmol, 1.2 equiv.) were added and the mixture stirred at 35 ºC during 2 h. After that, H_2O was added to dilute the reaction and the mixture was extracted with Et_2O (3 x 1 mL). The combined organic phases were dried with $MgSO_4$ and the solvent was evaporated under vacuum. Yields of **2a** were determined by ^{1}H NMR spectroscopy using an internal standard (1,2,4,5-tetrachloro-3-nitrobenzene).

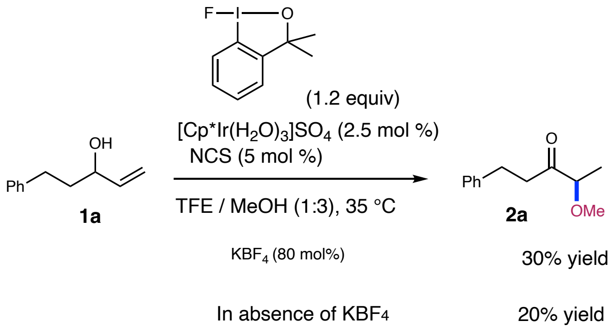

Supplementary Figure 4. Catalyst studies

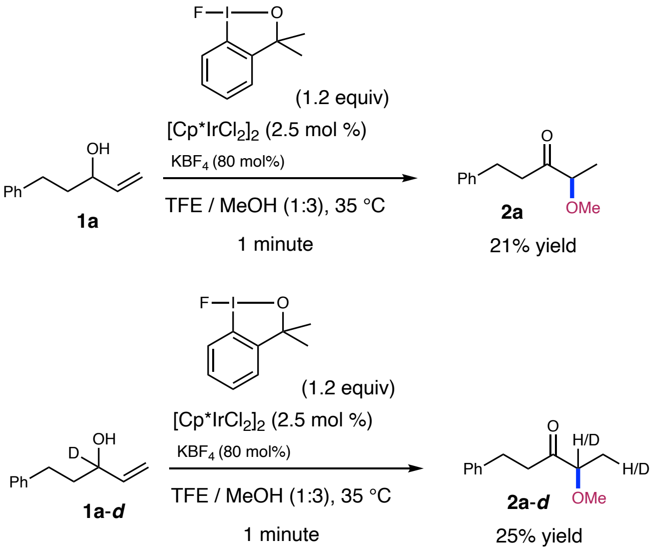

Supplementary Figure 5. Deuterium labelling experiments.

Allylic alcohol **1a** (0.1 mmol, 1 equiv.) was dissolved in a mixture MeOH / TFE (3:1) (5 mL). The corresponding radical scavenger (0.1 mmol or 0.3 mmol), KBF_4 (10 mg, 0.08 mmol, 80 mol%), 1-fluoro-3,3-dimethyl-1,3-dihydro-1-λ-3-benzo[*d*][1,2]iodaoxole (34 mg, 0.12 mmol, 1.2 equiv.), and $[Cp^*IrCl_2]_2$ (2 mg, 0.0025 mmol, 0.025 equiv.) were added and the mixture stirred at 35 ºC during 2 h. After that, H_2O was added to dilute the reaction and the mixture was extracted with Et_2O (3 x 1 mL). The combined organic phases were dried with $MgSO_4$ and the solvent was evaporated under vacuum. Yields of **2a** were determined by ^1H NMR spectroscopy using an internal standard (1,2,4,5-tetrachloro-3-nitrobenzene).

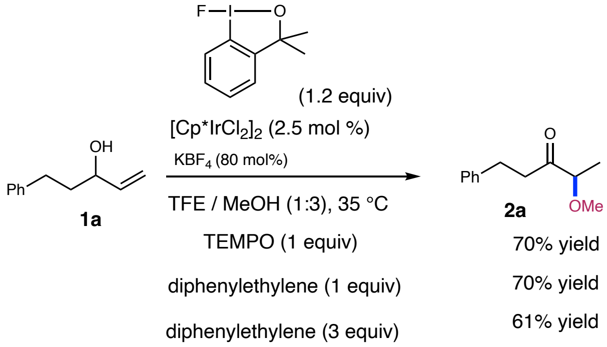

Supplementary Figure 6. Radical-trapping experiments.

Computational Methods

All structures were initially optimized using density functional theory (DFT) by using the M06[13] functional as implemented in Gaussian 16.[14] Optimizations were carried out by using the SDD[15] basis set for Iodine and 6-31G** basis set for the rest of the atoms. The critical stationary points were characterized by frequency calculations in order to verify that they have the right number of imaginary frequencies, and the intrinsic reaction coordinates (IRC)[16] were followed to verify the energy profiles connecting the key transition structures to the correct associated local minima.
The energies showed in the manuscript have been refined by single-point calculations on the previously optimized structures, by applying the M06 functional and def2tzvpp basis set in a solvent model (IEFPCM, solvent = methanol).[17-19]

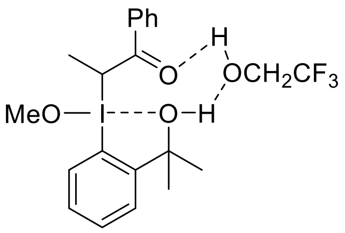

Enolium C

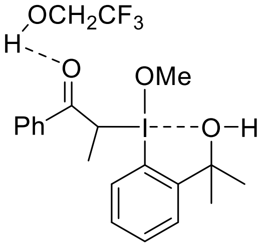

Enolium C"

Supplementary Figure 7. Two possible structures for enolonium C. Enolonium C (0.0 kcal mol^{-1}) and Enolonium C'' (13.9 kcal mol^{-1}).

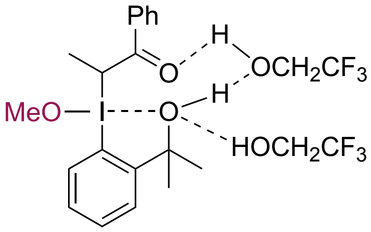

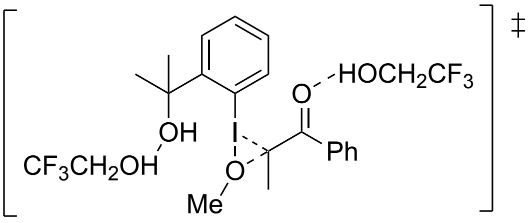

C_2TFE **TS_1_2TFE**

Supplementary Figure 8. Energies of intermediate C and TS1 with 2 molecules of TFE.

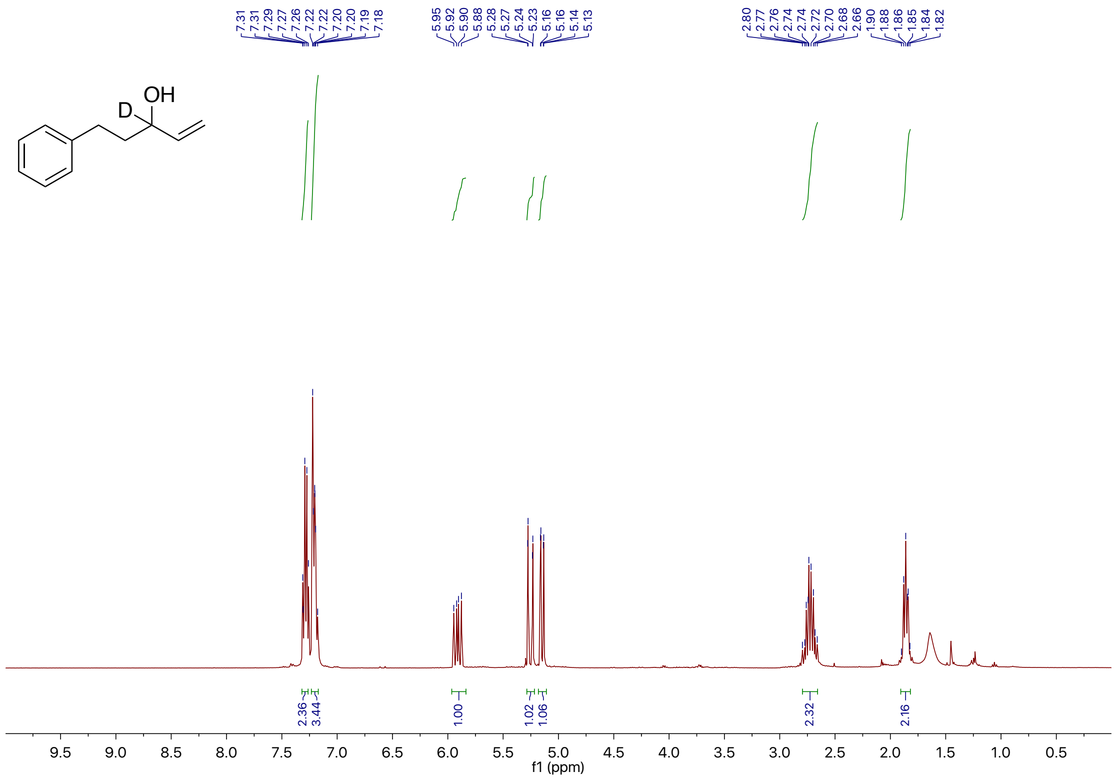

Supplementary Figure 9. ^{1}H NMR spectra ($CDCl_3$, 400 MHz) of **1a-*d***

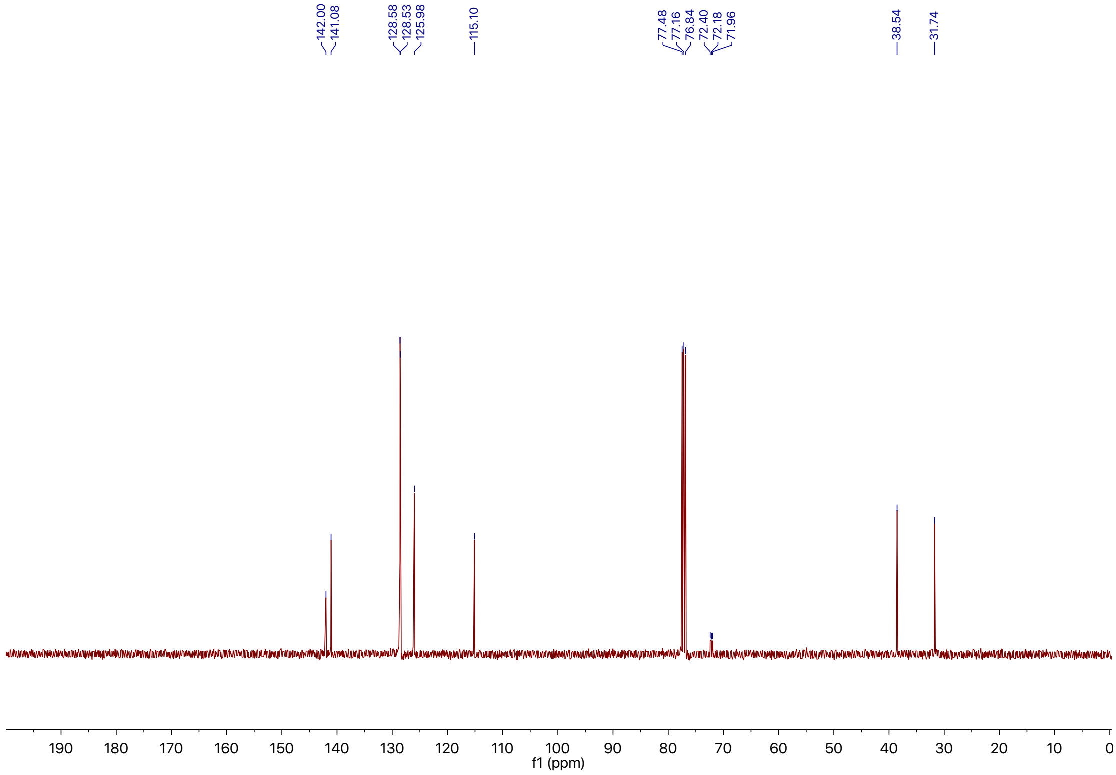

Supplementary Figure 10. ^{13}C NMR spectra ($CDCl_3$, 100 MHz) of **1a-*d***

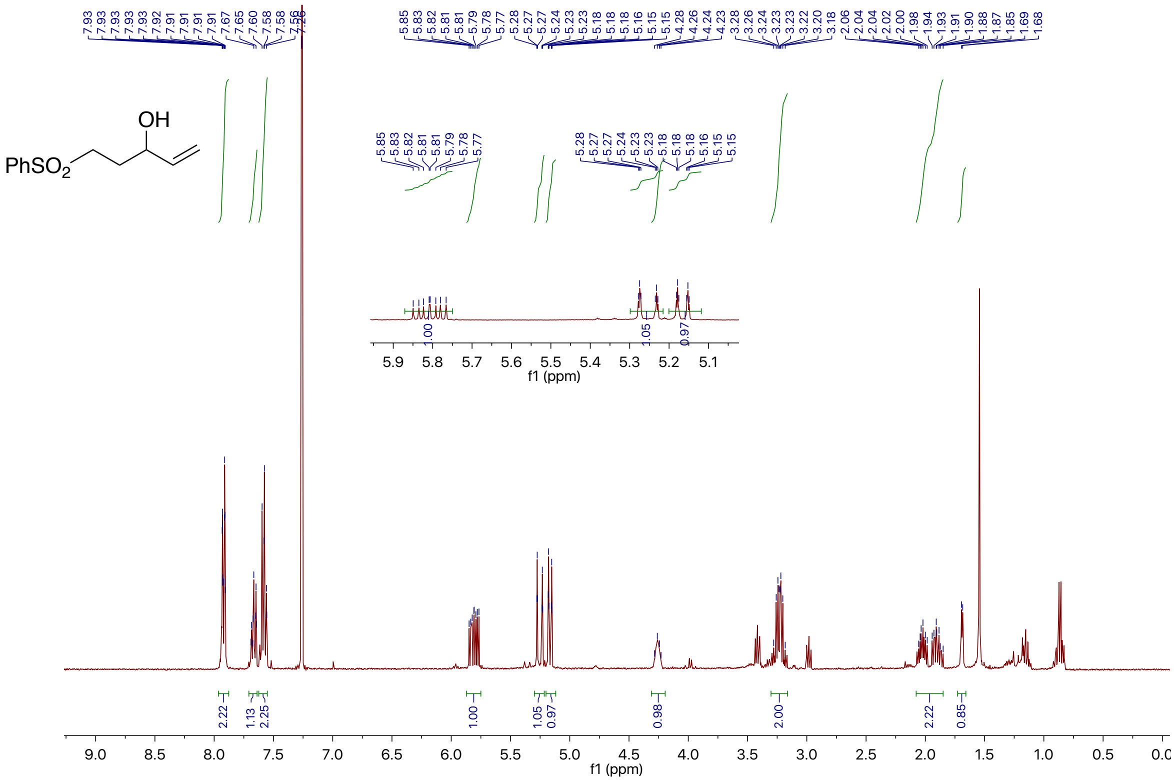

Supplementary Figure 11. ^{1}H NMR spectra ($CDCl_3$, 400 MHz) of **1j**

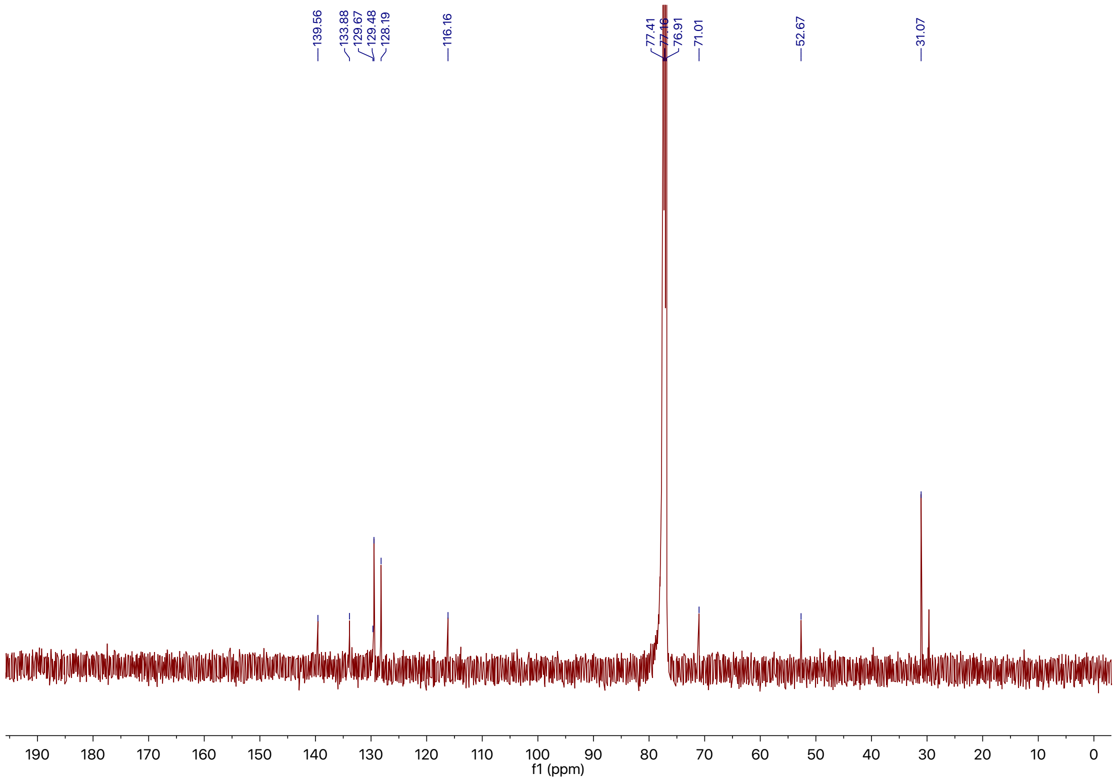

Supplementary Figure 12. ^{13}C NMR spectra ($CDCl_3$, 100 MHz) of **1j**

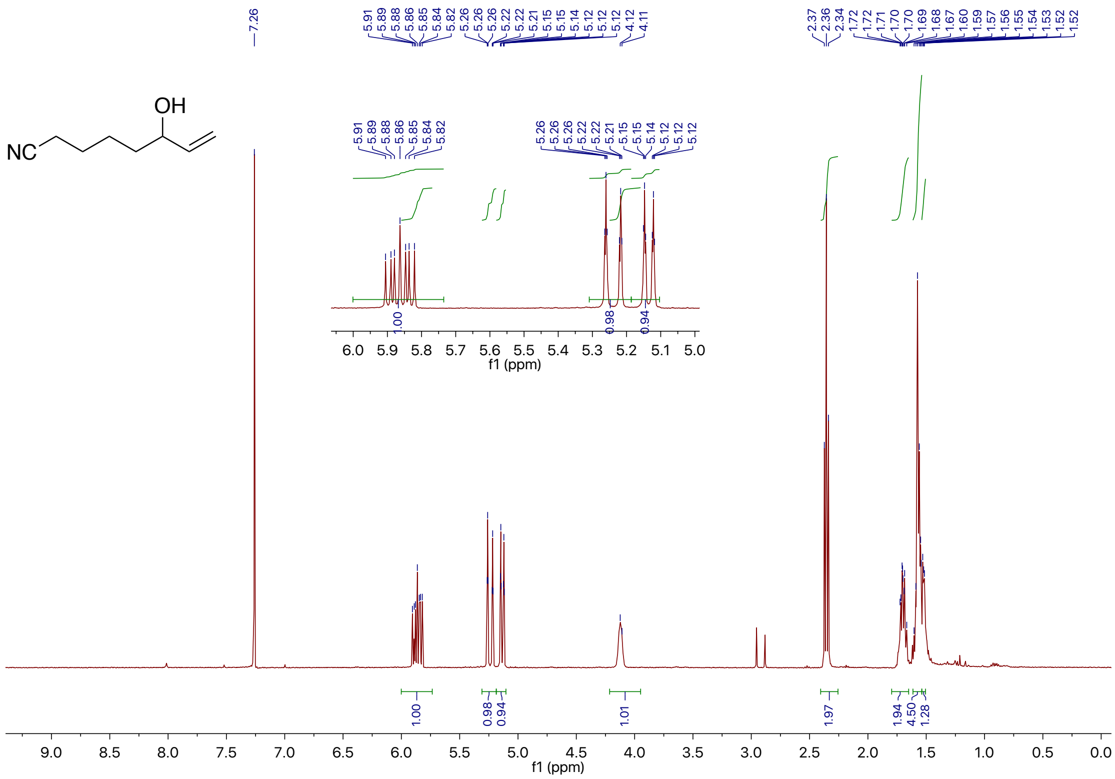

Supplementary Figure 13. ^{1}H NMR spectra ($CDCl_3$, 400 MHz) of **1l**

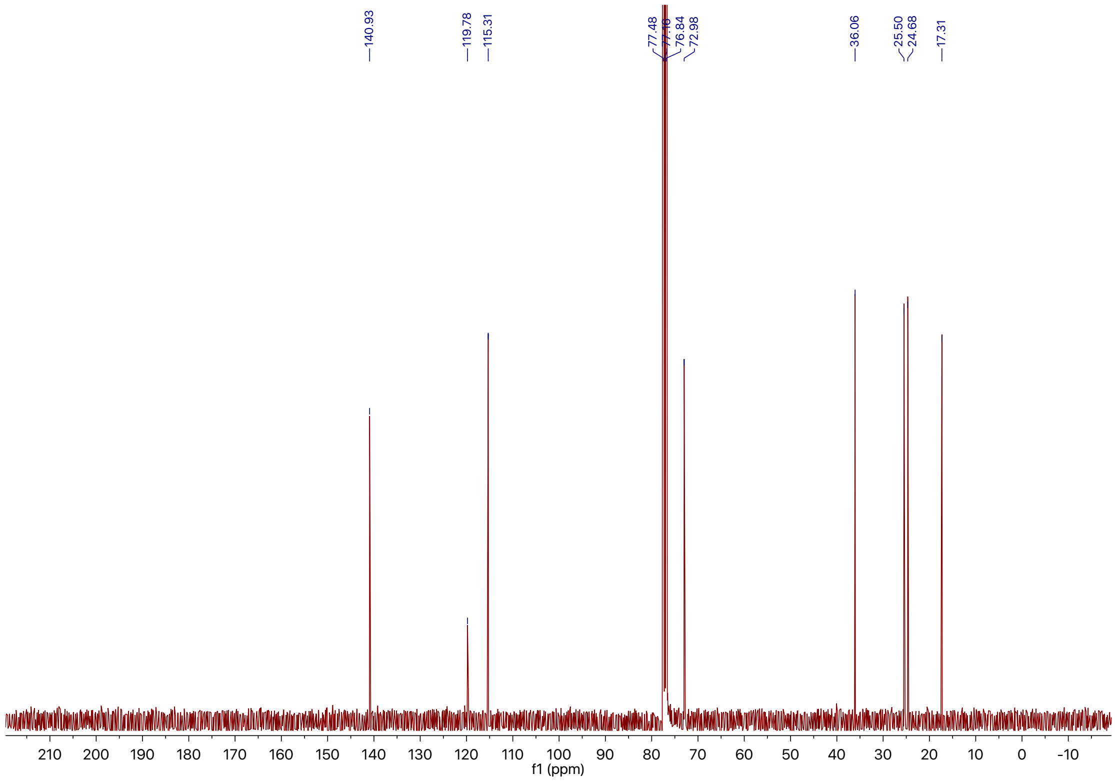

Supplementary Figure 14. ^{13}C NMR spectra ($CDCl_3$, 100 MHz) of **1l**

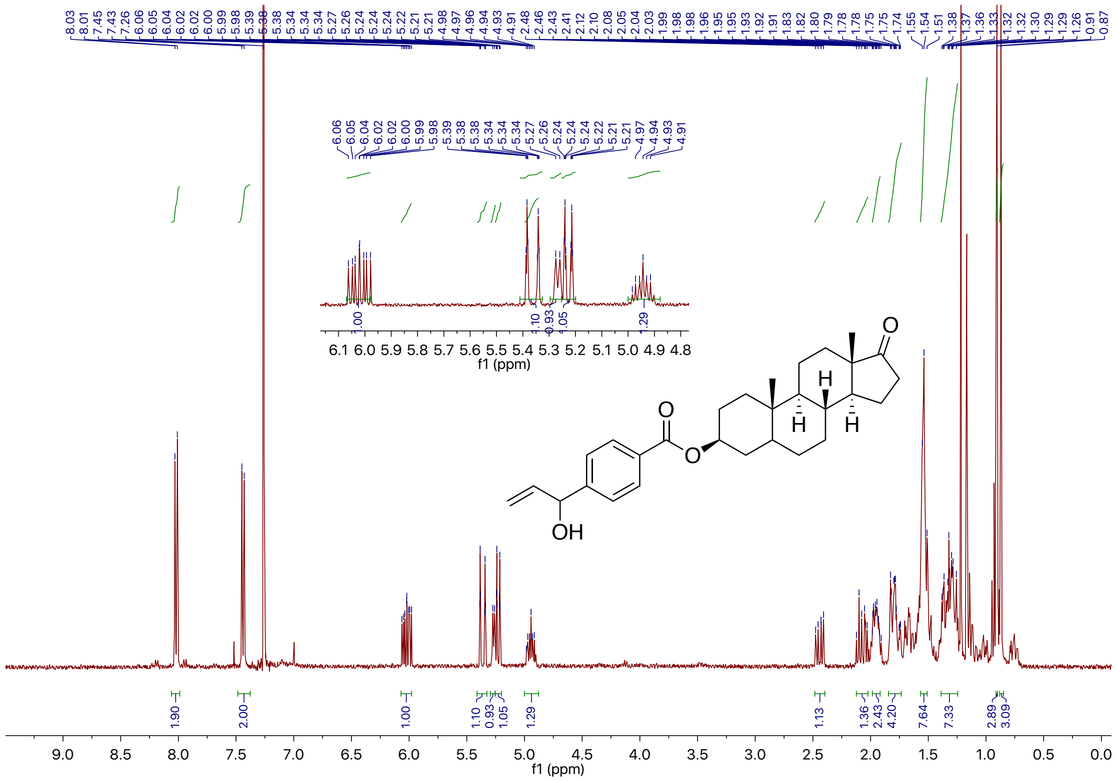

Supplementary Figure 15. ^{1}H NMR spectra ($CDCl_3$, 400 MHz) of **1x**

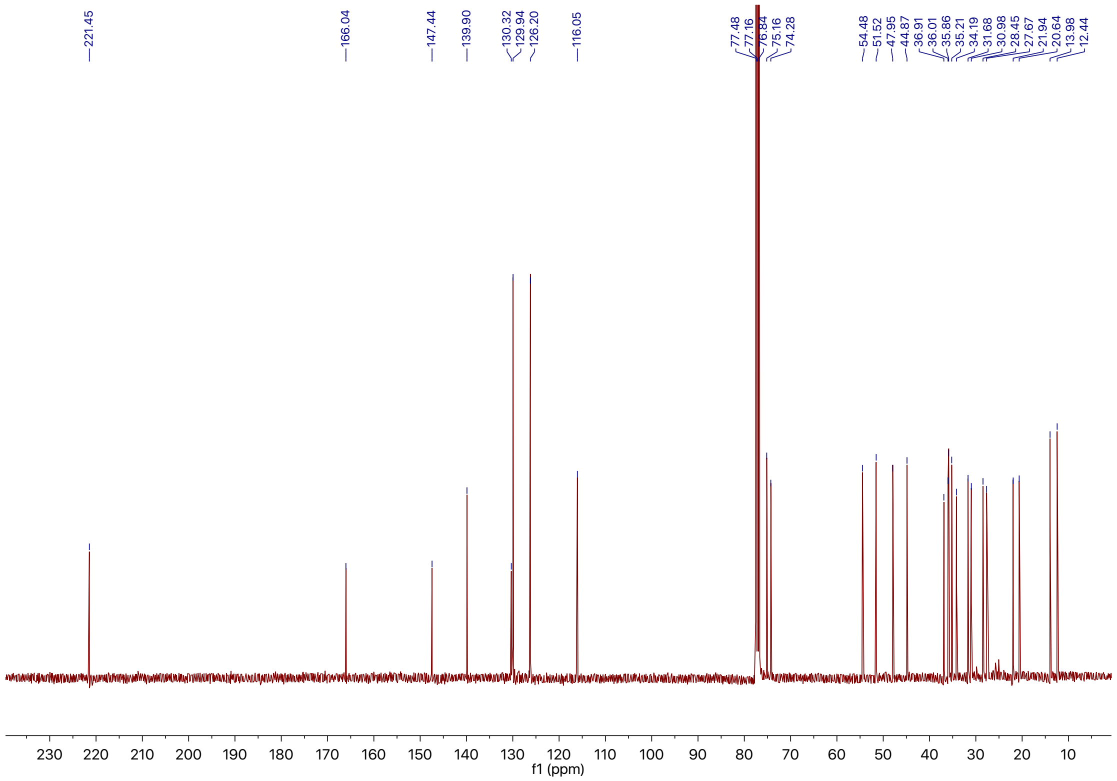

Supplementary Figure 16. ^{13}C NMR spectra ($CDCl_3$, 100 MHz) of **1x**

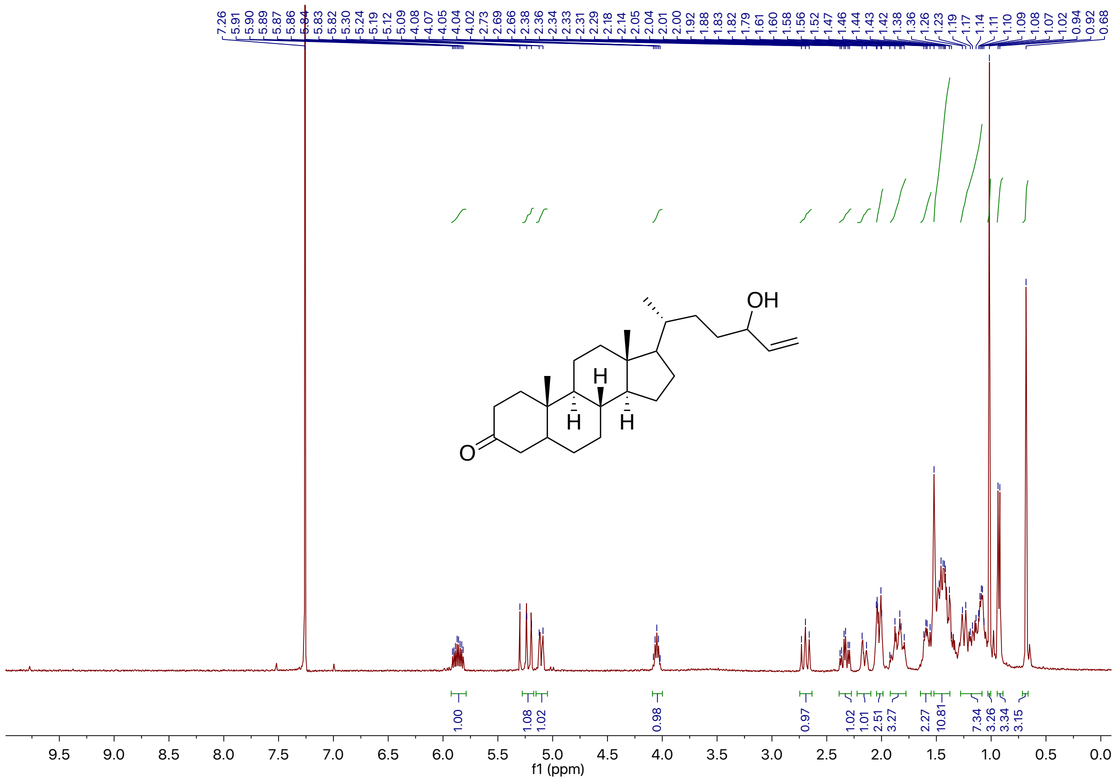

Supplementary Figure 17. ^{1}H NMR spectra ($CDCl_3$, 400 MHz) of **1y**

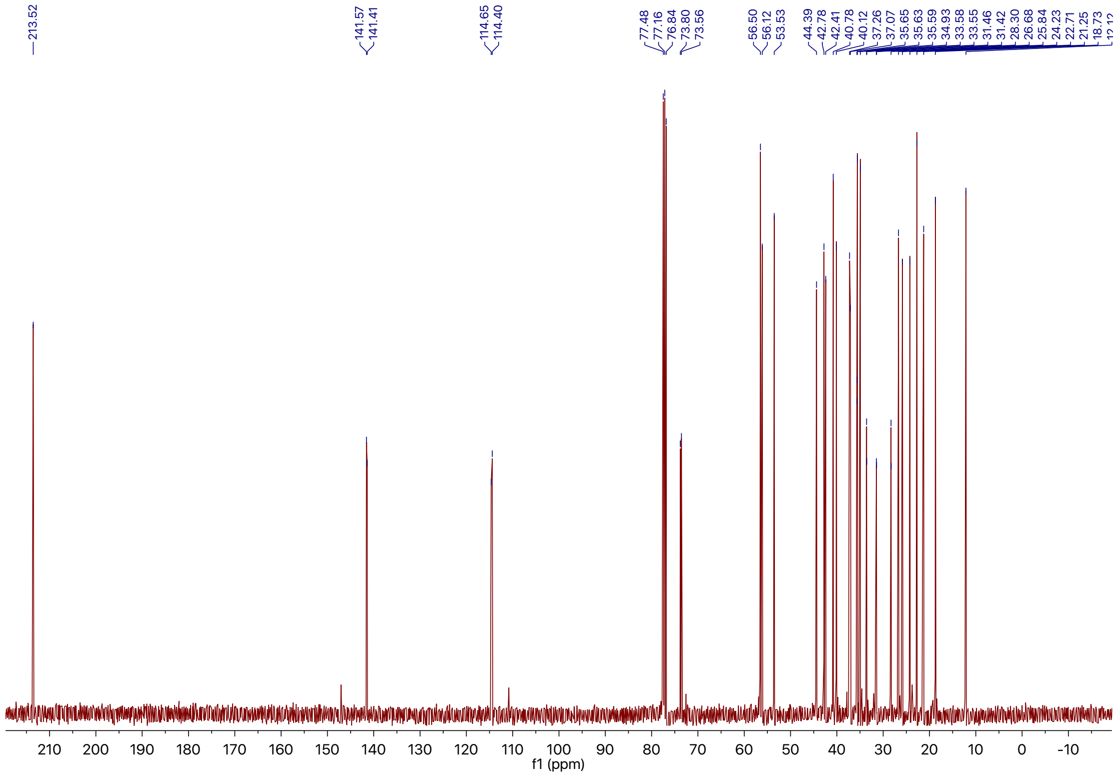

Supplementary Figure 18. ^{13}C NMR spectra ($CDCl_3$, 100 MHz) of **1y**

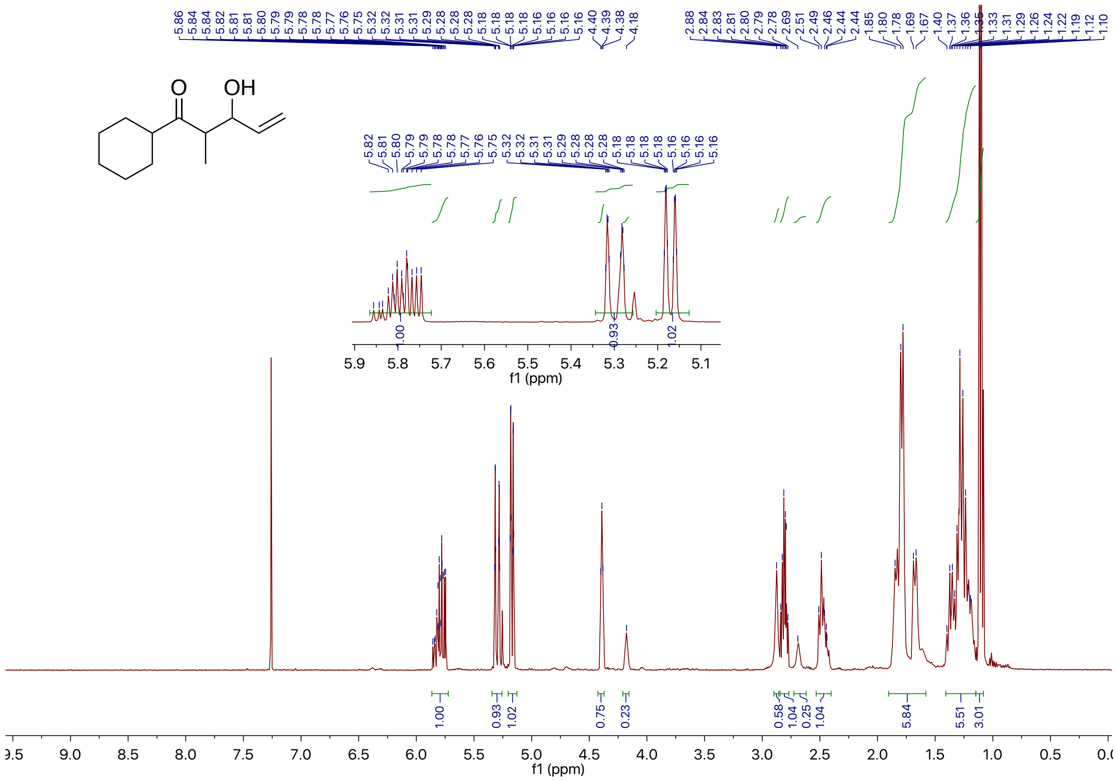

Supplementary Figure 19. ^{1}H NMR spectra ($CDCl_3$, 400 MHz) of **8b**

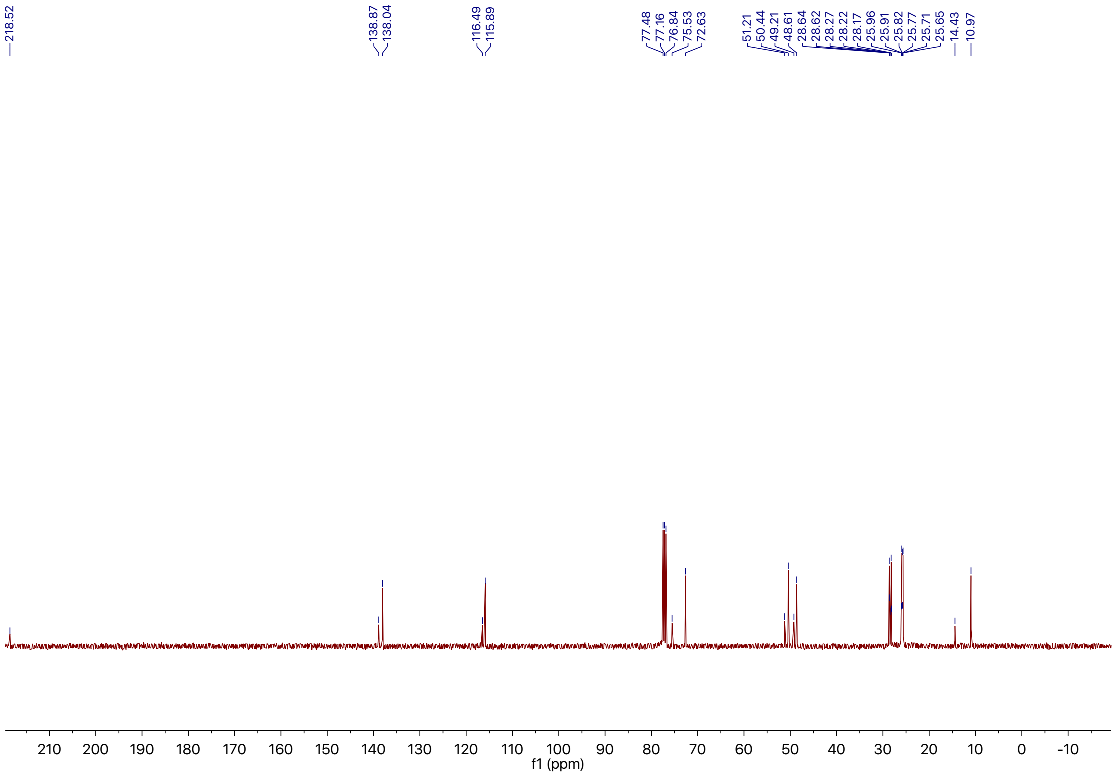

Supplementary Figure 20. ^{13}C NMR spectra ($CDCl_3$, 100 MHz) of **8b**

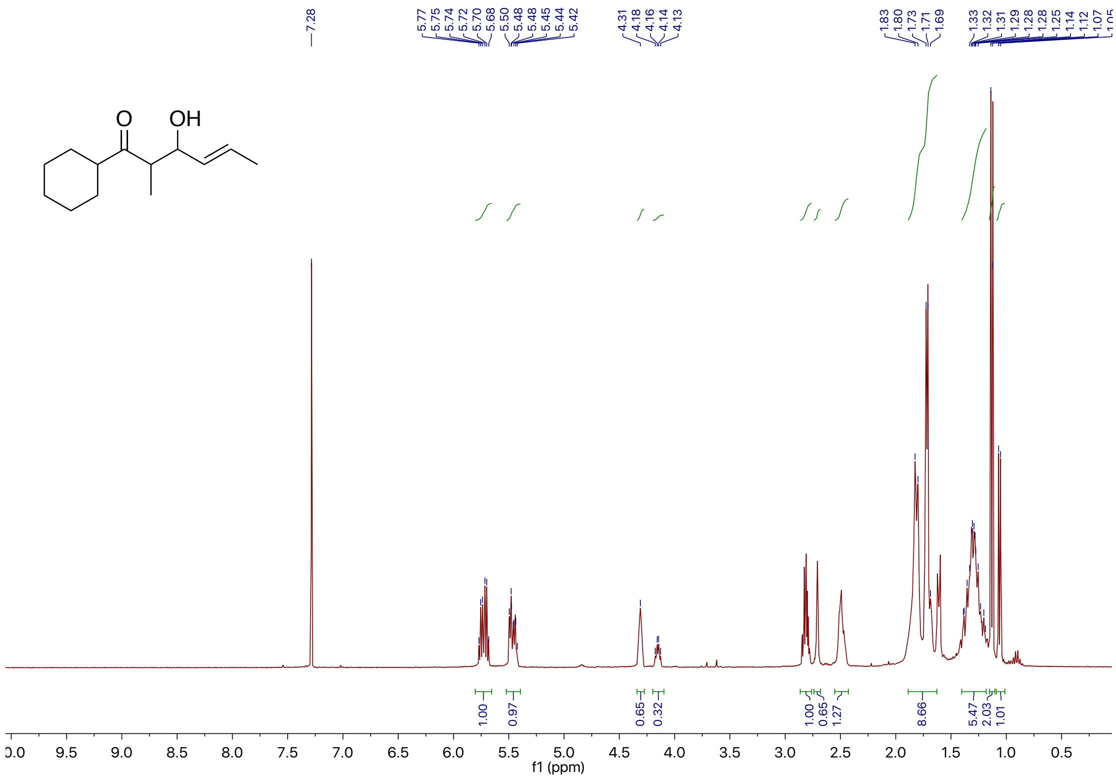

Supplementary Figure 21. ^{1}H NMR spectra ($CDCl_3$, 400 MHz) of **8c**

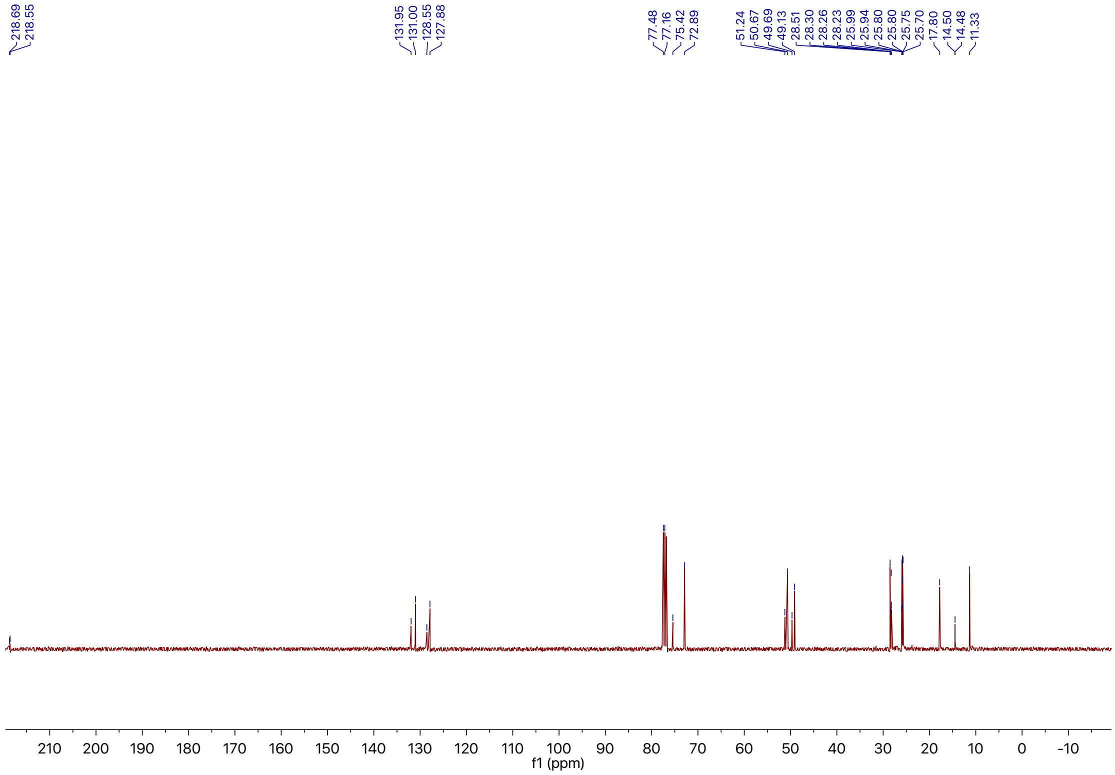

Supplementary Figure 22. ^{13}C NMR spectra ($CDCl_3$, 100 MHz) of **8c**

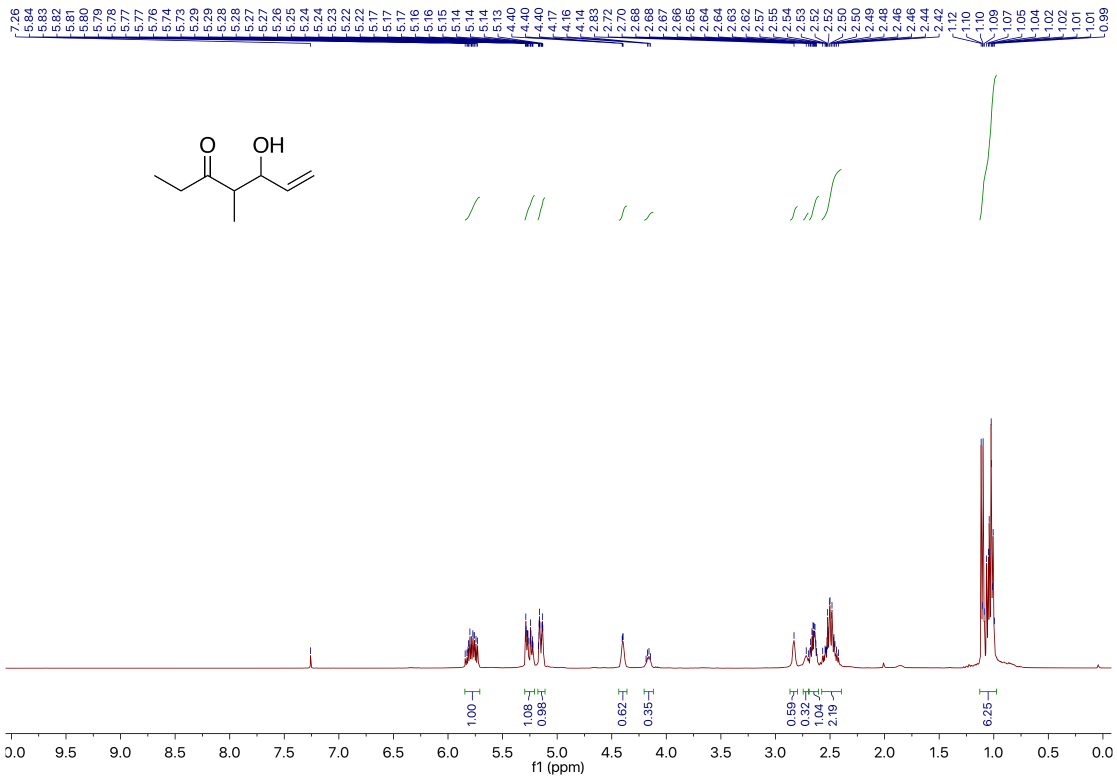

Supplementary Figure 23. ^{1}H NMR spectra ($CDCl_3$, 400 MHz) of **8d**

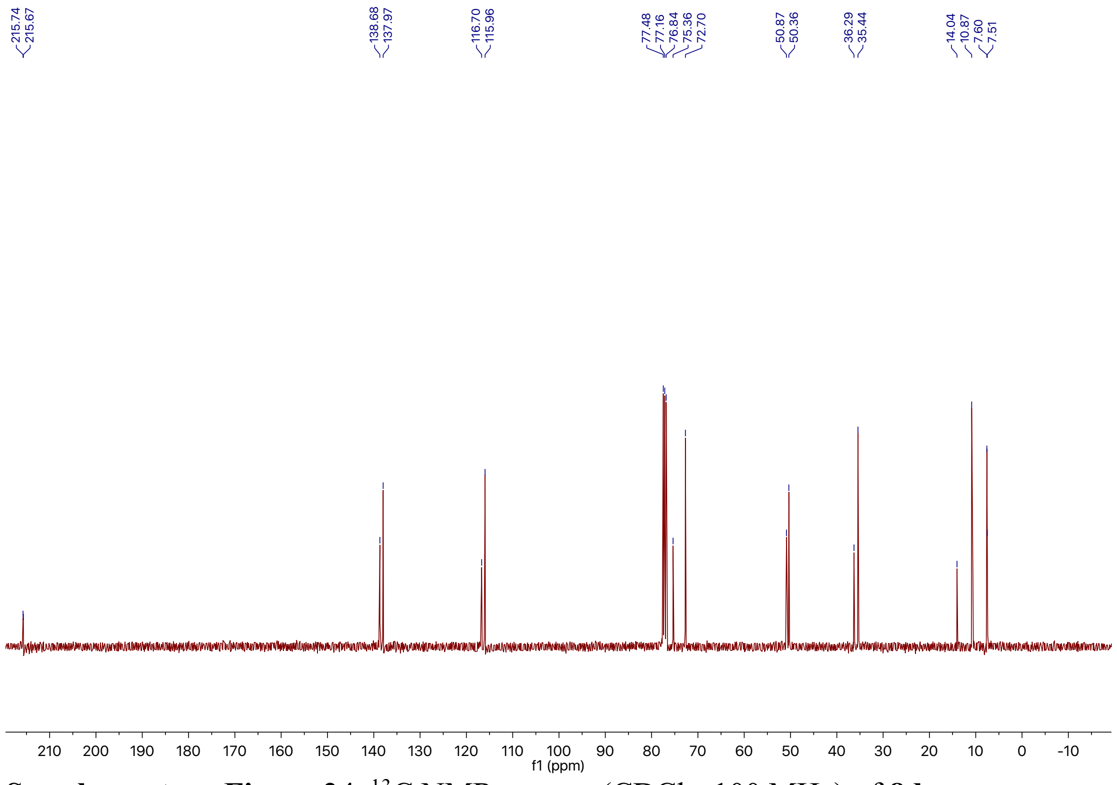

Supplementary Figure 24. ^{13}C NMR spectra ($CDCl_3$, 100 MHz) of **8d**

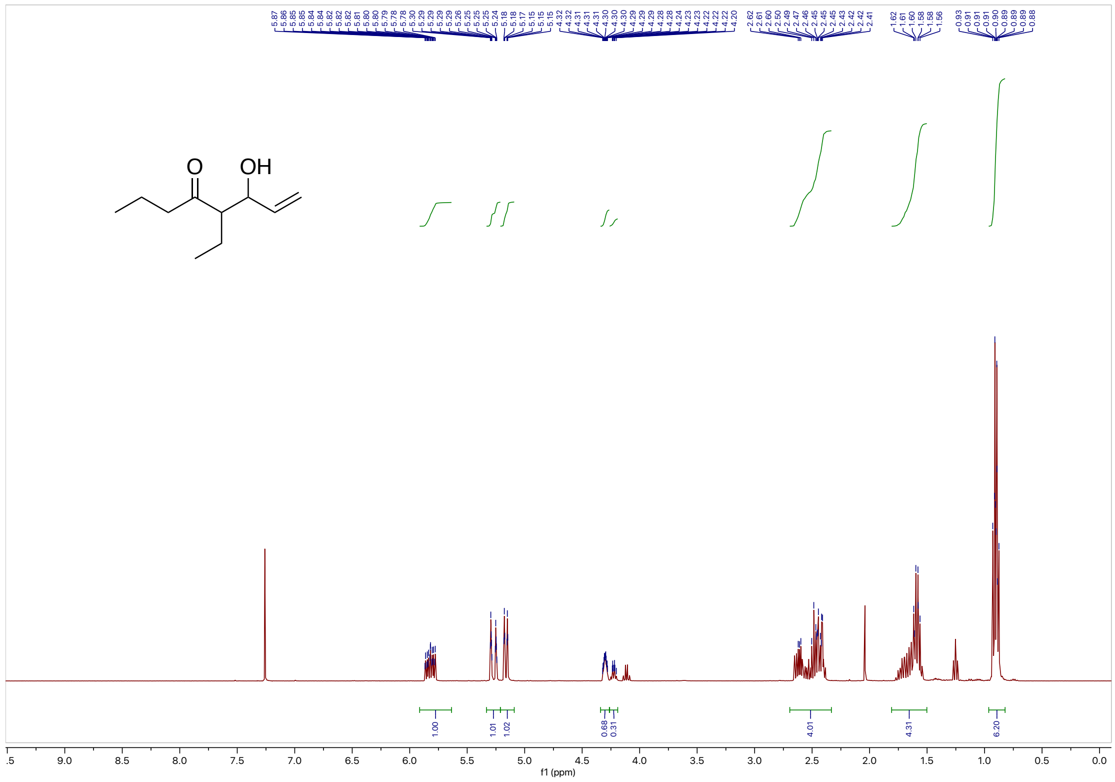

Supplementary Figure 25. ^{1}H NMR spectra ($CDCl_3$, 400 MHz) of **8e**

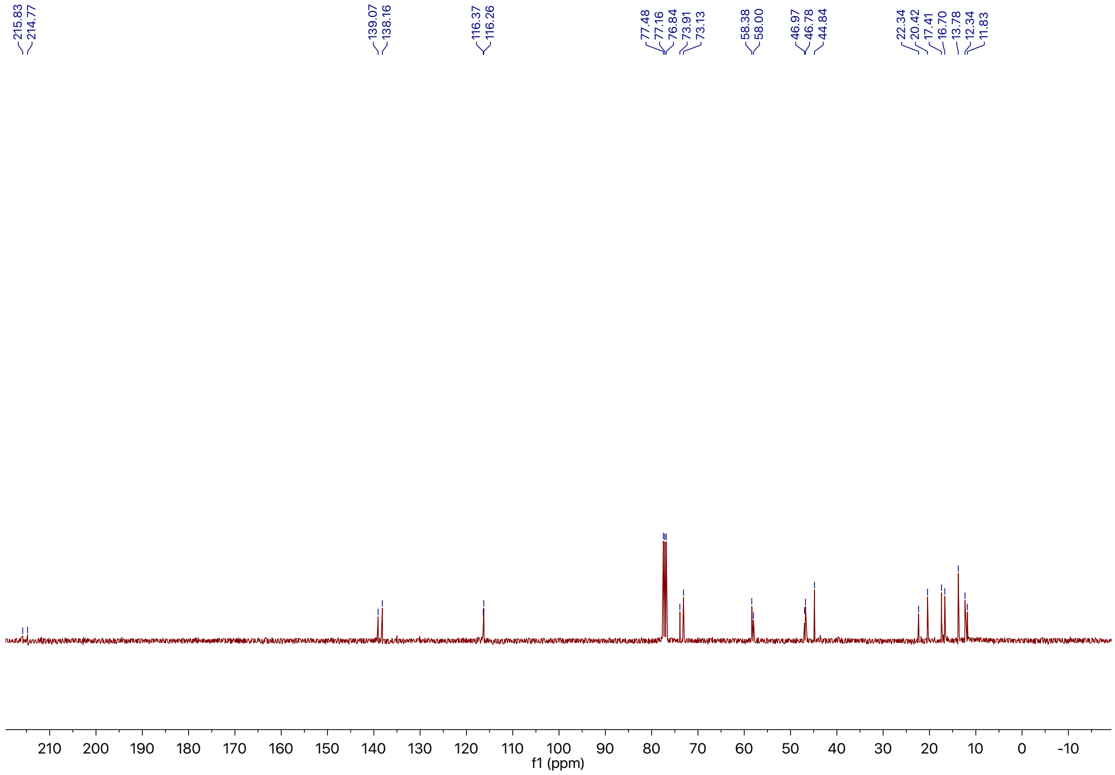

Supplementary Figure 26. ^{13}C NMR spectra ($CDCl_3$, 100 MHz) of **8e**

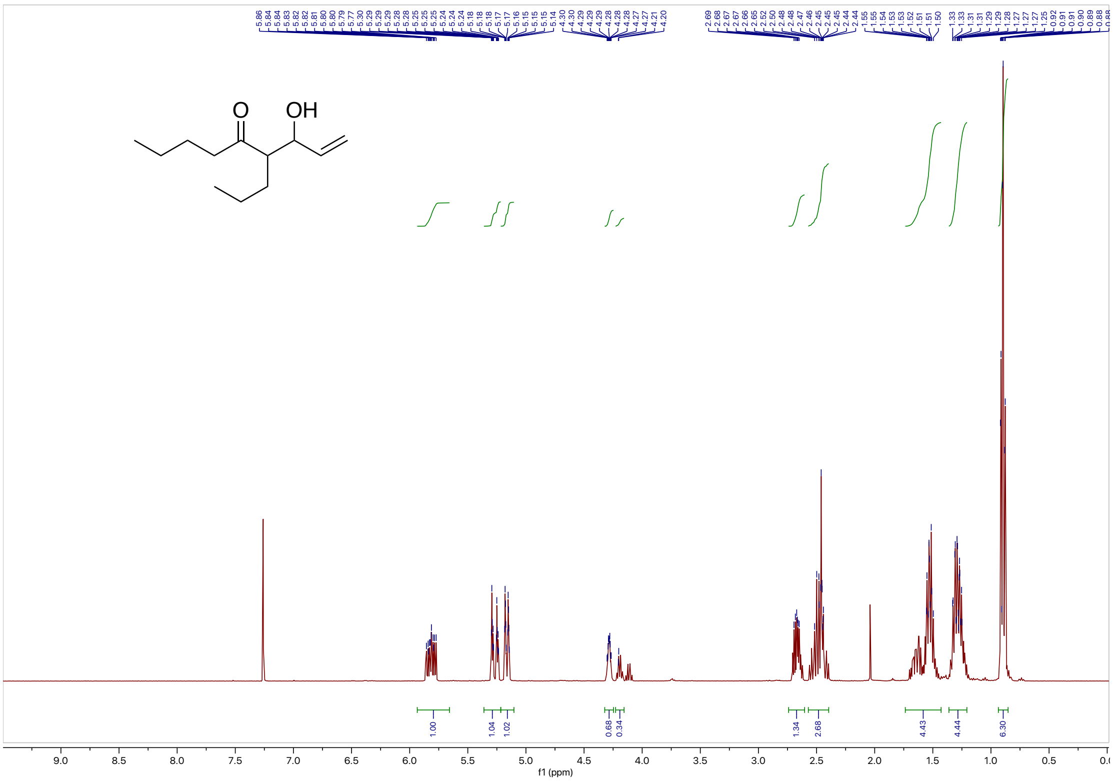

Supplementary Figure 27. ^{1}H NMR spectra ($CDCl_3$, 400 MHz) of **8f**

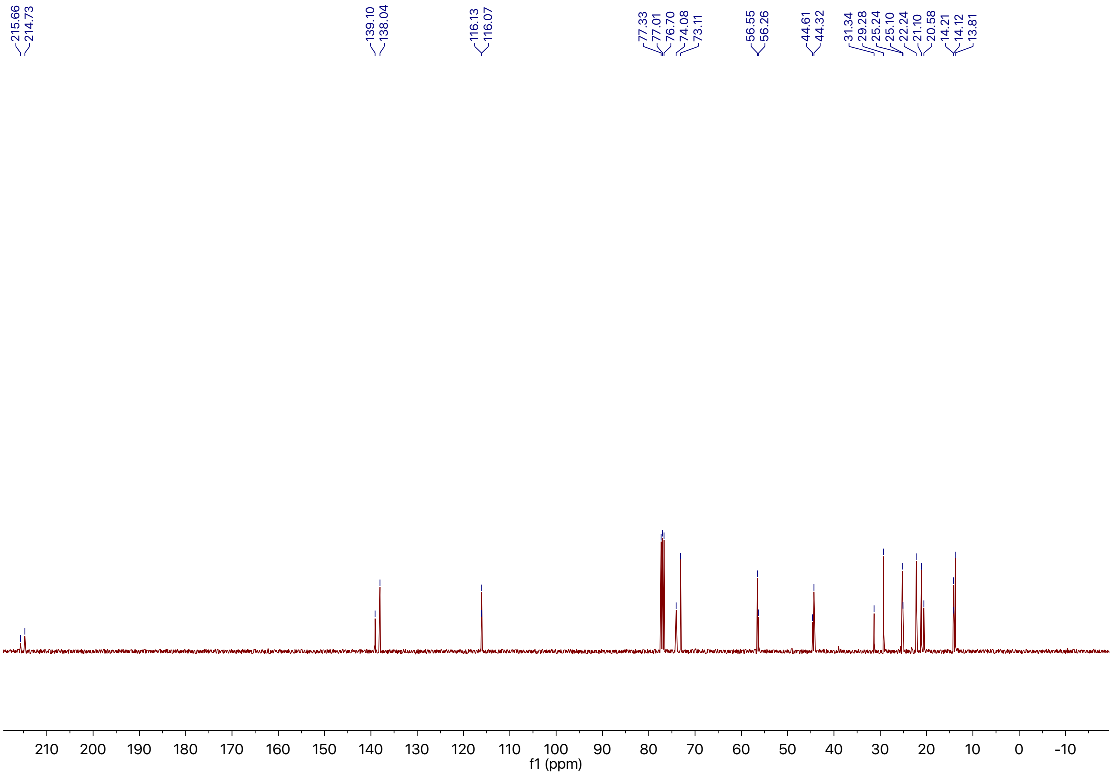

Supplementary Figure 28. ^{13}C NMR spectra ($CDCl_3$, 100 MHz) of **8f**

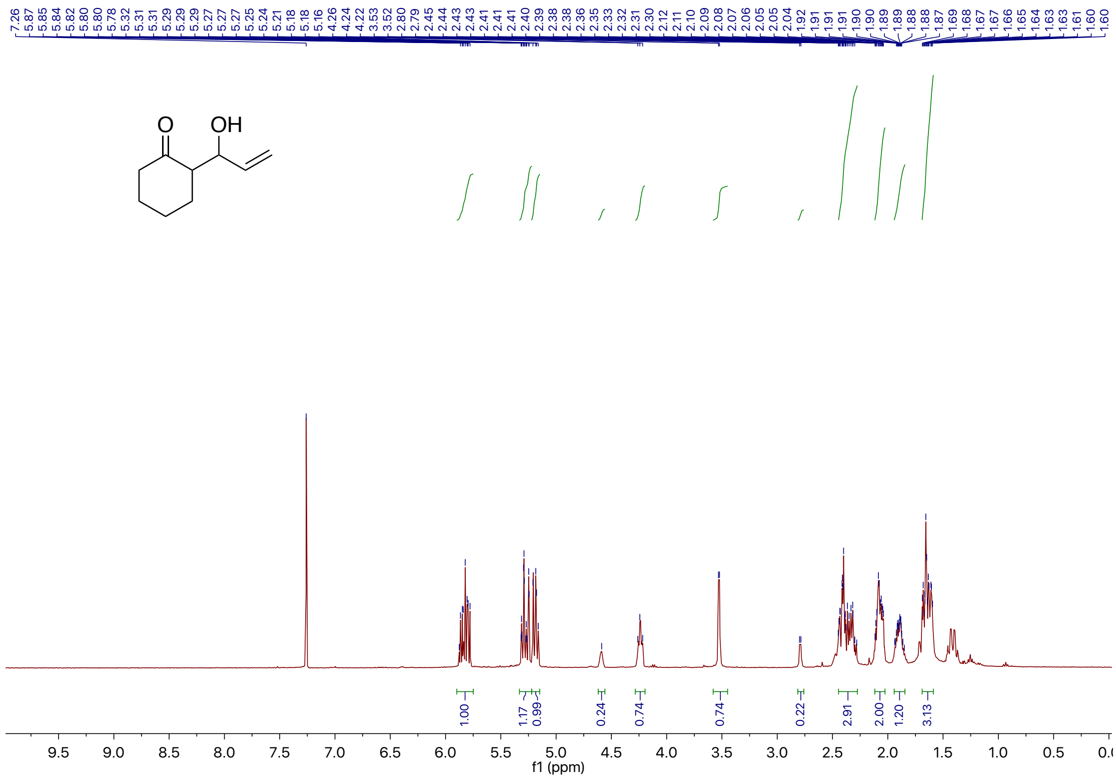

Supplementary Figure 29. ^{1}H NMR spectra ($CDCl_3$, 400 MHz) of **8g**

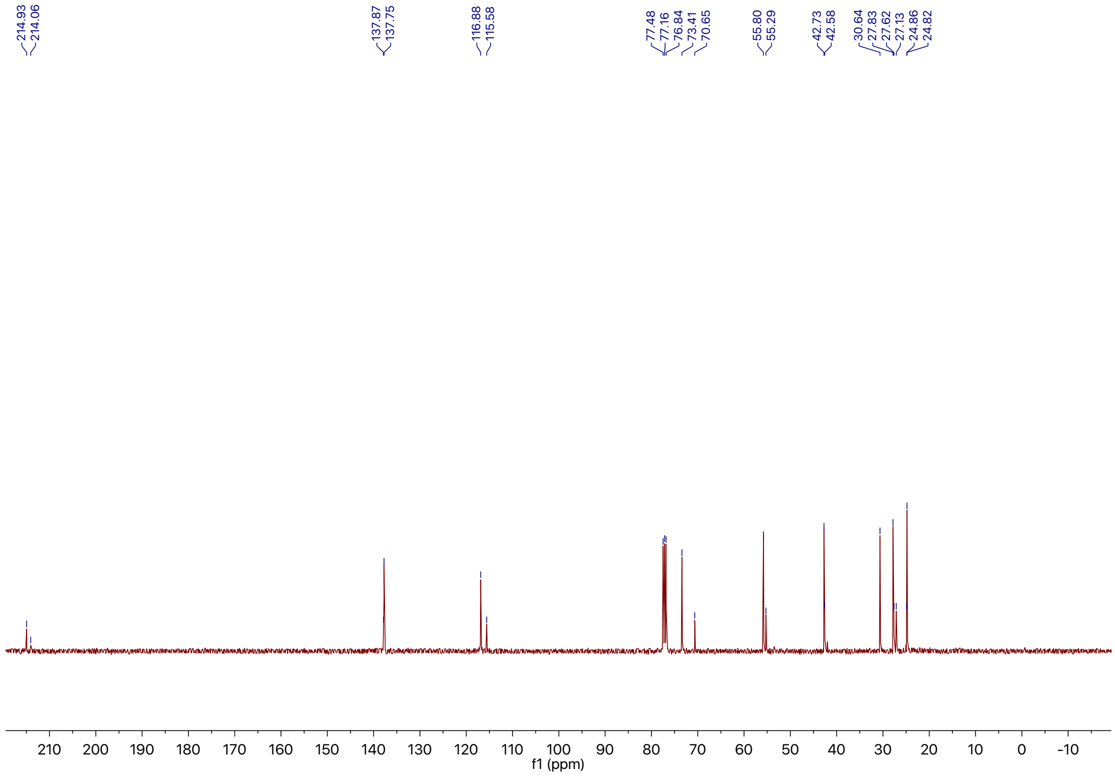

Supplementary Figure 30. ^{13}C NMR spectra ($CDCl_3$, 100 MHz) of **8g**

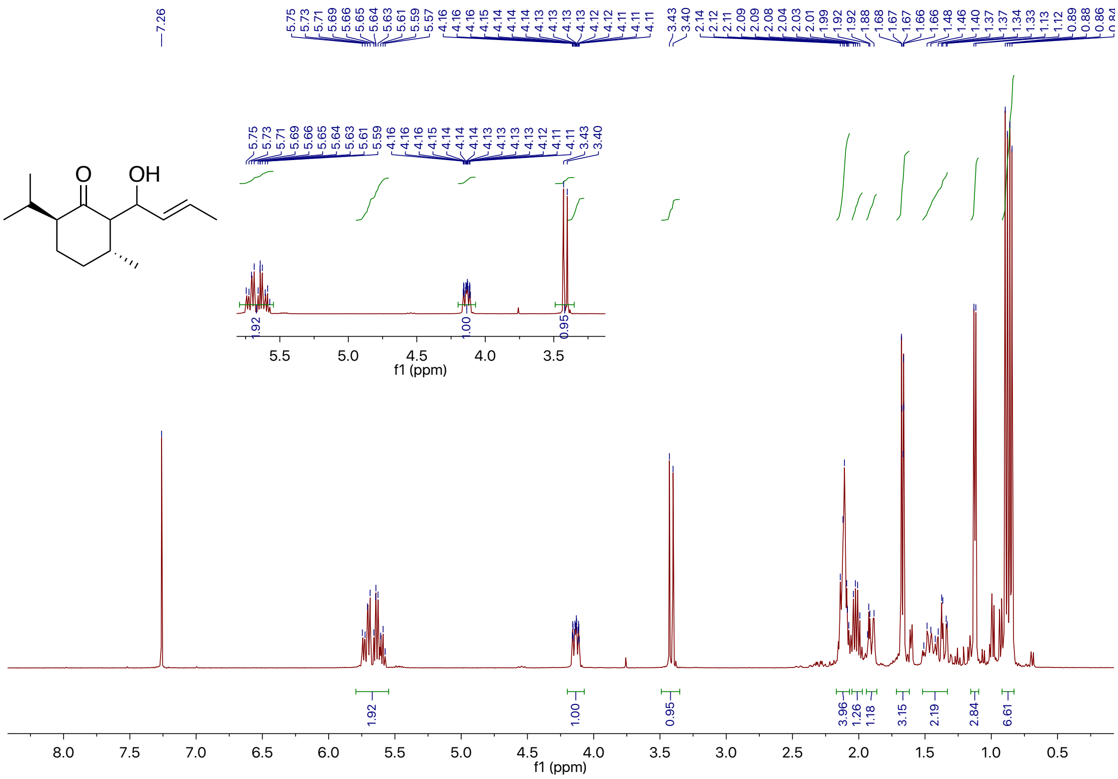

Supplementary Figure 31. ^{1}H NMR spectra ($CDCl_3$, 400 MHz) of **8h**

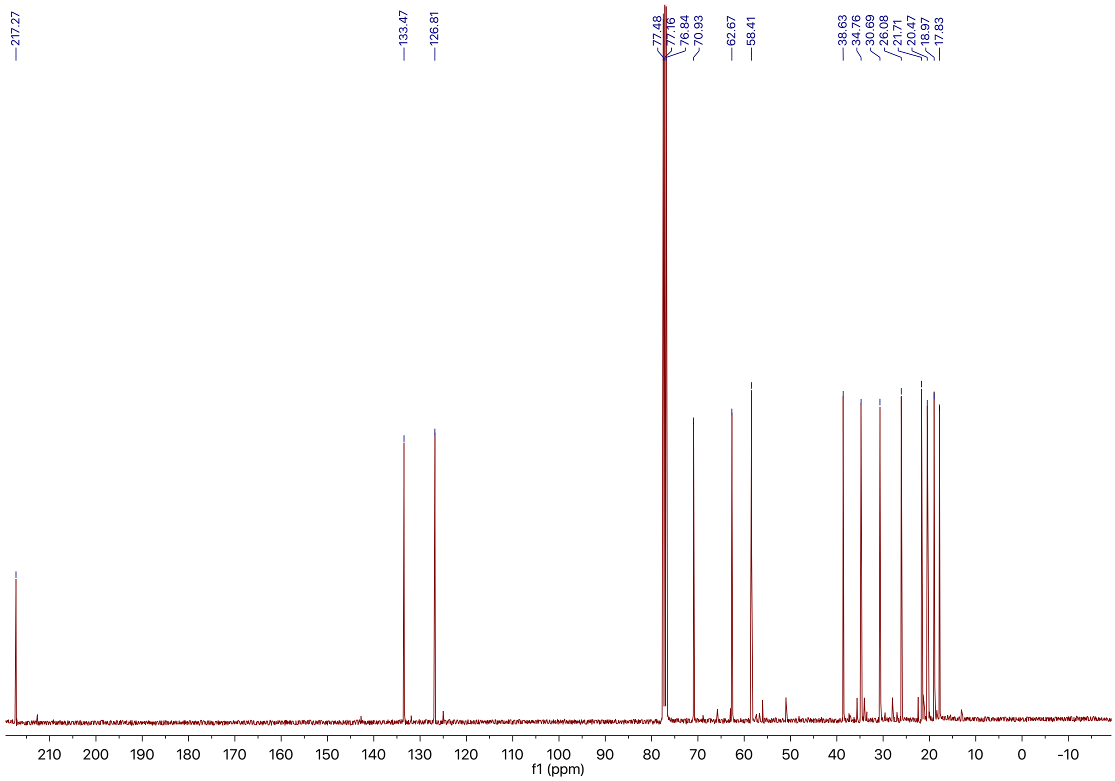

Supplementary Figure 32. ^{13}C NMR spectra ($CDCl_3$, 100 MHz) of **8h**

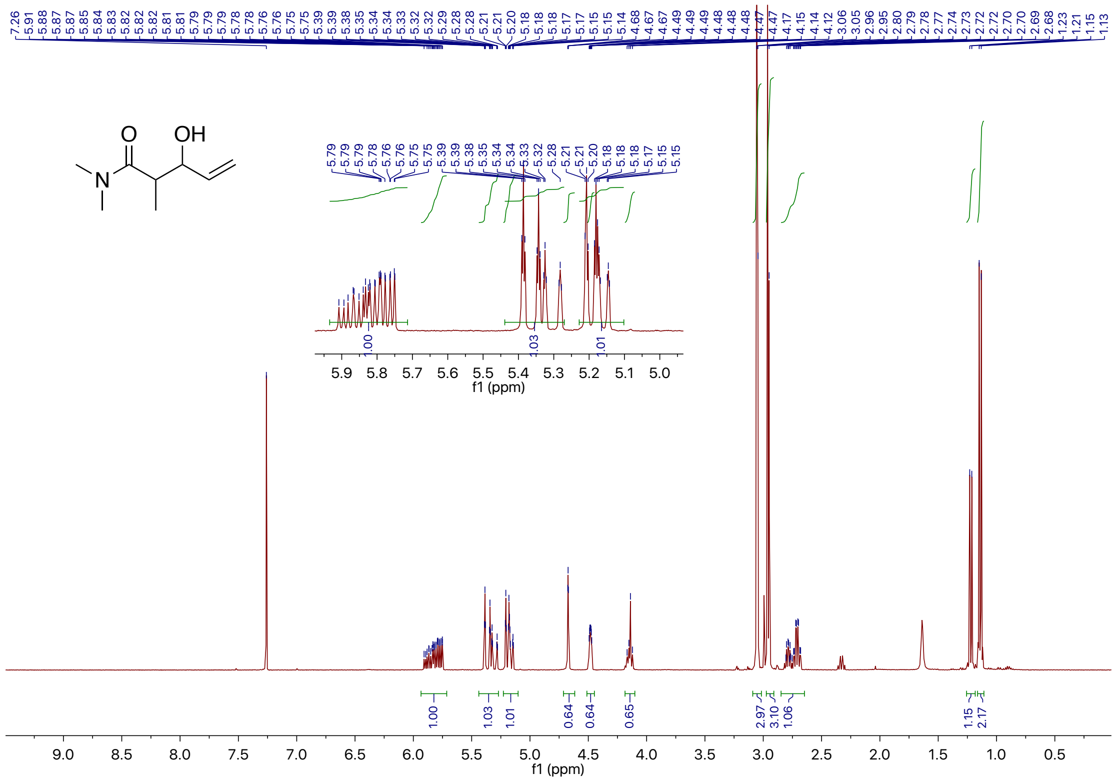

Supplementary Figure 33. ^{1}H NMR spectra ($CDCl_3$, 400 MHz) of **8j**

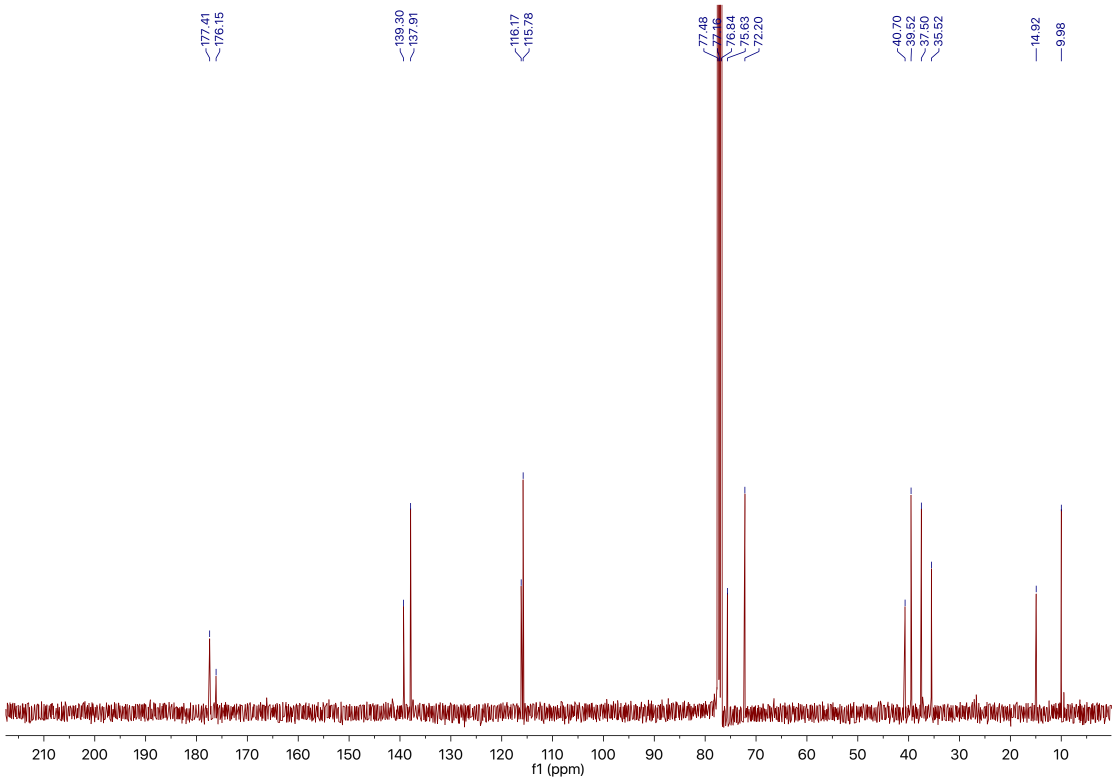

Supplementary Figure 34. ^{13}C NMR spectra ($CDCl_3$, 100 MHz) of **8j**

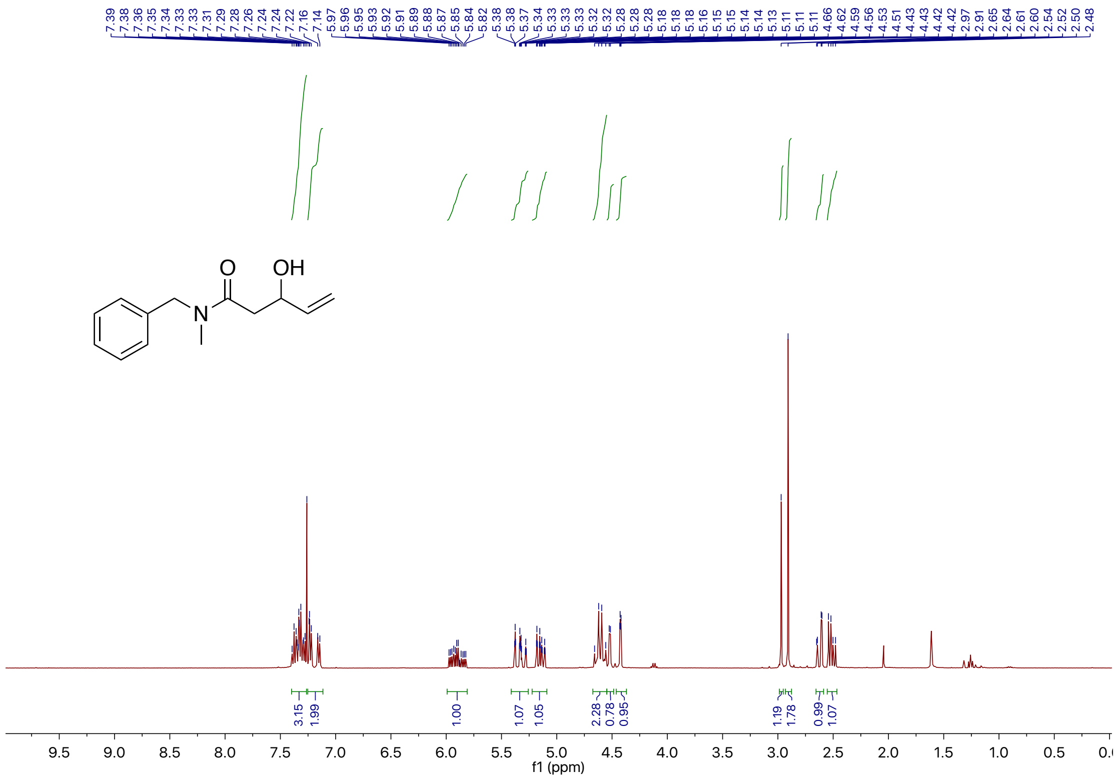

Supplementary Figure 35. ^{1}H NMR spectra ($CDCl_3$, 400 MHz) of **8k**

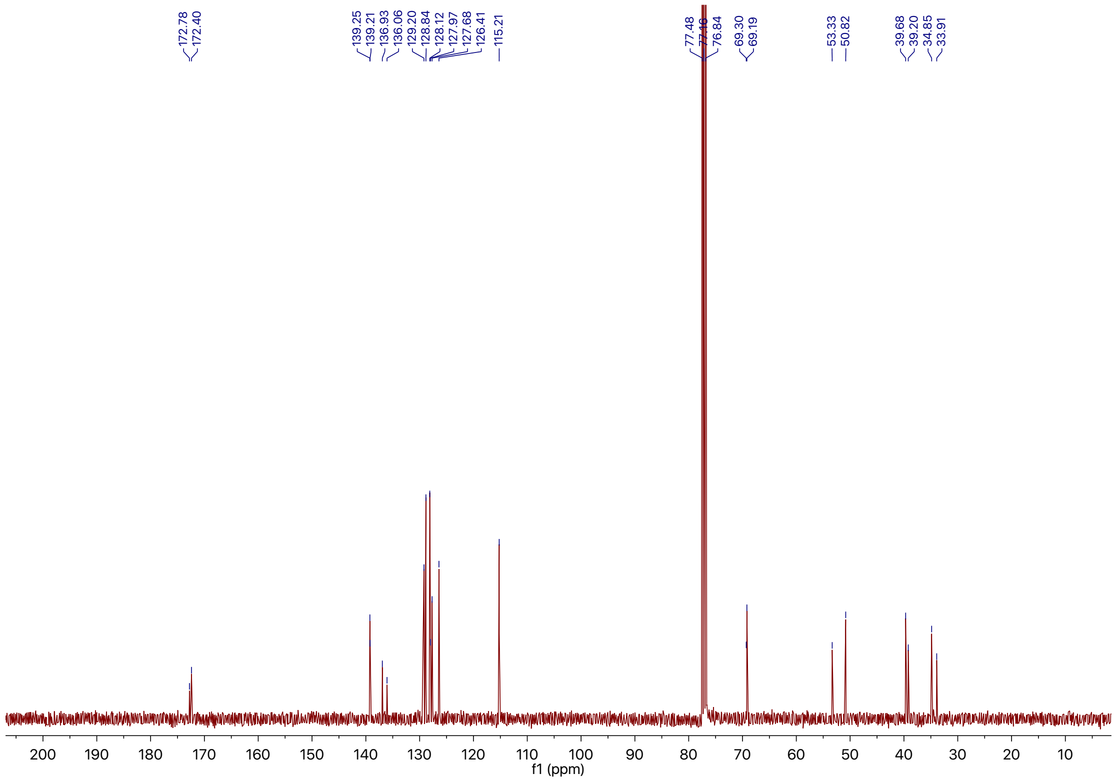

Supplementary Figure 36. ^{13}C NMR spectra ($CDCl_3$, 100 MHz) of **8k**

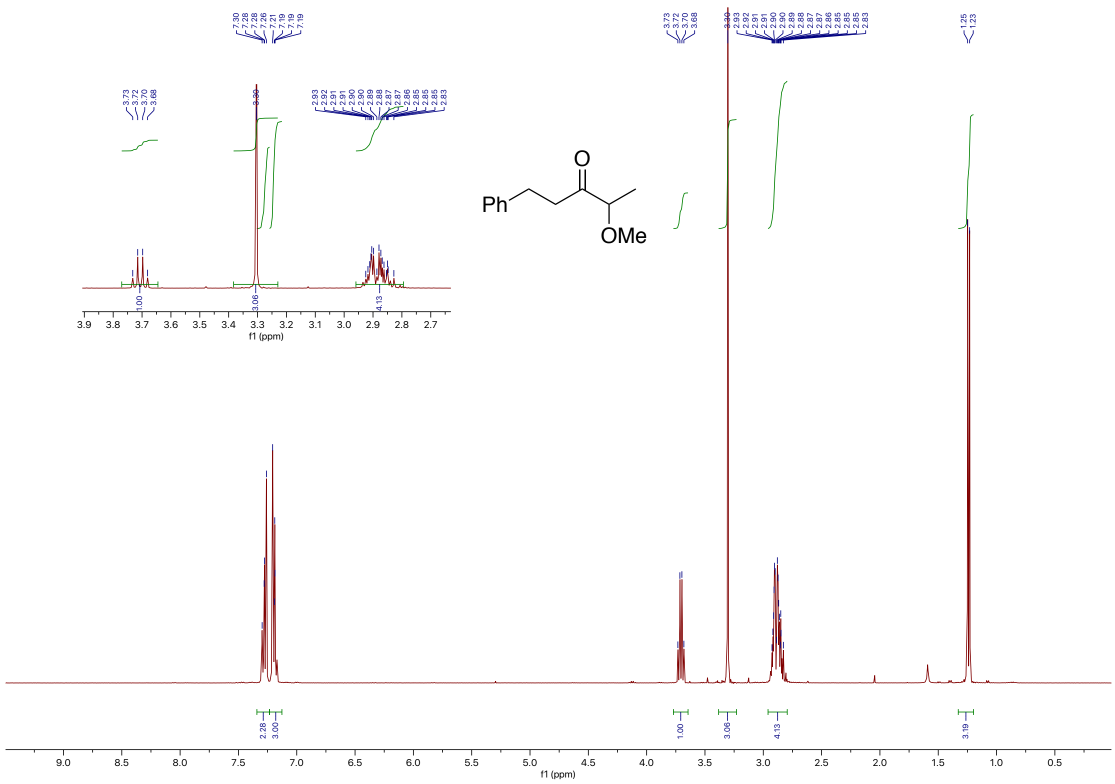

Supplementary Figure 37. ^{1}H NMR spectra ($CDCl_3$, 400 MHz) of **2a**

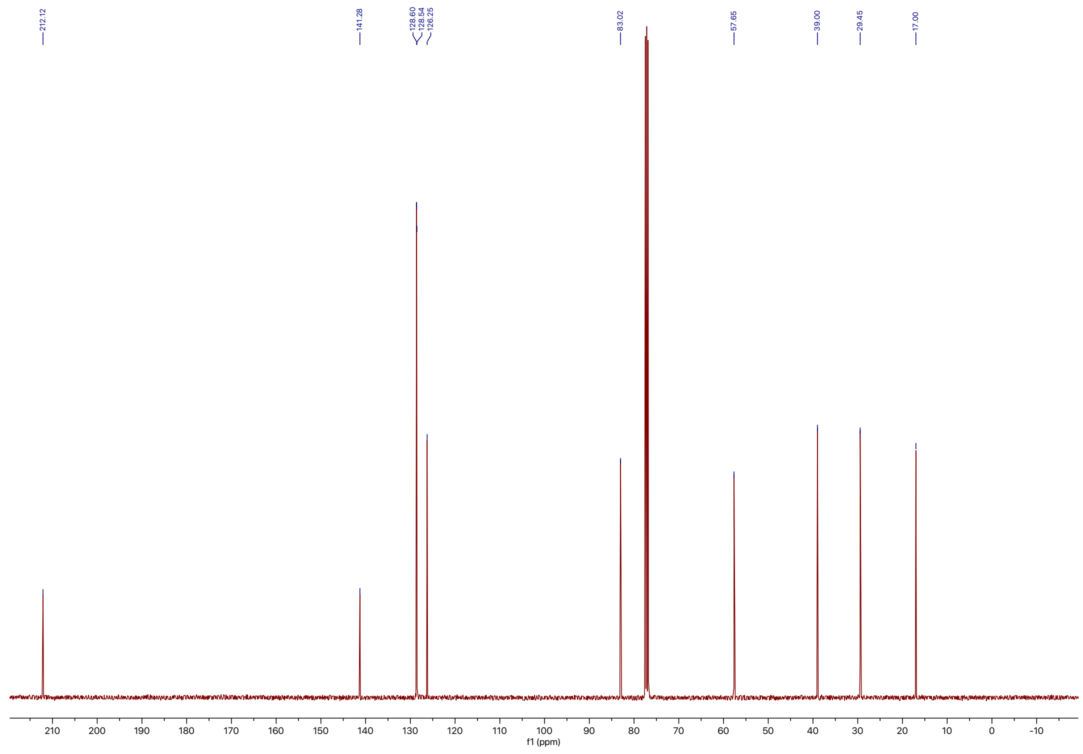

Supplementary Figure 38. ^{13}C NMR spectra ($CDCl_3$, 100 MHz) of **2a**

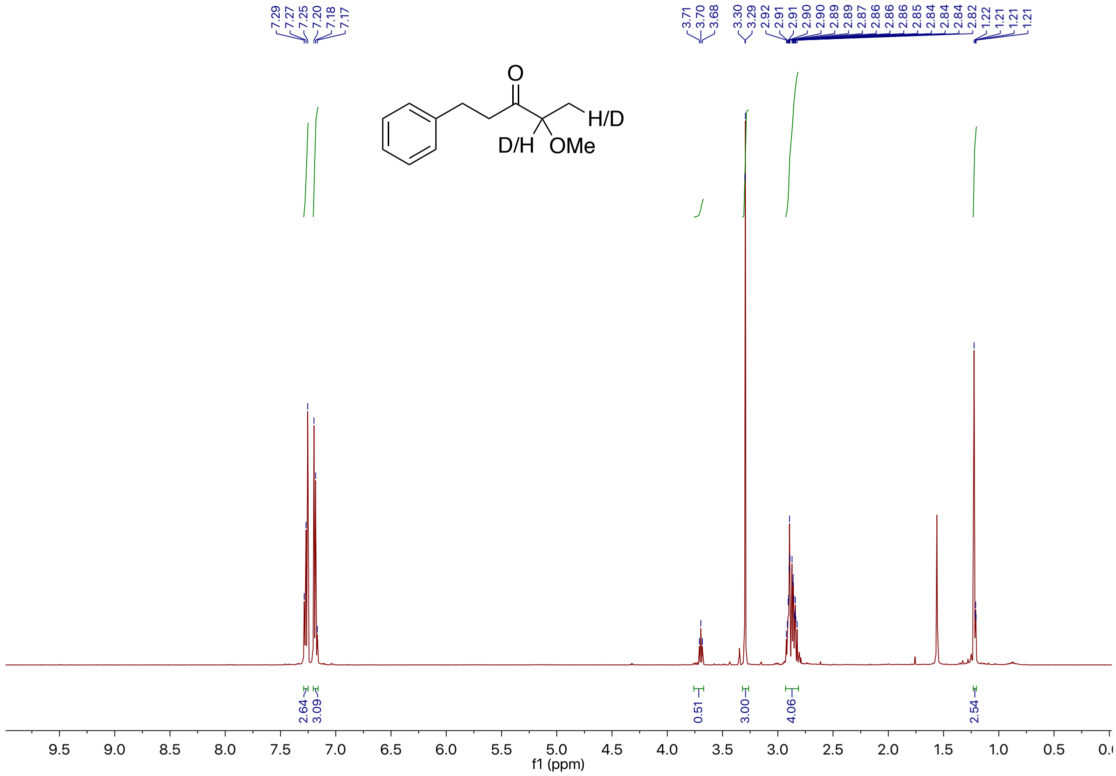

Supplementary Figure 39. ^{1}H NMR spectra ($CDCl_3$, 400 MHz) of **2a-*d***

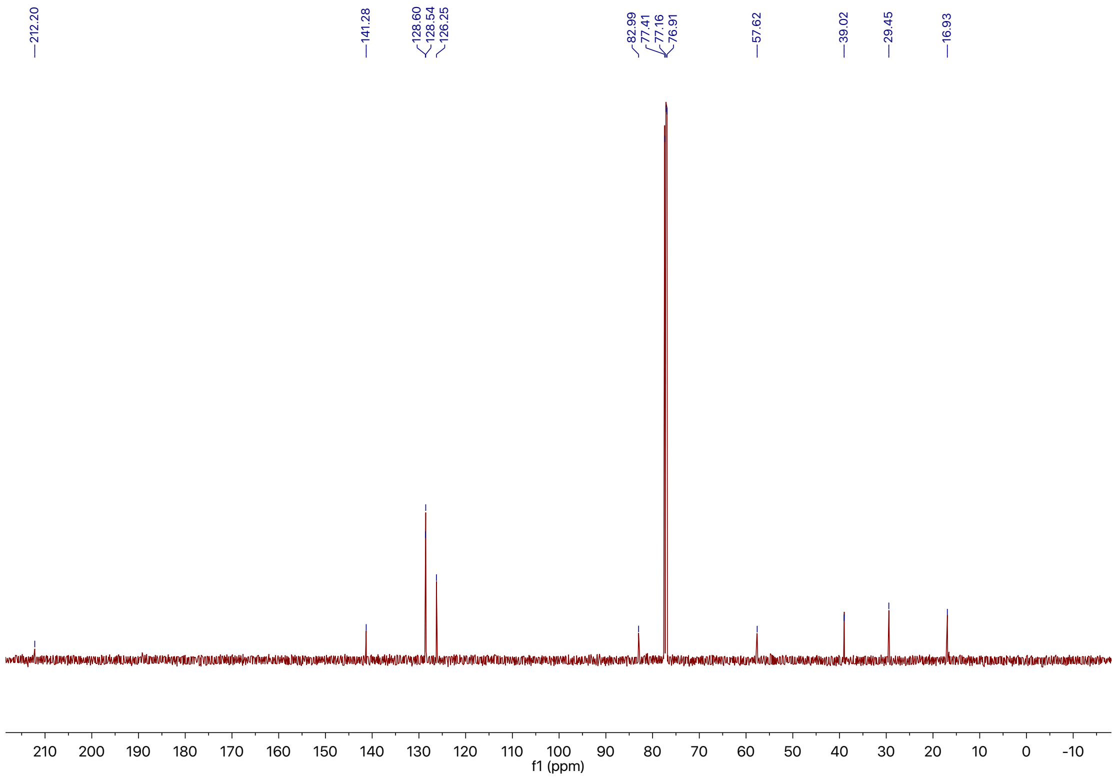

Supplementary Figure 40. ^{13}C NMR spectra ($CDCl_3$, 100 MHz) of **2a-*d***

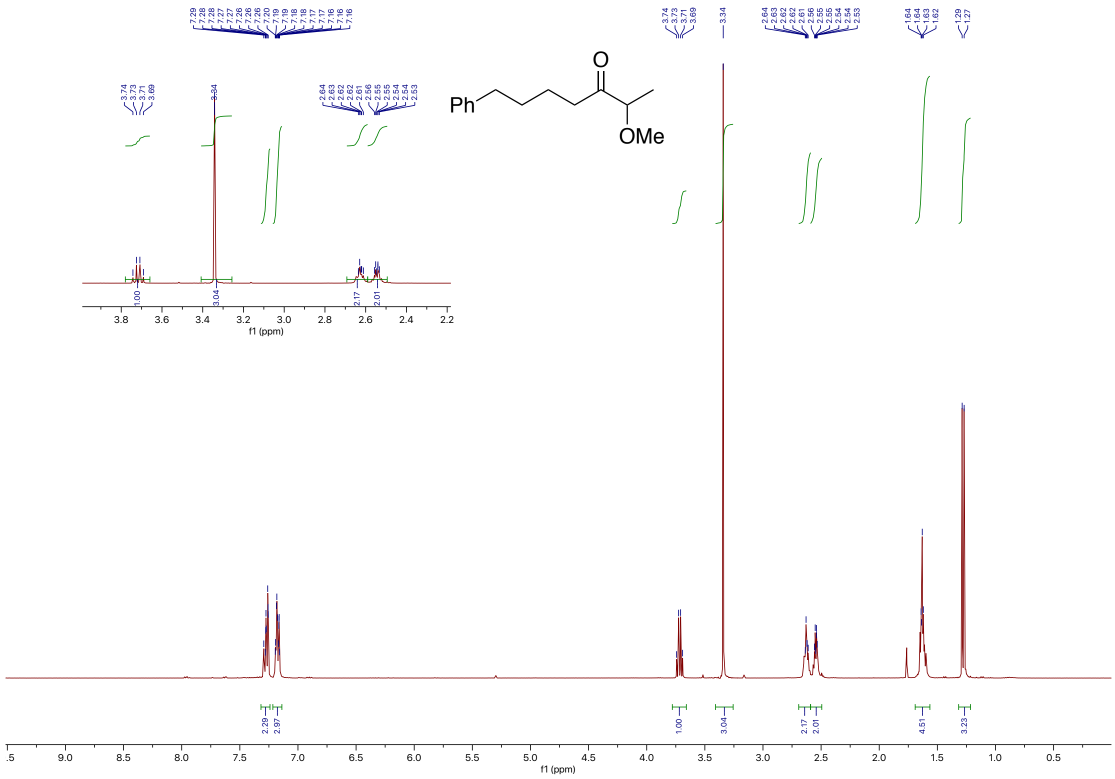

Supplementary Figure 41. ^{1}H NMR spectra ($CDCl_3$, 400 MHz) of **2b**

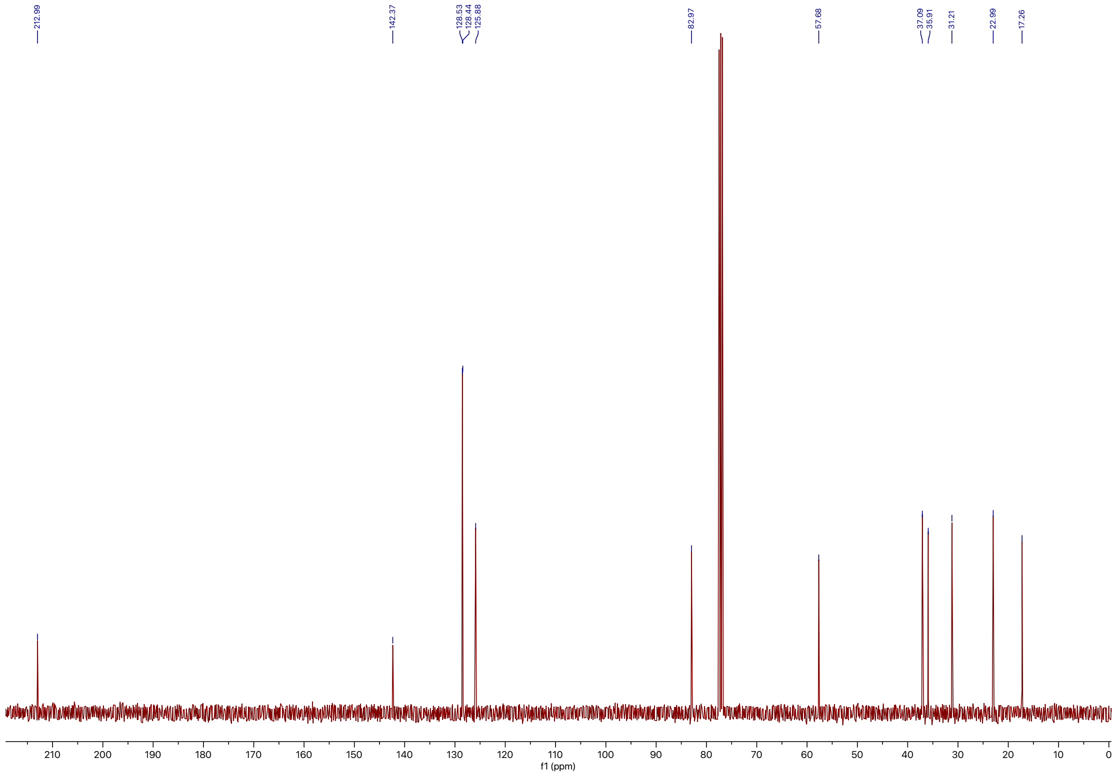

Supplementary Figure 42. ^{13}C NMR spectra ($CDCl_3$, 100 MHz) of **2b**

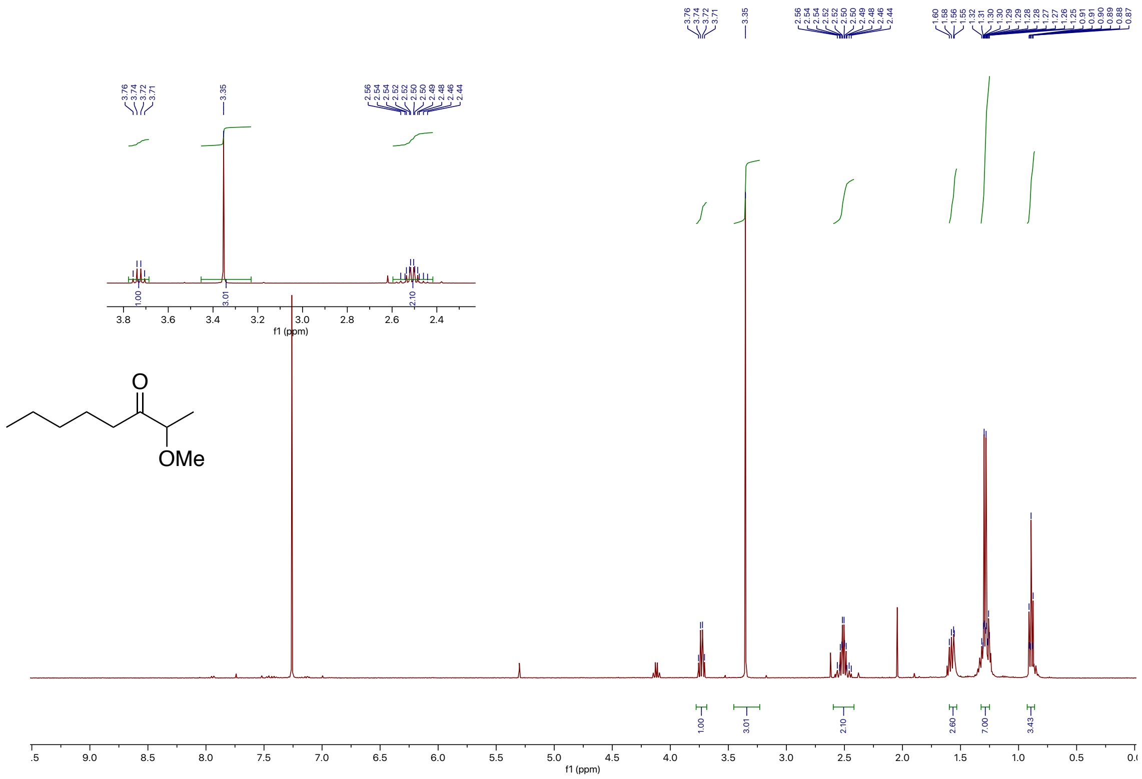

Supplementary Figure 43. ^{1}H NMR spectra ($CDCl_3$, 400 MHz) of **2c**. The compound is volatile and it cannot be kept for long in the vacuum. For this reason, some solvents can be spotted in ^{1}H NMR.

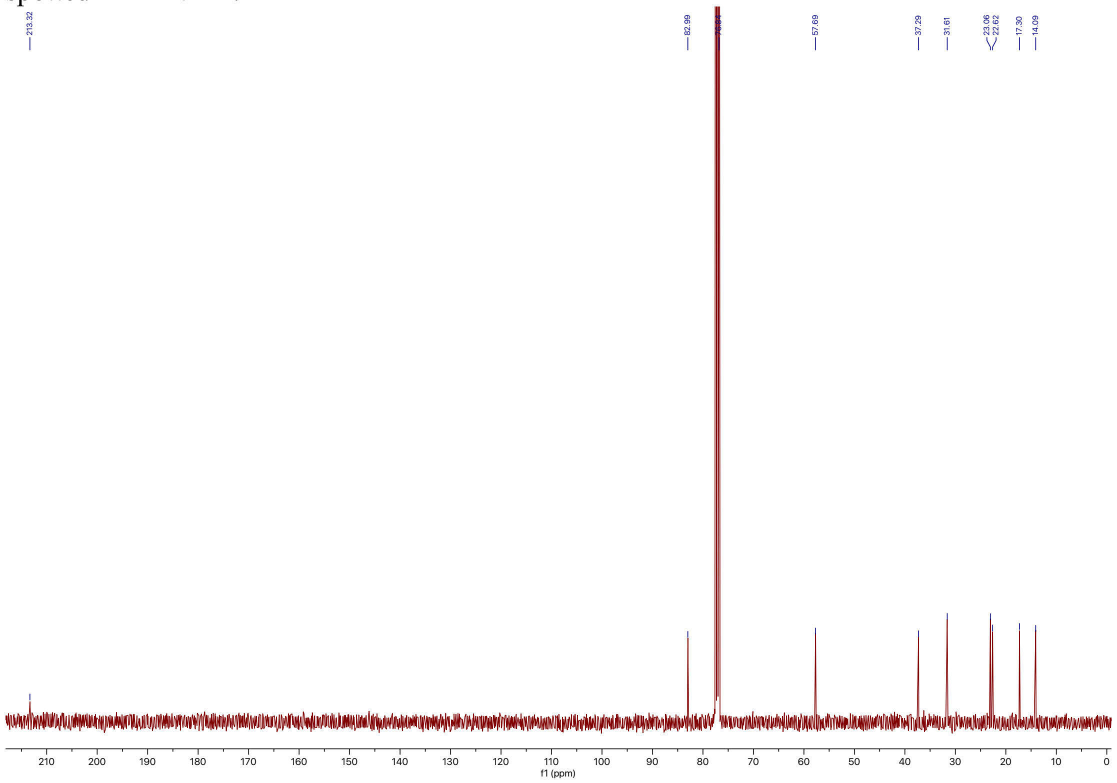

Supplementary Figure 44. ^{13}C NMR spectra ($CDCl_3$, 100 MHz) of **2c**

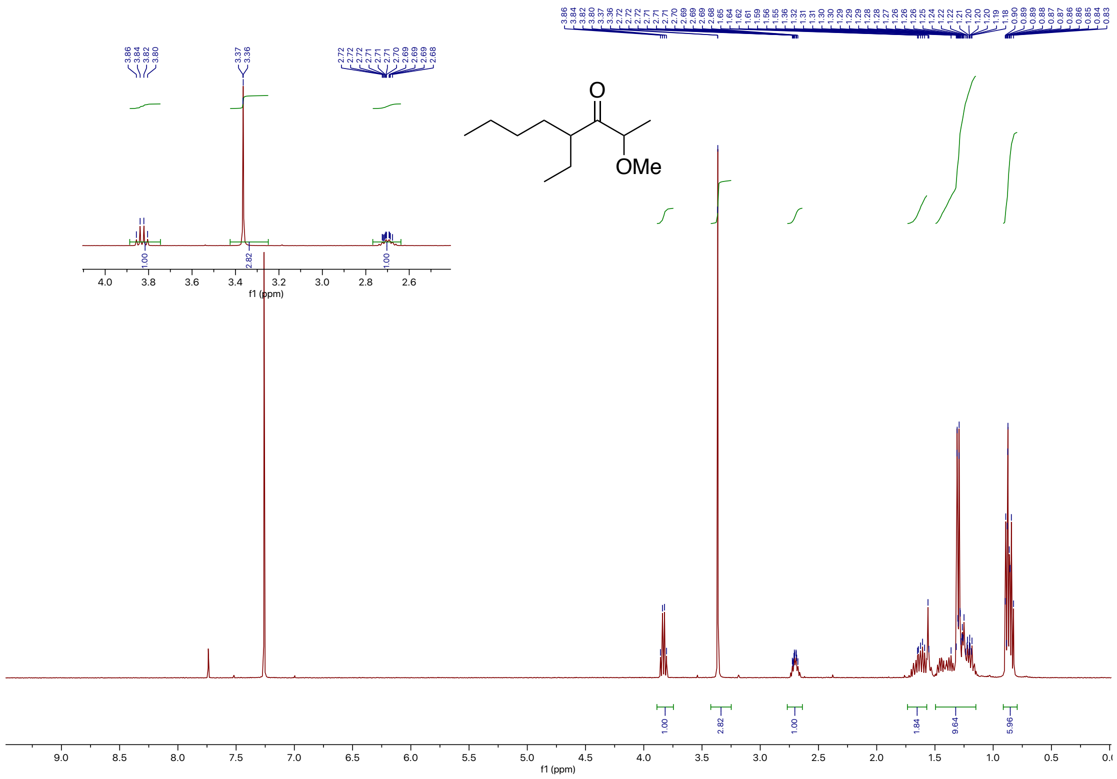

Supplementary Figure 45. ^{1}H NMR spectra ($CDCl_3$, 400 MHz) of **2d**

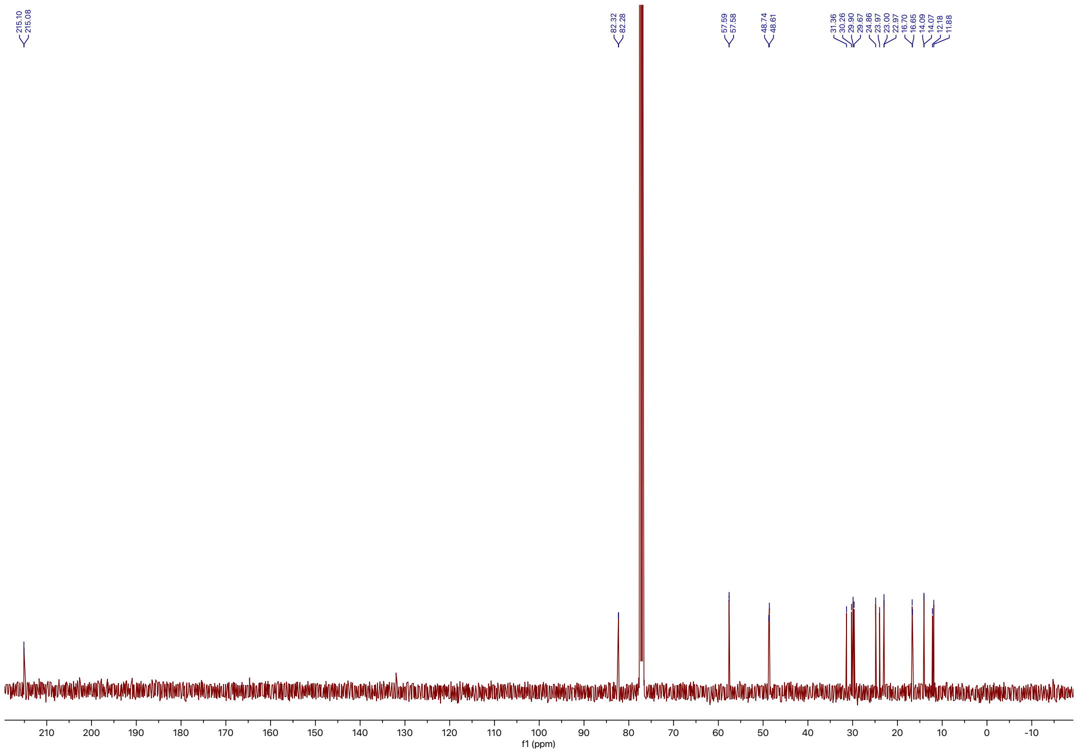

Supplementary Figure 46. ^{13}C NMR spectra ($CDCl_3$, 100 MHz) of **2d**

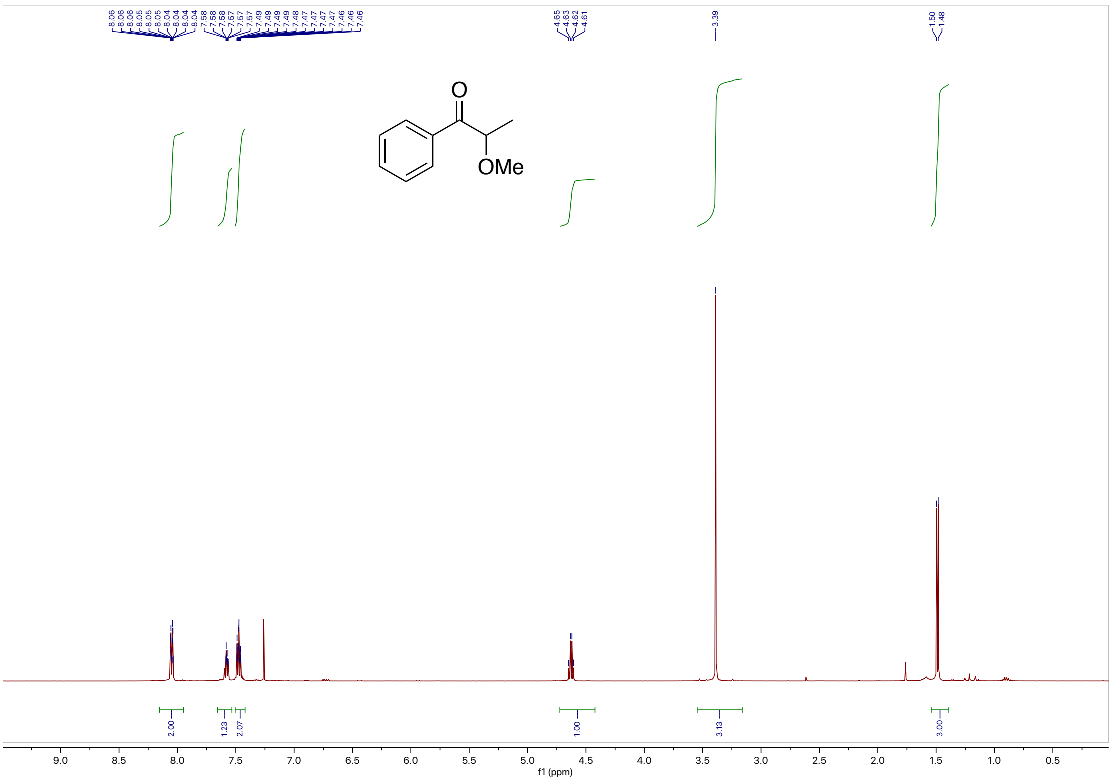

Supplementary Figure 47. ^{1}H NMR spectra ($CDCl_3$, 400 MHz) of **2e**

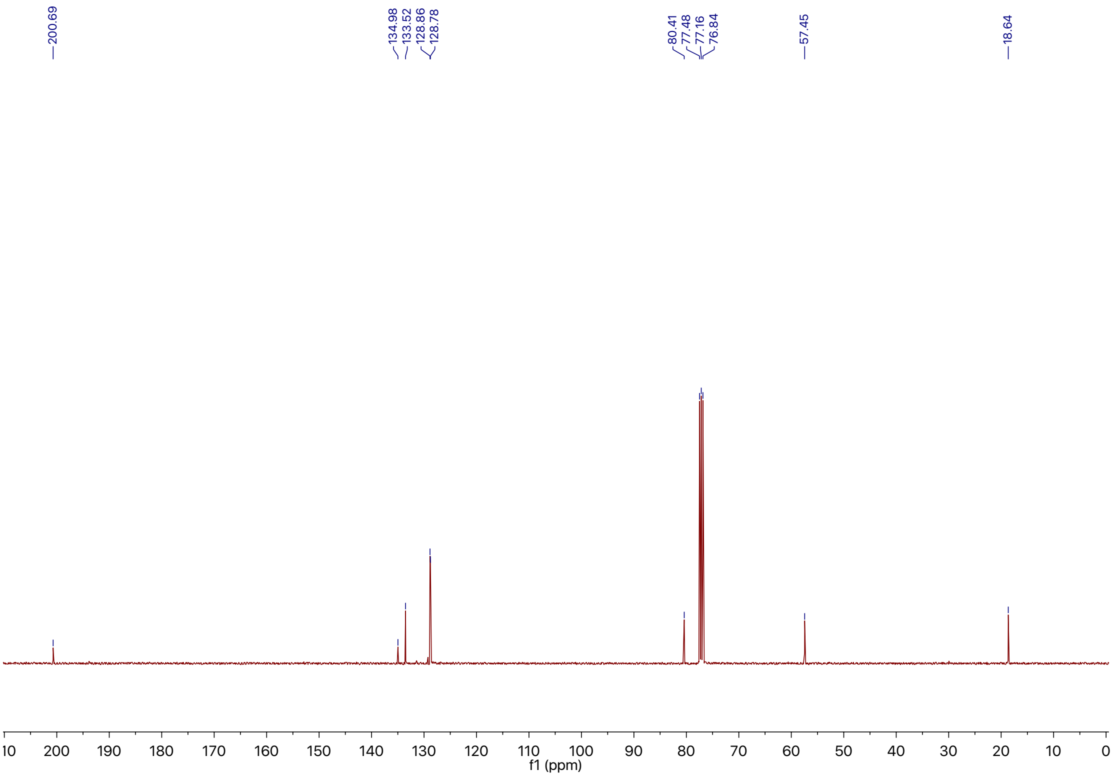

Supplementary Figure 48. ^{13}C NMR spectra ($CDCl_3$, 100 MHz) of **2e**

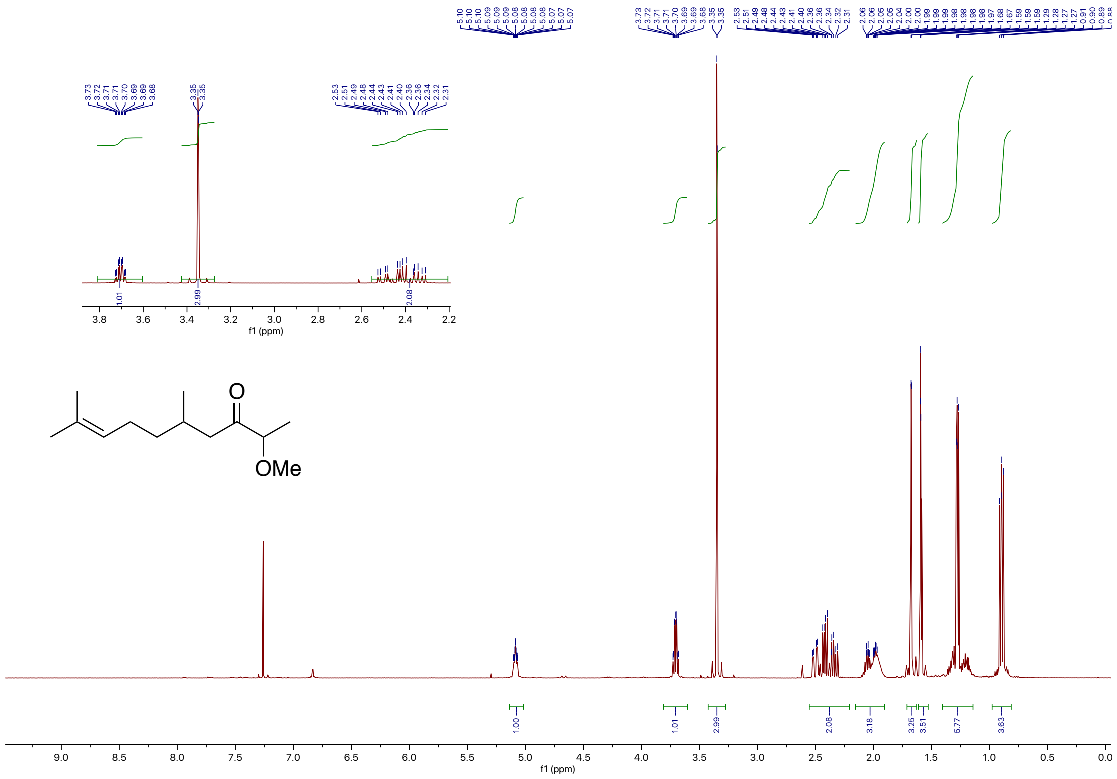

Supplementary Figure 49. ^{1}H NMR spectra ($CDCl_3$, 400 MHz) of **2f**

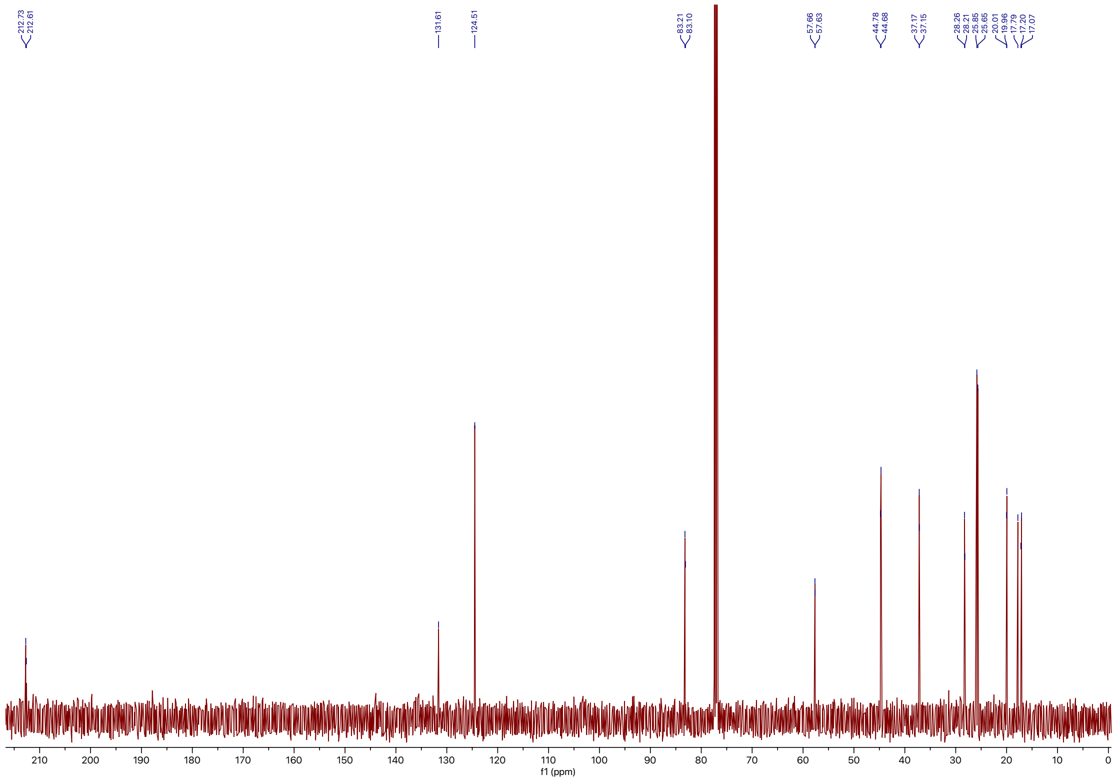

Supplementary Figure 50. ^{13}C NMR spectra ($CDCl_3$, 100 MHz) of **2f**

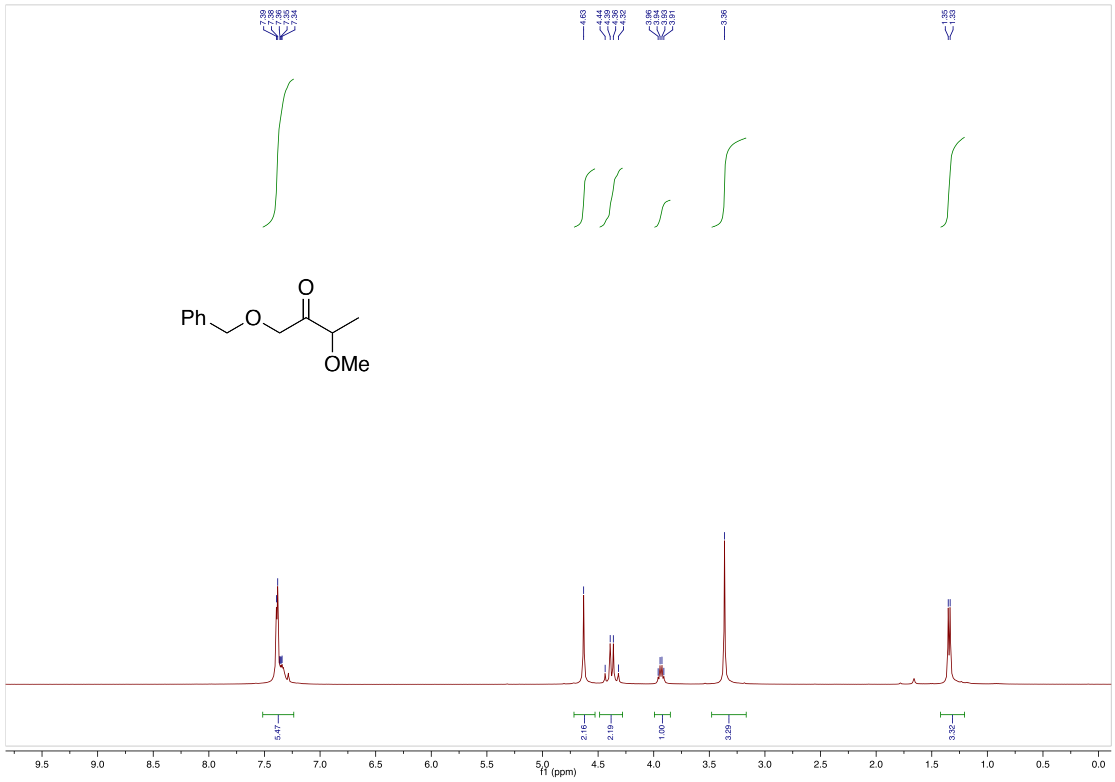

Supplementary Figure 51. ^{1}H NMR spectra ($CDCl_3$, 400 MHz) of **2g**

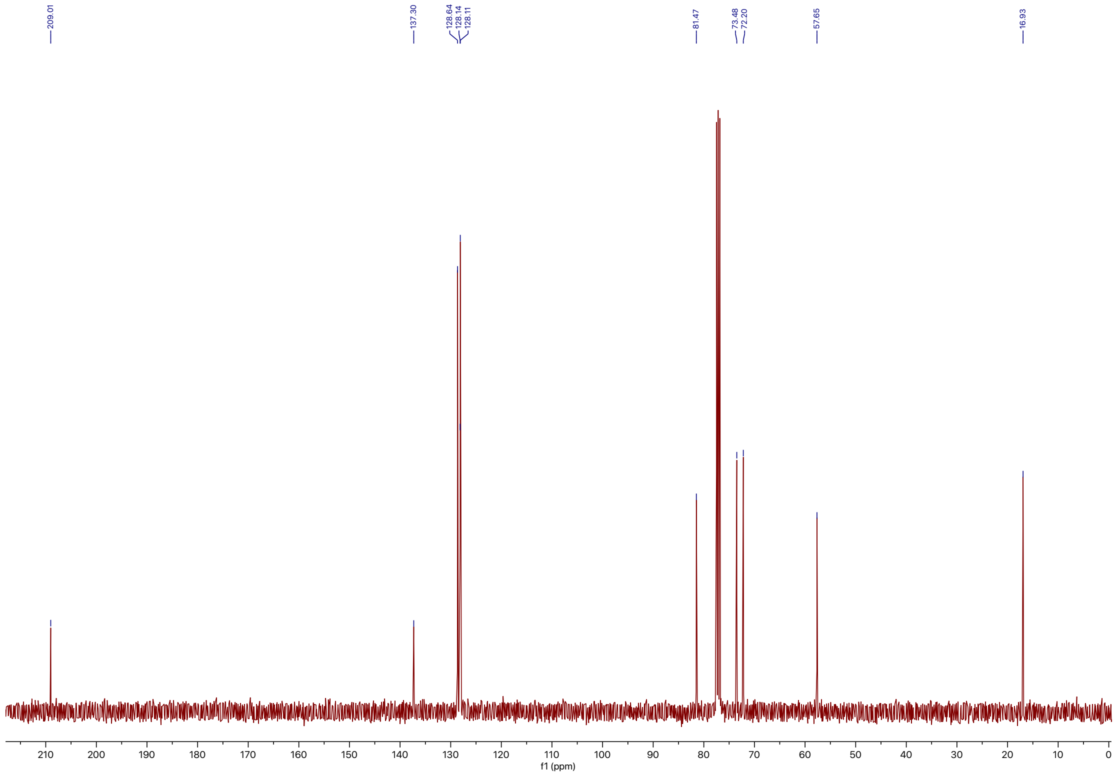

Supplementary Figure 52. ^{13}C NMR spectra ($CDCl_3$, 100 MHz) of **2g**

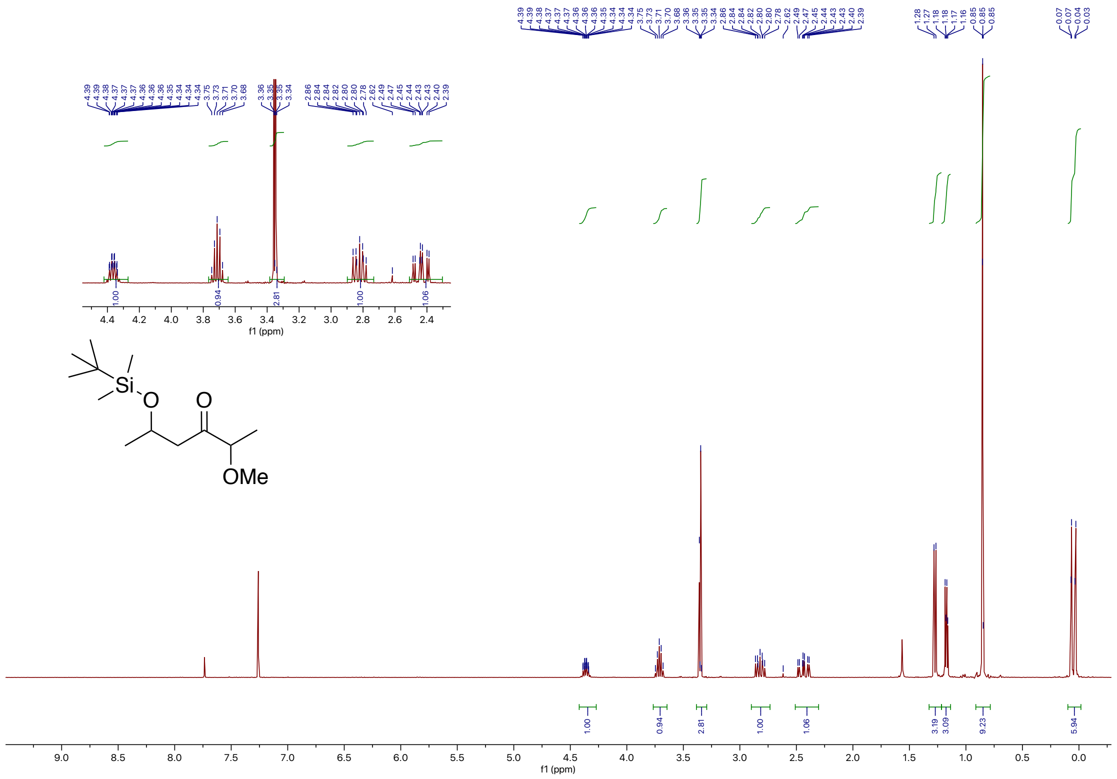

Supplementary Figure 53. ^{1}H NMR spectra ($CDCl_3$, 400 MHz) of **2h**

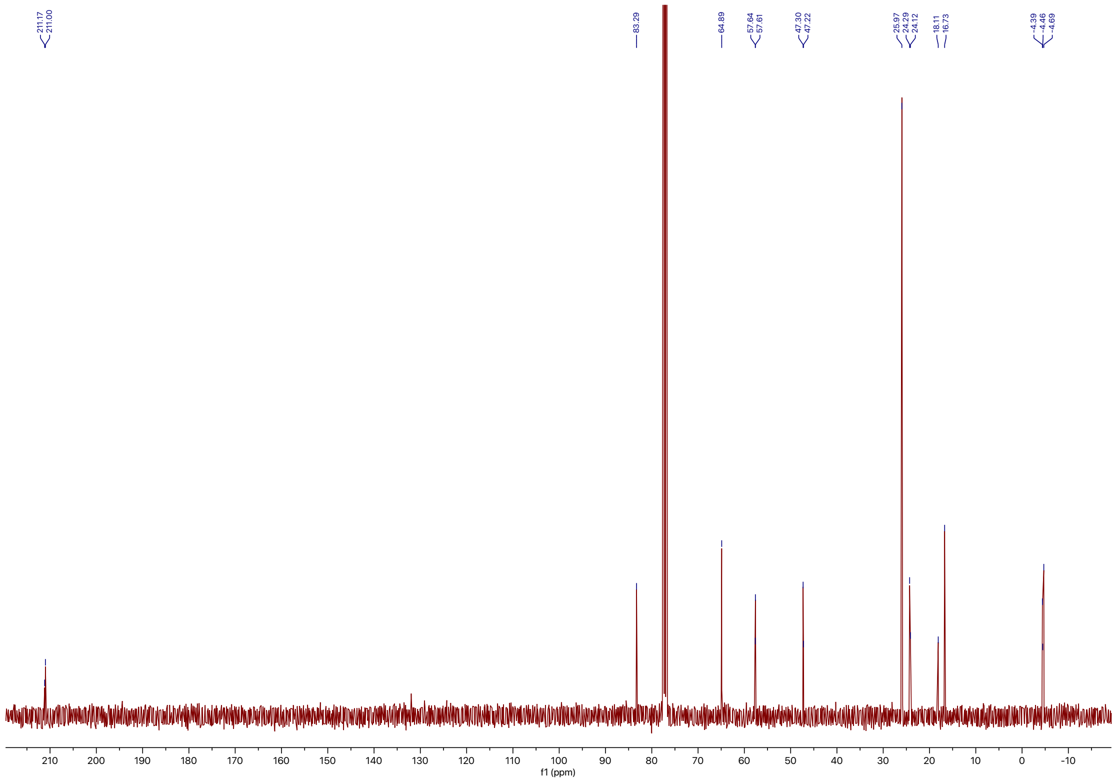

Supplementary Figure 54. ^{13}C NMR spectra ($CDCl_3$, 100 MHz) of **2h**

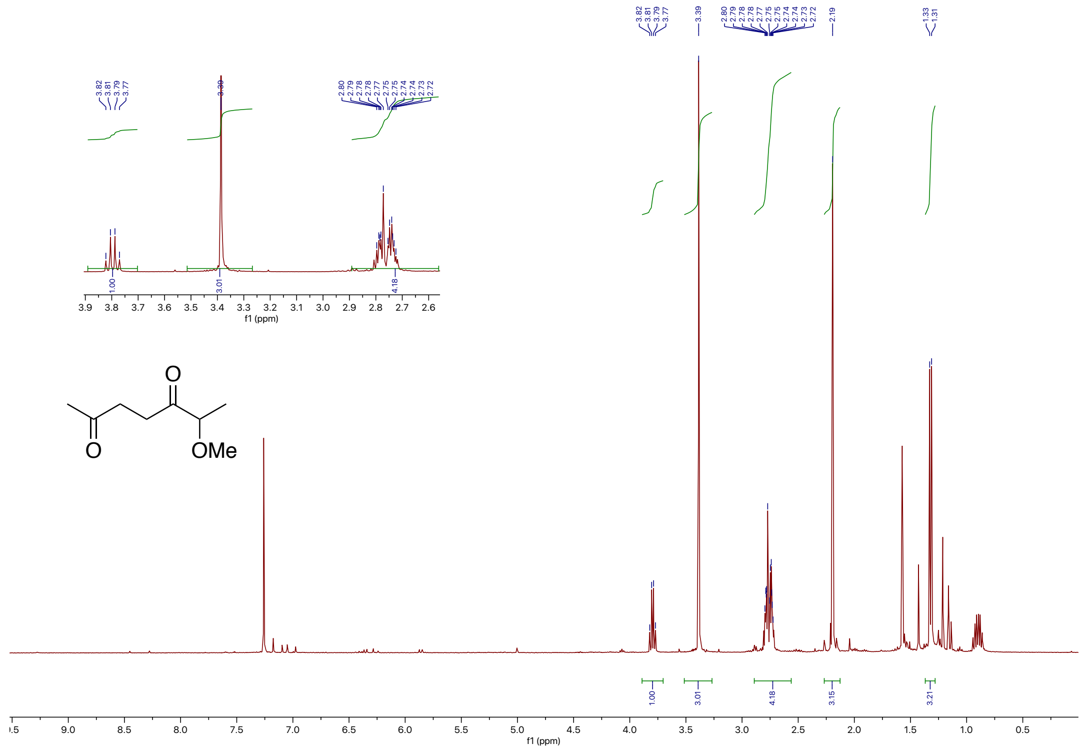

Supplementary Figure 55. ^{1}H NMR spectra ($CDCl_3$, 400 MHz) of **2i**

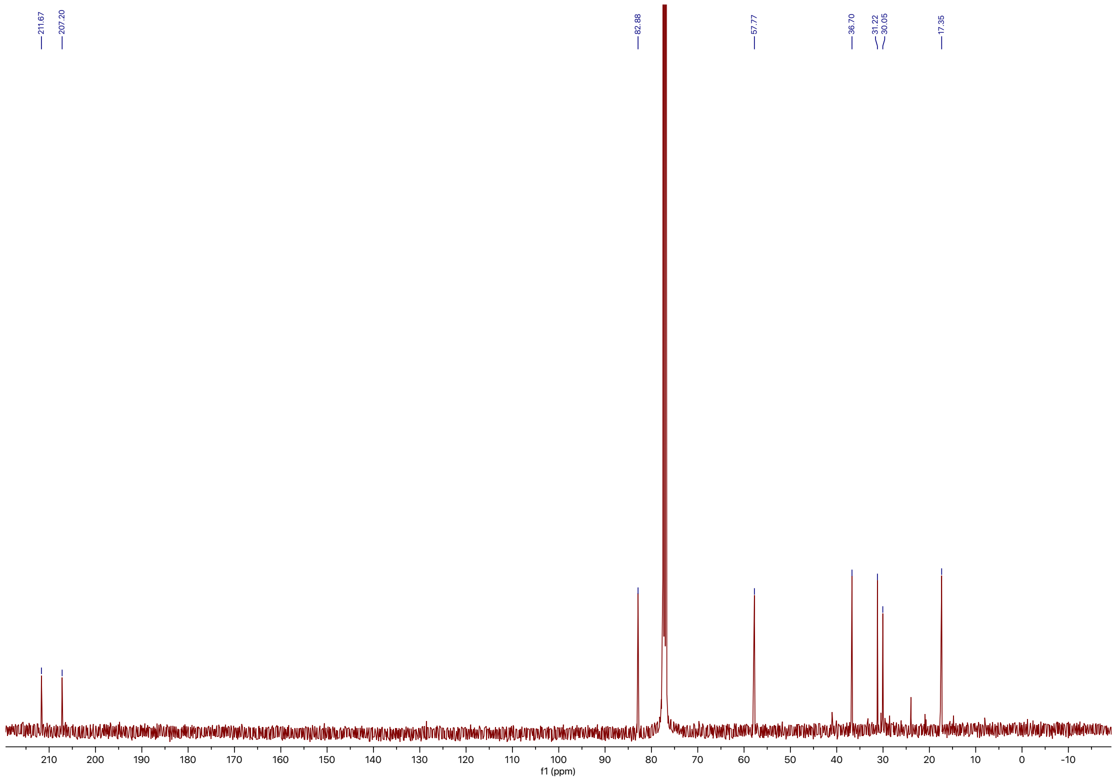

Supplementary Figure 56. ^{13}C NMR spectra ($CDCl_3$, 100 MHz) of **2i**

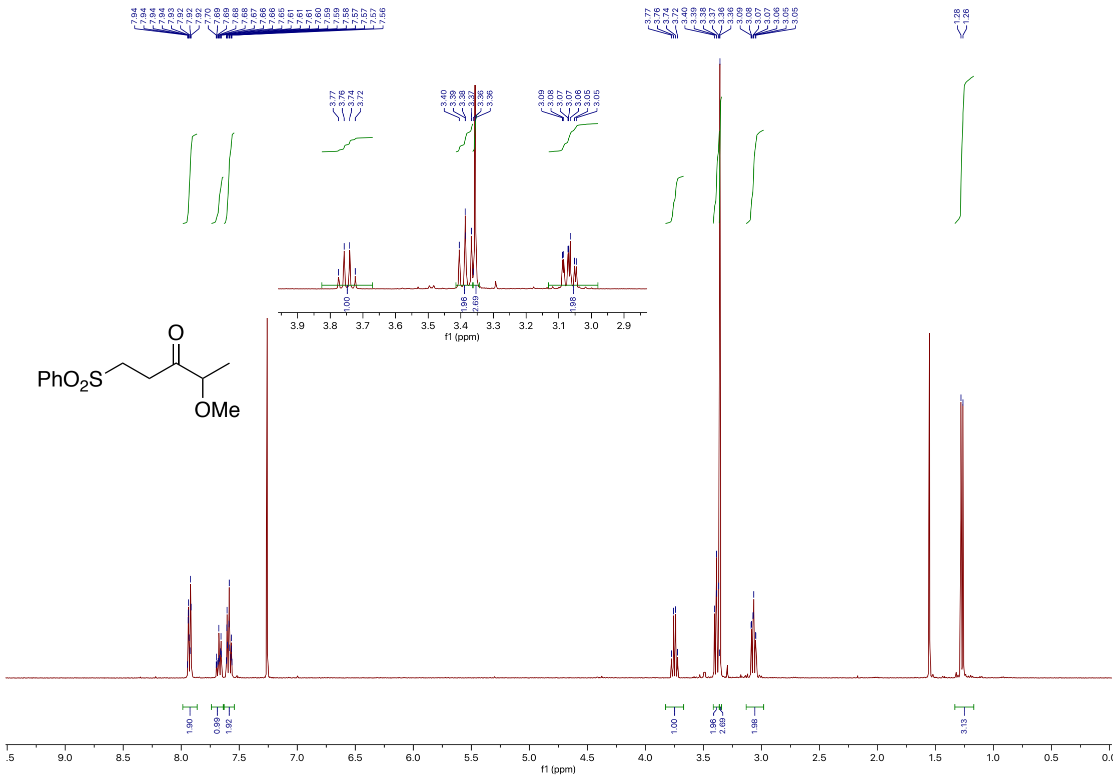

Supplementary Figure 57. ^{1}H NMR spectra ($CDCl_3$, 400 MHz) of **2j**

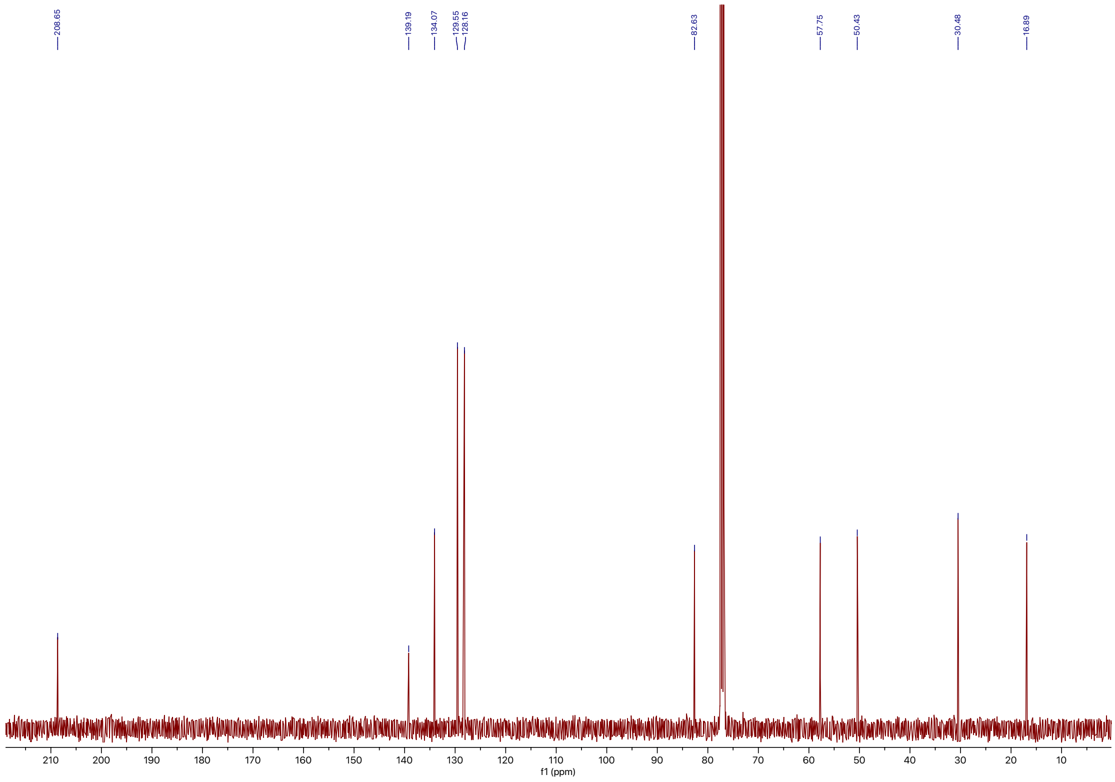

Supplementary Figure 58. ^{13}C NMR spectra ($CDCl_3$, 100 MHz) of **2j**

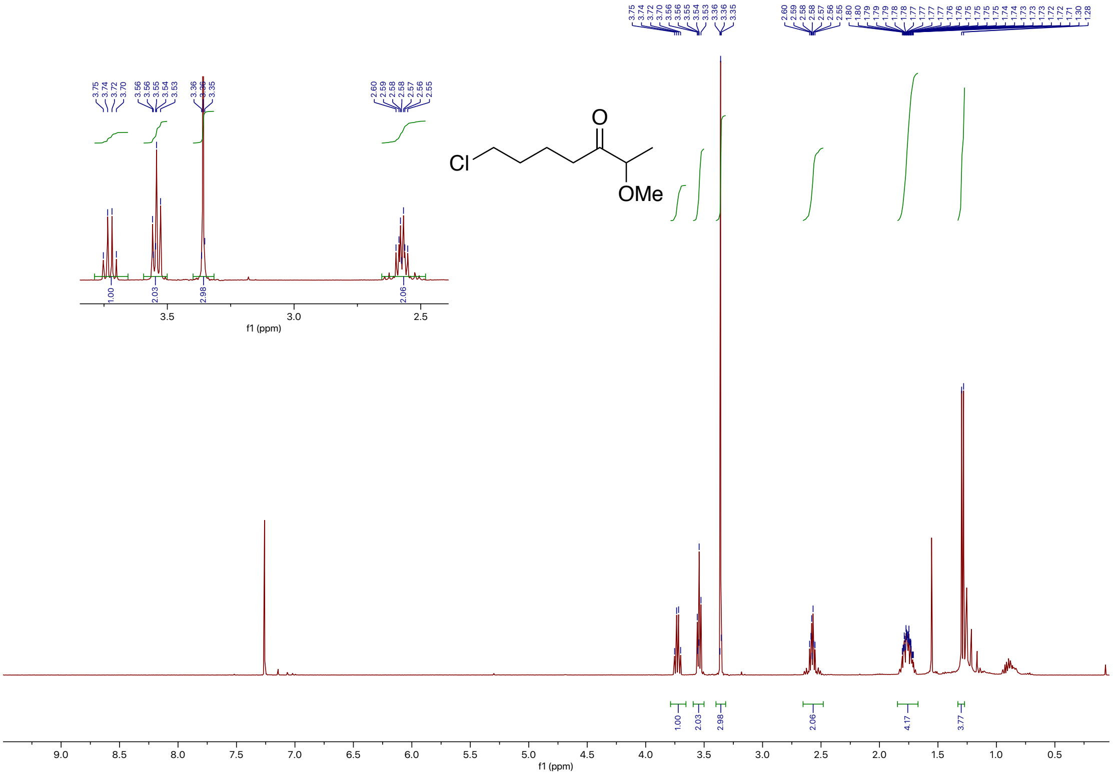

Supplementary Figure 59. ^{1}H NMR spectra ($CDCl_3$, 400 MHz) of **2k**

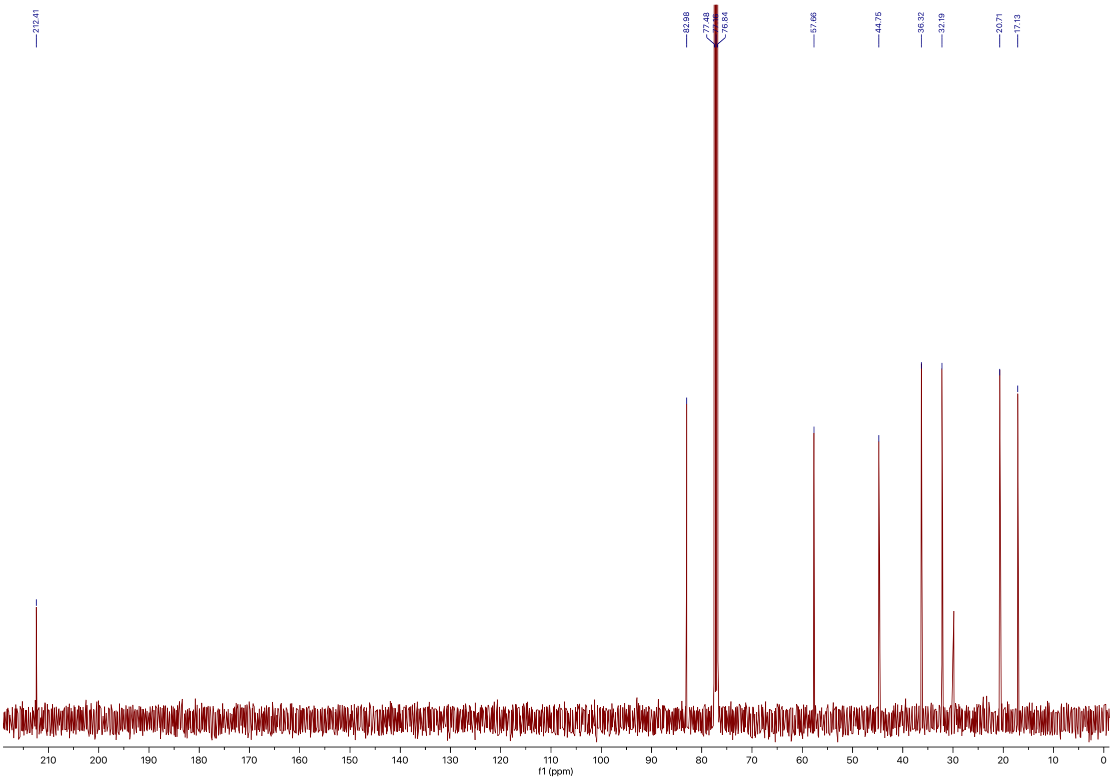

Supplementary Figure 60. ^{13}C NMR spectra ($CDCl_3$, 100 MHz) of **2k**

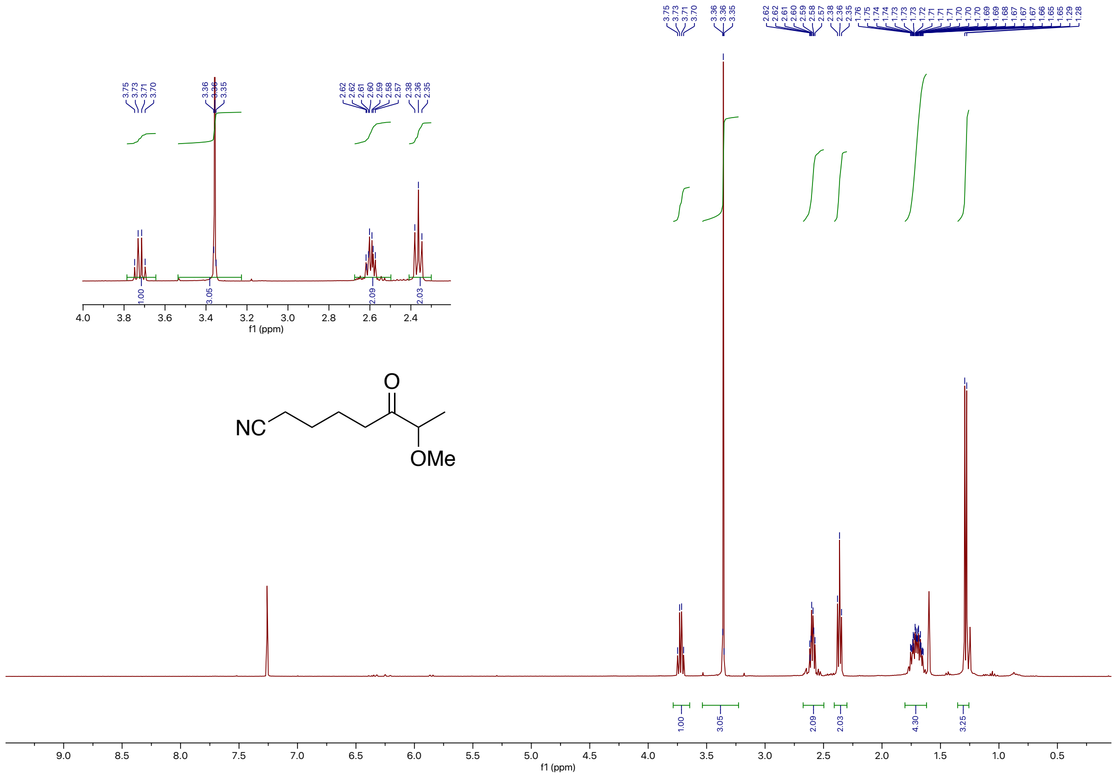

Supplementary Figure 61. ^{1}H NMR spectra ($CDCl_3$, 400 MHz) of **2l**

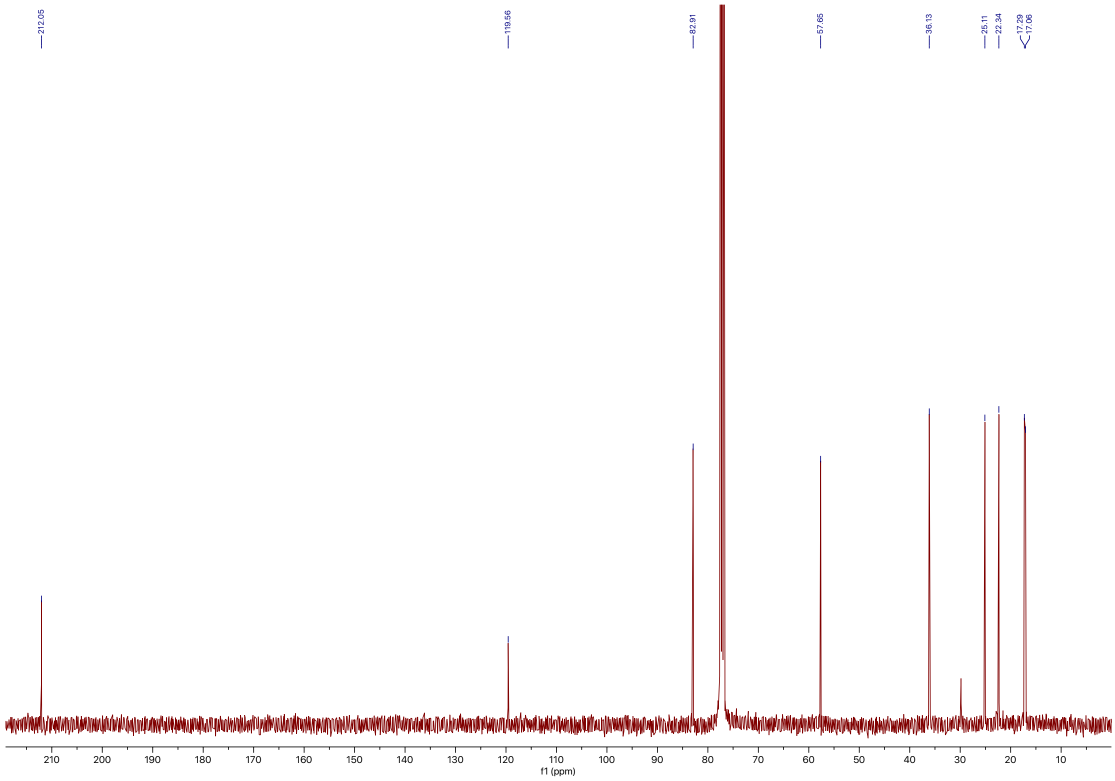

Supplementary Figure 62. ^{13}C NMR spectra ($CDCl_3$, 100 MHz) of **2l**

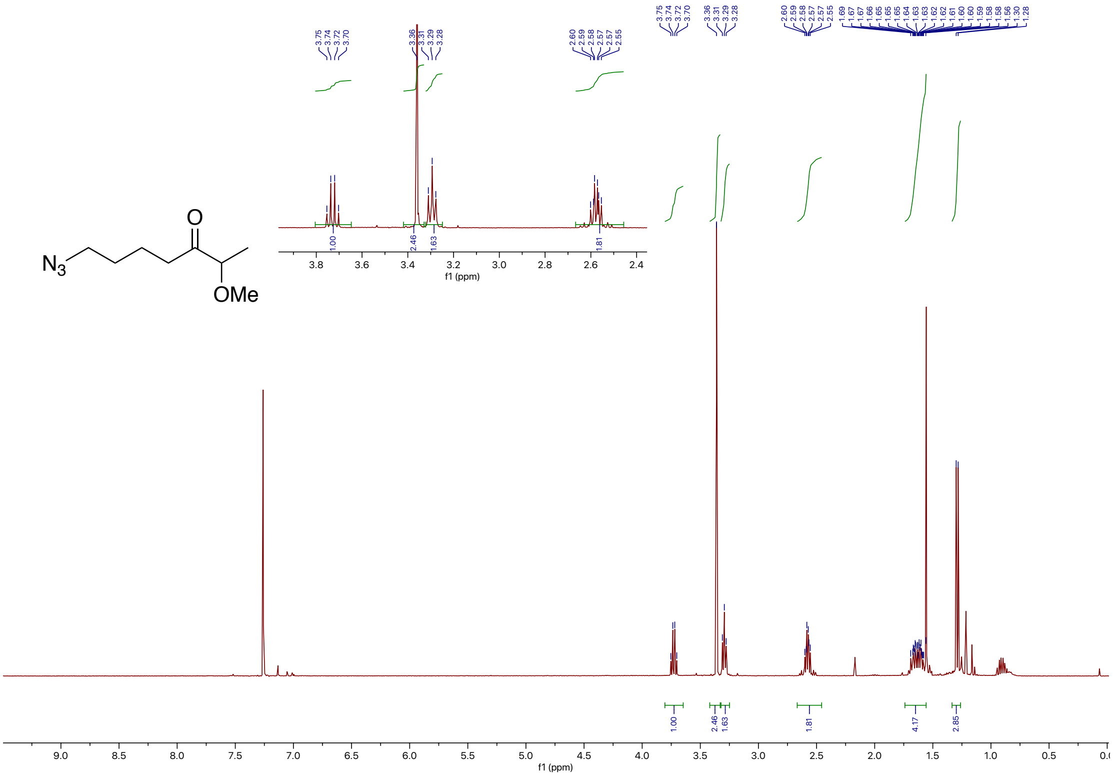

Supplementary Figure 63. ^1H NMR spectra ($CDCl_3$, 400 MHz) of **2m**

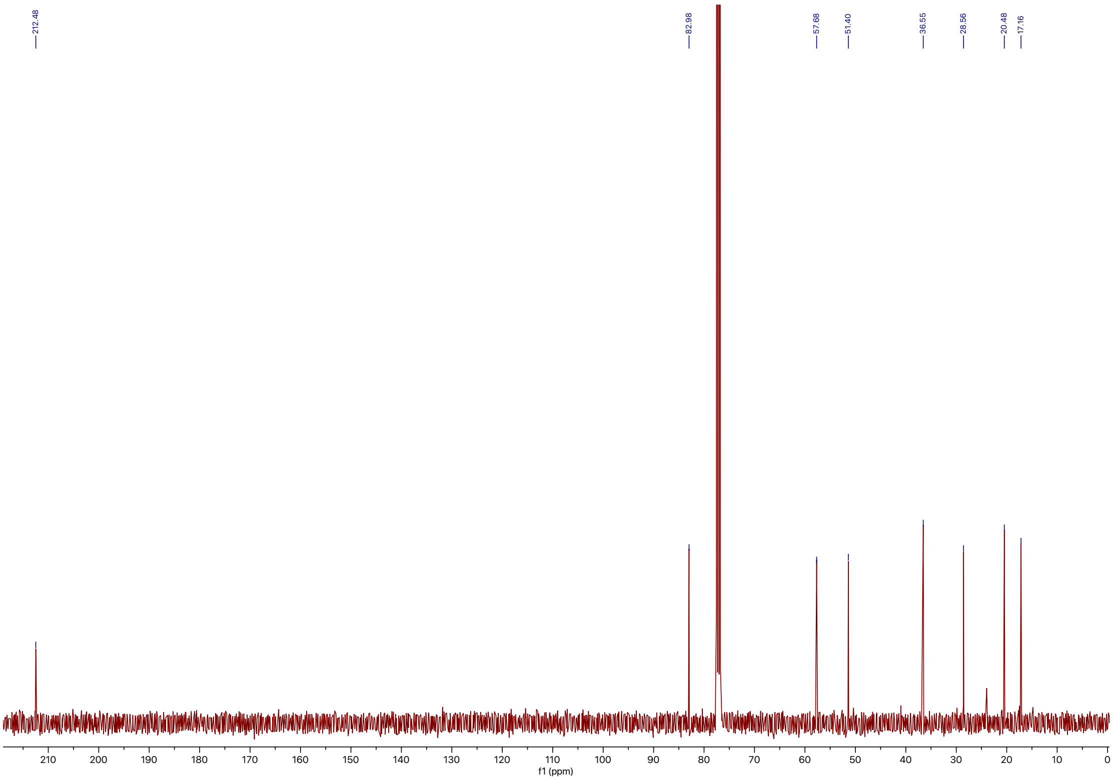

Supplementary Figure 64. ^{13}C NMR spectra ($CDCl_3$, 100 MHz) of **2m**

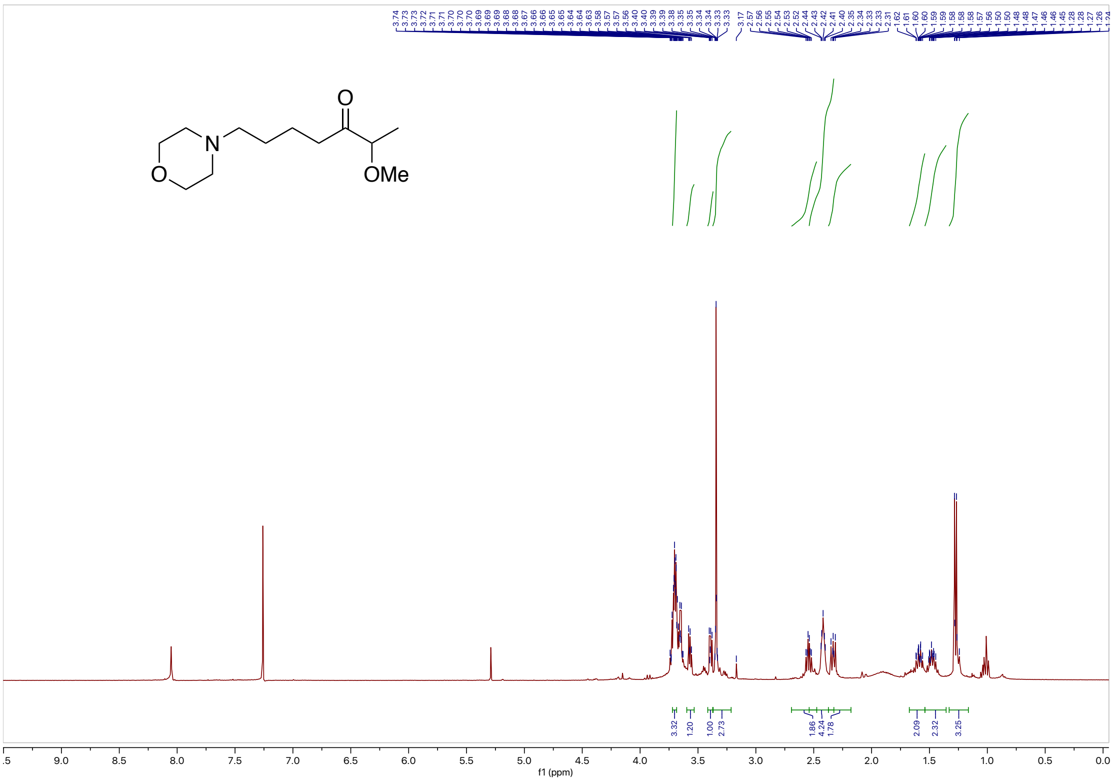

Supplementary Figure 65. ^{1}H NMR spectra ($CDCl_3$, 400 MHz) of **2n**. The compound could not be completely separated from some DMF traces. For this reason, DMF signals can be spotted in NMR.

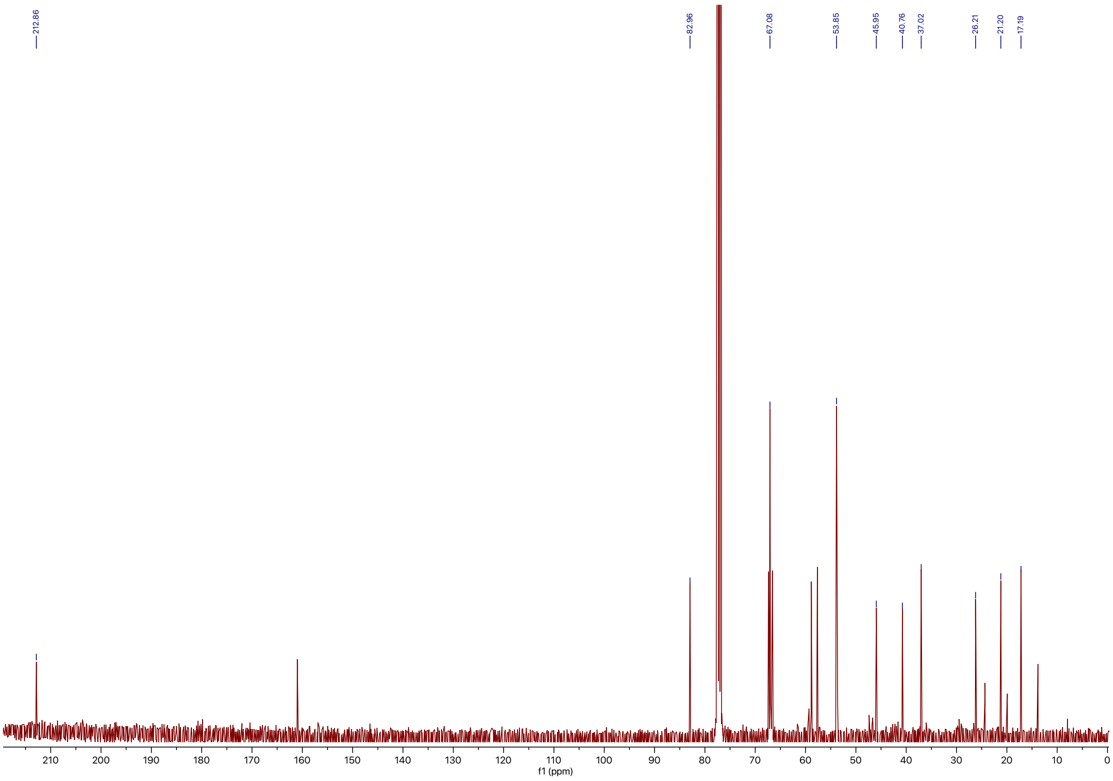

Supplementary Figure 66. ^{13}C NMR spectra ($CDCl_3$, 100 MHz) of **2n**. The compound could not be completely separated from some DMF traces. For this reason, DMF signals can be spotted in NMR.

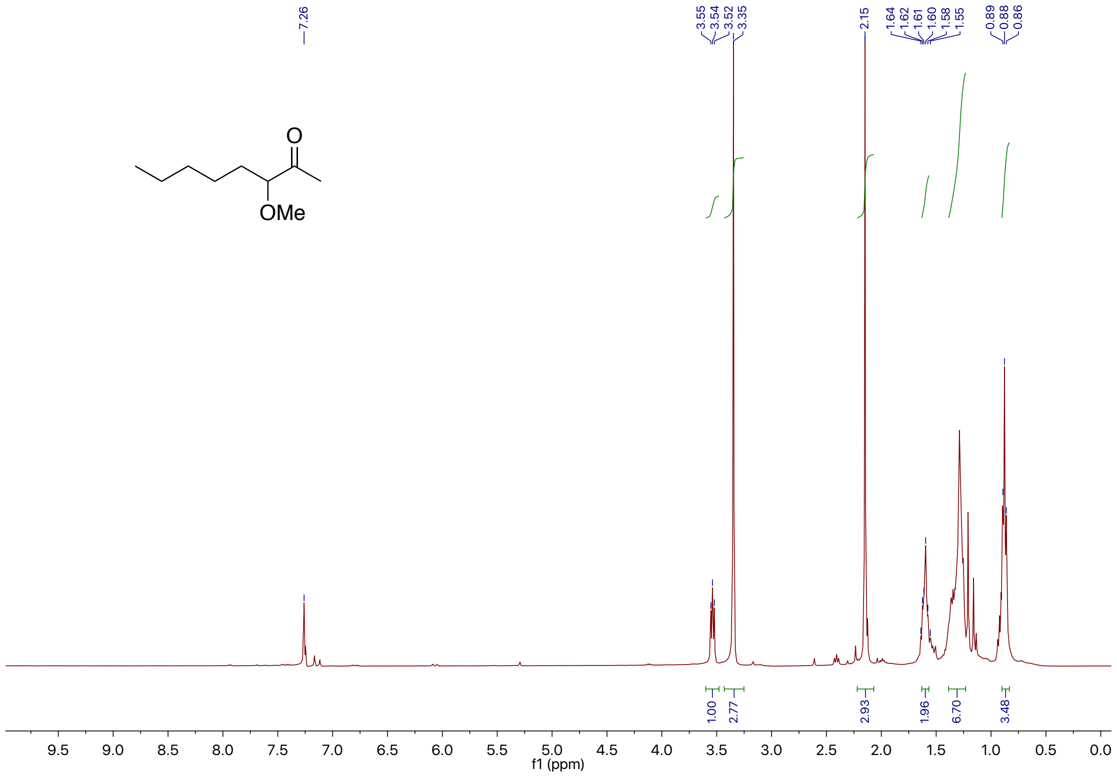

Supplementary Figure 67. ^{1}H NMR spectra ($CDCl_3$, 400 MHz) of **2o**

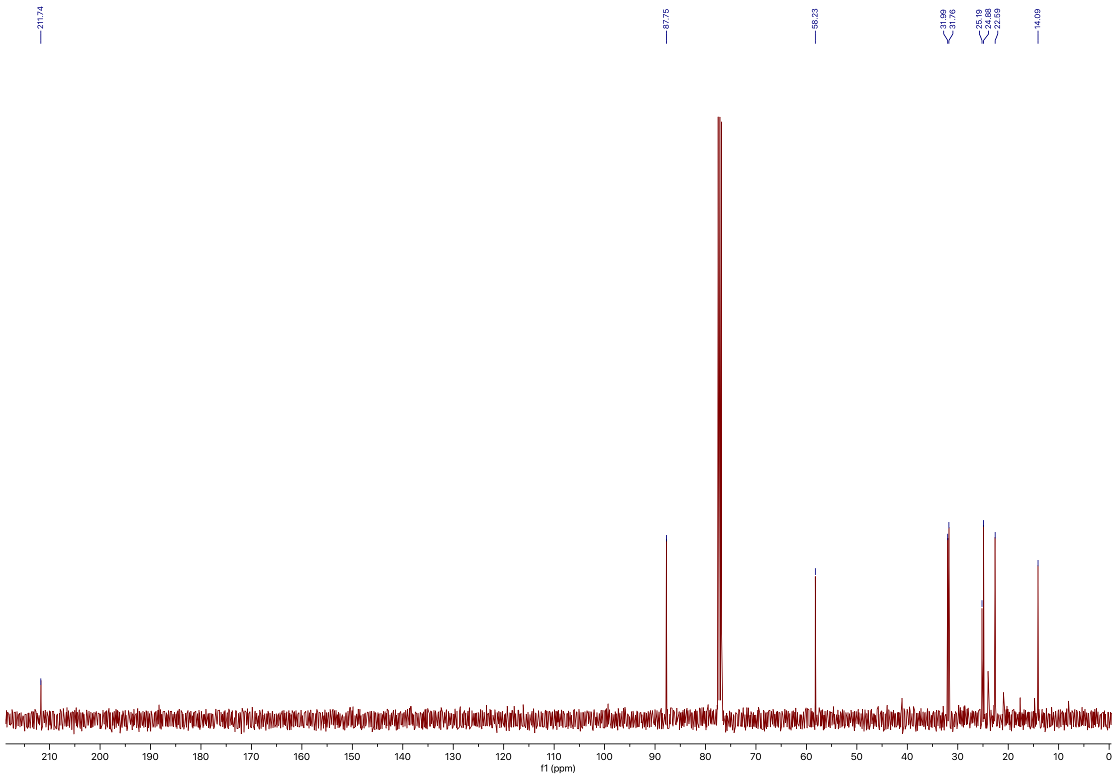

Supplementary Figure 68. ^{13}C NMR spectra ($CDCl_3$, 100 MHz) of **2o**

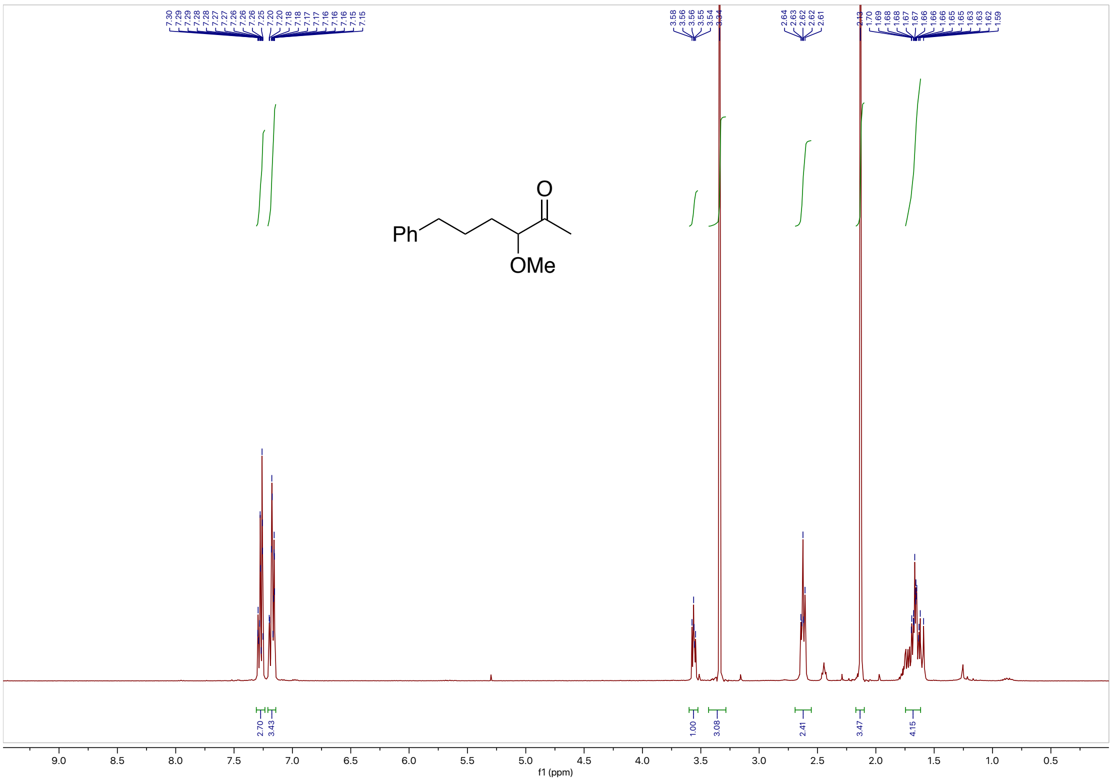

Supplementary Figure 69. ^{1}H NMR spectra ($CDCl_3$, 400 MHz) of **2p**

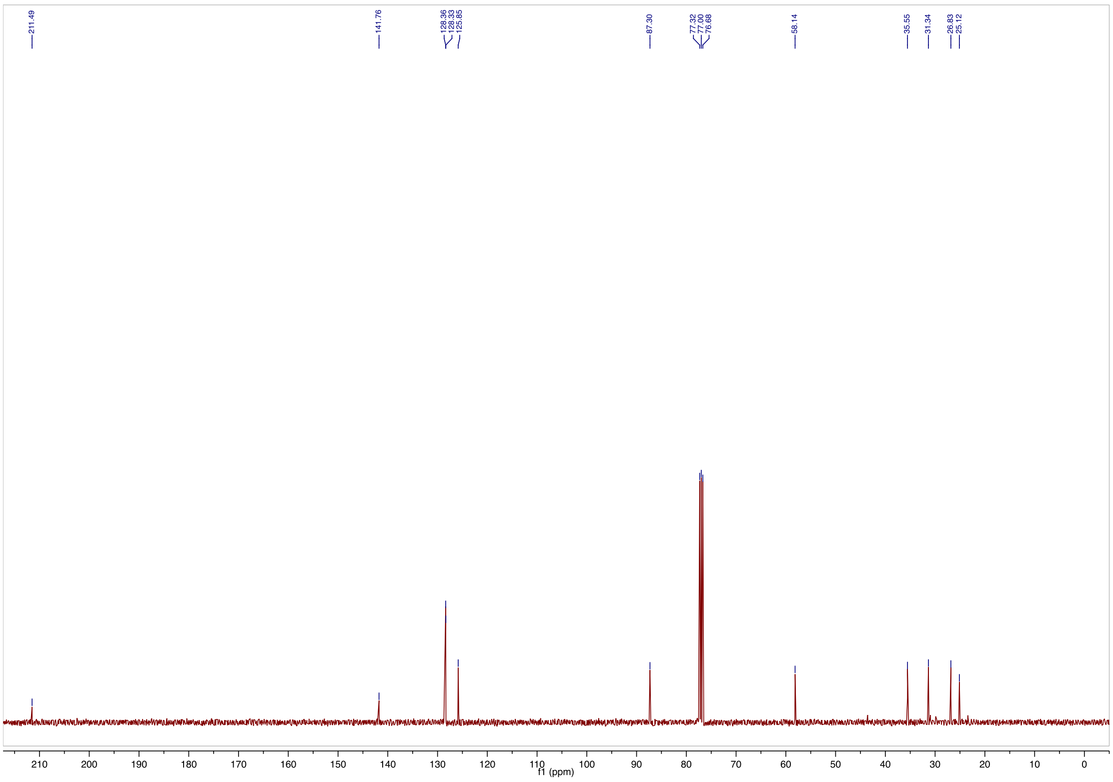

Supplementary Figure 70. ^{13}C NMR spectra ($CDCl_3$, 100 MHz) of **2p**

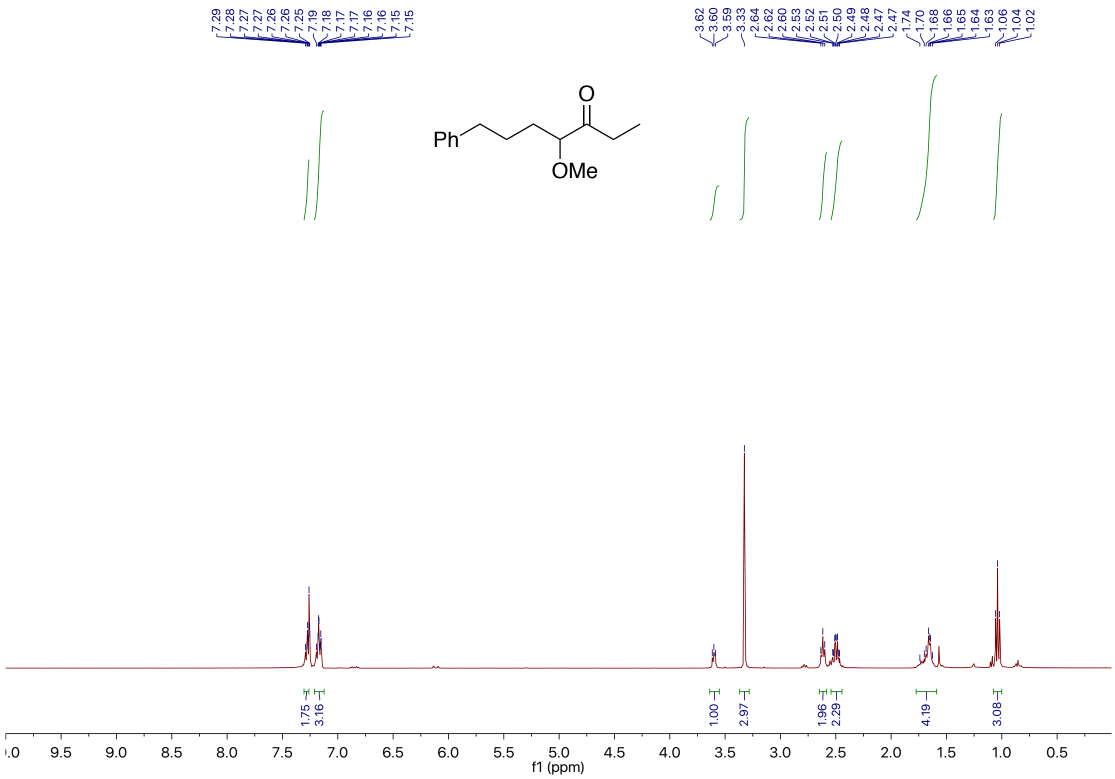

Supplementary Figure 71. ^{1}H NMR spectra ($CDCl_3$, 400 MHz) of **2q**

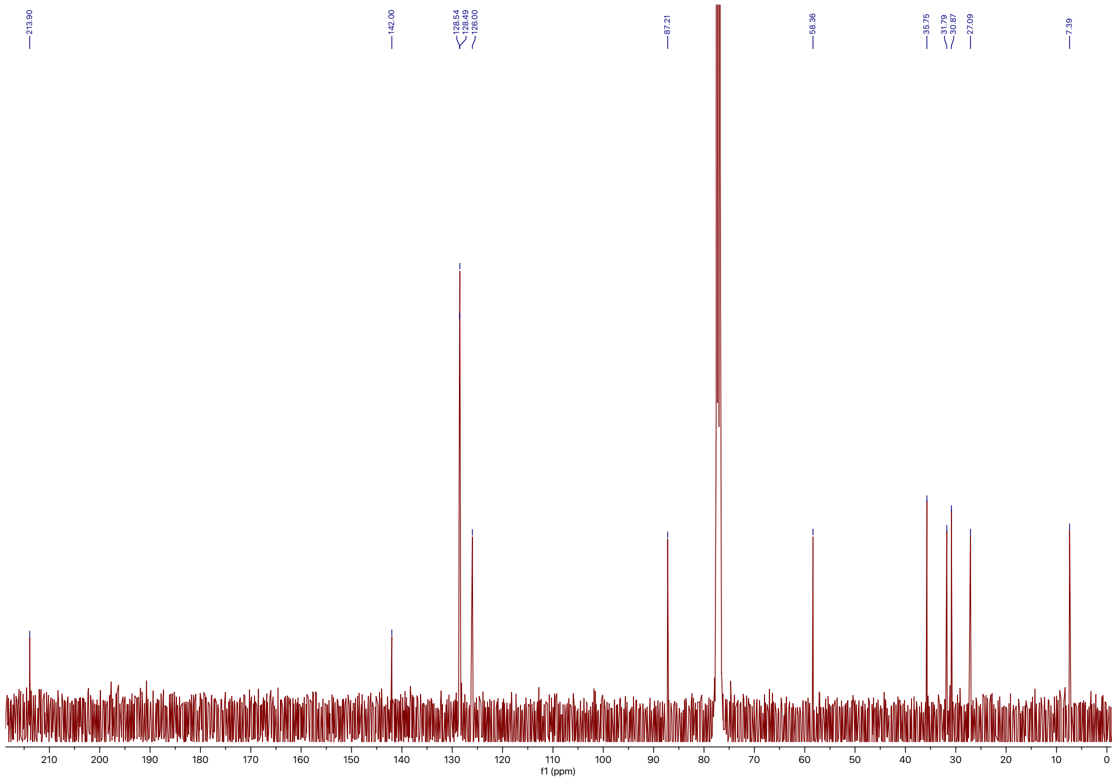

Supplementary Figure 72. ^{13}C NMR spectra ($CDCl_3$, 100 MHz) of **2q**

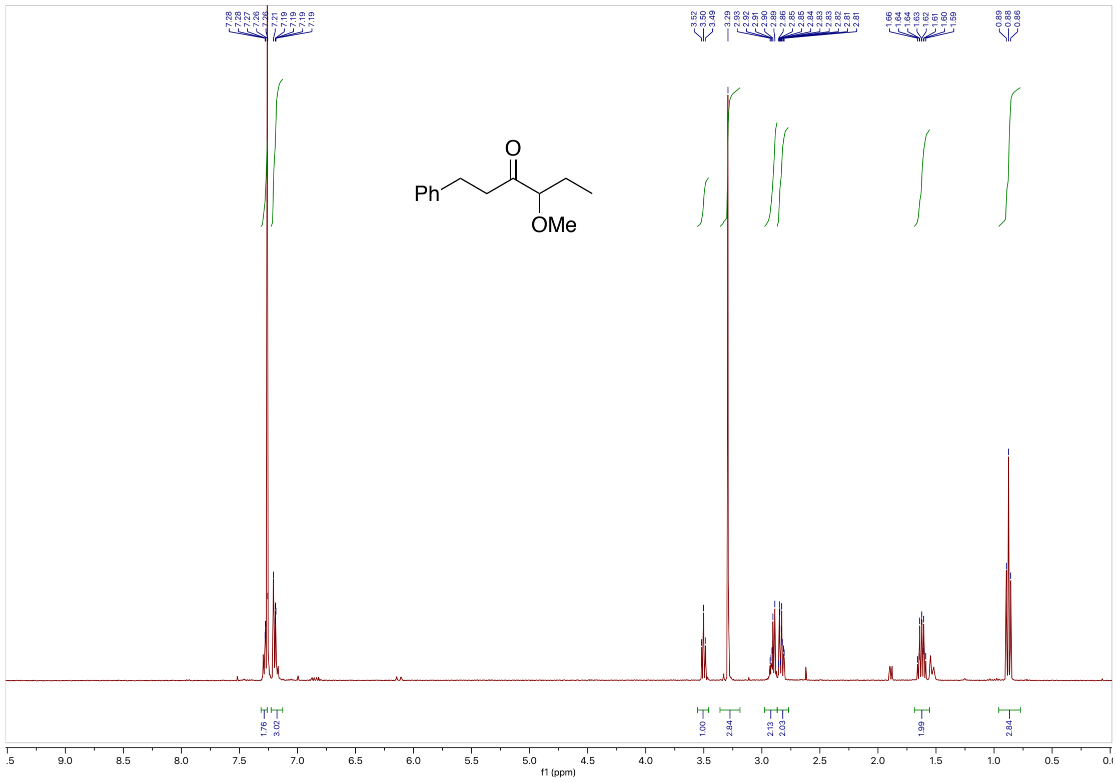

Supplementary Figure 73. ^{1}H NMR spectra ($CDCl_3$, 400 MHz) of **2r**

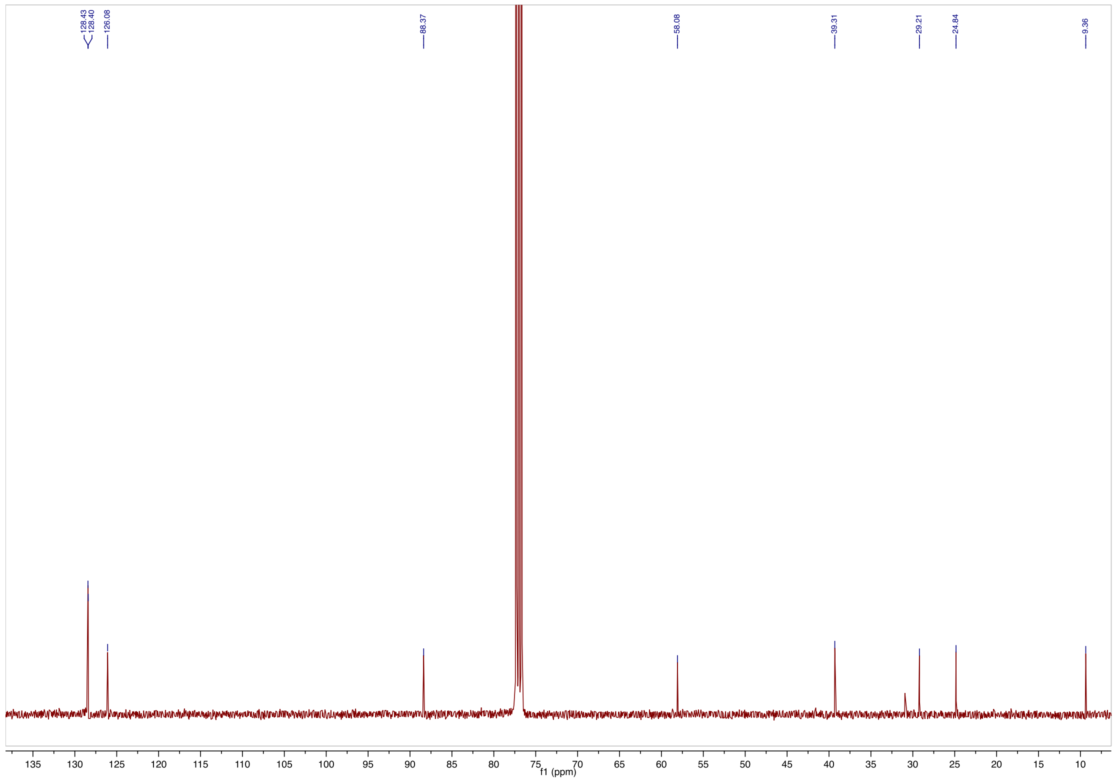

Supplementary Figure 74. ^{13}C NMR spectra ($CDCl_3$, 100 MHz) of **2r**

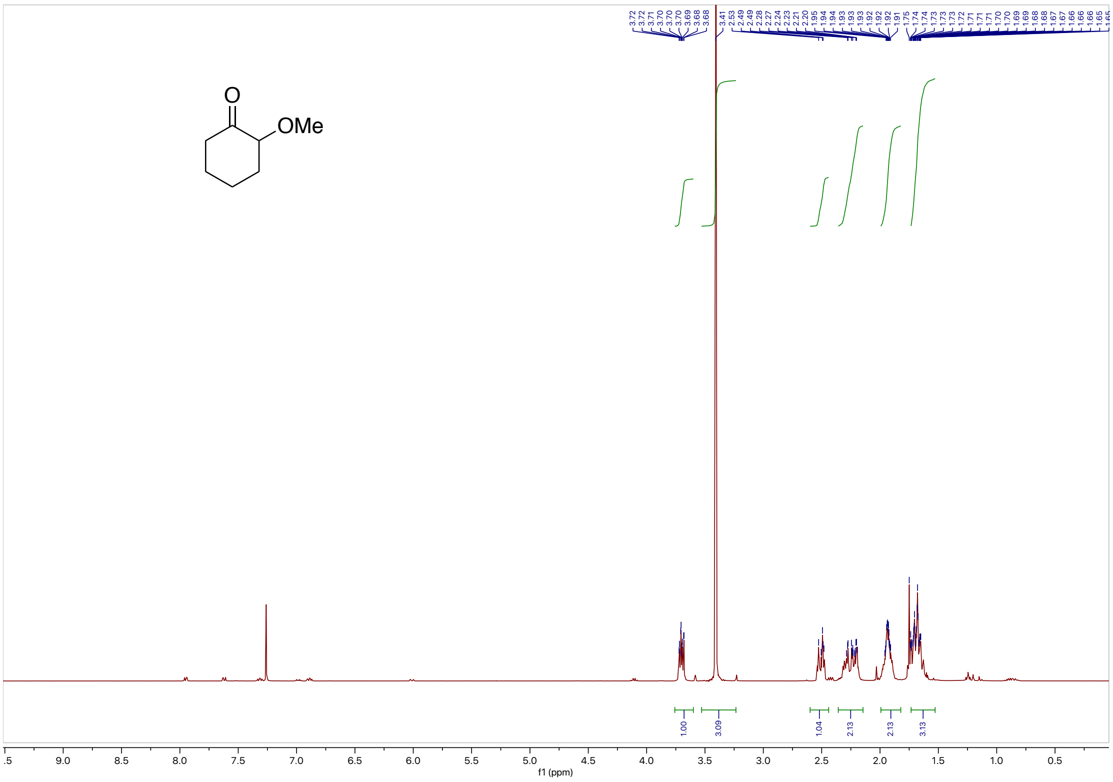

Supplementary Figure 75. ^{1}H NMR spectra ($CDCl_3$, 400 MHz) of **2s**

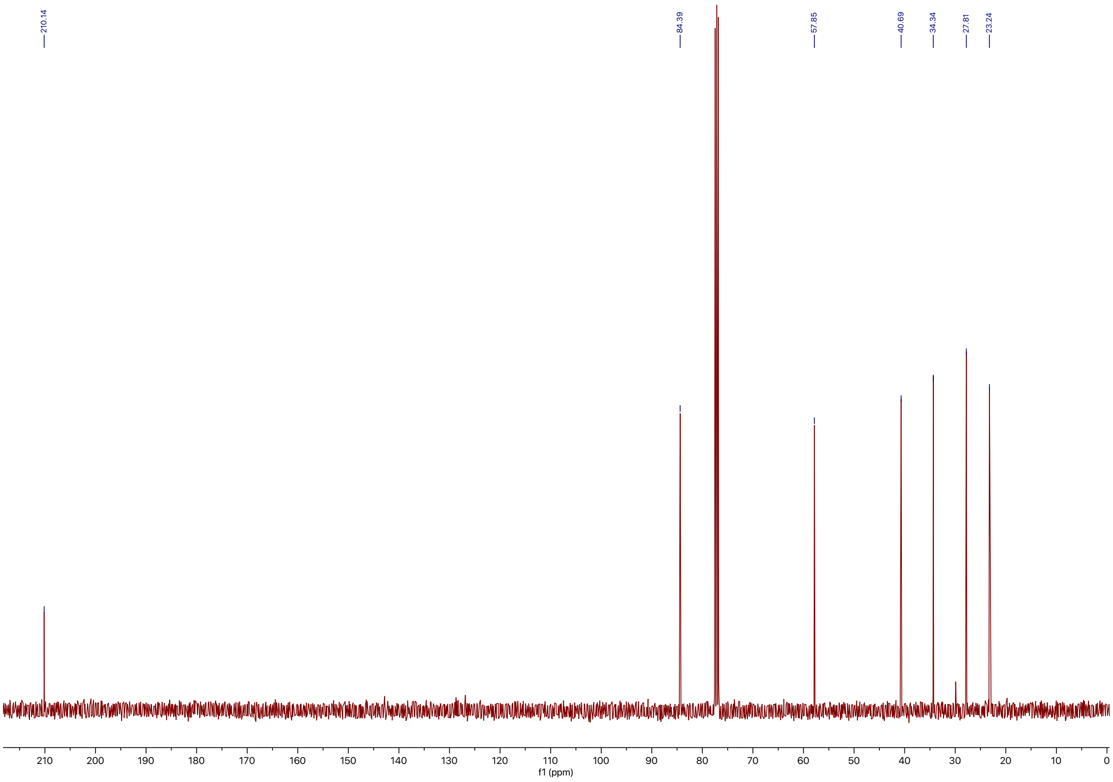

Supplementary Figure 76. ^{13}C NMR spectra ($CDCl_3$, 100 MHz) of **2s**

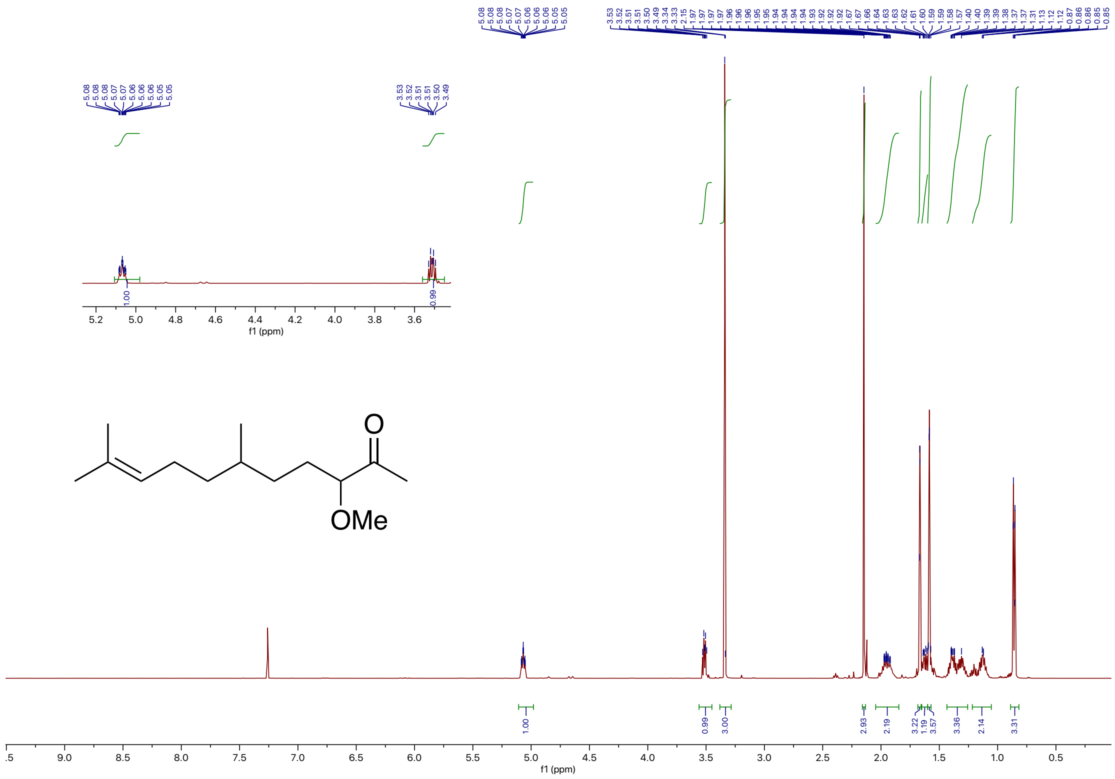

Supplementary Figure 77. ^{1}H NMR spectra ($CDCl_3$, 400 MHz) of **2t**

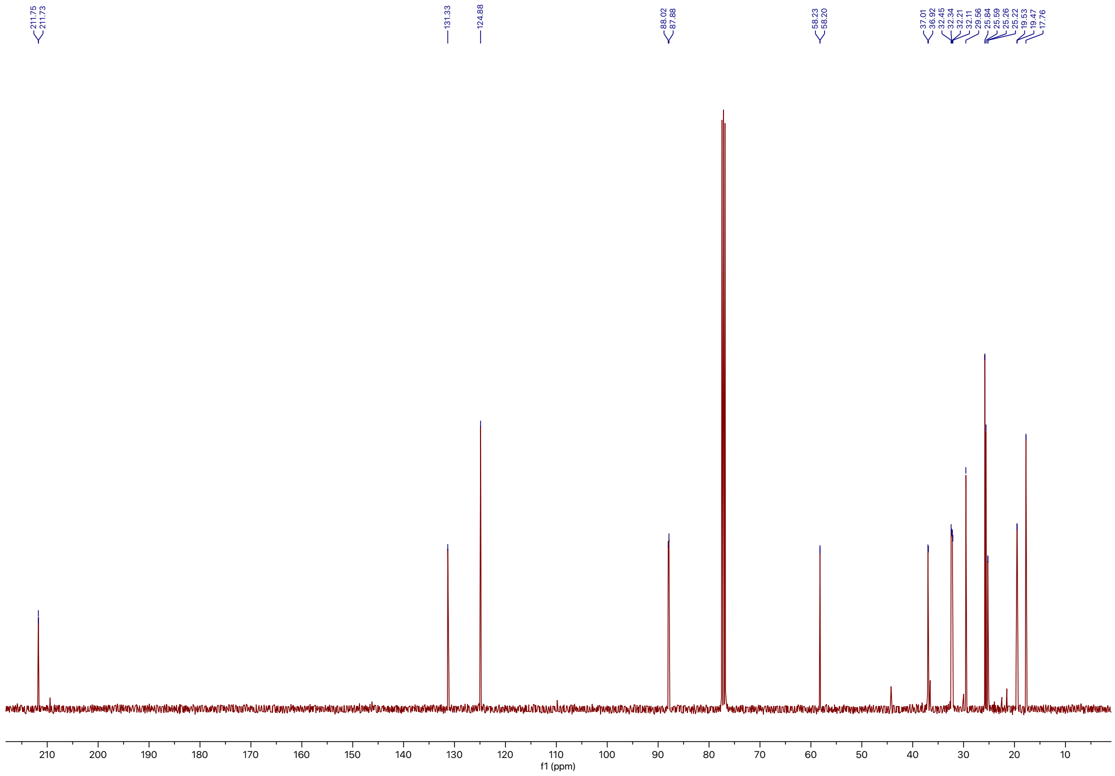

Supplementary Figure 78. ^{13}C NMR spectra ($CDCl_3$, 100 MHz) of **2t**

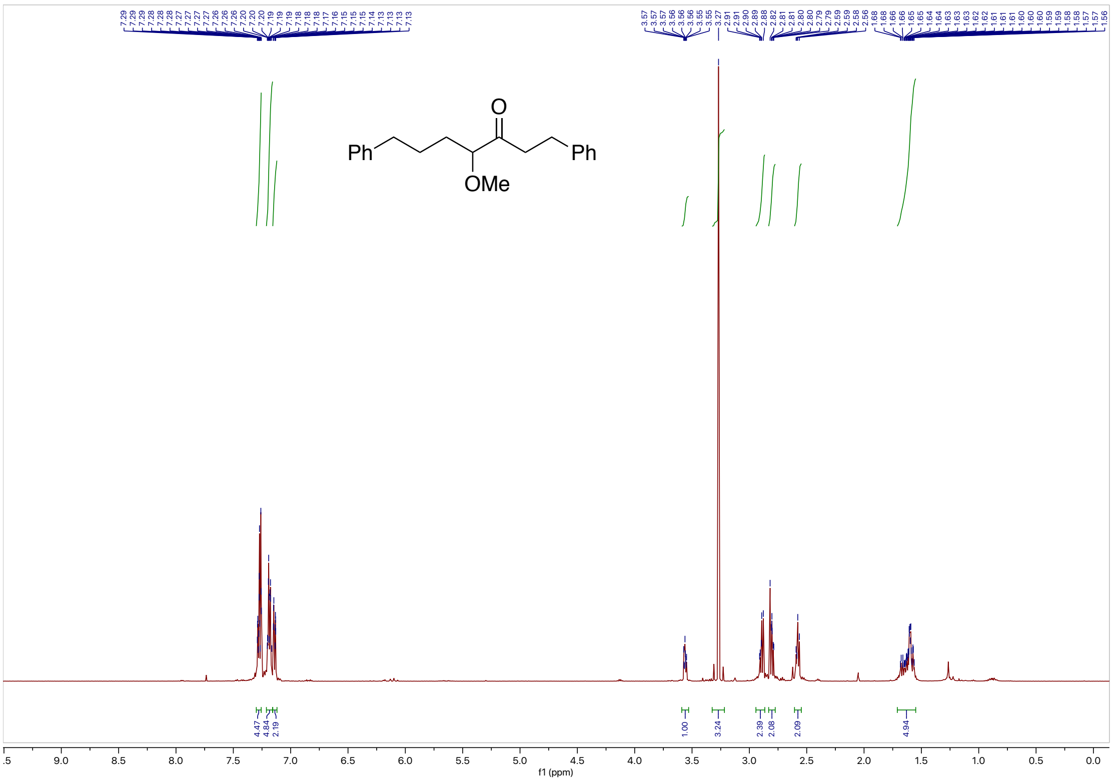

Supplementary Figure 79. ^{1}H NMR spectra ($CDCl_3$, 400 MHz) of **2u**

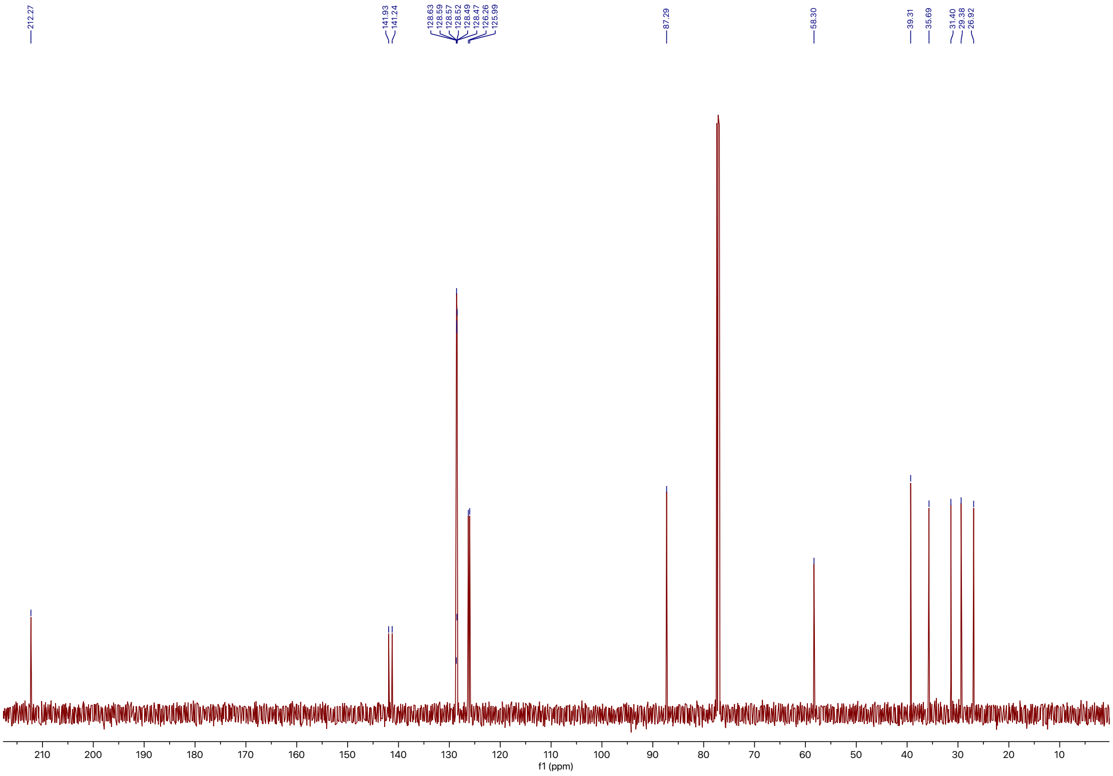

Supplementary Figure 80. ^{13}C NMR spectra ($CDCl_3$, 100 MHz) of **2u**

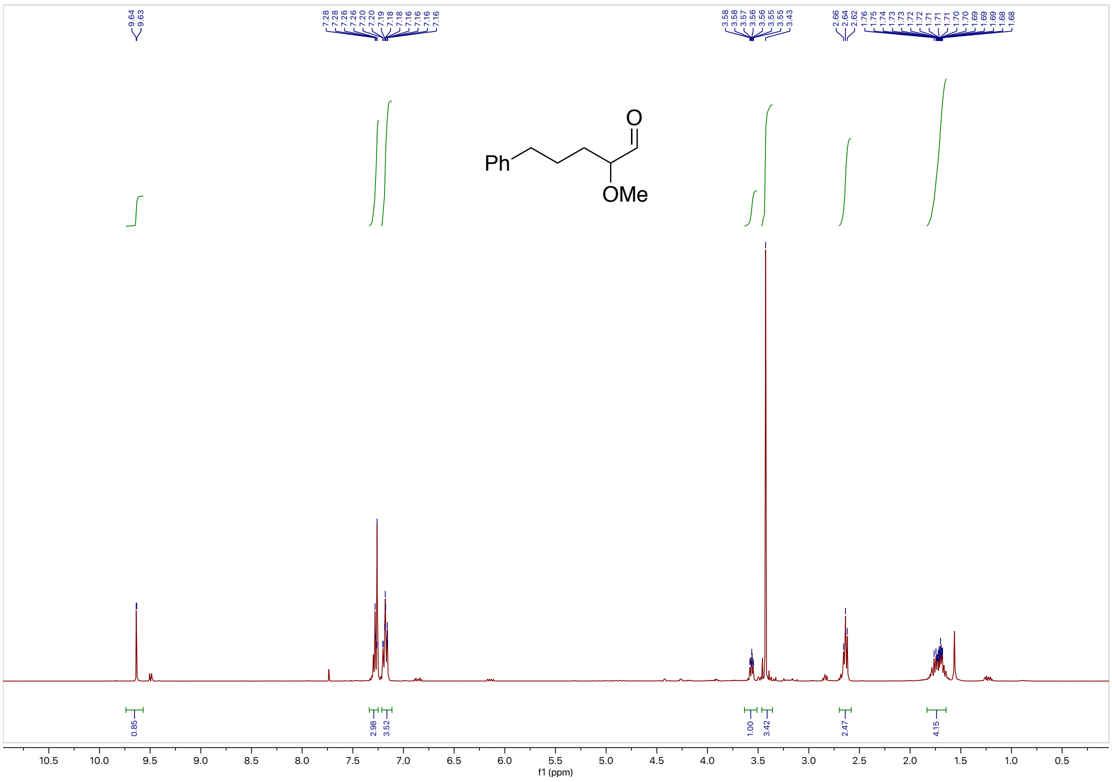

Supplementary Figure 81. ^{1}H NMR spectra ($CDCl_3$, 400 MHz) of **2v**

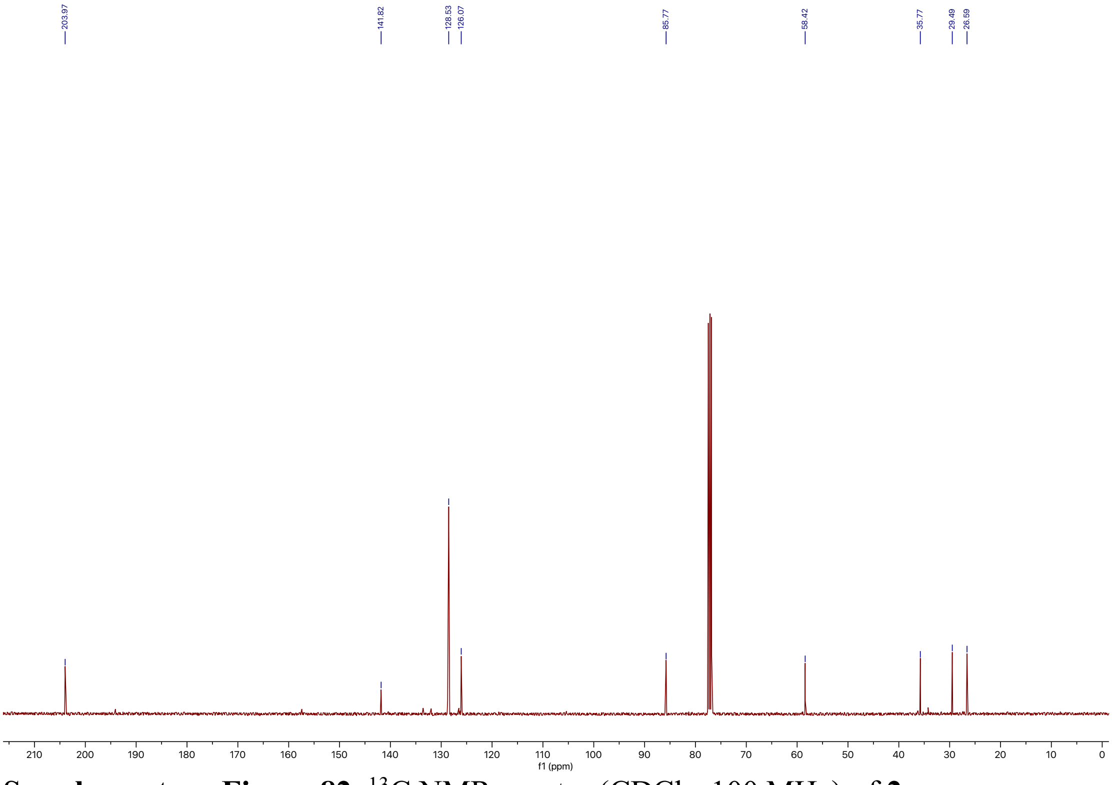

Supplementary Figure 82. ^{13}C NMR spectra ($CDCl_3$, 100 MHz) of **2v**

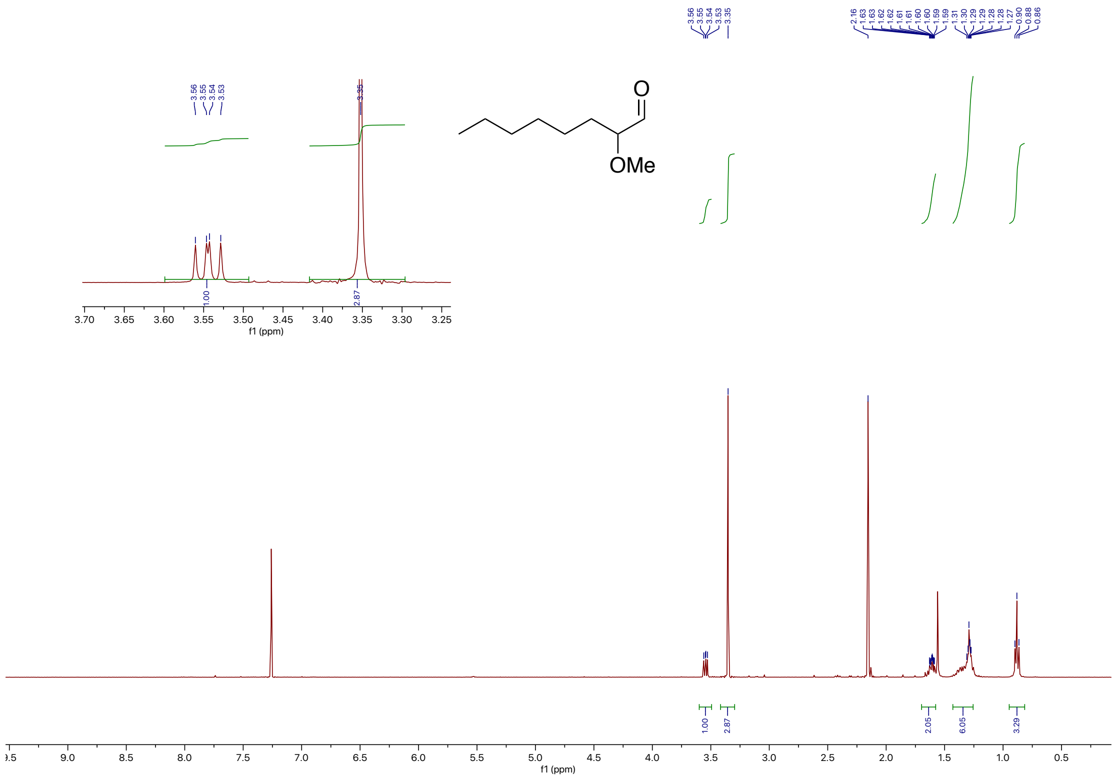

Supplementary Figure 83. ^{1}H NMR spectra ($CDCl_3$, 400 MHz) of **2w**. The compound is volatile and it cannot be kept for long under vacuum. For this reason, some solvents can be spotted in ^{1}H NMR.

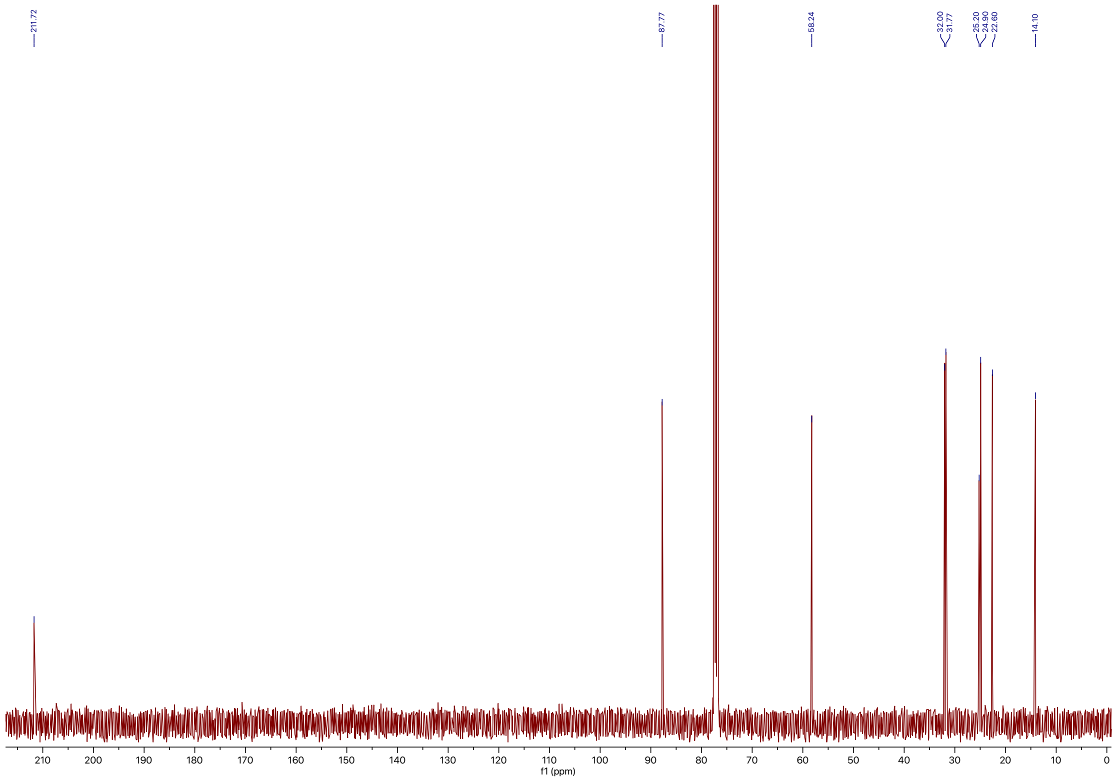

Supplementary Figure 84. ^{13}C NMR spectra ($CDCl_3$, 100 MHz) of **2w**

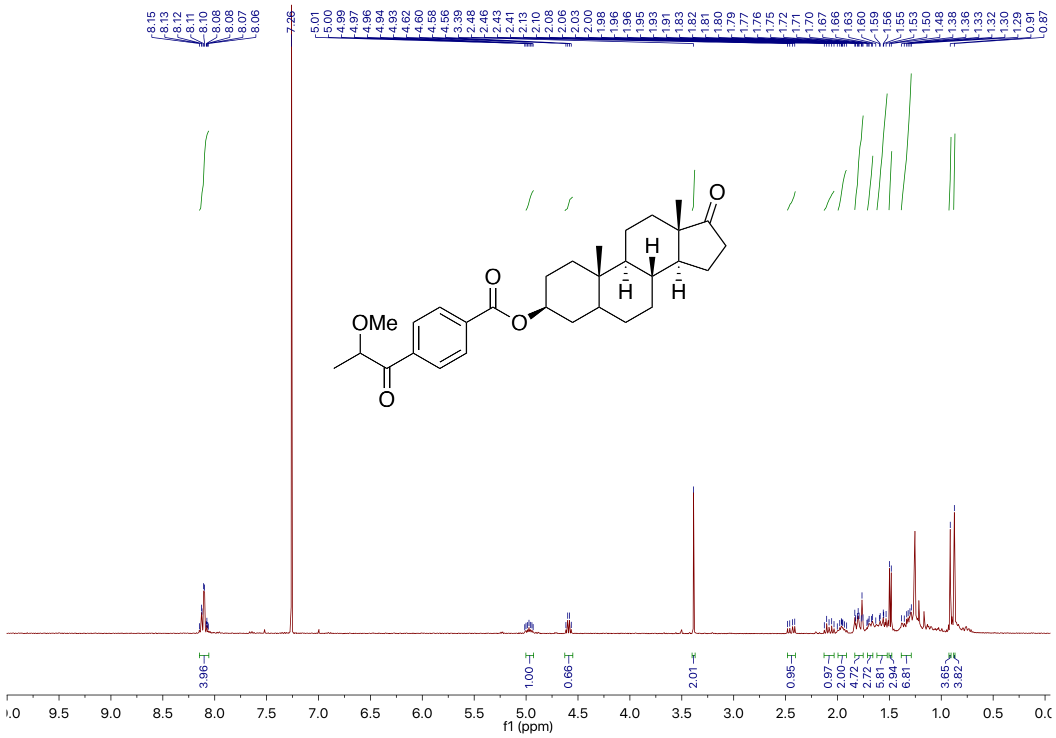

Supplementary Figure 85. ^{1}H NMR spectra ($CDCl_3$, 400 MHz) of **2x**

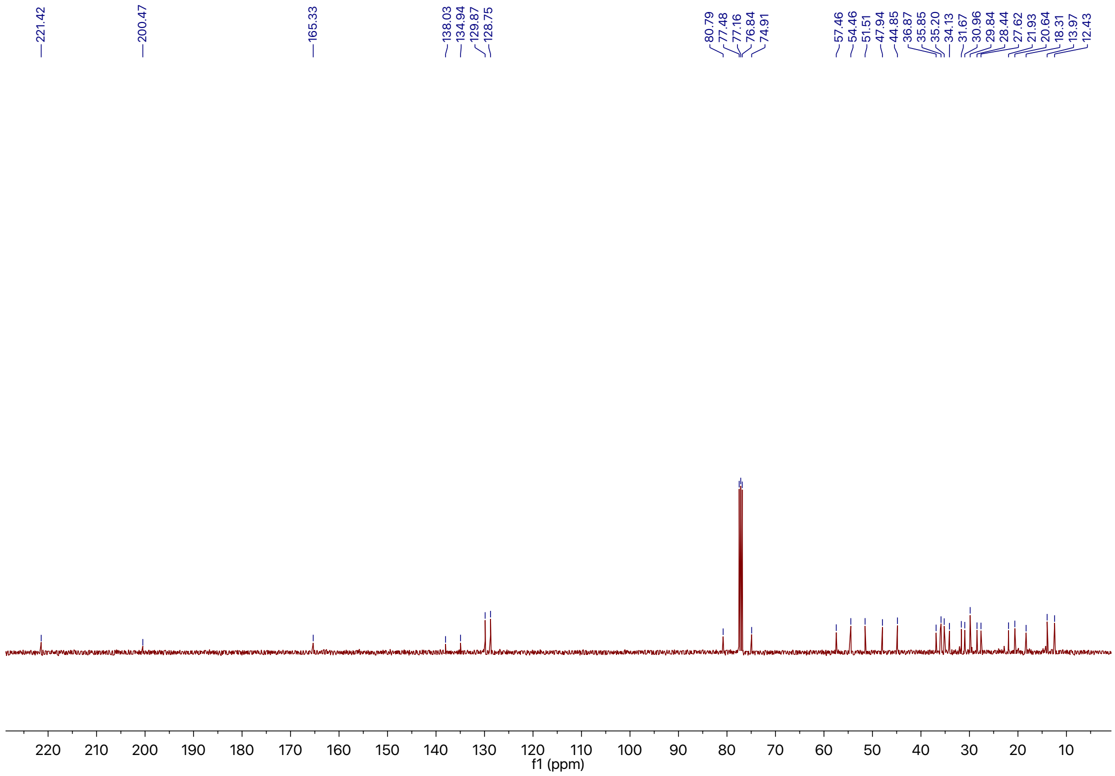

Supplementary Figure 86. ^{13}C NMR spectra ($CDCl_3$, 100 MHz) of **2x**

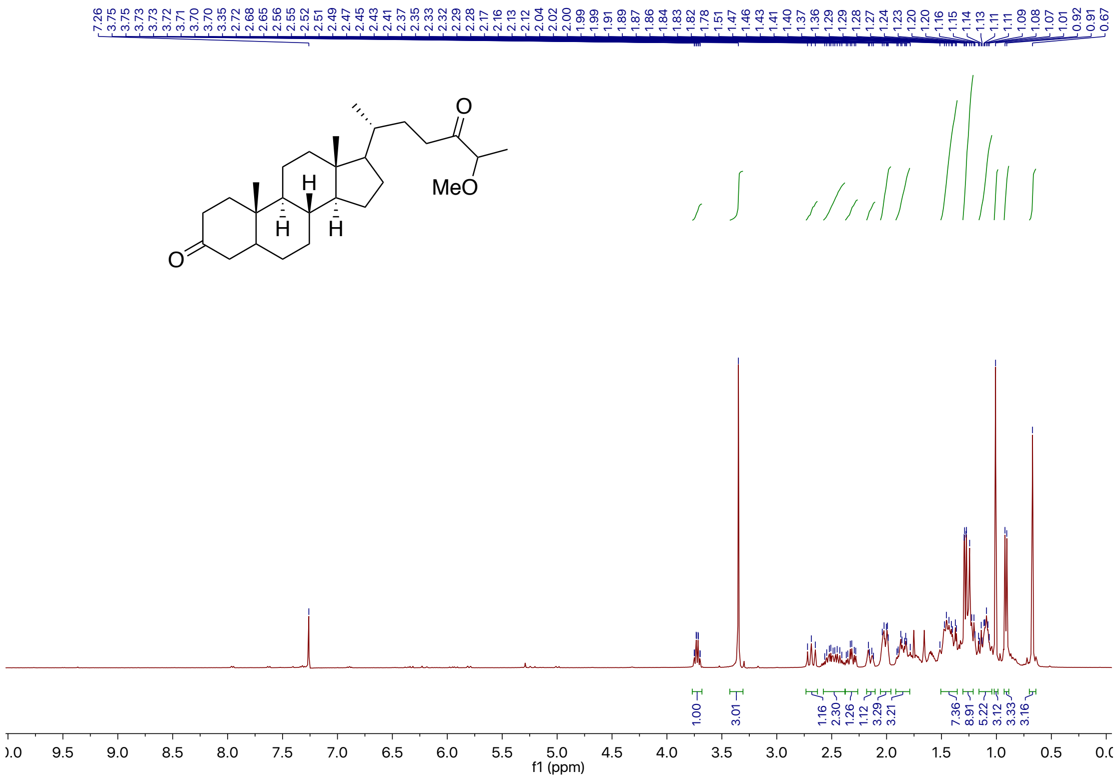

Supplementary Figure 87. ^{1}H NMR spectra ($CDCl_3$, 400 MHz) of **2y**

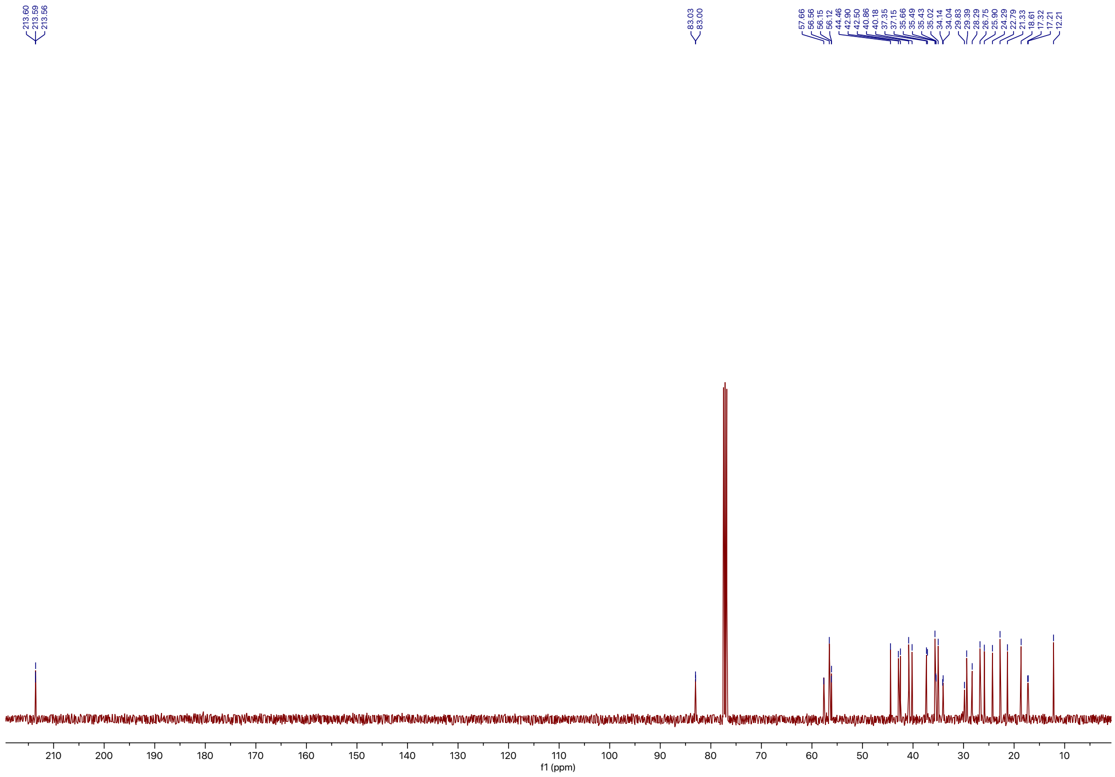

Supplementary Figure 88. ^{13}C NMR spectra ($CDCl_3$, 100 MHz) of **2y**

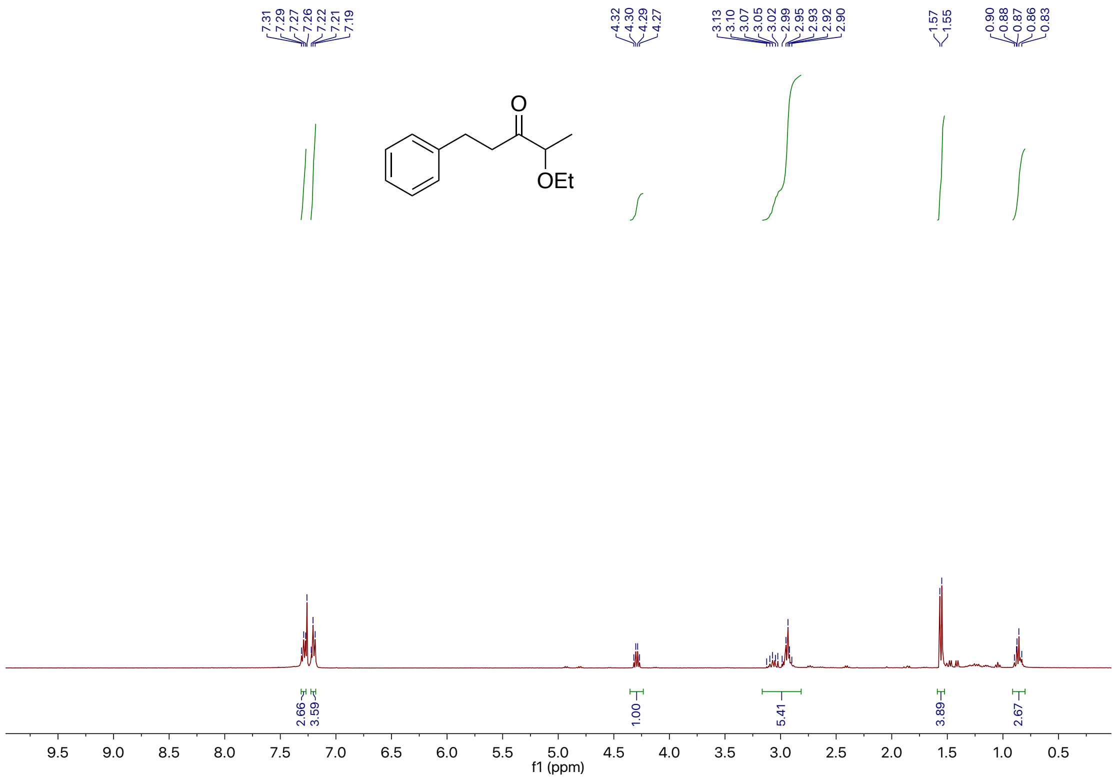

Supplementary Figure 89. ^{1}H NMR spectra ($CDCl_3$, 400 MHz) of **6a**

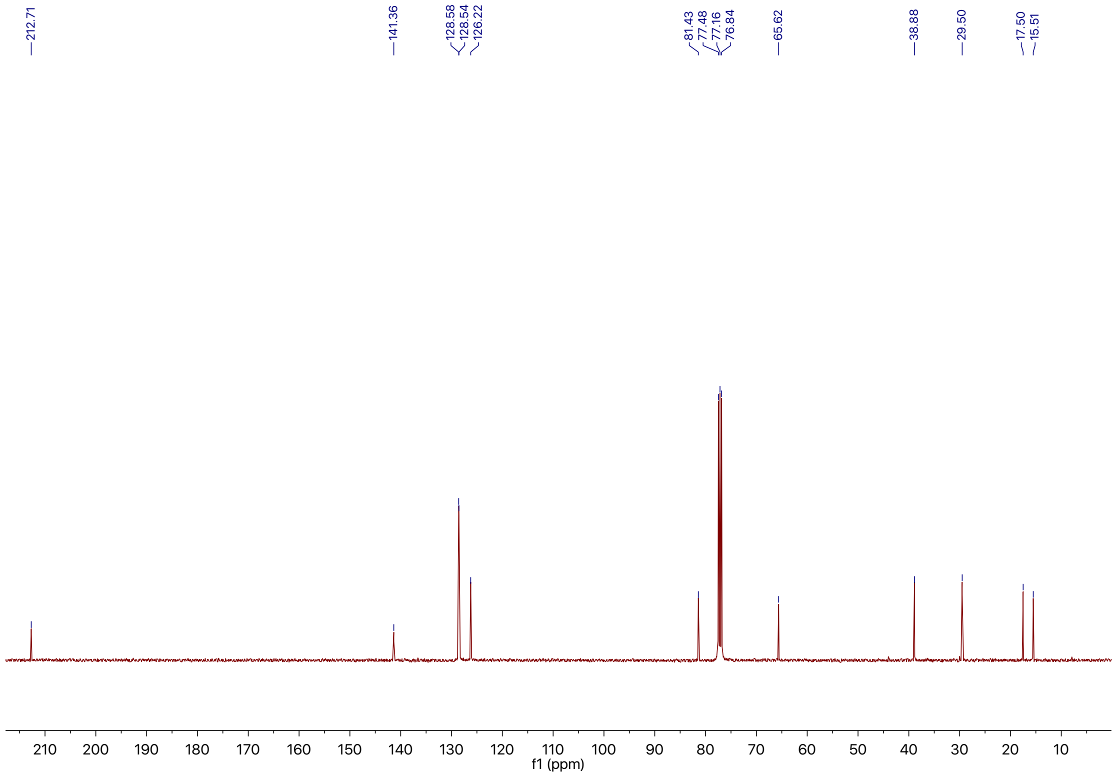

Supplementary Figure 90. ^{13}C NMR spectra ($CDCl_3$, 100 MHz) of **6a**

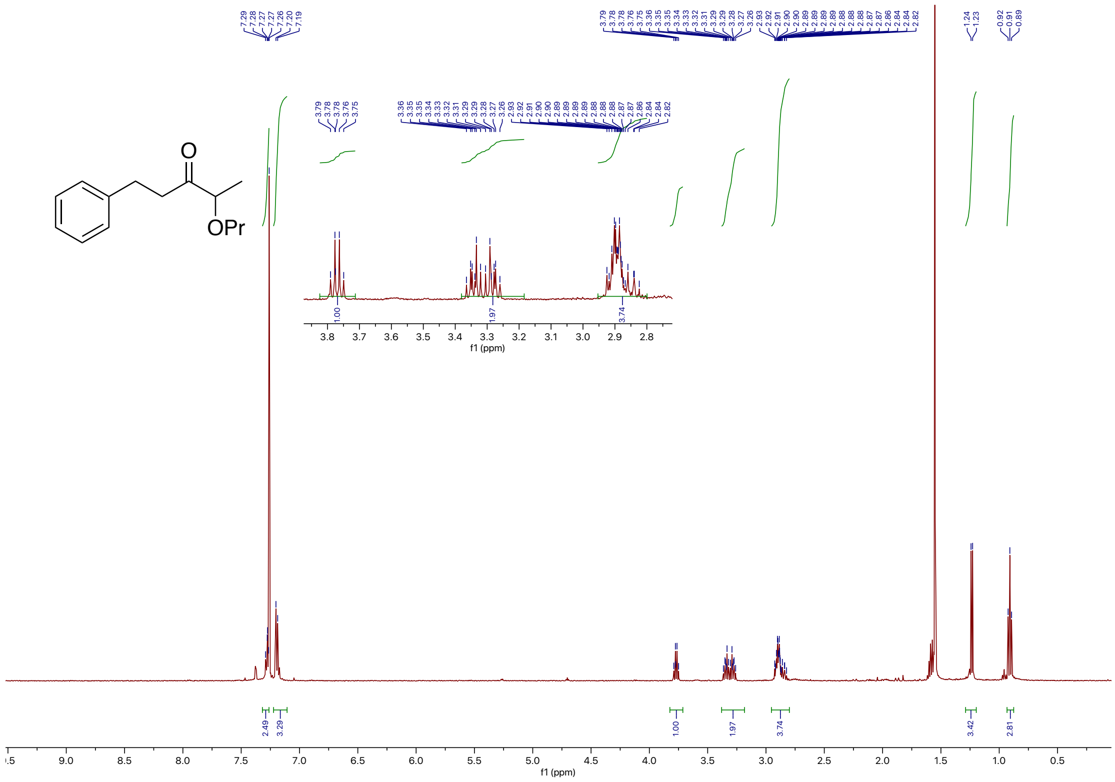

Supplementary Figure 91. ^{1}H NMR spectra ($CDCl_3$, 400 MHz) of **7a**

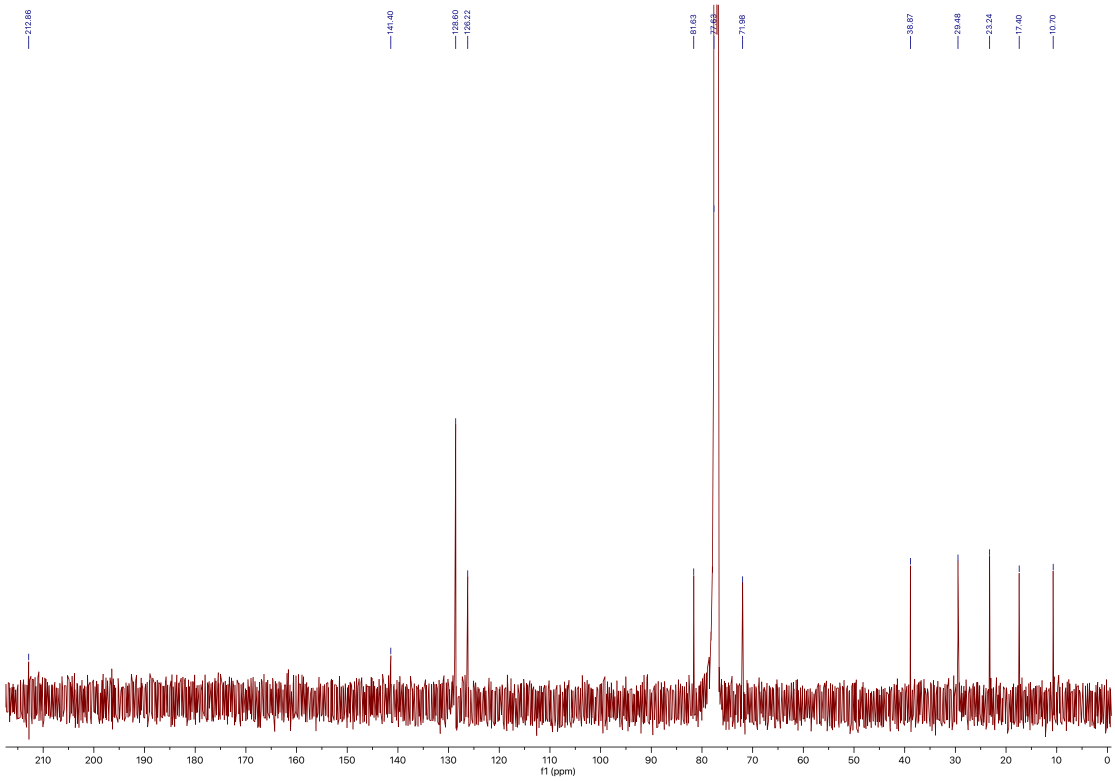

Supplementary Figure 92. ^{13}C NMR spectra ($CDCl_3$, 100 MHz) of **7a**

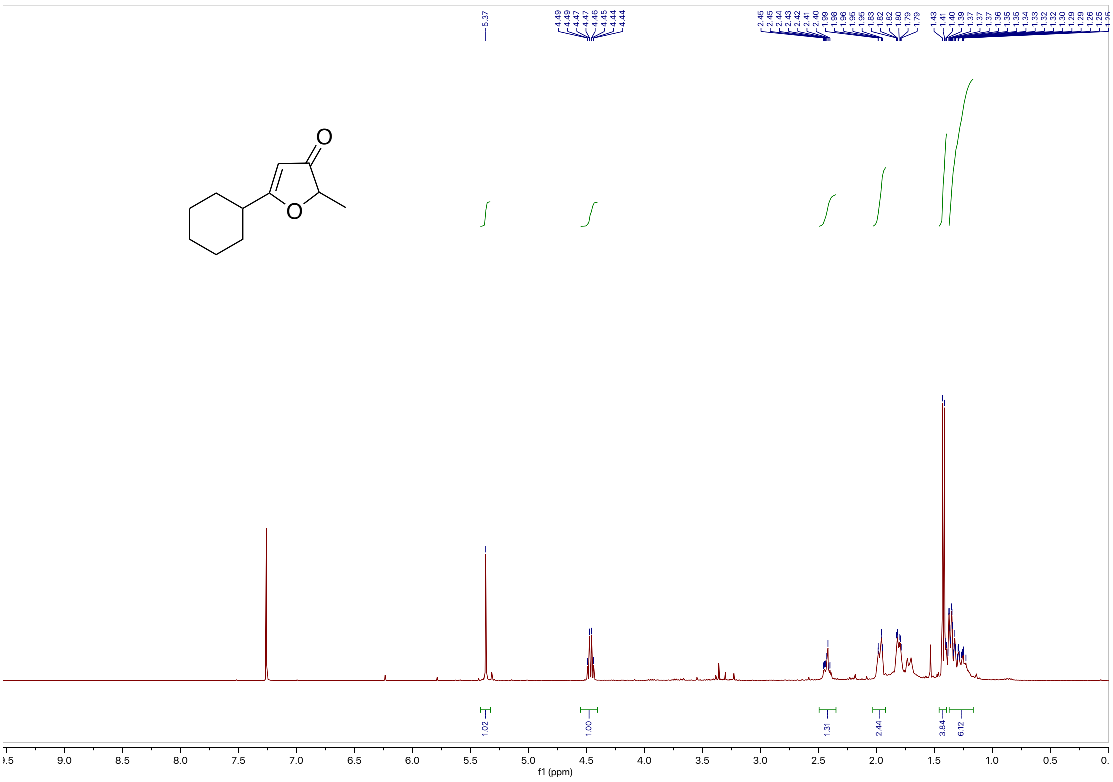

Supplementary Figure 93. ^{1}H NMR spectra ($CDCl_3$, 400 MHz) of **9a**

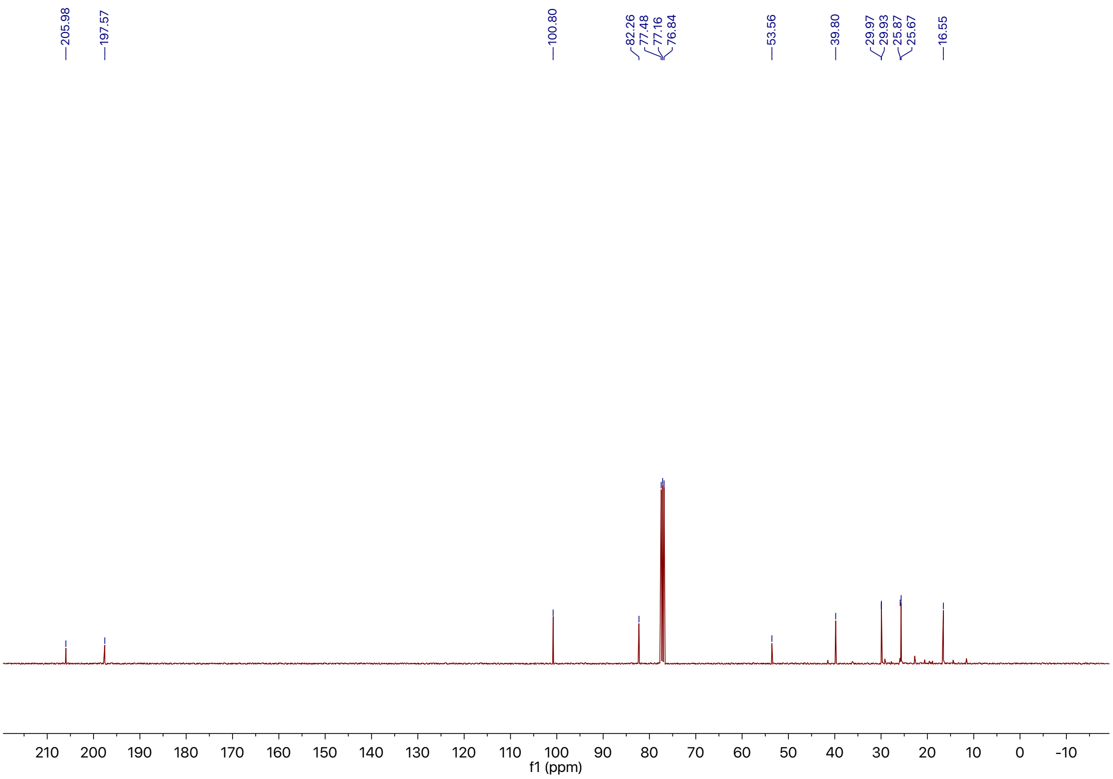

Supplementary Figure 94. ^{13}C NMR spectra ($CDCl_3$, 100 MHz) of **9a**

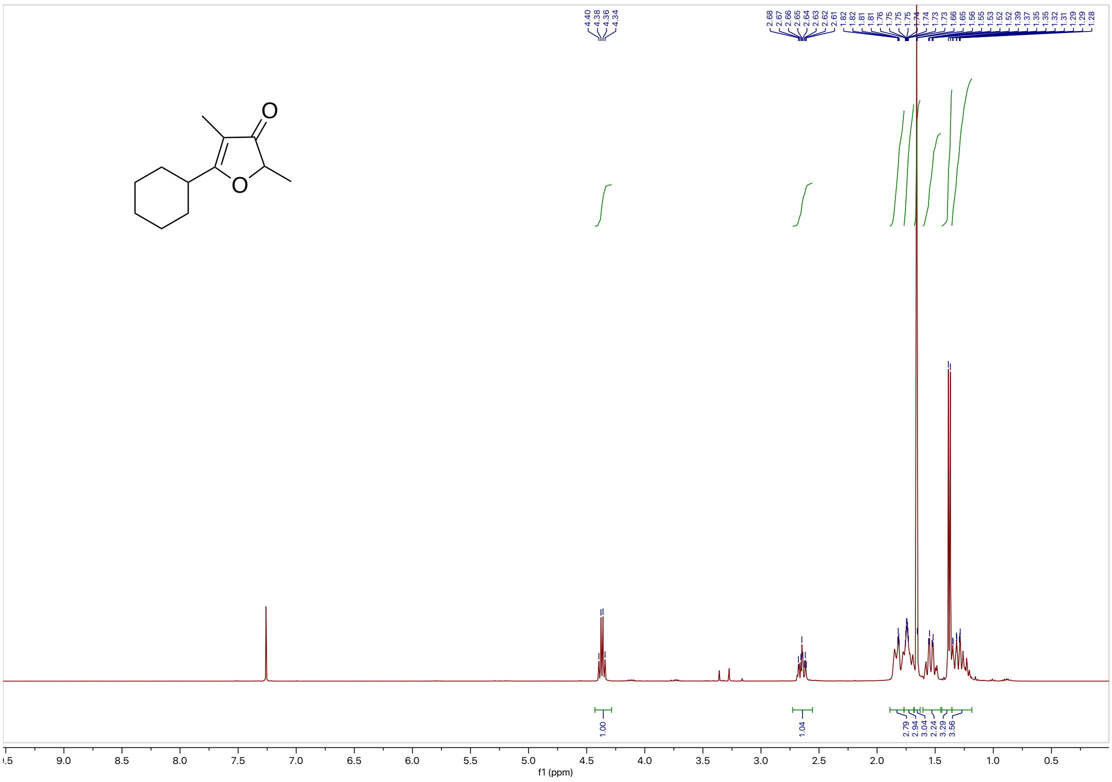

Supplementary Figure 95. ^{1}H NMR spectra ($CDCl_3$, 400 MHz) of **9b**

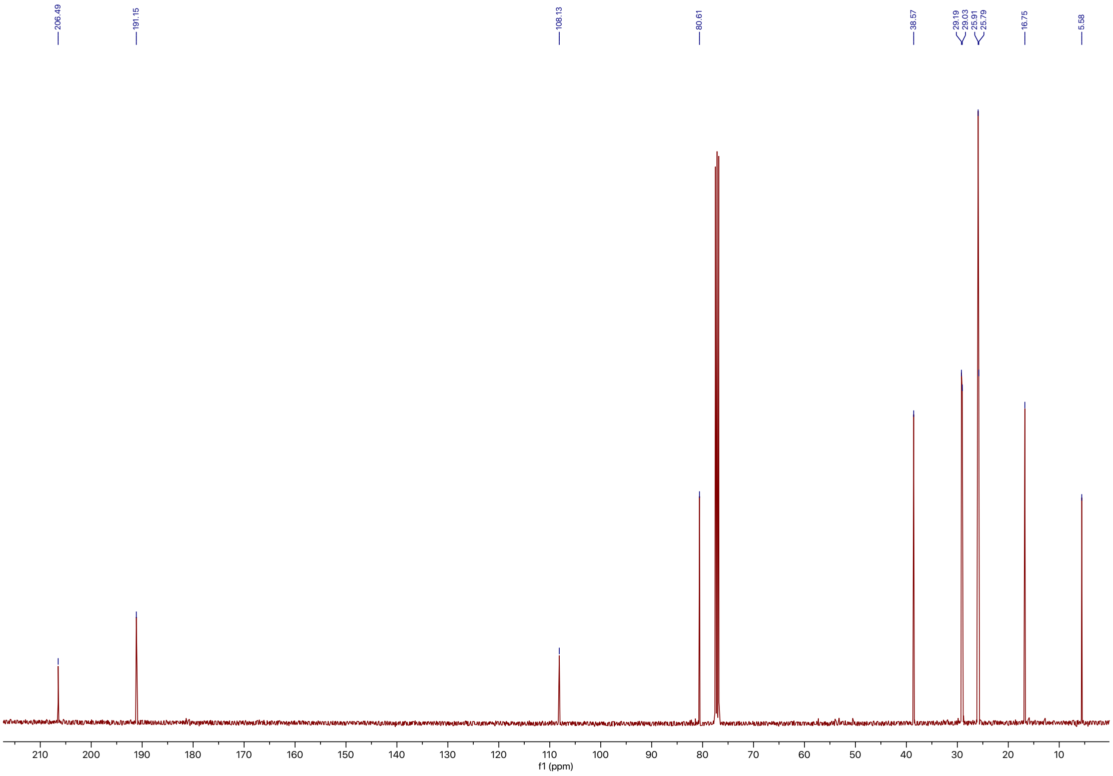

Supplementary Figure 96. ^{13}C NMR spectra ($CDCl_3$, 100 MHz) of **9b**

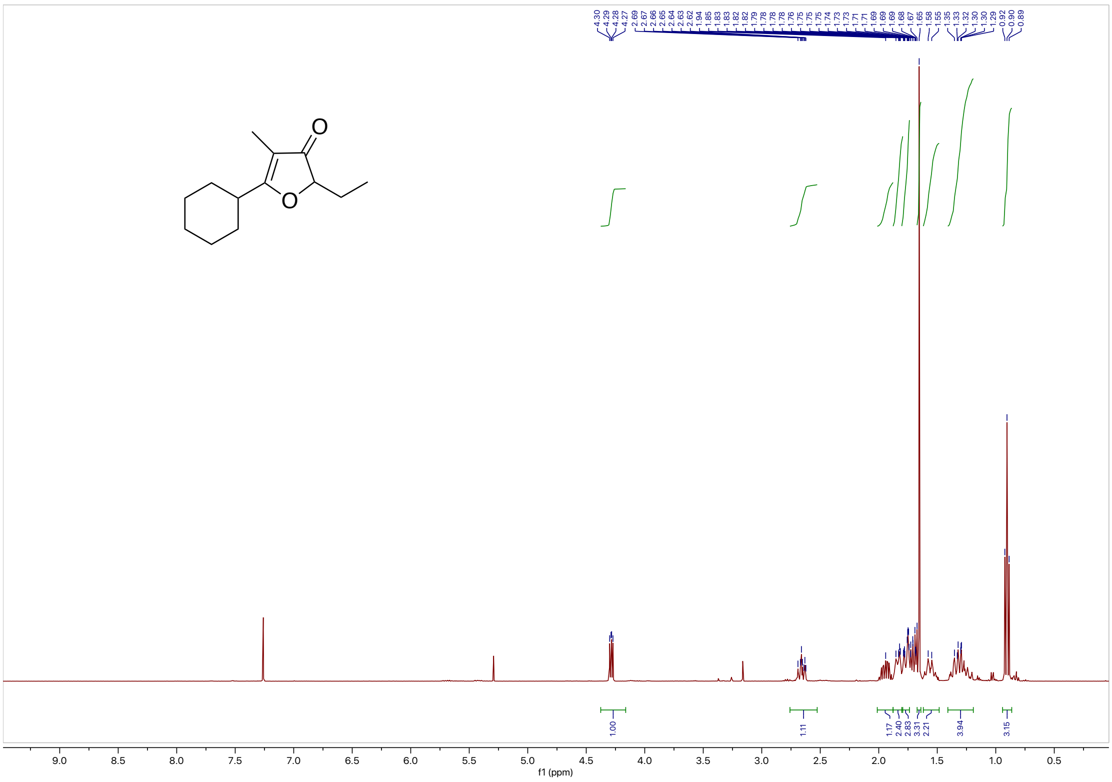

Supplementary Figure 97. ^{1}H NMR spectra ($CDCl_3$, 400 MHz) of **9c**

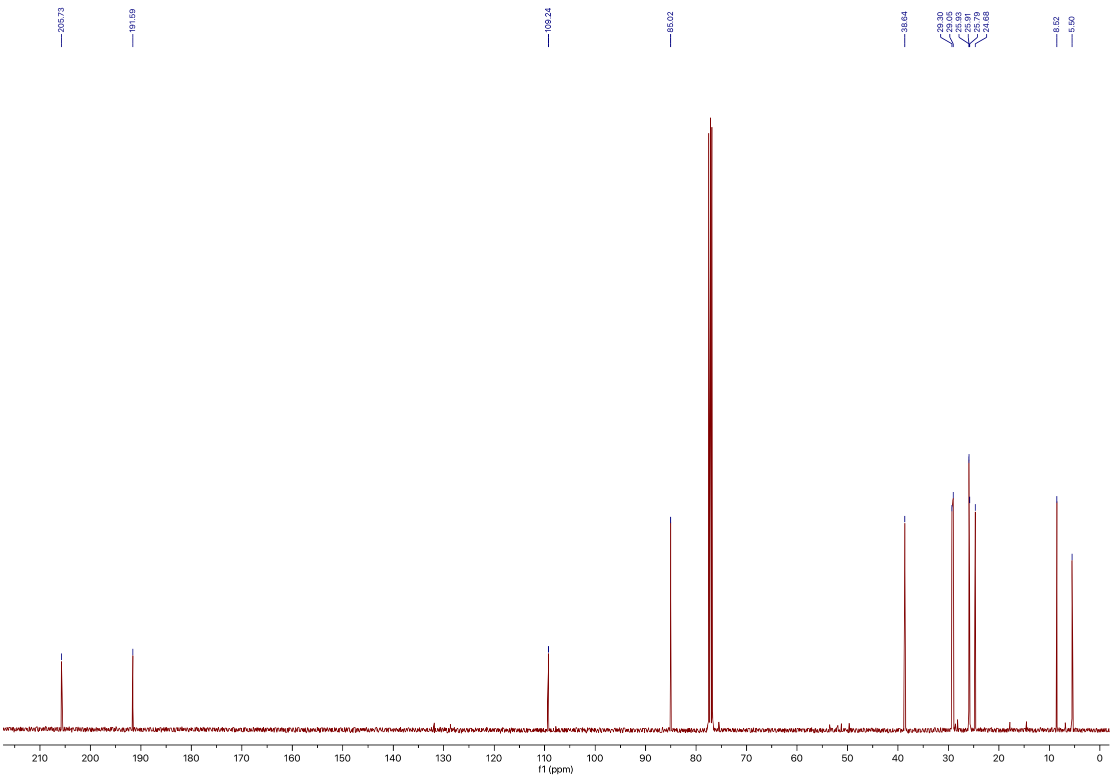

Supplementary Figure 98. ^{13}C NMR spectra ($CDCl_3$, 100 MHz) of **9c**

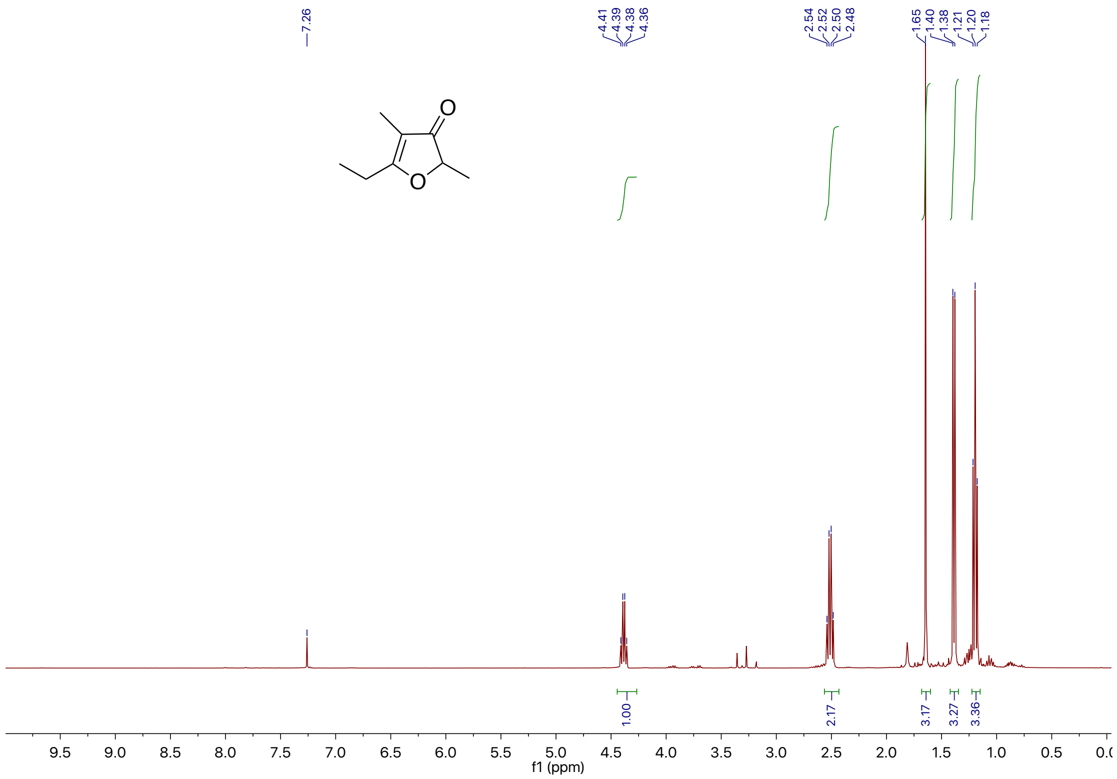

Supplementary Figure 99. ^{1}H NMR spectra ($CDCl_3$, 400 MHz) of **9d**

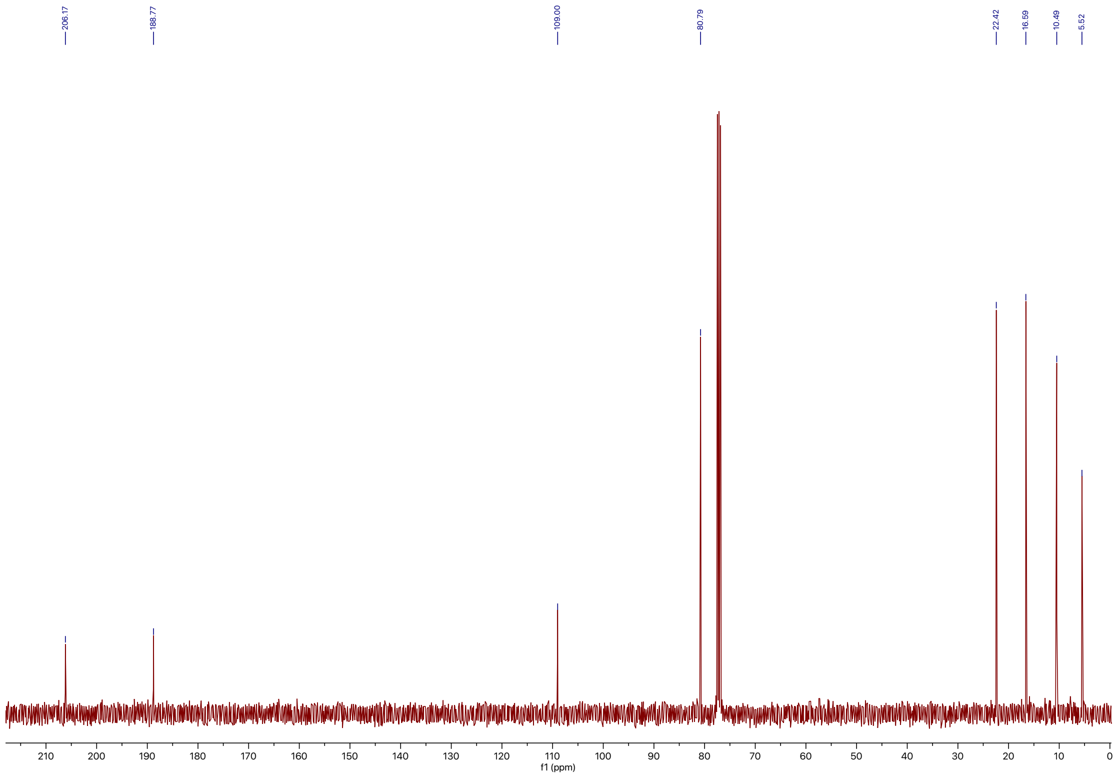

Supplementary Figure 100. ^{13}C NMR spectra ($CDCl_3$, 100 MHz) of **9d**

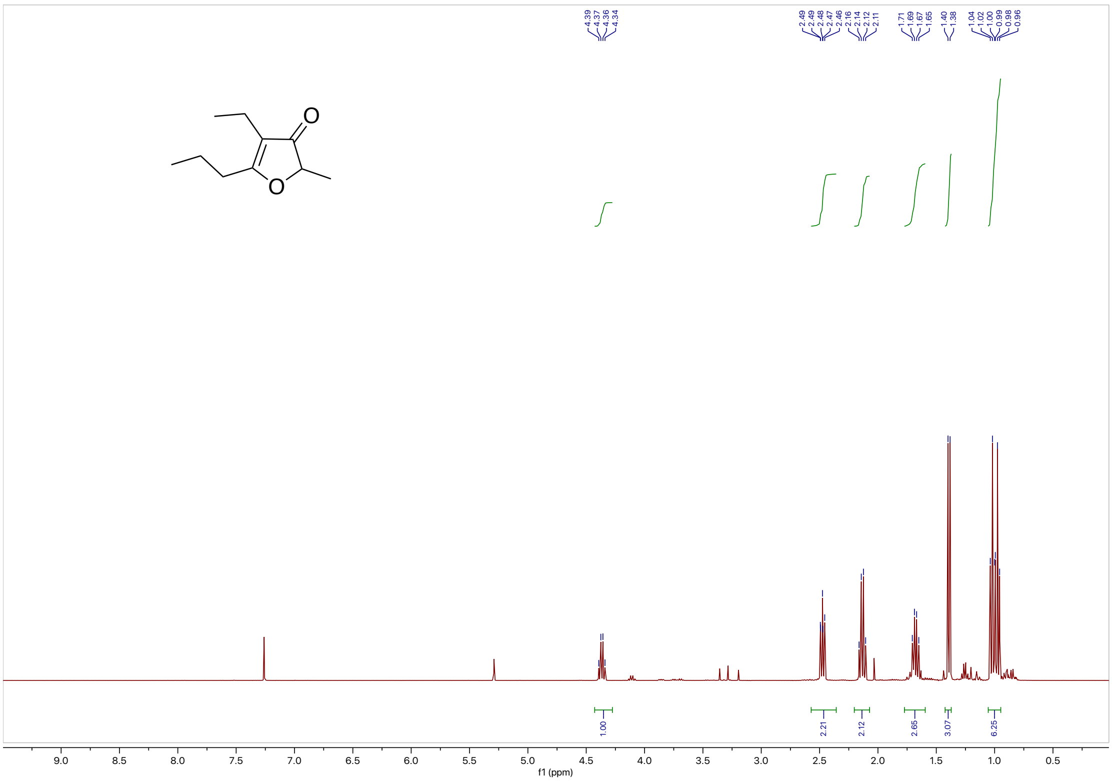

Supplementary Figure 101. ^{1}H NMR spectra ($CDCl_3$, 400 MHz) of **9e**

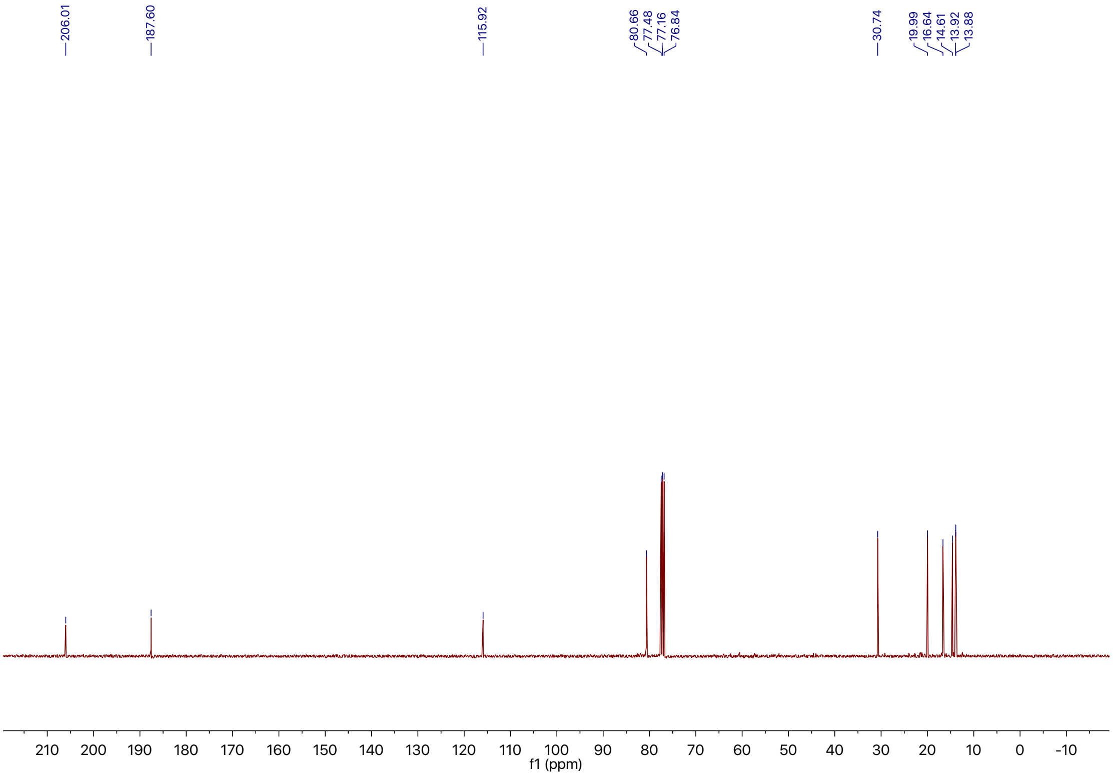

Supplementary Figure 102. ^{13}C NMR spectra ($CDCl_3$, 100 MHz) of **9e**

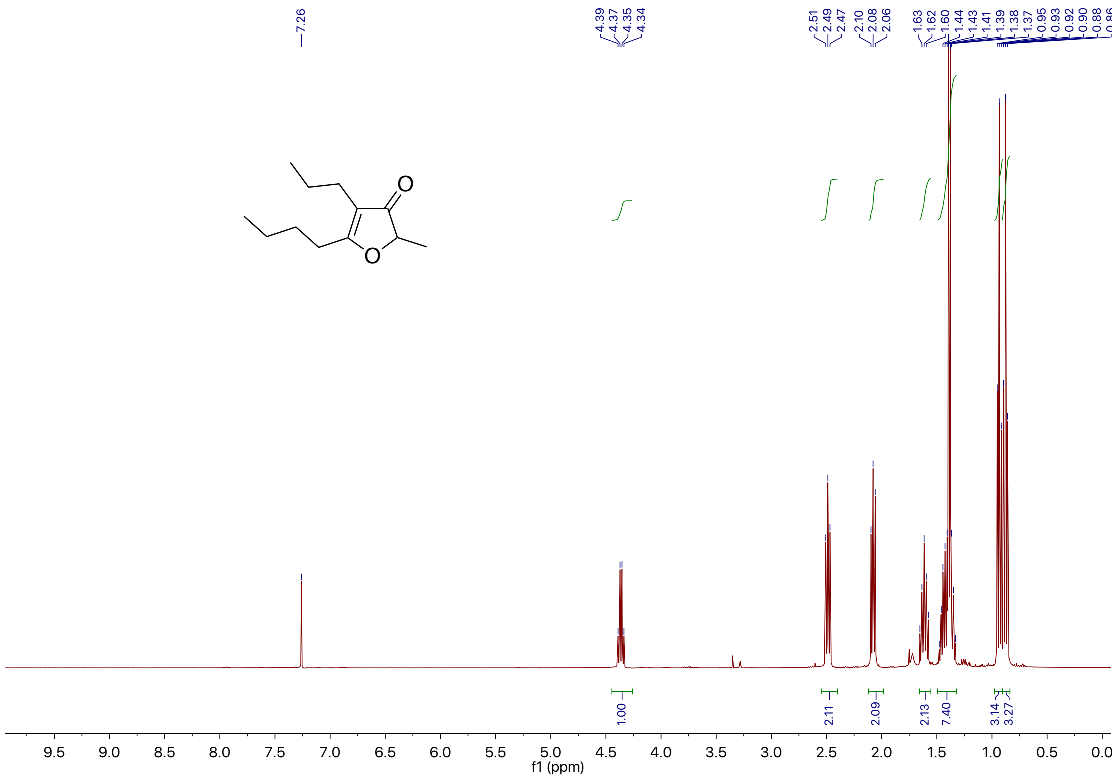

Supplementary Figure 103. ^{1}H NMR spectra ($CDCl_3$, 400 MHz) of **9f**

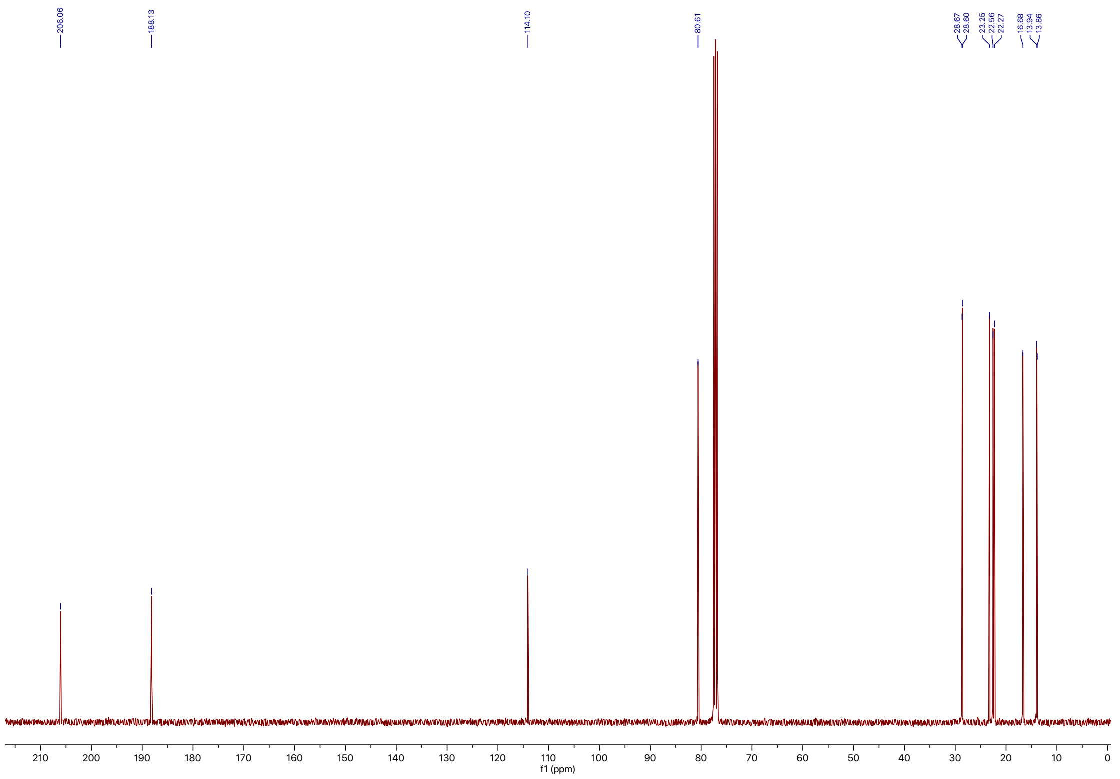

Supplementary Figure 104. ^{13}C NMR spectra ($CDCl_3$, 100 MHz) of **9f**

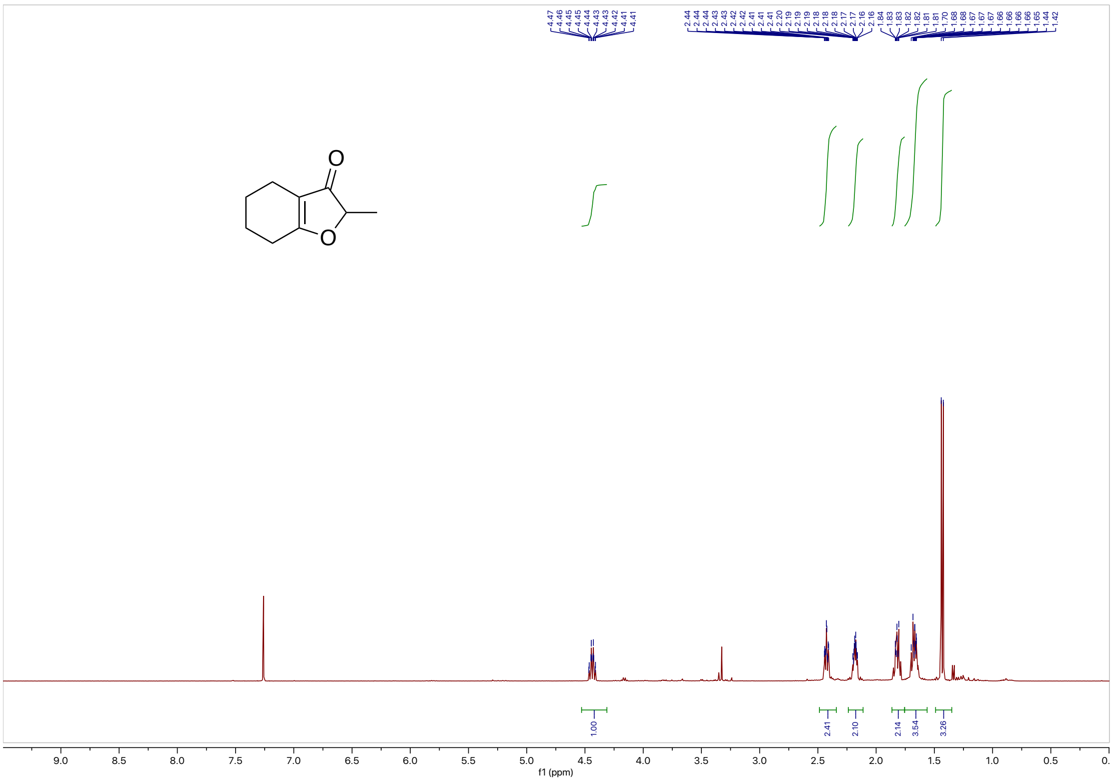

Supplementary Figure 105. ^{1}H NMR spectra ($CDCl_3$, 400 MHz) of **9g**

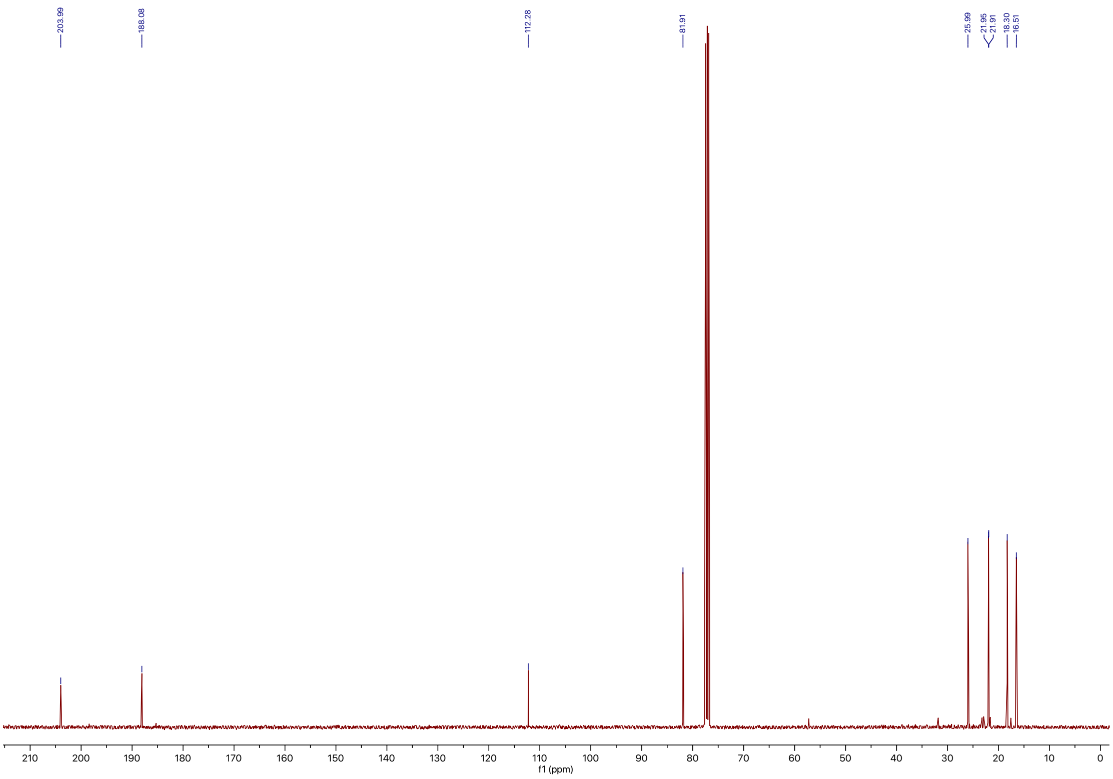

Supplementary Figure 106. ^{13}C NMR spectra ($CDCl_3$, 100 MHz) of **9g**

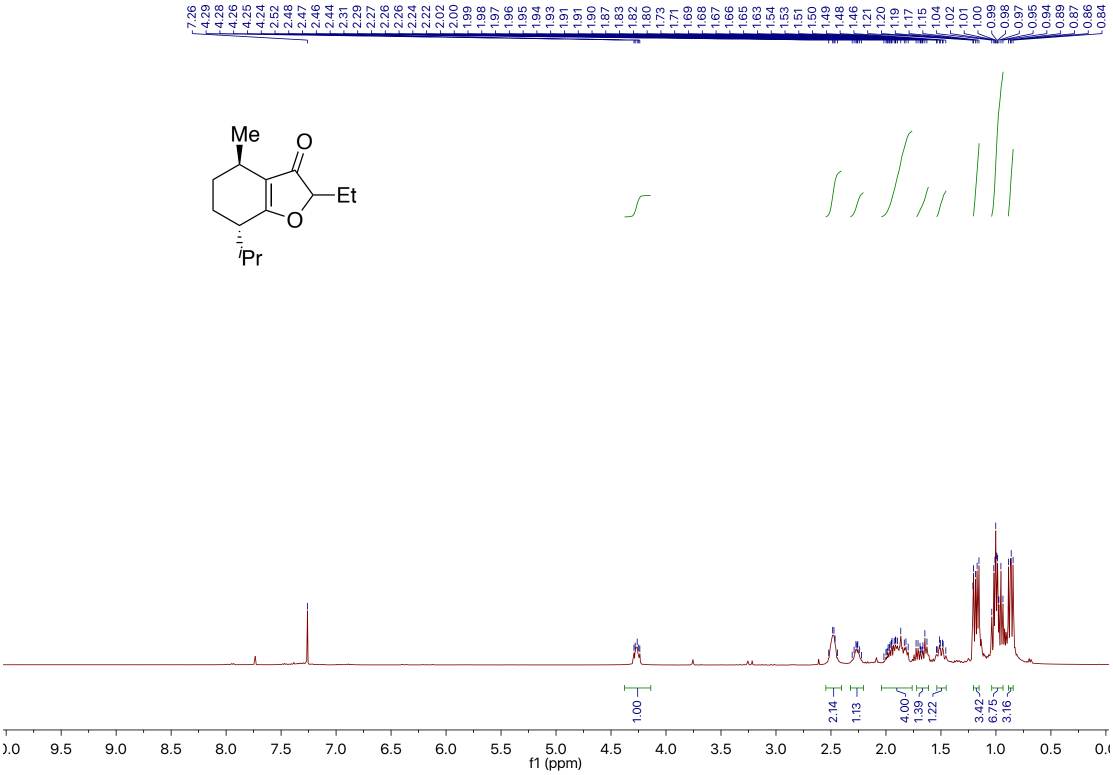

Supplementary Figure 107. ^{1}H NMR spectra ($CDCl_3$, 400 MHz) of **9h**

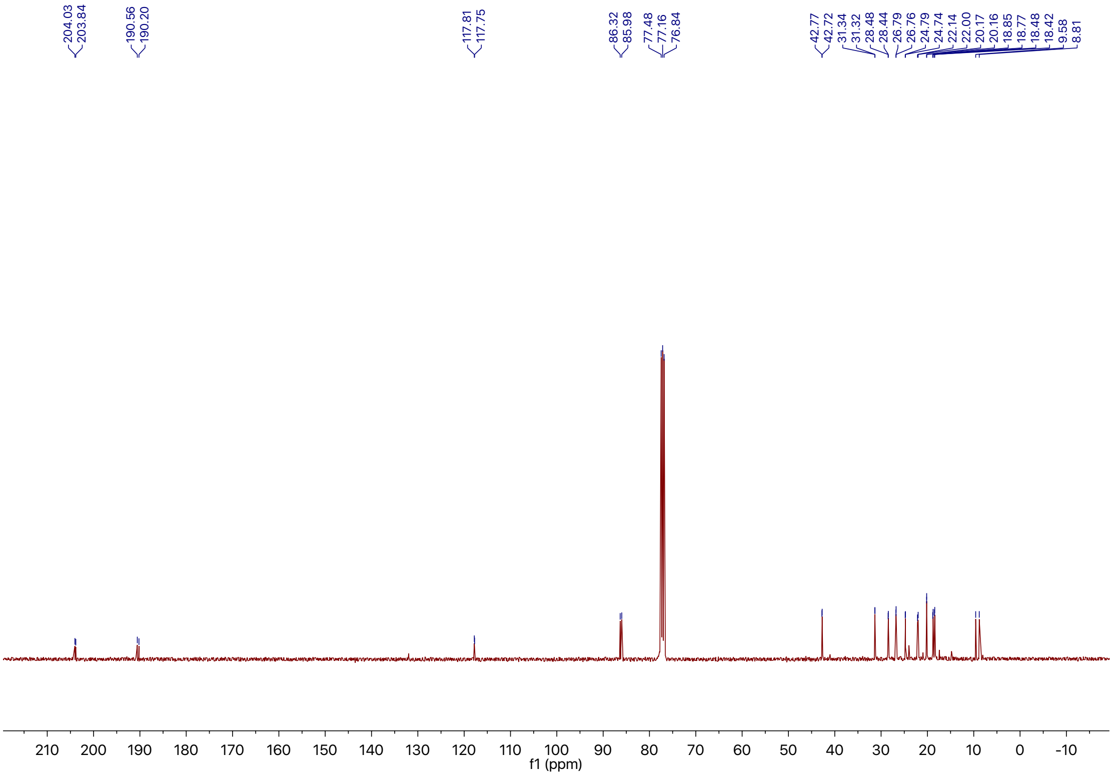

Supplementary Figure 108. ^{13}C NMR spectra ($CDCl_3$, 100 MHz) of **9h**

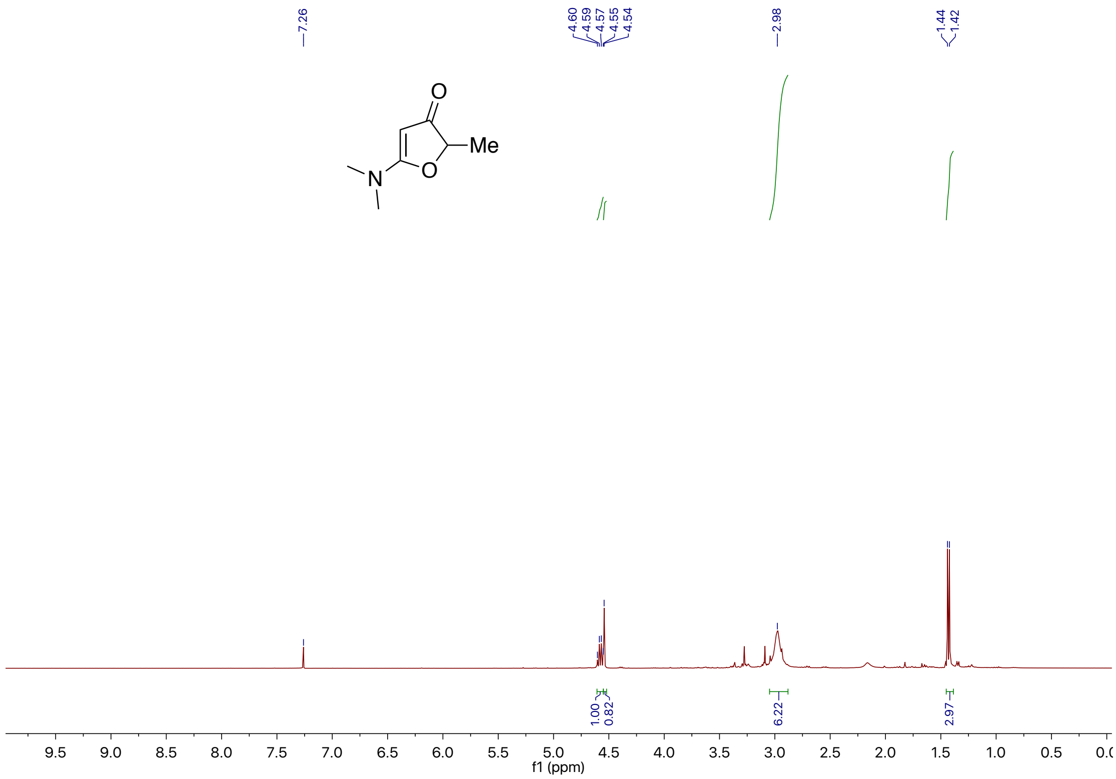

Supplementary Figure 109. ${}^{1}H$ NMR spectra ($CDCl_3$, 400 MHz) of **9i**

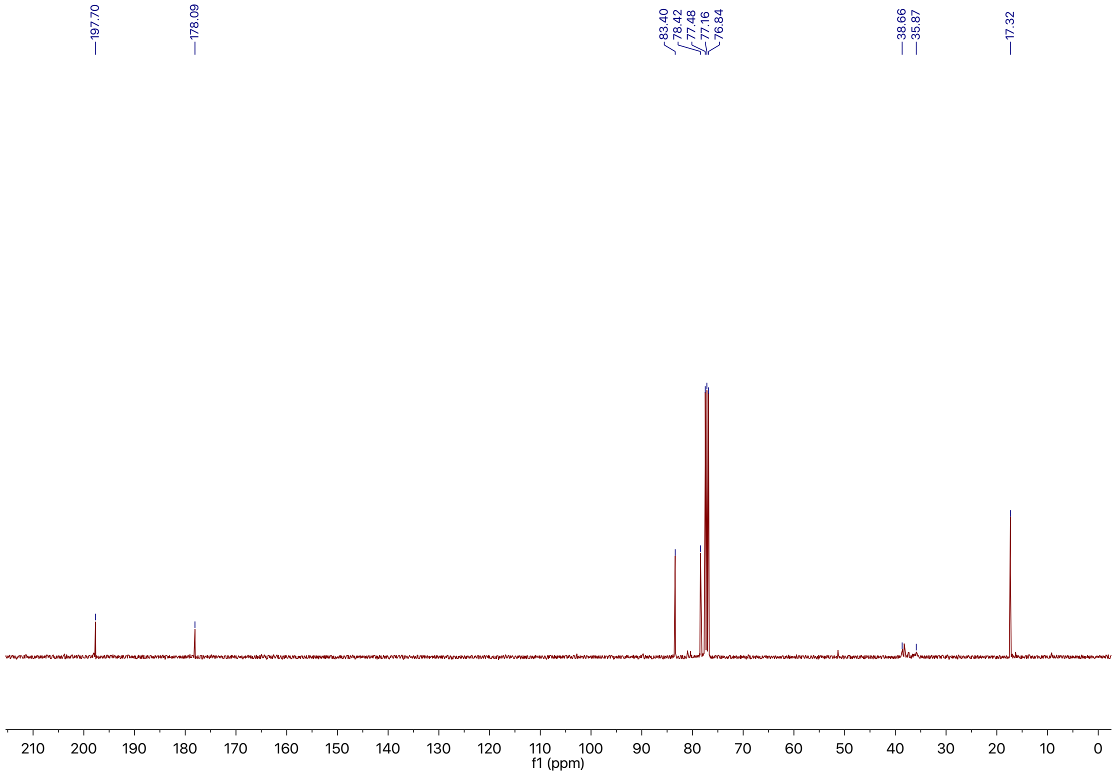

Supplementary Figure 110. ${}^{13}C$ NMR spectra ($CDCl_3$, 100 MHz) of **9i**

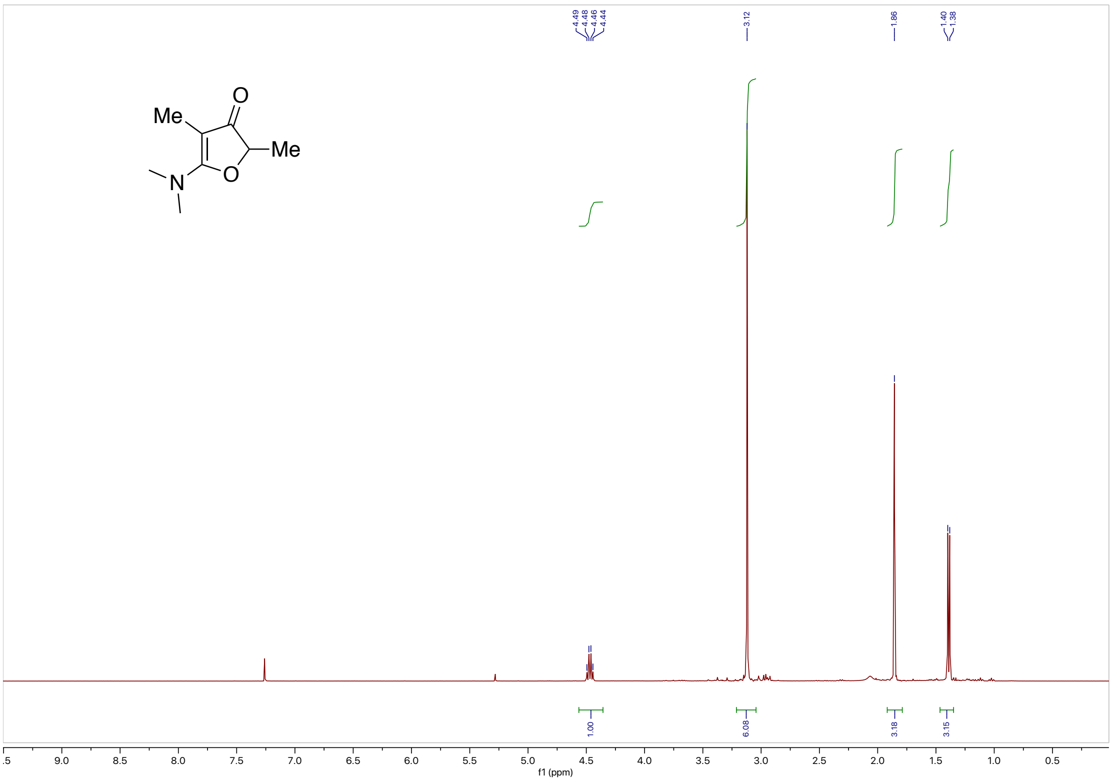

Supplementary Figure 111. ^{1}H NMR spectra ($CDCl_3$, 400 MHz) of **9j**

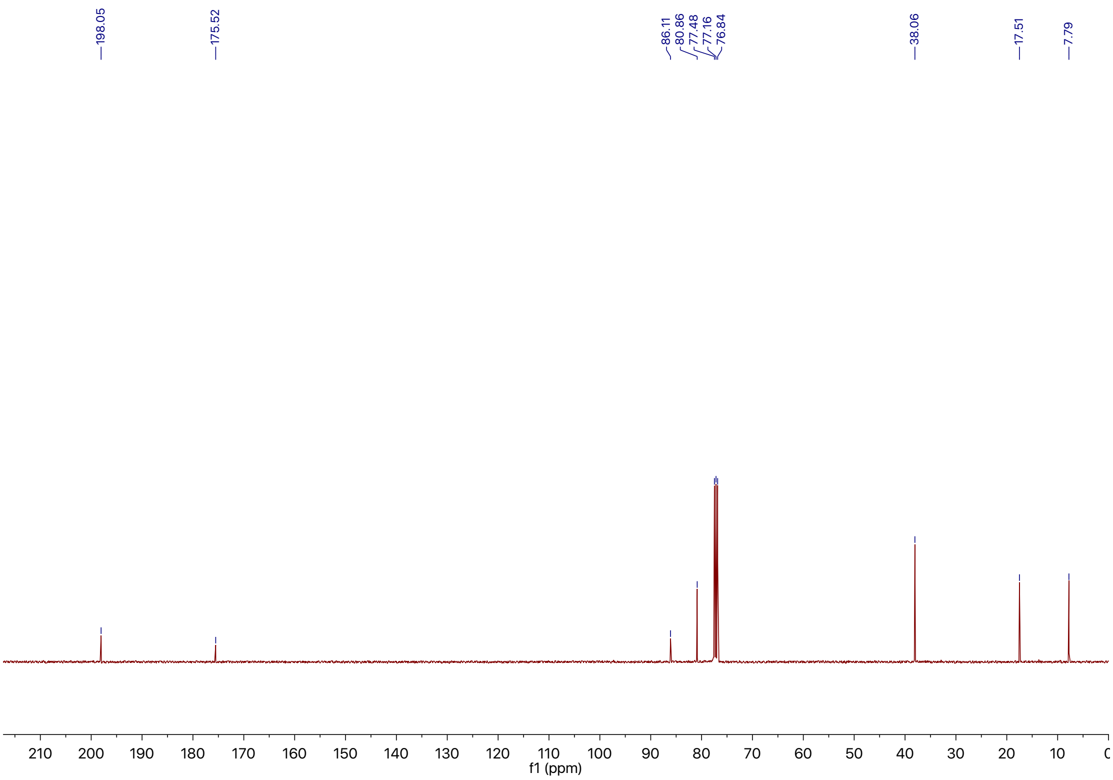

Supplementary Figure 112. ^{13}C NMR spectra ($CDCl_3$, 100 MHz) of **9j**

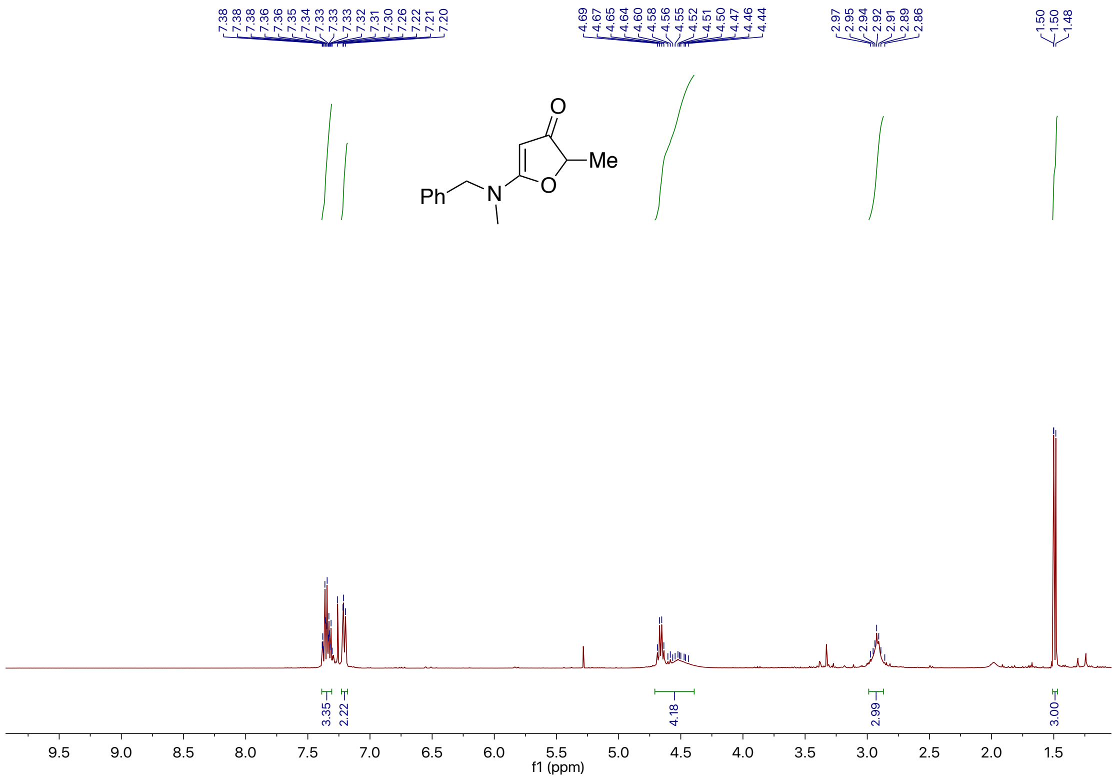

Supplementary Figure 113. ^{1}H NMR spectra ($CDCl_3$, 400 MHz) of **9k**

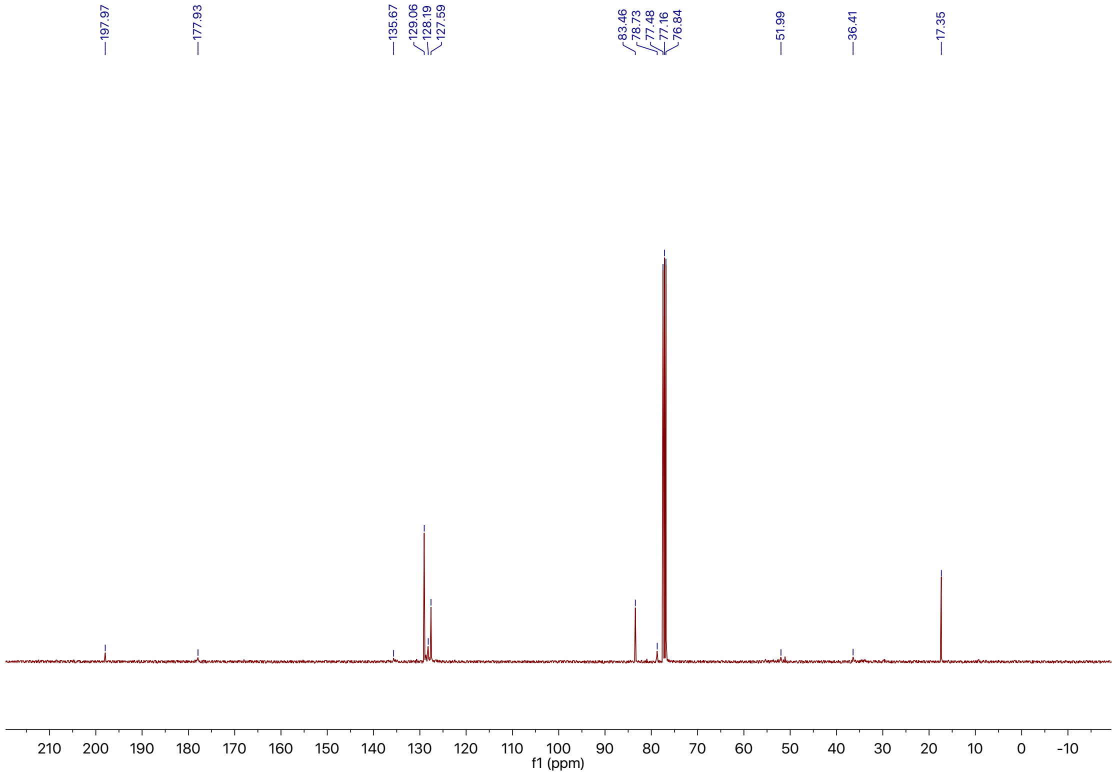

Supplementary Figure 114. ^{13}C NMR spectra ($CDCl_3$, 100 MHz) of **9k**

Supplementary References

[1]Kim, J. W., Koike, T., Kotani, M., Yamaguchi, K., Mizuno, N. Synthetic Scope of $Ru(OH)_x/Al_2O_3$-Catalyzed Hydrogen-Transfer Reactions: An Application to Reduction of Allylic Alcohols by a Sequential Process of Isomerization/Meerwein–Ponndorf–Verley-Type Reduction. *Chem. Eur. J.* **14**, 4104-4109 (2008).

[2]Sanz-Marco, A., Martinez-Erro, S, Martín-Matute, B. Selective Synthesis of Unsymmetrical Aliphatic Acyloins through Oxidation of Iridium Enolates. *Chem. Eur. J.* **45**, 11564-11567 (2018).

[3]Ahlsten, N., Martín-Matute, B. Ir-catalysed formation of C-F bonds. From allylic alcohols to α-fluoroketones. *Chem. Commun*. **47**, 8331-8333 (2011).

[4]Ahlsten, N., Bermejo Gómez, A., Martín-Matute, B. Iridium-Catalyzed 1,3-Hydrogen Shift/Chlorination of Allylic Alcohols. *Angew. Chem. Int. Ed.* **52**, 6273-6276 (2013).

[5]Liao, L., Guo, R., Zhao, X. Organoselenium-Catalyzed Regioselective C-H Pyridination of 1,3-Dienes and Alkenes. *Angew. Chem. Int. Ed.* **56**, 3201-3205 (2017).

[6]Li, D. R., He, A., Falck, J. R. Enantioselective, Organocatalytic Reduction of Ketones using Bifunctional Thiourea-Amine Catalysts. *Org. Lett.* **12**, 1756-1759 (2010).

[7]Peters, R., Xin, Z. Q., Maier, F. Catalyst Versus Substrate Induced Selectivity: Kinetic Resolution by Palladacycle Catalyzed Allylic Imidate Rearrangements. *Chem. Asian J.* **5**, 1770-1774 (2010).

[8]Akai, S., Hanada, R., Fujiwara, N., Kita, Y., Egi, M. One-Pot Synthesis of Optically Active Allyl Esters via Lipase−Vanadium Combo Catalysis. *Org. Lett.* **12**, 4900-4903 (2010).

[9]Borg, T., Danielsson, J., Mohiti, M., Restorp, P., Somfai, P. Diastereoselective Nucleophilic Addition to Aldehydes with Polar α- and α,β-Substituents. *Adv. Synth. Catal.* **353**, 2022-2036 (2011).

[10]Ahlsten, N., Bartoszewicz, A., Agrawal, S., Martín-Matute, B. A Facile Synthesis of α-Fluoro Ketones Catalyzed by $[Cp*IrCl_2]_2$. *Synthesis* **16**, 2600-2608 (2011).

[11]Ida, A., Hoshiya, N., Uenishi. J. Alkene migration to the end-terminal carbon bearing a phenyl group over a chiral siloxy carbon center in Heck reaction. *Tetrahedron* **71**, 6442–6448 (2015).

[12]Trost, B. M. Kulawiec, R. J. J Chemoselectivity in the Ruthenium-Catalyzed Redox Isomerization of Allylic Alcohols. *J. Am. Chem. Soc.* **5**, 2027-2036 (1993).

[13]Zhao, Y., Truhlar, D. G. The M06 suite of density functionals for main group thermochemistry, thermochemical kinetics, noncovalent interactions, excited states, and transition elements: two new functionals and systematic testing of four M06-class functionals and 12 other functionals. *Theor. Chem. Acc*. **120**, 215-241 (2008).

[14] Gaussian 16, Revision B.01, M. J. Frisch, G. W. Trucks, H. B. Schlegel, G. E. Scuseria, M. A. Robb, J. R. Cheeseman, G. Scalmani, V. Barone, G. A. Petersson, H. Nakatsuji, X. Li, M. Caricato, A. V. Marenich, J. Bloino, B. G. Janesko, R. Gomperts, B. Mennucci, H. P. Hratchian, J. V. Ortiz, A. F. Izmaylov, J. L. Sonnenberg, D. Williams-Young, F. Ding, F. Lipparini, F. Egidi, J. Goings, B. Peng, A. Petrone, T. Henderson, D. Ranasinghe, V. G. Zakrzewski, J. Gao, N. Rega, G. Zheng, W. Liang, M. Hada, M. Ehara, K. Toyota, R. Fukuda, J. Hasegawa, M. Ishida, T. Nakajima, Y. Honda, O. Kitao, H. Nakai, T. Vreven, K. Throssell, J. A. Montgomery, Jr., J. E. Peralta, F. Ogliaro, M. J. Bearpark, J. J. Heyd, E. N. Brothers, K. N. Kudin, V. N. Staroverov, T. A. Keith, R. Kobayashi, J. Normand, K. Raghavachari, A. P. Rendell, J. C. Burant, S. S. Iyengar, J. Tomasi, M. Cossi, J. M. Millam, M. Klene, C. Adamo, R. Cammi, J. W. Ochterski, R. L. Martin, K. Morokuma, O. Farkas, J. B. Foresman, and D. J. Fox, Gaussian, Inc., Wallingford CT, 2016.

[15]Dolg, M., Wedig, U., Stoll, H., Preuss, H. Energy-adjusted *ab initio* pseudopotentials for the first row transition elements. *J. Chem. Phys.* **86**, 866 (1987). (b) Andrae, D., Haussermann, U., Dolg, M., Stoll H., Preuss, H. Energy-adjusted*ab initio* pseudopotentials for the second and third row transition elements. *Theor. Chim. Acta* **77**, 123-141 (1990).

[16]González, C., Schlegel, H. B. Reaction path following in mass-weighted internal coordinates. *J. Phys. Chem.* **94**, 5523-5527 (1990).

[17]Cancès, E., Mennucci, B., Tomasi, J. A New Integral Equation Formalism for thePolarizable Continuum Model: Theoretical Background and Applications to Isotropic and Anisotropic Dielectrics. *J. Chem. Phys.* **107**, 3032–3041 (1997).

[18] Cossi, M., Barone, V., Mennucci, B., Tomasi, J. Ab Initio Study of Ionic Solutions by a Polarizable Continuum Dielectric Model. *Chem. Phys. Lett.* **286**, 253–260 (1998).

[19] Tomasi, J., Mennucci, B., Cancès, E. The IEF Version of the PCM Solvation Method: an Overview of a New Method Addressed to Study Molecular Solutes at the QM Ab Initio Level. *J. Mol. Struct.* **464**, 211–226 (1999).
