# Supplementary material for: An umpolung strategy to react catalytic enols with nucleophiles
Source: Nat Commun. 2019 Nov 20;10:5244. doi: 10.1038/s41467-019-13175-5 (PMC6868166; doi:10.1038/s41467-019-13175-5)
Supplement: Supplementary file 3 — Description of Additional Supplementary Files [file 41467_2019_13175_MOESM3_ESM.pdf]

Description of Additional Supplementary Files

File Name: Supplementary Data 1

Description: The Cartesian Coordinates of the DFT calculations.
