# Supplementary material for: An umpolung strategy to react catalytic enols with nucleophiles
Source: Nat Commun. 2019 Nov 20;10:5244. doi: 10.1038/s41467-019-13175-5 (PMC6868166; doi:10.1038/s41467-019-13175-5)
Supplement: Supplementary file 4 — Supplementary Data 1 [file 41467_2019_13175_MOESM4_ESM.docx]

**Cartesian Coordinates of the computed structures**

**Enolonium B**

SCF = -1145.209873

Thermal correction to Gibbs Free Energy = 0.269859

C -2.312324 0.226812 -0.670034

C -1.264133 1.201477 -0.242072

O -2.996509 0.455931 -1.661665

C -1.492969 2.610423 -0.732341

H -1.088067 1.145039 0.835163

H -2.418613 3.020323 -0.309390

H -0.666806 3.271129 -0.447570

H -1.590814 2.636305 -1.822457

C 1.398412 1.719092 1.543214

C 2.243540 1.925887 2.632540

C 3.461106 1.257282 2.701884

C 3.852858 0.397958 1.680613

C 3.037844 0.187193 0.565242

C 1.823808 0.847760 0.556189

C 3.391448 -0.741886 -0.606217

O 2.712712 -0.316382 -1.734826

I 0.639256 0.375523 -1.207558

C 4.887924 -0.688908 -0.908333

C 2.996212 -2.172434 -0.204635

H 0.454506 2.252970 1.483993

H 1.944090 2.615009 3.417606

H 4.116759 1.413189 3.554850

H 4.809432 -0.116654 1.747872

H 5.197799 0.340092 -1.122213

H 5.501691 -1.081685 -0.088771

H 5.081687 -1.301222 -1.796093

H 3.218035 -2.853662 -1.035178

H 3.544792 -2.512056 0.683321

H 1.922387 -2.238162 0.016353

C -2.479857 -1.047106 0.089750

C -3.518361 -1.901101 -0.298140

C -1.647617 -1.428682 1.149607

C -3.728103 -3.104646 0.359785

H -4.154461 -1.595559 -1.124887

C -1.852242 -2.637548 1.803609

H -0.822640 -0.795305 1.470779

C -2.893403 -3.475351 1.412028

H -4.541220 -3.757570 0.052542

H -1.196994 -2.925970 2.621470

H -3.053512 -4.419302 1.927434

**Enolonium B’**

SCF = -1145.19241638

Thermal correction to Gibbs Free Energy = 0.269711

C -2.387312 -0.985691 0.714751

C -3.116156 -2.019661 1.187698

O -1.139278 -0.788019 1.200419

C -4.567547 -2.294598 0.974633

H -2.585422 -2.681978 1.873505

H -4.733824 -3.163860 0.322055

H -5.059329 -2.528578 1.927372

H -5.092302 -1.444538 0.526644

C 0.759687 1.441765 1.689605

C 1.483754 2.503321 2.229506

C 2.748619 2.808917 1.733956

C 3.309627 2.057570 0.705793

C 2.604130 0.994260 0.138739

C 1.344962 0.743958 0.650437

C 3.160838 0.053109 -0.925896

O 2.091288 -0.593625 -1.573744

I 0.430958 -0.939581 -0.312354

C 3.918368 0.812198 -2.007316

C 4.075839 -0.965178 -0.242400

H -0.216759 1.153471 2.067237

H 1.055556 3.086310 3.040660

H 3.310052 3.636570 2.159892

H 4.308697 2.294574 0.344343

H 3.275184 1.571951 -2.463448

H 4.816617 1.298096 -1.609384

H 4.232248 0.105496 -2.783155

H 4.475101 -1.657268 -0.992914

H 4.915258 -0.472910 0.263515

H 3.522196 -1.546021 0.505941

C -2.876183 0.011788 -0.270293

C -2.517553 1.358546 -0.133818

C -3.646414 -0.362300 -1.378239

C -2.942844 2.307341 -1.055533

H -1.901551 1.658282 0.711394

C -4.069369 0.585588 -2.304676

H -3.894419 -1.411396 -1.527185

C -3.723122 1.924436 -2.144411

H -2.662661 3.350380 -0.925754

H -4.661965 0.274425 -3.162087

H -4.052090 2.665237 -2.869437

**Enolonium C**

SCF = -1713.706968

Thermal correction to Gibbs Free Energy = 0.366748

C 2.815406 -3.662908 2.016777

C 3.223820 -2.501787 2.668856

C 2.950238 -1.260413 2.099809

C 2.273149 -1.183006 0.888321

C 1.847899 -2.341566 0.228986

C 2.133071 -3.583390 0.807393

C 1.114074 -2.220569 -1.068597

C 0.095890 -3.129234 -1.357359

O 1.324149 -1.188202 -1.800326

C -0.520463 -3.263421 -2.715448

H 3.037045 -4.636548 2.448137

H 3.758907 -2.565280 3.613574

H 3.267276 -0.346059 2.596799

H 2.057522 -0.210625 0.451347

H 1.837169 -4.495762 0.292874

H -0.081659 -3.938186 -0.649292

H -0.078827 -4.102078 -3.273526

H -1.599558 -3.455760 -2.660891

H -0.361385 -2.356731 -3.309230

C -4.077069 0.143219 0.525117

C -5.011960 1.028292 1.056254

C -4.664955 2.357990 1.258213

C -3.392327 2.806114 0.922094

C -2.437729 1.942640 0.380166

C -2.810764 0.608593 0.204496

C -1.033817 2.420558 0.057354

O -0.630095 1.644378 -1.079409

I -1.341173 -0.792918 -0.598955

C -0.960040 3.887218 -0.337241

C -0.109399 2.148509 1.242288

H -4.324768 -0.903053 0.372120

H -6.007409 0.672081 1.308798

H -5.387356 3.055414 1.674379

H -3.135941 3.850589 1.083843

H -1.650226 4.107518 -1.158109

H -1.181430 4.551391 0.504868

H 0.062181 4.107316 -0.667351

H 0.915739 2.450275 0.999427

H -0.439915 2.721623 2.115629

H -0.101793 1.086338 1.515009

H 1.904219 0.267369 -1.548106

O -2.527982 -2.285297 0.144686

C -2.175552 -2.644801 1.466254

H -1.128140 -2.977180 1.539858

H -2.341814 -1.823303 2.180507

H -2.824670 -3.482926 1.742243

O 2.018726 1.254699 -1.429302

H 0.344908 1.714987 -1.189858

C 3.361955 1.608499 -1.349193

H 4.014616 0.736449 -1.202875

H 3.707084 2.145195 -2.245004

C 3.570130 2.526964 -0.173464

F 2.764446 3.596714 -0.237276

F 4.830349 2.965392 -0.135291

F 3.315501 1.918113 0.994543

**Enolonium C’**

SCF = -1713.69844550

Thermal correction to Gibbs Free Energy = 0.372381

C -3.295053 2.654316 1.964969

C -2.527019 3.773303 1.652130

C -1.844307 3.818353 0.438091

C -1.922371 2.747097 -0.447318

C -2.694458 1.617308 -0.145143

C -3.378526 1.587798 1.073629

C -2.749883 0.476222 -1.108269

C -3.971866 0.047888 -1.526786

O -1.582685 0.013886 -1.508451

C -4.183832 -0.983132 -2.589331

H -3.827369 2.608506 2.912892

H -2.461972 4.606162 2.348429

H -1.249922 4.692228 0.177118

H -1.416215 2.802628 -1.411920

H -3.968601 0.706512 1.321414

H -4.848393 0.553474 -1.121810

H -4.754398 -1.858976 -2.242108

H -4.742552 -0.580531 -3.446922

H -3.222810 -1.349696 -2.969111

C 3.907885 1.323815 -0.363498

C 5.235130 0.931530 -0.207088

C 5.538115 -0.398545 0.062254

C 4.524070 -1.343687 0.173479

C 3.186506 -0.978152 0.019612

C 2.920643 0.362312 -0.232713

C 2.049248 -1.960900 0.208411

O 0.998910 -1.448075 -0.632976

I 0.860267 0.955691 -0.517606

C 2.358817 -3.369349 -0.264005

C 1.638585 -1.951110 1.676848

H 3.645385 2.356675 -0.578812

H 6.027291 1.669261 -0.302460

H 6.573267 -0.706860 0.181894

H 4.774808 -2.380752 0.385623

H 2.705990 -3.367914 -1.301919

H 3.114348 -3.848017 0.367232

H 1.444750 -3.972112 -0.195801

H 0.805545 -2.635743 1.853386

H 2.486724 -2.269656 2.292863

H 1.343769 -0.946368 2.000796

H -1.524194 -1.512928 -1.436429

O 1.392421 2.889255 -0.237708

C 1.468773 3.260283 1.131782

H 0.506434 3.131352 1.647410

H 2.249037 2.699533 1.667304

H 1.730342 4.323433 1.142929

O -1.394171 -2.502207 -1.212695

H 0.159085 -1.971246 -0.588752

C -2.449567 -2.876816 -0.377981

H -3.323083 -2.220878 -0.496205

H -2.755823 -3.912492 -0.573275

C -2.035133 -2.790725 1.063963

F -1.077778 -3.690624 1.354437

F -3.061161 -3.015820 1.886053

F -1.531721 -1.579886 1.350153

**C_no_tfe**

SCF = -1260.908579

Thermal correction to Gibbs Free Energy = 0.320616

C -4.707993 -0.226835 -0.540781

C -5.342999 1.012112 -0.550081

C -4.722489 2.120391 0.024822

C -3.473801 1.982067 0.610844

C -2.830326 0.737362 0.628743

C -3.454493 -0.373041 0.044399

C -1.503688 0.672793 1.294212

C -0.732729 -0.634454 1.338452

O -1.013836 1.655287 1.829686

C 0.382219 -0.636463 2.340393

H -5.191955 -1.087804 -0.995524

H -6.323364 1.116279 -1.009293

H -5.215458 3.089233 0.014881

H -2.971943 2.831221 1.067674

H -2.957385 -1.346427 0.010381

H -1.382978 -1.513004 1.382674

H -0.055448 -0.535148 3.341381

H 0.965503 -1.558998 2.293957

H 1.048942 0.218077 2.188200

C 0.582068 1.930673 -0.994736

C 1.163535 3.189576 -1.037652

C 2.494316 3.324679 -0.661417

C 3.209858 2.210167 -0.252086

C 2.642551 0.926954 -0.191221

C 1.299184 0.802460 -0.576598

C 3.584271 -0.197349 0.237502

O 2.835002 -1.393317 0.377059

I 0.071560 -1.027604 -0.716884

C 4.641272 -0.407485 -0.847092

C 4.241534 0.113943 1.581794

H -0.460269 1.824340 -1.296069

H 0.585079 4.050260 -1.363676

H 2.980963 4.296551 -0.689558

H 4.253810 2.341179 0.025940

H 4.156248 -0.668335 -1.794688

H 5.254782 0.485971 -1.004003

H 5.309777 -1.229299 -0.559297

H 4.882961 -0.728170 1.872555

H 4.871837 1.007759 1.551703

H 3.479104 0.250027 2.357028

O -1.478346 -2.758512 -0.534502

C -0.946411 -3.841797 0.134403

H -0.713047 -3.640919 1.206778

H -1.647782 -4.696797 0.139841

H -0.002381 -4.227198 -0.308619

H 3.442455 -2.077951 0.681261

**C’’**

SCF = -1713.73424826

Thermal correction to Gibbs Free Energy = 0.371761

C 3.194484 3.057476 0.288126

C 3.012132 3.641735 1.538819

C 1.727071 3.805933 2.052680

C 0.629616 3.382470 1.317778

C 0.800816 2.783822 0.062336

C 2.096159 2.629725 -0.447507

C -0.416452 2.303257 -0.640077

C -0.286278 1.326317 -1.760587

O -1.531358 2.613992 -0.215828

C -1.481807 1.224727 -2.674478

H 4.195144 2.935294 -0.117991

H 3.873854 3.971250 2.114044

H 1.583695 4.261958 3.028747

H -0.380019 3.497343 1.703931

H 2.265715 2.183467 -1.424072

H 0.654323 1.427137 -2.303095

H -1.656196 2.187001 -3.171494

H -1.314350 0.467277 -3.446225

H -2.396308 0.966176 -2.130636

C 2.501701 -0.503109 -2.209484

C 3.847630 -0.781591 -2.435851

C 4.570757 -1.507490 -1.494680

C 3.952742 -1.983764 -0.343857

C 2.599010 -1.737491 -0.096211

C 1.936506 -0.966609 -1.034050

C 1.848164 -2.244049 1.141002

O 0.490385 -2.338518 0.841516

I -0.134686 -0.596625 -0.532096

C 2.338087 -3.632301 1.545764

C 2.105162 -1.252756 2.285028

H 1.927131 0.040064 -2.955241

H 4.318849 -0.436662 -3.352067

H 5.622698 -1.720203 -1.666525

H 4.528895 -2.563726 0.374174

H 2.237038 -4.333986 0.710483

H 3.380708 -3.631989 1.885053

H 1.715764 -3.986984 2.374678

H 1.566350 -1.587138 3.181380

H 3.172712 -1.182772 2.527982

H 1.752335 -0.245367 2.023401

H -3.218401 2.021961 -0.408313

O -1.490836 -1.179187 2.359613

C -1.069767 -0.035412 3.052075

H -1.952012 0.443033 3.493209

H -0.367630 -0.259388 3.870229

H -0.586177 0.712868 2.394153

O -4.068254 1.546768 -0.356339

H -0.704794 -1.653495 2.005742

C -3.919023 0.500431 0.551721

H -2.898506 0.398927 0.951848

H -4.599643 0.599062 1.410662

C -4.244746 -0.805946 -0.123901

F -5.457160 -0.793678 -0.683340

F -4.205196 -1.816516 0.747850

F -3.363653 -1.086103 -1.103677

**C_2TFE**

SCF = -1845.08292977

Thermal correction to Gibbs Free Energy = 0.265797

C 2.630912 4.361593 -0.371201

C 1.652623 5.075423 0.316567

C 0.380999 4.531523 0.490169

C 0.087740 3.273864 -0.015664

C 1.070341 2.541979 -0.697416

C 2.342591 3.101048 -0.878309

C 0.716671 1.179043 -1.156308

C 1.778571 0.300814 -1.772607

O -0.415921 0.732828 -1.041304

C 1.259330 -0.795066 -2.653382

H 3.620801 4.787178 -0.512052

H 1.882290 6.059689 0.717595

H -0.381301 5.091166 1.026348

H -0.892626 2.826078 0.137078

H 3.124775 2.559128 -1.404899

H 2.591158 0.848296 -2.252719

H 0.782647 -0.334111 -3.528457

H 2.091951 -1.411914 -3.001570

H 0.513729 -1.404792 -2.139999

C 1.571288 0.915685 2.096537

C 0.782787 1.225137 3.200502

C -0.082291 0.264270 3.700326

C -0.152859 -0.986659 3.096644

C 0.625688 -1.319076 1.983350

C 1.497742 -0.333903 1.480675

C 0.534933 -2.726156 1.398109

O 0.424024 -2.527929 -0.023744

I 3.011094 -0.598954 -0.124518

C 1.790339 -3.530136 1.730465

C -0.683265 -3.520114 1.858619

H 2.257632 1.671144 1.714464

H 0.849817 2.209432 3.657971

H -0.709126 0.480543 4.561814

H -0.840221 -1.720456 3.508158

H 2.702103 -3.047135 1.369158

H 1.875786 -3.655247 2.815740

H 1.730325 -4.528561 1.278809

H -0.693136 -4.477197 1.322449

H -0.642511 -3.755438 2.927105

H -1.616940 -2.994300 1.633161

H -1.864687 0.671748 0.682034

O 4.381060 -0.626357 -1.987378

C 4.871633 -1.895971 -2.196066

H 5.701111 -1.893857 -2.927683

H 5.292709 -2.377526 -1.282969

H 4.116276 -2.614695 -2.591021

O -2.266205 1.346377 1.260441

H 0.360794 -3.396116 -0.444410

C -3.636377 1.313364 1.021489

H -4.042992 0.293632 0.989282

H -4.154781 1.869217 1.811148

C -3.974561 1.974859 -0.290780

F -3.627976 3.268944 -0.300555

F -5.288370 1.904849 -0.536844

F -3.335025 1.395357 -1.316443

C -2.552374 -1.643414 -1.213632

C -3.953635 -2.162434 -1.045112

H -2.572180 -0.831809 -1.954411

H -1.956492 -2.471356 -1.624677

F -4.792954 -1.214586 -0.599377

F -4.428677 -2.597602 -2.216582

F -4.007992 -3.176463 -0.176757

O -2.075492 -1.223952 0.031119

H -1.118624 -1.414441 0.020104

**TS1**

SCF = -1713.665327

Thermal correction to Gibbs Free Energy = 0.359327

C 1.083086 -4.032780 1.430980

C 2.184062 -3.626141 2.182255

C 2.755144 -2.374110 1.964792

C 2.230805 -1.529801 0.996419

C 1.126734 -1.933373 0.232658

C 0.554072 -3.194235 0.459956

C 0.574333 -0.999183 -0.772907

C -0.462184 -1.493310 -1.720260

O 0.969503 0.159956 -0.900625

C -0.911997 -0.635771 -2.820367

H 0.630951 -5.005007 1.608578

H 2.594818 -4.285440 2.943114

H 3.611729 -2.055836 2.553255

H 2.671734 -0.550372 0.830148

H -0.335848 -3.503561 -0.087468

H -0.545938 -2.563802 -1.864460

H -0.177467 -0.755024 -3.634667

H -1.870046 -0.988811 -3.217375

H -0.967752 0.419194 -2.540740

C -0.387207 0.527616 2.186864

C 0.400705 1.457399 2.855589

C 0.297528 2.796582 2.510574

C -0.583095 3.188927 1.507718

C -1.380216 2.276348 0.808613

C -1.273021 0.924066 1.181328

C -2.359004 2.785485 -0.247110

O -2.189050 1.951806 -1.390343

I -2.274100 -0.764961 0.257275

C -3.787760 2.682731 0.288770

C -2.096718 4.225638 -0.674875

H -0.307927 -0.525843 2.452269

H 1.088282 1.129720 3.631553

H 0.903579 3.545059 3.014877

H -0.641128 4.245185 1.261838

H -4.047273 1.653480 0.556639

H -3.908144 3.311502 1.178448

H -4.500024 3.032079 -0.470518

H -2.778362 4.469609 -1.498505

H -2.287874 4.946976 0.127028

H -1.069696 4.350395 -1.034326

H 2.040522 1.289756 0.033340

O -2.458606 -2.880853 -1.109490

C -3.529815 -2.890506 -1.953906

H -3.641450 -3.833369 -2.527633

H -4.498428 -2.747612 -1.420155

H -3.513351 -2.065739 -2.710899

O 2.809820 1.835718 0.282389

H -2.898260 2.157961 -2.011493

C 3.555741 2.049445 -0.874011

H 2.938073 2.185900 -1.773807

H 4.169931 2.949188 -0.748407

C 4.502067 0.906465 -1.152597

F 5.296545 0.653214 -0.107378

F 5.282823 1.191913 -2.202407

F 3.848825 -0.230086 -1.432943

**TS1_no_TFE**

SCF = -1260.871011

Thermal correction to Gibbs Free Energy = 0.317736

C -4.128484 1.224975 -0.764798

C -4.043946 2.605195 -0.589499

C -3.062362 3.147507 0.237438

C -2.169598 2.308763 0.889669

C -2.250329 0.922447 0.721918

C -3.236376 0.382524 -0.114444

C -1.247786 0.084579 1.423972

C -1.371246 -1.400675 1.360916

O -0.353373 0.573658 2.106023

C -0.428970 -2.228317 2.121944

H -4.889106 0.804976 -1.417929

H -4.743192 3.260179 -1.103859

H -2.993725 4.224261 0.369923

H -1.388527 2.709232 1.531315

H -3.281239 -0.690929 -0.295277

H -2.355614 -1.815406 1.176301

H -0.786604 -2.249588 3.164487

H -0.441442 -3.263058 1.764614

H 0.587287 -1.824645 2.106290

C 0.565767 1.725781 -1.368088

C 1.096494 3.003041 -1.272946

C 2.259406 3.192637 -0.535903

C 2.859980 2.109859 0.086629

C 2.347776 0.804264 0.008916

C 1.182806 0.626087 -0.753224

C 3.128722 -0.253122 0.787643

O 2.531372 -1.521949 0.576812

I 0.003697 -1.179190 -1.034667

C 4.572913 -0.322812 0.289876

C 3.076131 0.066147 2.281677

H -0.354585 1.575941 -1.931331

H 0.601326 3.837744 -1.762881

H 2.698296 4.182456 -0.437747

H 3.763904 2.285722 0.666519

H 4.591719 -0.578324 -0.775290

H 5.116778 0.616046 0.432856

H 5.109811 -1.103780 0.843785

H 3.598519 -0.720772 2.842157

H 3.559255 1.019302 2.520768

H 2.031726 0.108529 2.611940

O -1.906670 -2.775801 -0.624666

C -1.554138 -4.093483 -0.645075

H -0.806823 -4.372820 0.139967

H -2.407664 -4.790637 -0.516439

H -1.063559 -4.390345 -1.601322

H 3.020354 -2.157420 1.113429

**TS_1_2TFE**

SCF = -1558.96869049

Thermal correction to Gibbs Free Energy = 0.306584

C 3.452683 3.880174 -0.150175

C 2.639278 4.712663 0.616637

C 1.297844 4.392577 0.814775

C 0.766775 3.241495 0.249473

C 1.578449 2.402264 -0.524714

C 2.927569 2.730318 -0.723261

C 0.973323 1.175807 -1.088281

C 1.737884 0.399568 -2.108407

O -0.162710 0.805448 -0.802305

C 1.065016 -0.682889 -2.829977

H 4.500777 4.126835 -0.297952

H 3.054448 5.613046 1.063174

H 0.665881 5.043257 1.413801

H -0.276486 2.974234 0.408204

H 3.581257 2.069707 -1.291156

H 2.561059 0.891805 -2.612630

H 0.512185 -0.214520 -3.661606

H 1.790116 -1.365762 -3.284458

H 0.355263 -1.220164 -2.197551

C 1.432427 0.284792 2.410566

C 0.578056 0.505563 3.484330

C -0.410964 -0.426328 3.761625

C -0.547050 -1.552619 2.958191

C 0.291739 -1.798410 1.866010

C 1.310635 -0.858938 1.616449

C 0.081065 -3.055184 1.024218

O 0.061378 -2.592112 -0.337539

I 2.818920 -0.945850 0.049045

C 1.221027 -4.048153 1.237959

C -1.243025 -3.764032 1.287899

H 2.213212 1.012514 2.192187

H 0.689207 1.399822 4.092416

H -1.091106 -0.277577 4.596457

H -1.341730 -2.256131 3.188914

H 2.198388 -3.609593 1.018134

H 1.224177 -4.393987 2.277917

H 1.082596 -4.924026 0.591259

H -1.328904 -4.603350 0.586278

H -1.296127 -4.185717 2.297109

H -2.092269 -3.092341 1.122285

H -1.512887 1.059797 0.714856

O 4.111270 -0.366499 -2.021687

C 4.494772 -1.464190 -2.735186

H 5.126072 -1.228495 -3.615921

H 5.082790 -2.189890 -2.125547

H 3.637641 -2.071354 -3.123336

O -1.913139 1.644767 1.383382

H -0.069802 -3.360010 -0.909227

C -3.272236 1.738751 1.098771

H -3.749008 0.761386 0.950061

H -3.776213 2.253013 1.924814

C -3.512269 2.546192 -0.150702

F -3.085749 3.809420 -0.024240

F -4.815772 2.585103 -0.450814

F -2.864823 2.024245 -1.203668

C -2.753418 -1.171965 -1.340936

C -4.205092 -1.540677 -1.205245

H -2.682959 -0.298300 -2.006927

H -2.256366 -2.022297 -1.831777

F -4.925260 -0.553806 -0.650605

F -4.733658 -1.796403 -2.406497

F -4.373485 -2.626591 -0.444382

O -2.239642 -0.922681 -0.067895

H -1.299424 -1.171624 -0.109067

**2**

SCF = -538.558661819

Thermal correction to Gibbs Free Energy = 0.16035

C -2.925592 0.679216 -0.103844

C -1.733671 0.811552 0.617475

C -1.096305 2.039874 0.707887

C -1.640780 3.151516 0.068221

C -2.823026 3.030298 -0.656958

C -3.466596 1.802133 -0.740052

H -0.173354 2.133960 1.274278

H -1.141866 4.115280 0.134926

H -3.246757 3.897142 -1.157255

H -4.394544 1.722293 -1.298972

C -3.536792 -0.674238 -0.189103

C -4.904732 -0.821791 -0.859344

H -4.798032 -0.458231 -1.901502

O -2.958041 -1.654669 0.247601

C -5.378151 -2.257659 -0.870948

H -6.349234 -2.341891 -1.367822

H -4.667191 -2.893344 -1.406057

H -5.468612 -2.632466 0.153284

O -5.783903 0.033358 -0.152241

C -6.940428 0.378940 -0.876923

H -7.577254 -0.490877 -1.092660

H -7.510962 1.083226 -0.266458

H -6.684317 0.864485 -1.833106

H -1.322361 -0.070379 1.101820

**Enolonium D**

SCF = -1558.96988398

Thermal correction to Gibbs Free Energy = 0.307495

C -2.815857 0.591032 0.588671

C -2.457293 0.297214 -0.817953

C -2.724584 1.400205 -1.812888

H -3.795307 1.641845 -1.831749

H -2.185165 2.327098 -1.589865

H -2.441799 1.083658 -2.821668

O -3.232976 1.691960 0.938725

C -2.606209 -0.462849 1.653686

H -1.565235 -0.389681 2.009748

H -3.214318 -0.179666 2.523484

C -2.902876 -1.914347 1.327874

C -2.386517 -2.898564 2.332423

H -2.831443 -3.881471 2.166727

H -1.297185 -2.974156 2.212114

H -2.569283 -2.561174 3.358112

O -3.510540 -2.257766 0.331748

I -0.371974 -0.634390 -1.014130

C 0.634975 0.943066 0.088312

C 1.931918 0.608910 0.430221

C 0.026696 2.145463 0.397924

C 2.645564 1.561395 1.162597

C 0.774268 3.086496 1.103699

H -0.990610 2.376186 0.102642

C 2.076342 2.787649 1.490412

H 3.664114 1.339921 1.475716

H 0.333959 4.054211 1.332717

H 2.658058 3.518679 2.046194

C 2.478651 -0.770112 0.038888

C 3.956043 -0.683565 -0.338810

H 4.283493 -1.671843 -0.680333

H 4.098964 0.031645 -1.156399

H 4.592669 -0.390841 0.504757

C 2.305986 -1.700727 1.248936

H 2.856586 -1.337851 2.126262

H 1.246751 -1.786977 1.525849

H 2.676213 -2.700374 0.991221

O 1.783121 -1.233883 -1.065352

H -2.985711 -0.629144 -1.084619

H -3.832789 3.190154 0.107084

O -4.032230 4.136876 -0.007420

C -3.117682 4.831318 0.777809

H -3.610054 5.636556 1.340068

H -2.598953 4.186667 1.503695

C -2.064716 5.476507 -0.086528

F -1.388389 4.566475 -0.809217

F -1.168662 6.129505 0.667887

F -2.587256 6.349755 -0.948712

**Enolonium** **E**

SCF = -1558.98658826 Thermal correction to Gibbs Free Energy = 0.305506

C 1.373368 2.135122 -0.353204

C 0.435546 1.437323 0.625738

O 0.987945 3.272178 -0.721857

C 0.964316 1.339548 2.035921

H -0.537845 1.939597 0.595493

H 0.202818 0.963878 2.726231

H 1.851338 0.703468 2.087822

H 1.245501 2.351829 2.356468

C -2.389145 1.194640 -1.007703

C -3.690967 1.622718 -1.245874

C -4.750076 0.770289 -0.966645

C -4.505281 -0.507209 -0.476052

C -3.208437 -0.967884 -0.226572

C -2.166857 -0.068973 -0.474444

C -2.966245 -2.380665 0.301240

O -1.752002 -2.888938 -0.267271

I -0.096662 -0.599567 -0.088420

C -2.758624 -2.348943 1.809391

C -4.091059 -3.346839 -0.041548

H -1.562908 1.861429 -1.240606

H -3.862366 2.617054 -1.649313

H -5.773476 1.089813 -1.144668

H -5.348672 -1.165523 -0.285327

H -1.924379 -1.699374 2.094024

H -3.664095 -1.975167 2.299166

H -2.551993 -3.360740 2.175350

H -3.776552 -4.354439 0.248956

H -5.014117 -3.116905 0.499707

H -4.310849 -3.343548 -1.116175

C 2.611581 1.583758 -0.724378

C 3.110052 0.308593 -0.392531

O 2.479169 -0.589705 0.235937

C 4.512377 -0.034686 -0.827466

H 4.483109 -0.907581 -1.489643

H 5.018842 0.787074 -1.340653

H 5.100260 -0.323818 0.051003

H -0.553866 3.897183 -0.345486

O -1.416855 4.171045 0.049109

C -1.115942 4.804585 1.248916

H -0.302721 4.313616 1.809144

H -2.001773 4.825290 1.894588

C -0.687765 6.232752 1.018534

F 0.390162 6.296786 0.226789

F -0.377355 6.828034 2.178077

F -1.649717 6.956306 0.436086

H 3.234792 2.233225 -1.335673

H -1.927329 -3.106861 -1.192935

**TS2**

SCF = -1558.97144690 Thermal correction to Gibbs Free Energy = 0.303112

C -1.979599 -1.305072 0.789979

C -0.595016 -1.857356 0.499723

O -2.064787 -0.129026 1.239738

C 0.242101 -2.337665 1.635593

H -0.346891 -2.185587 -0.505085

H 1.299532 -2.413272 1.362656

H 0.126666 -1.688414 2.509595

H -0.108939 -3.340744 1.903179

C 2.032368 -1.102370 -1.852127

C 3.114562 -1.711977 -2.476238

C 4.396408 -1.447834 -2.015990

C 4.587126 -0.567176 -0.958218

C 3.523158 0.068901 -0.307844

C 2.238873 -0.241986 -0.775782

C 3.801951 1.015805 0.856866

O 2.937718 2.133791 0.667827

I 0.417773 0.482092 0.118928

C 3.505865 0.312984 2.181952

C 5.237316 1.530910 0.876711

H 1.026534 -1.292407 -2.219889

H 2.947917 -2.382836 -3.314610

H 5.257403 -1.915507 -2.486226

H 5.602770 -0.364021 -0.631632

H 2.462243 -0.010062 2.248428

H 4.149699 -0.565975 2.299767

H 3.707403 0.993964 3.019191

H 5.315402 2.297921 1.655928

H 5.964782 0.749015 1.119652

H 5.506210 1.988057 -0.081394

C -3.048449 -2.166616 0.542740

C -2.755179 -3.479338 0.118116

O -1.566548 -3.880044 0.044561

C -3.862555 -4.419799 -0.265794

H -3.777383 -5.343109 0.316892

H -4.855077 -3.987539 -0.115056

H -3.750619 -4.696487 -1.320073

H 2.932052 2.642898 1.487775

O -4.392937 0.951272 0.453084

C -3.836808 1.338913 -0.759735

H -4.618875 1.418947 -1.524563

H -3.072323 0.636375 -1.130164

C -3.181128 2.693960 -0.646090

F -4.066509 3.656941 -0.369443

F -2.564714 3.024403 -1.789686

F -2.261515 2.717451 0.330066

H -3.645346 0.573613 0.968867

H -4.064049 -1.808513 0.690287

**TS3**

SCF = -1674.628397

Thermal correction to Gibbs Free Energy = 0.352153

C 0.703247 -1.031856 -0.779711

C -0.327306 -1.897152 -1.412268

O 0.961590 0.061144 -1.273382

C -0.927079 -1.459487 -2.672943

H -0.296530 -2.952458 -1.165733

H -0.245660 -1.785113 -3.477145

H -1.881732 -1.961996 -2.855044

H -1.028147 -0.372565 -2.723595

C -0.244430 1.157201 2.133672

C 0.403026 2.300728 2.585435

C 0.214439 3.491297 1.900224

C -0.616298 3.525455 0.785723

C -1.284299 2.391686 0.308898

C -1.083189 1.191957 1.016429

C -2.264263 2.542463 -0.853639

O -2.052464 1.441539 -1.731067

I -1.957967 -0.743678 0.547142

C -3.691437 2.519953 -0.302870

C -2.061628 3.823780 -1.656880

H -0.108011 0.223330 2.677134

H 1.045741 2.254094 3.460682

H 0.711248 4.401367 2.227018

H -0.747674 4.474812 0.274827

H -3.901433 1.583388 0.224112

H -3.847803 3.351914 0.393390

H -4.413833 2.625539 -1.123216

H -2.728443 3.792046 -2.526878

H -2.314381 4.726519 -1.090582

H -1.031284 3.903463 -2.019887

H 2.137337 1.141355 -0.377437

O -2.260672 -3.042693 -0.352213

C -3.446280 -3.154340 -1.015943

H -3.664090 -4.181458 -1.371495

H -4.318488 -2.858524 -0.385508

H -3.528160 -2.488290 -1.912469

O 2.797009 1.549016 0.217597

H -2.778635 1.435602 -2.366627

C 4.008888 1.659798 -0.455162

H 3.932454 2.155766 -1.434841

H 4.703841 2.242872 0.159682

C 4.642914 0.311216 -0.689697

F 4.809377 -0.371562 0.448244

F 5.838544 0.438175 -1.272660

F 3.880174 -0.453056 -1.491962

C 1.517795 -1.511701 0.402796

H 2.531460 -1.678695 0.005889

H 1.625356 -0.661351 1.090517

C 1.133001 -2.789038 1.121927

C 1.208612 -2.743968 2.616671

H 1.087947 -3.744634 3.035870

H 2.156738 -2.303277 2.945658

H 0.409595 -2.094213 2.998694

O 0.829249 -3.797070 0.512801

**9**

SCF = -383.754877376

Thermal correction to Gibbs Free Energy = 0.097367

C -0.582390 -0.744323 0.246214

C -1.218650 -0.675074 1.481491

H -0.486741 -0.743679 2.258988

C -2.255034 -1.797084 1.677966

H -2.995571 -1.737439 0.907935

H -1.765535 -2.747302 1.629222

H -2.724083 -1.686089 2.633253

O -0.514033 -1.732980 -0.529322

C -0.016940 0.630971 0.028424

H 0.742609 0.925868 -0.665134

C -0.699805 1.416132 0.891507

C -0.391281 2.899119 1.169288

H -0.672737 3.140025 2.173104

H 0.656143 3.075731 1.040404

H -0.942885 3.511987 0.487361

O -1.786403 0.653460 1.513074

**TFE**

SCF = -452.7924547

Correction = 0.02777

H 1.239185 1.128349 -0.882489

O 1.082890 1.733555 -1.621291

C 2.034045 2.771175 -1.537943

H 3.065758 2.405781 -1.462948

H 1.947387 3.378289 -2.441885

C 1.776118 3.670764 -0.340860

F 0.569014 4.268688 -0.399685

F 2.711705 4.641916 -0.258751

F 1.814546 2.963740 0.814641

**MeOH**

SCF = -115.707363 correction = 0.028578

O -2.451293 -0.617177 -0.528182

H -2.618273 -0.859024 -1.444897

C -1.055186 -0.568748 -0.346265

H -0.868950 -0.293683 0.696362

H -0.567502 -1.537489 -0.529502

H -0.568052 0.183887 -0.983535

**Iodobenzene_alcohol**

SCF = -722.4208262

Correction = 0.140575

C -2.911691 0.655687 0.115194

C -1.614798 0.760317 0.640819

C -0.925762 1.971615 0.665228

C -1.517515 3.127467 0.170725

C -2.803699 3.061079 -0.344468

C -3.474790 1.845375 -0.367685

H 0.079559 2.015531 1.075525

H -0.971189 4.066835 0.194951

H -3.290475 3.951185 -0.734669

H -4.477786 1.821157 -0.782757

C -3.739360 -0.630263 0.087748

C -4.963678 -0.539176 -0.819303

H -5.430721 -1.530375 -0.859644

H -5.720699 0.159915 -0.447493

H -4.680487 -0.255498 -1.838610

C -4.205119 -0.972341 1.503669

H -4.785284 -1.904719 1.490843

H -3.363213 -1.100103 2.190766

H -4.854080 -0.179141 1.892320

I -0.488549 -0.862262 1.517724

O -2.889151 -1.643683 -0.434636

H -3.315838 -2.493163 -0.27092
